# Efflux-Enhanced Imidazoquinolines To Exploit Chemoresistance

**DOI:** 10.1021/acsomega.4c11297

**Published:** 2025-03-17

**Authors:** Muhammad Haroon, Sharmin Sultana, Seyedeh A. Najibi, Emily T. Wang, Abbey Michaelson, Pranto S. M. Al Muied, Amy E. Nielsen, Rock J. Mancini

**Affiliations:** † Department of Chemistry and Biochemistry, Miami University, 651 E. High Street, Oxford, Ohio 45056, United States; ‡ Astante Therapeutics Inc., 201 E. Fifth Street, Cincinnati, Ohio 45202, United States

## Abstract

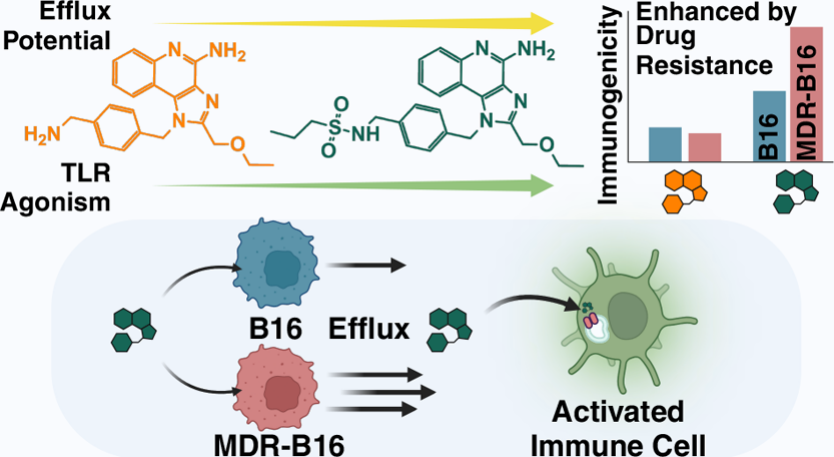

The imidazoquinoline family of toll-like receptor (TLR) immune cell agonists has long demonstrated moderate anticancer immunogenic effects by activating tumoricidal immune cells and depleting immunosuppressive cells within the tumor microenvironment. At a molecular level, we have also established that several imidazoquinolines traffic from within cancer cells to the extracellular space via P-glycoprotein (P-gp)-mediated efflux, a process commonly upregulated as multidrug-resistant (MDR) cancers acquire chemoresistance. However, imidazoquinoline P-gp efflux has never been deliberately enhanced to exploit this process. This study pioneers efforts to optimize imidazoquinoline efflux, ultimately balancing immunogenic potency alongside functional efflux susceptibility. Starting from an established imidazoquinoline scaffold previously optimized for potency, efflux was significantly enhanced by elaborating the N1 benzylic position with amide- and sulfonamide-linked P-gp affinity fragments consisting of empirically established P-gp substrates as well as computationally predicted P-gp binders. Lead compounds were identified from this series that exhibited enhanced P-gp efflux with functional retention of TLR agonism. Similar to the parent imidazoquinoline scaffold, leads had limited direct cytotoxicity in both treatment-naive and MDR B16 melanoma models and did not significantly affect the efficacy or trafficking of the chemotherapeutic doxorubicin. Efflux-enhanced imidazoquinolines were preferentially expelled from MDR-B16 cells relative to treatment-naive cells, resulting in immunogenicity that was enhanced as a consequence of the acquired MDR phenotype. Because enhanced P-gp-mediated efflux is common to most MDR cancer types, we envision that these results could inspire the design of immunotherapeutic drugs with mechanisms of action that are broadly enhanced in MDR cancers that have failed treatment or acquired resistance to chemotherapeutics.

## Introduction

Multi-drug-resistant (MDR) cancers traffic xenobiotic drugs from within the cell to the extracellular space via a range of active transport processes broadly termed drug efflux.^1,2^ Predominantly facilitated by the adenosine triphosphate binding cassette (ABC) superfamily of transport proteins, drug efflux ultimately lowers the intracellular concentration of drugs and, in the case of effluxed chemotherapeutics, results in diminished efficacy. The first discovery of at least 48 distinct ABC human transporter genes responsible for drug efflux was multidrug resistance protein 1 (MDR1), also known as ATP-binding cassette subfamily B member 1 (ABCB1) or permeability glycoprotein (P*-*gp).^3,4^ MDR cancers commonly overexpress P-gp,^5^ either innately, or as a consequence of repeated treatment with chemotherapy; this makes therapies which inhibit or act orthogonal to drug efflux particularly valuable.^6−8^

Orthogonal to the drug efflux mechanism of multidrug resistance, toll-like receptor (TLR) agonist immunotherapeutics also exhibit synergistic efficacy with chemotherapy or other types of immunotherapy like checkpoint inhibitors.^9^ At a cellular level, TLR agonists can modulate the activity of tumor-infiltrating lymphocytes^10^ and tumor-associated macrophages^11^ or deplete Gr1/CD11b myeloid-derived suppressor cells.^12^ They can also promote tumoricidal responses in CD11c dendritic cells^13^ and natural killer cells,^14^ or boost the activity of CD8^15^ and CD4^16^ T cells supporting the overall tumoricidal immune response.^17,18^

**Figure 1:**
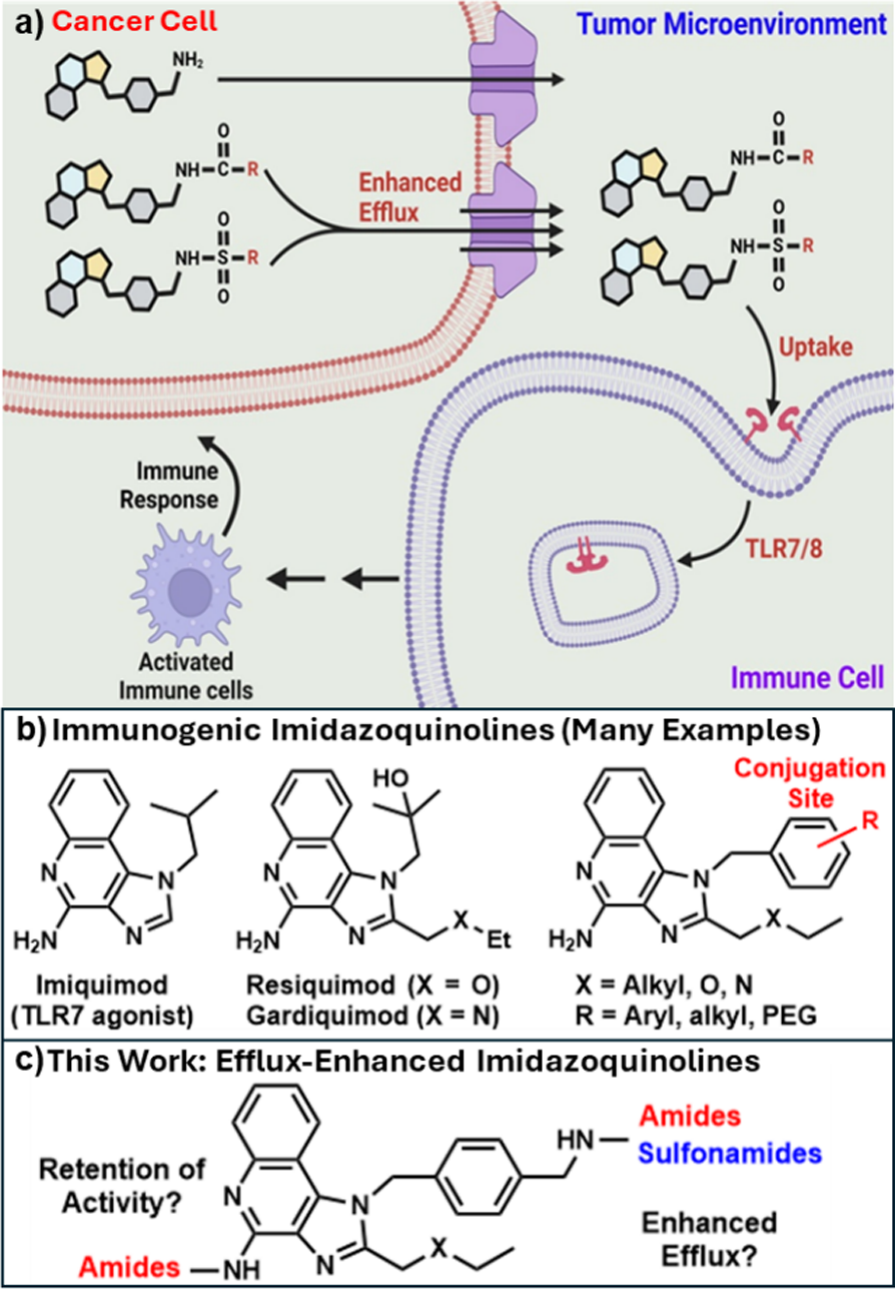
Concept of our current study. (a) By modifying the -R group (red) at the benzyl substituent on the imidazoquinoline N1 position, we created derivatives that are more susceptible to efflux with the retention of TLR7/8 agonism. (b) Some of the many established imidazoquinoline-based TLR7/8 agonists and common conjugation sites. (c) Synthetic modulation at the N1 benzylic and C4 amines to obtain efflux-enhanced amide- and sulfonamide-imidazoquinoline conjugates.

Previously, our group demonstrated highly variable P-gp efflux susceptibility among three well-established imidazoquinoline agonists of TLR7 and TLR8 (imiquimod, resiquimod, and gardiquimod).^19^ We also demonstrated that these imidazoquinolines elicited immunogenicity following efflux from MDR melanoma, breast cancer, and prostate cancer cells. Based on these findings, we envisioned that the imidazoquinoline scaffold could be enhanced to serve a dual role; first as a substrate for P-gp efflux, and second by their MDR-orthogonal action as TLR7/8 agonists once effluxed to the extracellular space (Figure 1a).

Derivatives of the imidazoquinoline scaffold have been thoroughly optimized for agonism of innate immune cells (Figure 1b),^20,21^ particularly through using structure–activity relationships to immuno-engineer cytokine profiles predictive of therapeutic anticancer immune responses.^22−26^ These effects can be enhanced with cytotoxic chemotherapeutics which create an environment of immunogenic dying cells;^27,28^ however, the precise interplay between chemotherapeutic and imidazoquinoline trafficking within, or efflux from, cancer cells remains ill-defined with some evidence suggesting these pathways might not affect each other.^29^

More specifically, imidazoquinolines have been used in contexts that require efflux from cancer cells to be effective,^30^ and this effect is enhanced by coercing intracellular accumulation when administered as glycoconjugate prodrugs.^31^ However, to the best of our knowledge, imidazoquinoline efflux susceptibility has never been deliberately enhanced (or even examined) in the setting of acquired MDR relative to a treatment-naive phenotype. As a first step to ultimately exploit chemoresistance for therapeutic benefit, here we report the first efforts to enhance imidazoquinoline susceptibility to P-gp efflux with retention of TLR agonism enhanced by the acquired MDR phenotype.

## Results and Discussion

We began our efforts to enhance imidazoquinoline efflux by examining the structure of P-gp. Owing to its high promiscuity, the P-gp structure remains limited in resolution (3.8 Å), particularly for the highly flexible regions that can audit substrates across many conformations.^32^ While this limits the reliability of predictive in silico modeling for P-gp efflux susceptibility, there remain some established empirical trends, notably, a positive correlation between topological polar surface area (tPSA) and efflux, particularly for tPSA > 90 Å^2^ and 2 or more hydrogen bond donors (HBDs).^33^ Further, placing HBDs adjacent to hydrogen bond acceptors (HBAs) also enhances efflux, as exemplified with substituted amide and sulfonamide drug conjugates. For example, the introduction of a methyl-sulfonamide has been used to confer P-gp recognition^34,35^ and the removal of a secondary sulfonamide group erased P-gp recognition.^36^ Based on this, we rationally designed 2 strategies to enhance P-gp efflux in the imidazoquinoline series.

**Scheme 1:**
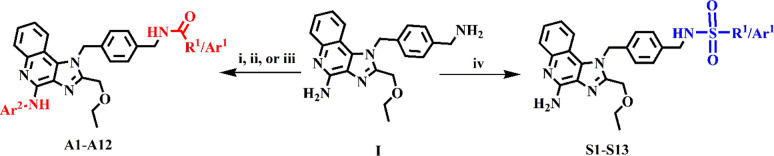
Synthetic Strategy To Elaborate the Established Imidazoquinoline Core (**I**) with Fragments for P-gp Efflux Reagents and conditions: (i) Succinic or maleic anhydride, glacial acetic acid, 3 h reflux; (ii) corresponding carboxylic acids, HBTU, TEA, DCM, 4 h room temperature; (iii) corresponding carboxylic acids, EDC/PS-750M, pyridine, DI water, 60 °C, 0.5–2 h; (iv) corresponding sulfonyl chloride, TEA, DMAP (cat.), DCM, 20–48 h room temperature.

The first strategy involved conjugating the benzylamine on established imidazoquinoline (**I**) to methyl-sulfonamide and larger systematically varied fragments (**S1–13**) to determine how the sulfonamide linkage and corresponding elaboration from this position would modulate efflux (Scheme 1). A smaller subset of amide linkages was also created for comparison of linker chemistry (**A1–5**). The second strategy involved the covalent attachment of an established P-gp ligand (coumarin) in both mono-(**A6–7**) and divalent (**A8–12**) amide attachment motifs (Figure 1c). For this latter series, we also took advantage of the large internal cavity of P-gp (∼6 nm^3^)^37^ to add a variable-length spacer, envisioning that this could help circumvent any potential interference in TLR agonism that might come from the coumarin moiety, while simultaneously increasing tPSA. From this, we performed preliminary docking predictions with P-gp (Autodock Vina) and screened our molecules through an AI model (Swiss ADME) predictive of P-gp transport (Figure S1 and Table S1). With predicted P-gp affinity confirmed, we then synthesized the imidazoquinoline conjugate library.

### Synthesis

Several investigations have previously reported the synthesis and structure–activity relationship (SAR) of imidazoquinolines as TLR7/8 agonists with the N1, C2, and C4 positions routinely optimized for potency or specific tranches of polarizing cytokines.^38,39^ Here, a commonly accepted paradigm is that preserving the primary −NH_2_ functionality at C4 is critical for TLR7/8 agonism; most C4 derivatives lose potency, and we have previously exploited this effect to modulate imidazoquinoline activity as enzyme-directed prodrugs.^40^ Many analogues developed by other groups have demonstrated that substitution at the N1 and C2 positions can alter activity as well, with the largest modifications in terms of molecular weight, tolerated at N1.^41−43^ As such, we rationalized that the well-established potent imidazoquinoline (**I**) would make a convenient starting point for efflux enhancement, as it has an ideally positioned N1 benzylamine that could serve as a synthetic handle for conjugation to P-gp affinity fragments. The synthesis of (**I**) was accomplished similarly to previously reported methods.^44−47^ Briefly, 4-hydroxyquinoline was nitrated and subsequently functionalized with Boc-protected N1 benzylamine before cyclization to the imidazoquinoline scaffold. This also afforded synthetic control over the C2 position, where we chose to use the ethyl methyl ether variant. Although this is established to retain lower, yet comparable, potency relative to alkyl C2 fragments, we wanted to prioritize solubility for some of the more hydrophobic efflux fragments. In our hands, the subsequent *N*-oxidation was significantly enhanced by pretreatment with *m*-CPBA in dichloromethane (DCM) overnight at room temperature, followed by a shorter reflux period the following day. The final stages of the synthesis involved the installation of the C4 amine through *N*-oxide rearrangement to reveal imidazoquinoline (**I**) with a primary N1 benzylamine available for further elaboration alongside the less reactive C4 amine (Scheme S1). Thus, synthesis of all derivatives began from (**I**) with the hypothesis that elaborating the N1 benzylamine would modulate susceptibility to P-gp efflux with retention of TLR7/8 activity (Table 1). Amidation of (**I**) was accomplished via ring opening of succinic or maleic anhydride (**A1**-**2**) followed by carbodiimide coupling using EDC/PS-750 M (**A3**-**7**) or HBTU (**A8**-**12**) to obtain mono- or diamidated products, respectively.^48,49^ To create the sulfonamide series, (**I**) was treated with a systematically varied selection of sulfonyl chlorides under basic reaction conditions following a previously reported method^50,51^ using anhydrous triethylamine (TEA) with catalytic DMAP in DCM to obtain the range of sulfonamides tested (**S1–13**). Yields across both conjugate series were variable and left unoptimized; however, all compounds were obtained in testable quantities.

**Table 1:**
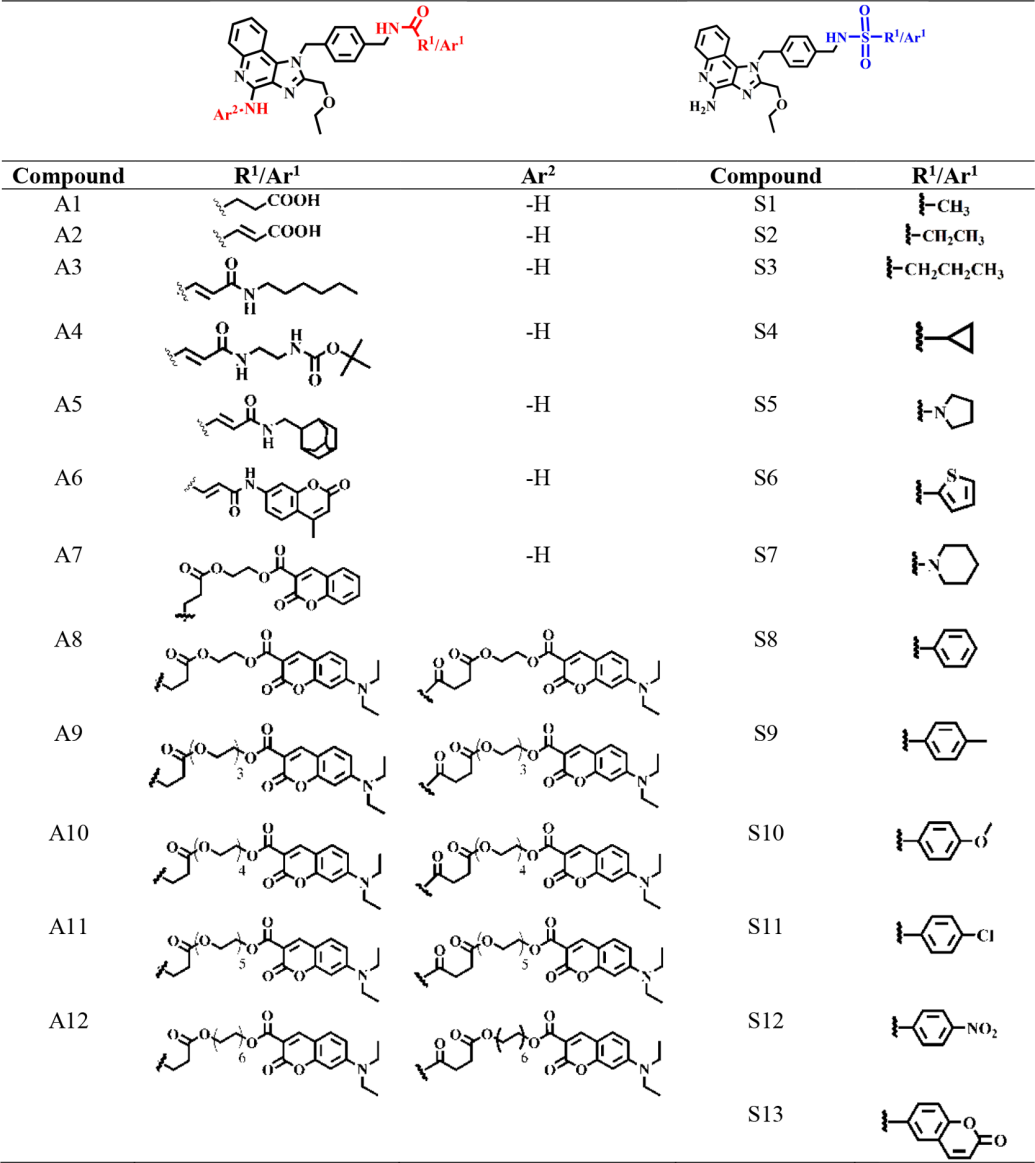
TLR7/8 Agonist Structures Synthesized by Derivatizing Scaffold (**I**)

### Performance of Imidazoquinoline Conjugates As Dual Efflux Substrates and Immunogens

**Figure 2:**
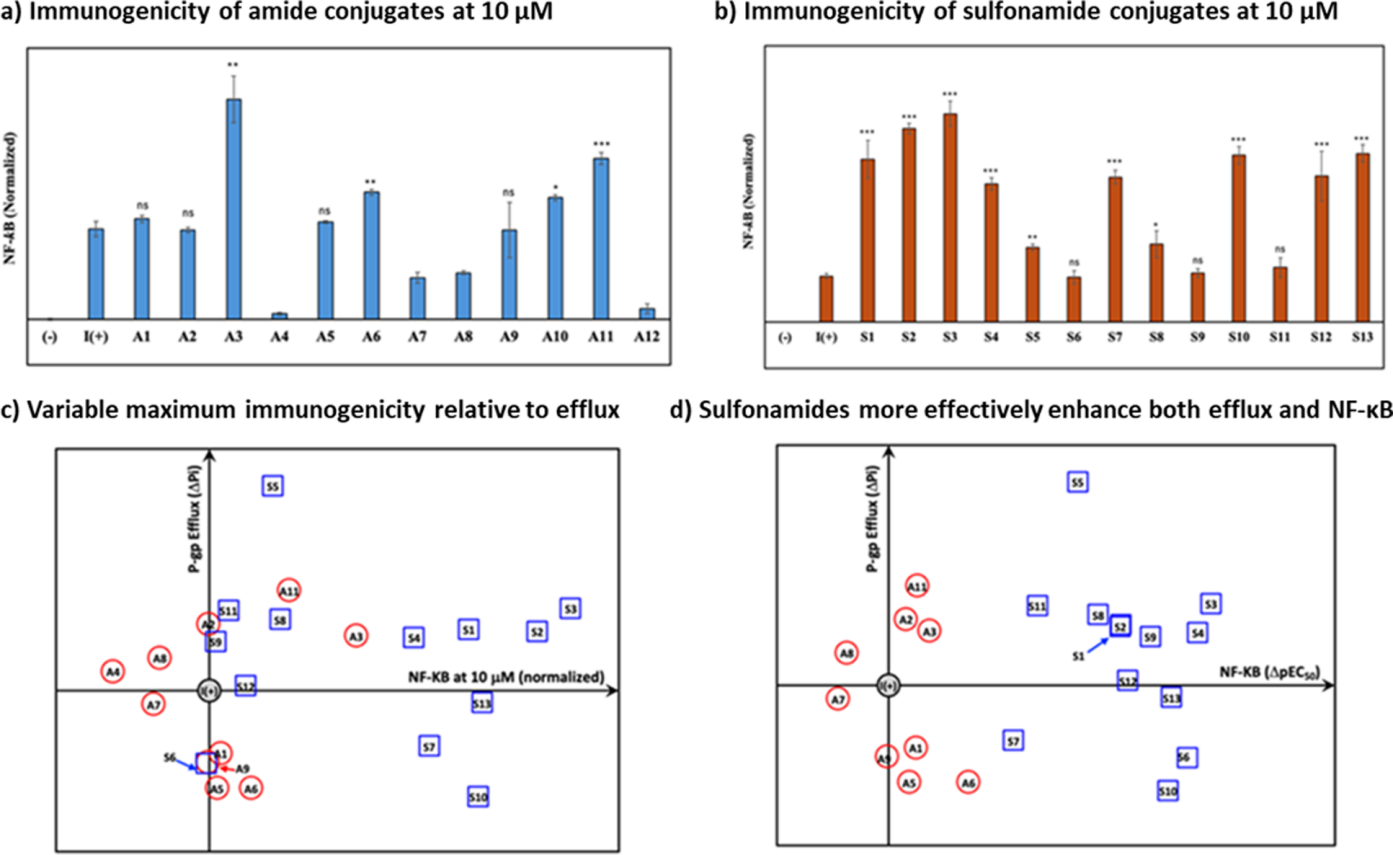
Immunogenicity and P-gp efflux susceptibility of imidazoquinoline conjugates. See the Supporting Information for standard deviations and statistical comparisons for all compounds across additional doses. NF-kB activation was measured in Raw-Blue cells by detection of the corresponding alkaline phosphatase activity using a colorimetric assay (Abs 620 nm) for (a) amide imidazoquinoline conjugates and (b) sulfonamide imidazoquinoline conjugates. Data points are the mean values of experiments repeated in triplicate and normalized relative to vehicle negative (−) and positive (+) control (**I**) at 10 μM. *p < 0.05, **p < 0.01, ***p < 0.001, ns: not significant relative to (**I**). (c) P-gp efflux susceptibility was examined by membrane vesicle assay measuring inorganic phosphorus (*P_i_*) as a consequence of ATP-dependent P-gp transport. Data points are the mean of experiments performed in triplicate. The *y*-axis is plotted as the difference (Δ*P_i_*) for each compound relative to the (+) control (**I**). The *x*-axis is the difference in potency between the indicated compound and (+) control (**I**) at 10 μM. (d) Because the sulfonamides exhibited irregular dose–response behavior, efflux susceptibility relative to (**I**) was also related to ΔpEC_50_.

Following synthesis, each compound was evaluated for retention of immunogenicity with added susceptibility to efflux, both specifically through P-gp and functionally in the efflux potential added more broadly by the MDR phenotype. First, immunological performance was assessed using a modified RAW 264.7 macrophage (RAW-Blue) reporter cell line that expresses a range of pattern recognition receptors including TLRs 1–9 (with the exception of TLR5). This cell line also had subsequent NF-κB transcription linked to an alkaline phosphatase readout, making for a convenient measure of immunogenicity. We chose this broad generalized type of immunological readout over a more specific TLR 7 or 8 assay (or multiplexed cytokine readout) for two reasons. First, we envisioned it would be possible that some structures could activate other innate immune cell receptors as would be present in the tumor microenvironment, and we wanted to capture this synergistic effect. Second, once we identified an efflux-enhanced imidazoquinoline, we envisioned that further optimization, such as the established effects of modifying the benzo ring, could be used in future studies to immunoengineer a desired cytokine profile. As such, the immunogenicity of compounds **A1–12** and **S1**-**13** was examined using RAW-Blue cells across a dose range of 10 nM to 100 μM. Normalizing RAW-Blue NF-*k*B transcription relative to the 10 μM response of the parent benzylamine (**I**) revealed that, except for some of the disubstituted coumarins, most derivatives retained the ability to activate RAW-Blue cells, with several compounds exhibiting more potent EC50s (Figures 2a,b and S2). This confirmed amide and sulfonamide linkages at the N1 benzylamine were well-tolerated, consistent with previous reports.^52−54^

Most amide-linked N1 conjugates exhibited normal dose–response behavior, albeit with diminished potency for some derivatives (**A4**, **A7**, **A8**, and **A12**). Interestingly, potency was retained for **A9–11** which has an additional coumarin moiety blocking the primary aminoquinoline C4 nitrogen traditionally thought to be critical for activity. Further, increasing linker length between the imidazoquinoline and coumarin appeared to have a modest effect on efflux susceptibility. All sulfonamide-linked conjugates retained immunogenicity, with several exhibiting significantly increased potency relative to (**I**). Notably, simple aliphatic derivatives (**S1–4**) exhibited the highest potency (lowest EC50) alongside conjugation to a known coumarin P-gp substrate (**S13**) and an abbreviated ring-activated variant (**S10**). Derivatizing with nitrogenous hydrogen bond acceptors (**S5**, **S7**) or ring-deactivated aromatics (**S11**, **S12**) was tolerated but did not increase potency as dramatically as the alkylated portion of the series. Most of the sulfonamide conjugates also exhibited an irregular NF-*k*B dose–response, particularly for higher concentrations (Figure S2). Initially, we thought that this could be due to loss of cell viability; however, we subsequently confirmed minimal cytotoxicity for all derivatives in RAW-Blue cells (Figure S3). At present, we suspect that this is due to the endosomal acidification required for intracellular TLR signaling as the sulfonamides could buffer this, particularly at the higher concentrations where we observe the effect. However, future studies are needed to conclusively determine the mechanism.

We also looked for any relationship between cLogP, HBA/HBD, or tPSA with immunogenicity (NF-*k*B) for both series of compounds (Table S2). Here we observed a correlation between HBA/HBD groups and potency for both sulfonamide- and amide-based compounds consistent with previously established effects.^17,55,56^ We also found increases in maximum NF-*k*B with increased cLogP for some of the PEGylated coumarin derivatives (**A8**, **A9**, **A10**, and **A11**), although this could alternatively be explained by the increased linker length ameliorating interference between the coumarin and imidazoquinoline as this also appeared to enhance efflux susceptibility. While some other weak correlations were observed, overall, the amide-linked conjugates provided a more regular NF-*k*B dose–response, whereas sulfonamide-based compounds retained superior potency at lower concentrations. Regardless, efflux significantly affects extracellular concentrations within the tested ranges for both series and could therefore result in differences between intrinsic immunogenicity relative to immunogenicity following efflux. As such, all compounds were subsequently evaluated for efflux susceptibility.

### Imidazoquinoline Susceptibility to P-gp Efflux

Because P-gp is highly promiscuous with regard to substrate scope, we reasoned that imidazoquinolines susceptible to efflux, specifically through P-gp, had the best likelihood of also being expelled from a broad range of MDR cancers that overexpress P-gp alongside other transport proteins. To test this, we first compared the P-gp efflux susceptibility among our imidazoquinoline conjugates using a membrane vesicle assay. The assay operates by incubating potential substrates with P-gp vesicles and ATP followed by colorimetric detection of the inorganic phosphate (*P_i_*) liberated as a consequence of ATP hydrolysis during the active transport process. To compare imidazoquinoline conjugates as P-gp substrates, efflux studies were performed under saturating active transport conditions (10 μM imidazoquinoline substrate, pH 7.4, 37 °C) with the total amount of effluxed imidazoquinoline measured as *P_i_* after 10 min. To confirm the pathway, we also ran parallel experiments using imidazoquinoline substrates and an inhibitor (sodium orthovanadate). Generally, we found that imidazoquinoline conjugates were substrates of P-gp with variable susceptibility relative to their performance as TLR agonists (Figure 2c), and similar to previous reports, did not correlate well with predicted binding. Due to the variable dose–response curves, we also crossed this readout with ΔpEC_50_ relative to (**I**). This revealed a distinct pattern where sulfonamides were more potent immunogens, but both libraries had variable, conjugate-dependent efflux susceptibility (Figure 2d). Here sulfonamide **S5** was the strongest P-gp substrate and **S3** was the most potent in terms of ΔpEC_50_ relative to (**I**). **A11** exhibited the best efflux performance out of the amide library. While this membrane vesicle assay is a precise measure of ATP hydrolysis due to P-gp-substrate interactions, it also presents some key limitations. Namely, this assay is not entirely predictive of how efflux will occur in aggregates, such as in the more complex system of transport across an MDR cancer cell membrane. As such, we were next interested in testing if efflux-enhanced imidazoquinolines (1) inhibited the efflux of chemotherapeutics or (2) themselves caused immunogenicity following efflux from MDR cancer cells that overexpress P-gp.

### Imidazoquinolines Do Not Inhibit Doxorubicin Efflux

**Figure 3:**
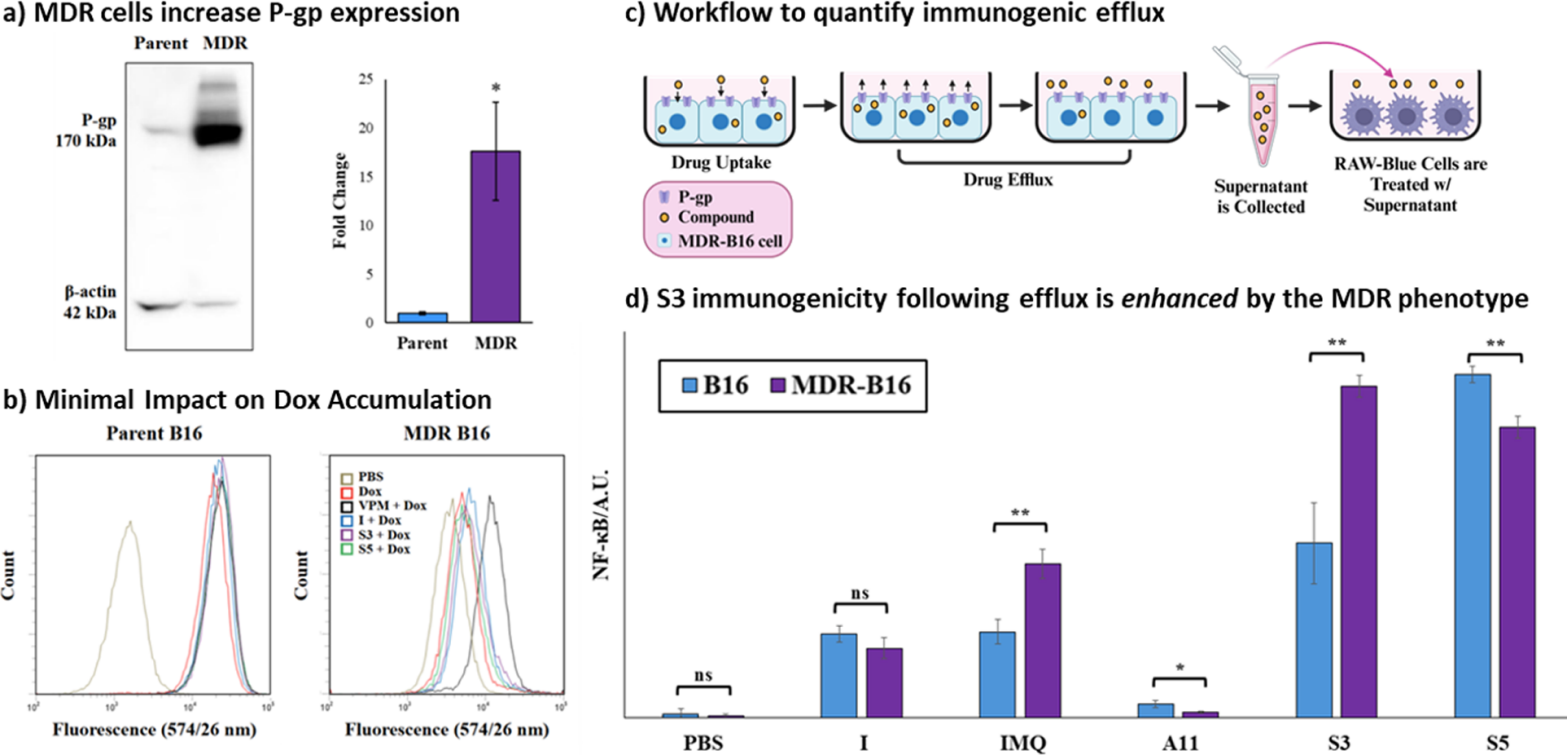
(a) Western blot detection of P-gp in parent and MDR-B16 cell lysates. Fold changes are represented as mean values with error bars as ± standard deviation for triplicate experiments. *p < 0.05, for P-gp expression in MDR-B16 relative to parent cell line. (b) Flow cytometry histograms of MDR-B16 and B16 cells treated with 50 μM of compounds **S3**, **S5**, and **I**, or 10 μM of the P-gp inhibitor verapamil (VPM) for 16 h, followed by 10 μM Dox treatment for 30 min. The histogram represents the fluorescence intensity of Dox within the cells. (c) Workflow for measuring efflux of most potent compounds from MDR-B16 cells by loading cells with compounds and subsequently measuring efflux by immunogenic readout. (d) Activation of RAW-Blue cells following efflux after 2 h. NF-*k*B activation was determined by measuring SEAP using a QUANTI-Blue Solution. Data are represented as mean ± SD (*n* = 3). *p < 0.05, **p < 0.01 for **IMQ**, **I**, **A11**, **S3,** and **S5**, comparing MDR-B16 cells to B16-cells. ns: nonsignificant.

Based on the observation that all derivatives in our catalogue retained some susceptibility to P-gp efflux, we next examined if these same molecules might competitively inhibit doxorubicin (Dox) efflux in an MDR cancer cell line, where P-gp efflux otherwise enables cell survival by reducing the intracellular accumulation of the drug. If true, this would imply that imidazoquinolines could resensitize MDR cancers to chemotherapy by acting as a dual efflux inhibitor and immunogen. To test this, we examined the effect of all compounds to restore Dox-mediated cytotoxicity in an in vitro MDR melanoma model. We chose the B16 melanoma cell line because it is a highly immunogenic cancer expressing numerous tumor-associated antigens (gp100^57^ and Trp2^58^) that respond to intratumoral TLR stimulation and have the capacity to stimulate strong immune responses.^59−62^ To create the model, we established a doxorubicin-resistant MDR-B16 melanoma cell line, and following confirmation of abundant P-gp expression (Figure 3a), we compared imidazoquinoline performance in MDR-B16 cells relative to treatment-naive cells.

Initially, we tested for both inherent imidazoquinoline cytotoxicity, as well as resensitization to Dox via efflux pump inhibition using cell viability assays (Figures S4 and S5) and flow cytometry to look at Dox accumulation (Figures 3b and S6). While some of the imidazoquinoline conjugates had an innate, yet modest, cytotoxicity consistent with previous reports,^63^ none effectively resensitized MDR-B16 cells to the cytotoxic action of Dox except for a few of the sulfonamides at higher than clinically relevant (50 μM) concentrations. This was consistent with the flow cytometry readouts that indicated that imidazoquinoline conjugates caused only minimal enhancements in Dox retention compared to the P-gp inhibitor verapamil.

### Imidazoquinolines Exhibit Enhanced Immunogenicity with Multidrug-Resistant Melanoma

Despite a lack of competitive inhibition with Dox, we still envisioned that our MDR cancer cell line could enhance immunogenicity by increasing the trafficking of intracellular imidazoquinoline to the extracellular space. This mechanism could be particularly significant because it would indicate imidazoquinoline variants that could be *more* effective when intracellularly delivered to cancers that have acquired MDR (and associated drug efflux proteins). Therefore, we next examined if immunogenicity would be retained following efflux from cancer cells (Figure 3c), and if this process would result in enhanced immunogenicity under conditions of acquired drug resistance (Figure 3d). Here, we observed that MDR melanoma cells loaded with either the most potent (**S3**) or most efflux susceptible (**S5**) sulfonamide conjugates provided stronger immunogenicity following efflux relative to the previously established P-gp substrate Imiquimod (**IMQ**) or the parent scaffold (**I**). Comparing the treatment-naive and MDR cell lines, differential immunogenicity *enhanced* by the MDR phenotype was observed for both **IMQ** and **S3**. While MDR-enhanced immunogenicity was not observed for **S5**, the overall activation was stronger in both cell lines even though **S5** was less potent than **S3** when directly added to immune cells. We also attribute this apparent increase in potency to the enhanced efflux susceptibility of **S5**. Activation, but no differential immunogenicity was observed for **I**, and the **A11** efflux supernatant from either cell type provided only minimal activation. This could be due to the cLogp of **A11** or the sterically bulky coumarin groups attached to it. In contrast, **S3** and **S5** are much smaller with more drug-like features. Regardless, this assay indicates that both **S3** and **S5** are more potent activators of NF-κB following efflux from MDR melanoma relative to **I** or the known efflux substrate **IMQ**.

## Conclusions

In this study, we developed a small library of 25 imidazoquinolines conjugated to P-gp affinity fragments via amide and sulfonamide linkages. Most of the synthesized molecules had *c*Log*P* values within a drug-like range (2 < *c*Log*P* < 5), except for some of the amide-linked compounds (**A8–12**). Out of the library, 17 conjugates activated RAW-Blue macrophages, while a membrane vesicle assay demonstrated that all conjugates are P-gp substrates of variable susceptibility with **S5** being the most susceptible. That said, susceptibility to efflux correlated poorly with prediction (Figure S7), indicating an ongoing need to produce these types of compounds to empirically determine efflux.

Weighting efflux susceptibility and immunogenicity equally, **S3** and **S5** from the sulfonamide series were selected for testing in a dual efflux-TLR agonism assay alongside the most promising, but less potent, candidate from the amide series (**A11**). Here, **S3** demonstrated differential activation with immunogenicity *enhanced* by the MDR phenotype. That said, developing a prodrug or drug delivery vehicle for this lead will likely require adding weight to efflux susceptibility, as the differential response between cell lines observed for **S3** suggests this is the rate-limiting step in trafficking from cancer cells. As such, it is perhaps unsurprising that the optimal efflux substrate **S5** also provided the most immunogenicity following efflux from either cell line even though its potency was lower when directly added to RAW-Blue cells. In the future, we envision this concept of efflux-enhanced imidazoquinolines could be combined with prodrug delivery strategies designed to enhance uptake, both of which could prove uniquely effective in exploiting common phenotypes presented by MDR cancers.

## Experimental Section

### Material and Methods

For chemical synthesis, all reagents and solvents were of high purity (ACS grade or better) and, except where noted, used as received without further purification. Reagents and suppliers include: Phosphorus oxychloride (POCl_3_) (Bean Town Chemical), anhydrous magnesium sulfate (JT Baker), 4-hydroxyquinoline, 1-(*N*-Bocaminomethyl)-4-(aminomethyl)benzene, (2-ethoxy)acetyl chloride, 2-oxo-2*H*-chromene-3-carboxylic acid, 7-(diethylamino)-2-oxo-2*H*-chromene-3-carboxylic acid (Ambeed), triethylamine, 5.4 M sodium methoxide solution in MeOH, ethylene glycol, triethylene glycol, tetraethylene glycol, dodecane-1,12-diol (TCI), 10% palladium on carbon, 3-chloroperbenzoic acid, benzoyl isocyanate, trifluoroacetic acid, glacial acetic acid (Sigma-Aldrich), maleic anhydride, *n*-hexyl amine (Acros), succinic anhydride, *tert*-butyl 2-aminoethylcarbamate, pentaethylene glycol (Aaron chemicals), 1-adamantanemethylamine (aablocks), 7-amino-4-methyl-2*H*-chromen-2-one (Apex chemicals), 4-dimethyl aminopyridine (Merck), EDC HCl (Acrotein). The remaining solvents were purchased from VWR. Reaction progress was monitored by using F254 TLC plates (Silicylce). Proton (^1^H−) and carbon (^13^C−) NMR spectra were obtained using Bruker 400 and 500 MHz NMR spectrometers. Electrospray ionization mass spectrometry (ESI/MS) was obtained on an Agilent Single-Quad LCMS G6125B.

In the biological assays, the following biologics, chemicals, and instruments were used: B16–F10–Luc2 (ATCC), RAW-Blue 264.7 macrophages (InvivoGen, USA), high-glucose (4.5 g/L) Dulbecco’s modified Eagle’s medium (DMEM) (Corning), heat-inactivated fetal bovine serum (FBS) (VWR), resazurin (R&D Biosystems), sterile, nontreated, flat bottomed 96-well plates (VWR), blasticidin, normocin, zeocin (InvivoGen), doxorubicin (Broadpharm), verapamil (Ambeed), trypsin-EDTA solution (Thermo Fisher Scientific), P-gp antibody (rabbit monoclonal), β-actin, and secondary antibody goat antirabbit conjugated to HRP (Abcam). Samples were centrifuged using a refrigerated centrifuge (Eppendorf). Labnet VX-200 S0200 Low Profile Lab Vortex (Labnet International) was used for the thorough mixing of samples. Cells were counted and examined by using an Olympus microscope (Model XYZ, Olympus Corporation, Japan). PAGE and Western Blots were run on an apparatus from BioRad with antibodies purchased from Abcam. Subsequent imaging was performed on a Biorad ChemiDoc MP Imaging System. Flow cytometry histograms were obtained with an Attune N×T flow cytometer (Thermo Fisher Scientific).

### Synthesis of Starting Imidazoquinoline **I**

Imidazoquinoline **I** was synthesized similarly to Wilson et al.^46^ and is further described in Scheme S1 (see Supporting Information).

### General Method for the Synthesis of **A1**–**2**

To a dry round-bottom flask equipped with a septum and a magnetic stir bar was added **I** (60 mg, 0.16 mmol) and respective anhydrides (0.24 mmol) in 5 mL of glacial acetic acid. The reaction mixture was stirred at room temperature for 3 h before removal of acetic acid by azeotropic vacuum distillation with toluene (2 × 10 mL) to provide a viscous liquid. Water (10 mL) was added to the obtained residue, and the precipitated product was filtered, dissolved in DCM, and washed with water, before drying over magnesium sulfate and evaporating the solvent via rotavap to obtain pure **A1–A2**.

#### (**A1**)

Succinic anhydride: 24.93 mg, 0.24 mmol; Yield: 44.30%; *R*_f_: 0.12 (DCM: MeOH; 90:10); ^1^H NMR (400 MHz, DMSO-*d*_6_): δ 10.87 (s, 1H, −COOH), 8.31 (t, 1H, −NH–CH_2_–, *J* = 5.4 Hz), 8.01 (d, 1H, Ar–H, *J* = 8.4 Hz), 7.93 (d, 1H, Ar–H, *J* = 8.2 Hz), 7.57 (t, 1H, Ar–H, *J* = 7.7 Hz), 7.39 (t, 1H, Ar–H, *J* = 7.7 Hz), 7.12 (d, 2H, Ar–H, *J* = 7.8 Hz), 6.95 (d, 2H, Ar–H, *J* = 7.9 Hz), 5.93 (s, 2H, −CH_2_−), 4.73 (s, 2H, −CH_2_−), 4.13 (d, 2H, −CH_2_–NH–, *J* = 5.8 Hz), 3.42 (q, 2H, -OCH_2_–CH_3_, *J* = 6.9 Hz), 2.32 (t, 2H, −CH_2_–CH_2_–, *J* = 6.5 Hz), 2.25 (t, 2H, −CH_2_–CH_2_–, *J* = 6.3 Hz), 0.90 (t, 3H, −CH_3_ ether, *J* = 6.9 Hz); ^13^C NMR (100 MHz, DMSO-*d*_6_): δ 171.7 (−COOH), 151.6, 139.3, 135.8, 135.1, 132.4, 129.6, 128.9, 128.6, 127.8, 126.0, 121.4 (Ar–C), 66.0, 64.6 (2x-OCH_2_−), 49.0 (−CH_2_−), 41.9 (−CH_2_–NH−), 30.7, 30.0 (2x-CH_2_−), 15.2 (−CH_3_ ether); ESI/MS (*m*/*z*) for C_25_H_27_N_5_O_4_: [M + H]^+^_theoretical_: *m*/*z* 462.2, [M + H]^+^_found_: *m*/*z* 462.2.

#### (**A2**)

Maleic anhydride: 24.43 mg, 0.24 mmol; Yield: 49.79%; *R*_f_: 0.10 (DCM: MeOH; 90:10); ^1^H NMR (400 MHz, DMSO-*d*_6_): δ 9.29 (t, 1H, −NH–CH_2_–, *J* = 5.8 Hz), 7.84 (d, 1H, Ar–H, *J* = 8.2 Hz), 7.68 (d, 1H, Ar–H, *J* = 8.3 Hz), 7.51 (t, 1H, Ar–H, *J* = 7.6 Hz), 7.24 (t, 1H, Ar–H, *J* = 7.6 Hz), 7.18 (d, 2H, Ar–H, *J* = 7.9 Hz), 7.0 (d, 2H, Ar–H, *J* = 7.9 Hz), 6.31 (d, 1H, −CH=CH–, *J* = 12.3 Hz), 6.16 (d, 1H, −CH=CH–, *J* = 12.4 Hz), 5.90 (s, 2H, −CH_2_−), 4.75 (s, 2H, −CH_2_−), 4.26 (d, 2H, −CH_2_–NH–, *J* = 5.7 Hz), 3.42 (q, 2H, -OCH_2_–CH_3_, *J* = 6.9 Hz), 0.88 (t, 3H, −CH_3_ ether, *J* = 6.9 Hz); ^13^C NMR (100 MHz, DMSO-*d*_6_): δ 166.3 (−COOH), 165.6 (C=O), 137.9, 135.0, 128.3, 126.2, 125.3, 122.3 (Ar–C), 131.8, 132.6 (−CH=CH−), 66.1, 64.3 (2x-OCH_2_−), 49.2 (−CH_2_−), 42.5 (−CH_2_–NH−), 15.1 (−CH_3_ ether); ESI/MS (*m*/*z*) for C_25_H_25_N_5_O_4_: [M + H]^+^_theoretical_: *m*/*z* 460.1, [M + H]^+^_found_: *m*/*z* 460.1.

### General Method for the Synthesis of **A3**–**7**

Compounds **A3**-**7** were synthesized following a reported method^48^ with slight modifications. In an oven-dried round-bottom flask, respective carboxylic acids (for amount see individual compound data), **I** (60.00 mg, 0.16 mmol), EDC HCl (35 mg, 1.1 equiv), and pyridine (0.013 mL, 1.0 equiv) were added in 10 mL of deionized water and stirred for 10 min at 60 °C. A catalytic amount of PS-750 M (0.1 mL) was added and the reaction mixture was stirred under heat until a solid precipitated. The solid was filtered and washed with excess deionized water. Residual water was subsequently lyophilized to obtain a pure compound.

#### (**A3**)

4-(Hexylamino)-4-oxobut-2-enoic acid: 33.00 mg, 0.16 mmol; Duration: 2 h; Yield: 54.21%; *R*_f_: 0.37 (DCM: MeOH; 90:10); ^1^H NMR (400 MHz, DMSO-d6): δ 9.63 (t, 1H, −NH–CH_2_–, *J* = 5.6 Hz), 9.02 (t, 1H, −NH–CH_2_–, *J* = 5.2 Hz), 7.80 (d, 1H, Ar–H, *J* = 8.2 Hz), 7.60 (d, 1H, Ar, *J* = 8.2 Hz), 7.37 (t, 1H, Ar–H, *J* = 7.6 Hz), 7.26–7.20 (m, 2H, Ar–H), 7.10–7.00 (m, 3H, Ar–H), 6.39–6.32 (m, 1H, −CH=CH−), 6.26–6.21 (m, 1H, −CH=CH−), 5.91 (s, 2H, −CH_2_−), 4.78 (s, 2H, −CH_2_−), 4.34–4.26 (m, 2H, −CH_2_–NH−), 3.49 (q, 2H, -OCH_2_–CH_3_, *J* = 7.2 Hz), 3.08 (q, 2H, −NH–CH_2_–CH_2_–, *J* = 6.7 Hz), 1.27–1.24 (m, 8H, 4x-CH_2_−), 0.98 (t, 3H, −CH_3_ ether, *J* = 6.9 Hz), 0.87–0.81 (m, 3H, −CH_3_ hexyl chain); ^13^C NMR (100 MHz, DMSO-d6): δ 165.1 (C=O), 164.9 (C=O), 134.2, 132.0, 128.3, 128.1, 126.6, 126.1, 121.2 (Ar–C), 65.8, 64.5 (2x-OCH_2_−), 48.8 (−CH_2_−), 40.6 (−CH_2_–NH−), 39.1, 31.3, 29.1, 26.5, 22.4, 14.3 (hexyl chain carbons), 15.2 (−CH_3_, ether); ESI/MS (*m*/*z*) for C_31_H_38_N_6_O_3_: [M + H]^+^_theoretical_: *m*/*z* 543.3; [M + H]^+^_found_: *m*/*z* 543.3.

#### (**A4**)

4-(2-(*tert*-Butoxycarbonyl)ethylamino)-4-oxobut-2-enoic acid: 42.00 mg, 0.16 mmol; Duration: 45 min; Yield: 30.43%; *R*_f_: 0.29 (DCM: MeOH; 90:10); ^1^H NMR (400 MHz, DMSO-d6): δ 10.93 (s, 1H, −NH), 9.44 (t, 1H, −NH–CH_2_–, *J* = 5.2 Hz), 8.83 (t, 1H, −NH–CH_2_–, *J* = 4.9 Hz), 8.01 (d, 1H, Ar–H, *J* = 8.2 Hz), 7.67–7.63 (m, 1H, Ar–H), 7.60–7.56 (m, 1H, Ar–H), 7.46 (t, 1H, Ar–H, *J* = 7.6 Hz), 7.25 (d, 2H, Ar–H, *J* = 8.0 Hz), 7.06 (d, 2H, Ar–H, *J* = 8.0 Hz), 6.15 (d, 1H, −CH=CH–, *J* = 12.8 Hz), 6.11 (d, 1H, −CH=CH–, *J* = 12.8 Hz), 6.01 (s, 2H, −CH_2_−), 4.81 (s, 2H, −CH_2_−), 4.30 (d, 2H, −CH_2_–NH–, *J* = 5.7 Hz), 3.49 (q, 2H, -OCH_2_–CH_3_, *J* = 6.7 Hz), 3.13–3.09 (m, 2H, −CH_2_–CH_2_−), 3.04–3.01 (m, 2H, −CH_2_–CH_2_−), 1.33 (s, 9H, −C(CH_3_)_3_), 0.97 (t, 3H, −CH_3_ ether, *J* = 6.9 Hz); ^13^C NMR (100 MHz, DMSO-d6): δ 165.6 (C=O), 164.9 (C=O), 156.0 (C=O), 151.5, 138.6, 137.8, 135.8, 135.4, 132.5, 129.3, 128.9, 128.6, 128.6, 128.2, 126.1, 125.7 (Ar–C), 131.2, 132.6 (−CH=CH−), 78.0 (−C(CH_3_)_3_), 66.0, 64.6 (−OCH_2_−), 49.0 (−CH_2_−), 42.2, 39.2, 21.5 (3x-CH_2_–NH−), 28.6 (−C(CH_3_)_3_), 15.1 (−CH_3_, ether).

#### (**A5**)

4-Oxo-4-[(adamantylmethyl)amino]but-2-enoic acid: 45.00 mg, 0.16 mmol; Duration: 1 h; Yield: 58.75%; *R*_f_: 0.31 (DCM: MeOH; 90:10); ^1^H NMR (400 MHz, DMSO-d6): δ 8.84 (t, 1H, −NH–CH_2_–, *J* = 5.8 Hz), 8.22 (t, 1H, −NH–CH_2_–, *J* = 6.2 Hz), 7.79 (d, 1H, Ar–H, *J* = 8.2 Hz), 7.58 (d, 1H, Ar–H, *J* = 8.2 Hz), 7.36 (t, 1H, Ar–H, *J* = 7.8 Hz), 7.19 (d, 2H, Ar–H, *J* = 8.0 Hz), 7.07 (d, 1H, Ar–H, *J* = 7.7 Hz), 7.03 (d, 2H, Ar–H, *J* = 8.0 Hz), 6.94 (d, 1H, −CH=CH–, *J* = 15.2 Hz), 6.83–6.79 (3H, m, −CH=CH– and −NH_2_), 5.90 (s, 2H, −CH_2_−), 4.77 (s, 2H, −CH_2_−), 4.31 (d, 2H, −CH_2_–NH–, *J* = 5.7 Hz), 3.48 (q, 2H, −CH_2_–, *J* = 6.7 Hz), 2.86 (d, 2H, −CH_2_–NH–, *J* = 6.2 Hz), 1.91 (s, 3H, adamantyl-H), 1.66–1.55 (m, 6H, adamantyl-H), 1.42 (s, 6H, adamantyl-H), 0.97 (t, 3H, −CH_3_, *J* = 6.7 Hz); ^13^C NMR (100 MHz, DMSO-d6): δ 164.3 (C=O), 152.3, 138.5, 135.6, 133.7, 132.6, 128.0, 126.7, 126.1, 121.1 (Ar–C), 65.85, 64.57 (2x-OCH_2_– ether), 50.84 (−CH_2_−), 48.84 (−CH_2_−), 42.34 (−CH_2_–NH−), 40.6, 36.4, 34.2, 28.1 (adamantyl-C), 15.2 (−CH_3_ ether); ESI/MS (*m*/*z*) for C_36_H_42_N_6_O_3_: [M + H]^+^_theoretical_: *m*/*z* 607.4, [M + H]^+^_found_: *m*/*z* 607.4.

#### (**A6**)

4-(4-Methyl-2-oxo-2*H*-chromen-7-ylamino)-4-oxobut-2-enoic acid: 45.00 mg, 0.16 mmol; Duration: 40 min; Yield: 49.86%; *R*_f_: 0.34 (DCM: MeOH; 90:10); ^1^H NMR (400 MHz, DMSO-*d*_6_): δ 11.20 (s, 1H, −NH), 8.88 (t, 1H, −NH–CH_2_–, *J* = 5.8 Hz), 8.02 (s, 1H, coumarin ring-H), 7.74–7.71 (m, 2H, coumarin ring-H), 7.66–7.63 (m, 1H, Ar–H), 7.51 (d, 1H, Ar–H, *J* = 8.3 Hz), 7.39–7.34 (m, 1H, Ar–H), 7.29 (t, 1H, Ar–H), 7.18 (d, 2H, Ar–H, *J* = 7.8 Hz), 6.96 (d, 2H, Ar–H, *J* = 8.1 Hz), 6.26 (d, 1H, −CH=CH–, *J* = 8.5 Hz), 6.20 (d, 1H, −CH=CH–, *J* = 7.3 Hz), 5.84 (s, 2H, −CH_2_−), 4.70 (s, 2H, −CH_2_−), 4.23 (d, 2H, −CH_2_–NH–, *J* = 5.6 Hz), 3.41 (q, 2H, −O–CH_2_CH_3_, *J* = 6.8 Hz), 2.33 (s, 3H, −CH_3_), 0.89 (t, 3H, −O–CH_2_CH_3_, *J* = 6.9 Hz); ^13^C NMR (100 MHz, DMSO-d6): δ 165.0 (C=O), 164.7 (C=O), 160.4 (C=O), 154.1, 153.6, 153.5, 152.1, 150.2, 142.6, 138.4, 135.6, 134.2, 132.8, 131.2, 129.9, 128.7, 128.2, 127.6, 126.4, 126.1, 121.2, 115.7, 114.6, 112.8, 112.6, 106.1 (coumarin+Ar–C), 65.8, 64.5 (−OCH_2_– ether), 48.8 (−CH_2_−), 42.2 (−CH_2_–NH−), 18.4 (coumarin-CH_3_), 15.1 (−CH_3_ ether); ESI/MS (*m*/*z*) for C_35_H_32_N_6_O_5_: [M + H]^+^_theoretical_: *m*/*z* 617.2, [M + H]^+^_found_: *m*/*z* 617.2.

#### (**A7**)

4-Oxo-4-(2-(2-oxo-2*H*-chromene-3-carbonyloxy)ethoxy)butanoic acid: 53.00 mg, 0.16 mmol; Duration: 2 h; Yield: 51.21%; *R*_f_: 0.27 (DCM: MeOH; 90:10); ^1^H NMR (400 MHz, DMSO-*d*_6_): δ 8.74 (s, 1H, coumarin ring-H), 8.34 (t, 1H, −NH–CH_2_–, *J* = 5.8 Hz), 8.21–8.10 (m, 2H, −NH_2_), 8.00 (d, 1H, Ar–H), 7.90 (d, 1H, Ar–H), 7.72 (d, 1H, Ar–H, *J* = 8.2 Hz), 7.63 (t, 1H, Ar–H, *J* = 6.0 Hz), 7.59–7.55 (m, 2H, coumarin ring-H), 7.42 (d, 1H, coumarin ring-H, *J* = 8.2 Hz), 7.35 (t, 1H, coumarin ring-H, *J* = 7.4 Hz), 7.14 (d, 2H, Ar–H, *J* = 8.0 Hz), 7.01 (d, 2H, Ar–H, *J* = 8.0 Hz), 5.99 (s, 2H, −CH_2_−), 4.81 (s, 2H, −CH_2_−), 4.44–4.41 (m, 2H, −CH_2_−), 4.33–4.31 (m, 2H, −CH_2_−), 4.16 (d, 2H, −CH_2_–NH–, *J* = 5.8 Hz), 3.49 (q, 2H, −O–CH_2_CH_3_, *J* = 6.9 Hz), 2.55 (t, 1H, −CH_2_–CH_2_–, *J* = 6.6 Hz), 2.41 (t, 2H, −CH_2_–CH_2_–, *J* = 6.6 Hz), 0.97 (t, 3H, −O–CH_2_CH_3_, *J* = 6.9 Hz); ^13^C NMR (100 MHz, DMSO-d6): δ 172.8 (C=O), 172.4 (C=O), 171.1 (C=O), 162.6 (C=O), 156.3, 155.0, 149.5, 139.1, 135.2, 135.0, 132.4, 130.8, 128.9, 128.6, 127.8, 126.0, 125.2, 118.2, 117.6, 116.6 (coumarin+Ar–C), 66.0, 64.6, 63.5, 62.1 (4x-O–CH_2_−), 49.0 (−CH_2_−), 42.0 (−CH_2_–NH−), 30.1, 29.3 (2x-CH_2_−), 15.1 (−CH_3_ ether); ESI/MS (*m*/*z*) for C_37_H_35_N_5_O_8_: [M]^−1^_theoretical_: *m*/*z* 624.2, [M]^−1^_found_: *m*/*z* 624.2.

### General Method for the Synthesis of **A8**–**12**

Compounds **A8**–**12** were synthesized from a previously reported method^63^ with slight modifications. In a dry round-bottom flask, DCM (10 mL) was added followed by cooling on ice to 0 °C before the addition of respective carboxylic acids (see below) and triethylamine (0.05 mL, 2.0 equiv). The resulting mixture was stirred for 15 min before the addition of HBTU (120 mg, 2.0 equiv). The reaction mixture was further warmed to room temperature and stirred for 4 h. Solvent was removed by rotavap. The crude product was suspended in deionized water and extracted with DCM before further purification by column chromatography (DCM: MeOH; 99:1). Fractions were collected and the solvent was removed by rotavap before lyophilization from benzene to obtain a pure product.

#### (**A8**)

4-(2-(7-(Diethylamino)-2-oxo-2*H*-chromene-3-carbonyloxy)ethoxy)-4-oxobutanoic acid: 64.00 mg, 0.16 mmol; Yield: 39.10%; *R*_f_: 0.43 (DCM: MeOH; 99:1); ^1^H NMR (400 MHz, DMSO-d6): δ 10.18 (s, 1H, −NH), 8.51 (s, 1H, coumarin ring-H), 8.45 (s, 1H, coumarin ring-H), 8.30 (t, 1H, −NH–CH_2_–, *J* = 5.8 Hz), 7.93 (d, 2H, Ar–H, *J* = 8.5 Hz), 7.86 (d, 1H, Ar–H, *J* = 8.2 Hz), 7.60 (d, 1H, coumarin ring-H, *J* = 9.00 Hz), 7.52 (t, 1H, Ar–H, *J* = 8.0 Hz), 7.48 (d, 1H, coumarin ring-H, *J* = 9.0 Hz), 7.34 (t, 1H, Ar–H, *J* = 7.9 Hz), 7.12 (d, 2H, Ar–H, *J* = 8.1 Hz), 6.98 (d, 2H, Ar–H, *J* = 8.0 Hz), 6.72 (dd, 2H, coumarin ring-H, *J* = 2.1, 9.1 Hz), 6.52–6.51 (m, 1H, coumain ring-H), 6.45 (s, 1H, coumarin ring-H), 5.91 (s, 2H, −CH_2_−), 4.80 (s, 2H, −CH_2_−), 4.38–4.33 (m, 6H, 3x-CH_2_−), 4.29–4.28 (m, 2H, −CH_2_−), 4.15 (d, 2H, −CH_2_–NH–, *J* = 5.7 Hz), 3.49–3.41 (m, 10H, 5x-CH_2_−), 3.01 (t, 2H, −CH_2_–CH_2_–, *J* = 6.2 Hz), 2.71 (t, 2H, −CH_2_–CH_2_–, *J* = 6.4 Hz), 2.54 (t, 2H, −CH_2_–CH_2_–, *J* = 6.7 Hz), 2.40 (t, 2H, −CH_2_–CH_2_–, *J* = 6.4 Hz), 1.12 (t, 12H, 4x-CH_2_CH_3_, *J* = 6.7 Hz), 0.96 (t, 3H, −O–CH_2_CH_3_, *J* = 6.9 Hz); ^13^C NMR (100 MHz, DMSO-d6): δ 172.8 (C=O), 171.1 (C=O), 163.4 (C=O), 163.2 (C=O), 158.6 (C=O), 158.4 (C=O), 157.4, 153.4, 153.2, 151.3, 149.9, 149.7, 139.2, 135.2, 132.3, 132.2, 129.9, 127.9, 127.8, 126.0, 116.6, 110.2, 109.9, 107.4, 107.3, 107.2, 107.1, 96.3, 96.2 (coumarin+Ar–C), 66.1, 62.7, 62.4, 62.3 (4x-O–CH_2_−), 44.8 (−CH_2_−), 44.7 [(−N(CH_2_)_2_)], 42.0 (−CH_2_–NH−), 30.2, 29.5, 29.4, 28.8 (4x-CH_2_−), 15.2 (−CH_3_ ether), 12.8 (−CH_3_); ESI/MS (*m*/*z*) for C_61_H_65_N_7_O_15_: [M]^−1^_theoretical_: *m*/*z* 1134.5, [M]^−1^_found_: *m*/*z* 1134.5.

#### (**A9**)

4-(2-(2-(2-(7-(Diethylamino)-2-oxo-2*H*-chromene-3-carbonyloxy)ethoxy)ethoxy)ethoxy)-4-oxobutanoic acid: 78.00 mg, 0.16 mmol; Yield: 35.50%; *R*_f_: 0.39 (DCM: MeOH; 99:1); ^1^H NMR (400 MHz, DMSO-d6): δ 10.13 (s, 1H, −NH), 8.51 (s, 2H, coumarin ring-H), 8.29 (t, 1H, −NH–CH_2_–, *J* = 5.8 Hz), 7.98 (d, 2H, Ar–H, *J* = 8.2 Hz), 7.90 (d, 1H, Ar–H, *J* = 8.2 Hz), 7.60 (d, 2H, coumarin ring-H, *J* = 9.0 Hz), 7.55 (t, 1H, Ar–H, *J* = 7.8 Hz), 7.36 (t, 1H, Ar–H, *J* = 7.6 Hz), 7.16 (d, 2H, Ar–H, *J* = 8.1 Hz), 7.00 (d, 2H, Ar–H, *J* = 8.1 Hz), 6.76–6.72 (m, 2H, coumarin ring-H), 6.51 (s, 2H, coumain ring-H), 5.96 (s, 2H, −CH_2_−), 4.82 (s, 2H, −CH_2_−), 4.28 (m, 4H, 2x-CH_2_−), 4.18 (d, 2H, −CH_2_–NH–, *J* = 5.8 Hz), 4.13 (t, 2H, −CH_2_–CH_2_–, *J* = 4.7 Hz), 4.06 (t, 2H, −CH_2_–CH_2_–, *J* = 4.7 Hz), 3.70–3.42 (m, 26H, 13x-CH_2_−), 3.03 (t, 2H, −CH_2_–CH_2_–, *J* = 6.6 Hz), 2.65 (t, 2H, −CH_2_–CH_2_–, *J* = 6.6 Hz), 2.49 (m, 2H, −CH_2_–CH_2_−), 2.36 (t, 1H, −CH_2_–CH_2_–, *J* = 6.8 Hz), 1.12 (dt, 12H, −CH_2_CH_3_, *J* = 6.9, 2.3 Hz), 0.97 (t, 3H, −O–CH_2_CH_3_, *J* = 6.9 Hz); ^13^C NMR (100 MHz, DMSO-d6): δ 172.9 (C=O), 172.8 (C=O), 171.3 (C=O), 171.1 (C=O), 163.7 (C=O), 163.6 (C=O), 158.5 (C=O), 157.4, 153.3, 151.3, 149.7, 144.9, 143.6, 139.2, 135.4, 135.2, 132.2, 129.2, 127.9, 127.8, 126.0, 125.4, 121.3, 116.6, 110.3, 107.6, 107.4, 96.3 (coumarin+Ar–C), 70.3, 70.2, 68.8, 68.7, 68.7, 66.1, 64.6, 64.1, 63.8, 63.7 (10x-O–CH_2_−), 49.1 (−CH_2_−), 44.8 [(-N(CH_2_)_2_)], 42.1 (−CH_2_–NH−), 31.7, 30.2, 29.3, 29.0 (4x-CH_2_−), 15.2 (−CH_3_ ether), 12.8 (−CH_3_); ESI/MS (*m*/*z*) for C_69_H_81_N_7_O_19_: [M]^−1^_theoretical_: *m*/*z* 1310.4, [M]^−1^_found_: *m*/*z* 1310.4.

#### (**A10**)

4-(2-(2-(2-(2-(7-(Diethylamino)-2-oxo-2*H*-chromene-3-carbonyloxy)ethoxy)ethoxy)ethoxy)ethoxy)-4-oxobutanoic acid: 86.00 mg, 0.16 mmol; Yield: 50.00%; *R*_f_: 0.45 (DCM: MeOH; 99:1); ^1^H NMR (400 MHz, DMSO-d6): δ 10.15 (s, 1H, −NH), 8.51 (s, 2H, coumarin ring-H), 8.30 (t, 1H, −NH–CH_2_–, *J* = 5.6 Hz), 7.99 (d, 2H, Ar–H, *J* = 8.4 Hz), 7.90 (d, 1H, Ar–H, *J* = 8.2 Hz), 7.60 (d, 2H, coumarin ring-H, *J* = 9.00 Hz), 7.58 (t, 1H, Ar–H, *J* = 7.9 Hz), 7.37 (t, 1H, Ar–H, *J* = 7.3 Hz), 7.16 (d, 2H, Ar–H, *J* = 8.2 Hz), 7.01 (d, 2H, Ar–H, *J* = 8.1 Hz), 6.76–6.73 (m, 2H, coumarin ring-H), 6.52 (s, 2H, coumain ring-H), 5.96 (s, 2H, −CH_2_−), 4.82 (s, 2H, −CH_2_−), 4.29 (t, 4H, 2x-CH_2_–, *J* = 4.6 Hz), 4.17 (d, 2H, −CH_2_–NH–, *J* = 5.6 Hz), 4.12 (t, 2H, −CH_2_–CH_2_–, *J* = 4.7 Hz), 4.05 (t, 2H, −CH_2_–CH_2_–, *J* = 4.6 Hz), 3.71–3.43 (m, 30H, 15x-CH_2_−), 3.03 (t, 2H, −CH_2_–CH_2_–, *J* = 6.7 Hz), 2.66 (t, 2H, −CH_2_–CH_2_–, *J* = 6.6 Hz), 2.50–2.48 (m, 2H, −CH_2_–CH_2_−), 2.37 (t, 2H, −CH_2_–CH_2_–, *J* = 6.7 Hz), 1.12 (t, 12H, 4x-CH_2_CH_3_, *J* = 6.9 Hz), 0.97 (t, 3H, −O–CH_2_CH_3_, *J* = 6.9 Hz); ^13^C NMR (100 MHz, DMSO-d6): δ 172.8 (C=O), 172.8 (C=O), 171.3 (C=O), 171.1 (C=O), 163.7 (C=O), 158.5 (C=O), 157.4, 153.3, 151.3, 149.7, 144.9, 143.6, 139.3, 135.5, 135.2, 132.2, 129.2, 127.9, 127.8, 126.0, 125.4, 121.3, 116.6, 110.3, 107.6, 107.4, 96.3 (coumarin+Ar–C), 70.3, 70.2, 70.2, 68.8, 68.7, 68.7, 66.2, 66.1, 64.6, 64.1, 63.8, 63.7 (12x-O–CH_2_−), 49.1 (−CH_2_−), 44.8 [(-N(CH_2_)_2_)], 42.1 (−CH_2_–NH−), 31.7, 30.2, 29.4, 29.3 (4x-CH_2_−), 15.2 (−CH_3_ ether), 12.8 (−CH_3_); ESI/MS (*m*/*z*) for C_73_H_89_N_7_O_21_: [M]^−1^_theoretical_: *m*/*z* 1398.4, [M]^−1^_found_: *m*/*z* 1398.4.

#### (**A11**)

4-(2-(2-(2-(2-(2-(7-(Diethylamino)-2-oxo-2*H*-chromene-3-carbonyloxy)ethoxy)ethoxy)ethoxy)ethoxy) ethoxy)-4-oxobutanoic acid: 93.00 mg, 0.16 mmol; Yield: 30.71%; *R*_f_: 0.37 (DCM: MeOH; 99:1); ^1^H NMR (400 MHz, DMSO-d6): δ 10.15 (s, 1H, −NH), 8.52 (s, 2H, coumarin ring-H), 8.30 (t, 1H, −NH–CH_2_–, *J* = 5.6 Hz), 7.99 (d, 2H, Ar–H, *J* = 8.5 Hz), 7.90 (d, 1H, Ar–H, *J* = 8.3 Hz), 7.61 (d, 2H, coumarin ring-H, *J* = 9.00 Hz), 7.56 (t, 1H, Ar–H, *J* = 7.8 Hz), 7.39–7.36 (m, 1H, Ar–H), 7.16 (d, 2H, Ar–H, *J* = 8.0 Hz), 7.01 (d, 2H, Ar–H, *J* = 7.9 Hz), 6.75 (d, 2H, coumarin ring-H, *J* = 9.0 Hz), 6.52 (s, 2H, coumain ring-H), 5.96 (s, 2H, −CH_2_−), 4.82 (s, 2H, −CH_2_−), 4.29 (t, 4H, 2x-CH_2_–, *J* = 4.6 Hz), 4.17 (d, 2H, −CH_2_–NH–, *J* = 5.6 Hz), 4.13 (t, 2H, −CH_2_–CH_2_–, *J* = 4.6 Hz), 4.05 (t, 2H, −CH_2_–CH_2_–, *J* = 4.6 Hz), 3.69 (t, 4H, 2x-CH_2_–, *J* = 4.5 Hz), 3.60–3.44 (m, 36H, 18x-CH_2_−), 3.04 (t, 2H, −CH_2_–CH_2_–, *J* = 6.4 Hz), 2.66 (t, 2H, −CH_2_–CH_2_–, *J* = 6.6 Hz), 2.37 (t, 2H, −CH_2_–CH_2_–, *J* = 6.8 Hz), 1.12 (t, 12H, 4x-CH_2_CH_3_, *J* = 6.9 Hz), 0.97 (t, 3H, −O–CH_2_CH_3_, *J* = 6.9 Hz); ^13^C NMR (100 MHz, DMSO-d6): δ 172.9 (C=O), 172.8 (C=O), 171.1 (C=O), 163.7 (C=O), 158.5 (C=O), 157.4, 153.3, 151.3, 149.7, 144.9, 143.6, 139.2, 135.4, 135.2, 132.2, 129.2, 127.9, 127.8, 126.0, 121.3, 116.6, 110.3, 107.6, 107.4, 96.3 (coumarin+Ar–C), 70.3, 70.2, 70.1, 68.8, 68.7, 68.6, 66.1, 64.6, 64.1, 63.8, 63.7 (11x-O–CH_2_−), 44.8 [(-N(CH_2_)_2_], 42.1 (−CH_2_NH−), 31.7, 30.2, 29.4, 29.0 (4x-CH_2_−), 15.2 (−CH_3_ ether), 12.8 (−CH_3_); ESI/MS (*m*/*z*) for C_77_H_97_N_7_O_23_: [M]^−1^_theoretical_: *m*/*z* 1486.4, [M]^−1^_found_: *m*/*z* 1486.4.

#### (**A12**)

4-(12-(7-(Diethylamino)-2-oxo-2*H*-chromene-3-carbonyloxy)dodecyloxy)-4-oxobutanoic acid: 87.00 mg, 0.16 mmol; Yield: 31.97%; *R*_f_: 0.51 (DCM: MeOH; 99:1); ^1^H NMR (400 MHz, DMSO-d6): δ 8.51 (s, 2H, coumarin ring-H), 8.29 (t, 1H, −NH–CH_2_–, *J* = 5.6 Hz), 8.00 (d, 2H, Ar–H, *J* = 8.0 Hz), 7.92 (d, 1H, Ar–H, *J* = 8.2 Hz), 7.61 (d, 2H, coumarin ring-H, *J* = 9.00 Hz), 7.57 (t, 1H, Ar–H, *J* = 7.9 Hz), 7.38 (t, 1H, Ar–H, *J* = 7.6 Hz), 7.16 (d, 2H, Ar–H, *J* = 8.2 Hz), 7.01 (d, 2H, Ar–H, *J* = 8.1 Hz), 6.75 (dd, 2H, coumarin ring-H, *J* = 1.6, 8.9 Hz), 6.52 (s, 2H, coumain ring-H), 5.97 (s, 2H, −CH_2_−), 4.82 (s, 2H, −CH_2_−), 4.19–4.14 (m, 6H, 3x-CH_2_−), 4.00 (t, 2H, −CH_2_–CH_2_–, *J* = 6.6 Hz), 3.91 (t, 2H, −CH_2_–CH_2_–, *J* = 6.5 Hz), 3.51–3.44 (m, 10H, 5x-CH_2_−), 3.04 (t, 2H, −CH_2_–CH_2_–, *J* = 6.4 Hz), 2.65 (t, 2H, −CH_2_–CH_2_–, *J* = 6.6 Hz), 2.47 (t, 2H, −CH_2_–CH_2_–, *J* = 6.5 Hz), 2.36 (t, 1H, −CH_2_–CH_2_–, *J* = 6.6 Hz), 1.65–1.18 (m, 40H, 20x-CH_2_−), 1.12 (t, 12H, 4x-CH_2_CH_3_, *J* = 6.9 Hz), 0.97 (t, 3H, −O–CH_2_CH_3_, *J* = 6.9 Hz); ^13^C NMR (100 MHz, DMSO-d6): δ 172.8 (C=O), 172.7 (C=O), 171.1 (C=O), 163.9 (C=O), 158.5 (C=O), 153.3, 149.6, 139.3, 135.1, 132.2, 129.9, 127.9, 126.0, 125.5, 121.3, 110.2, 107.9, 107.5, 96.3 (coumarin+Ar–C), 66.0, 64.7, 64.6, 64.3, 64.2 (5x-O–CH_2_−), 49.1 (−CH_2_−), 44.8 (-N(CH_2_)_2_, 42.0 (−CH_2_–NH−), 31.7, 30.4, 30.3, 29.5, 29.4, 29.1, 28.8, 28.6, 28.5, 25.8, 25.7, 23.9, (12x-CH_2_−), 15.2 (−CH_3_ ether), 12.8 (−CH_3_).

### General Method for the Synthesis of **S1**–**13**

The synthesis of compounds **S1**-**13** was accomplished following reported methods with slight modifications.^50,51^ In a flame-dried 100 mL round-bottom flask, compound **I** (50 mg, 0.138 mmol) was dissolved in 0.8 mL of dry triethylamine and 5 mL of dry DCM. The reaction mixture was cooled to 0 °C on an ice bath and placed under argon protection. After continuous stirring for 10 min, a catalytic amount of DMAP (4.6 mg, 0.038 mmol) dissolved in 1 mL dry DCM was added to the reaction mixture. Respective sulfonyl chloride (1.1 equiv) was then added dropwise to the reaction mixture and stirred further for 40 min before warming to room temperature and stirring for up to an additional 48 h until complete by TLC (duration noted for each compound below). The solvent was removed from the crude reaction mixture by rotavap, and the residue was dissolved in DCM before gradient flash column chromatography 0 to 10% MeOH in DCM. Following rotavap removal of solvent, final products were obtained by lyophilization from benzene before characterization.

#### (**S1**)

Methanesulfonyl chloride: 22.0 mg, 0.152 mmol; Duration: 21 h; Yield: 65.27%; *R*_f_: 0.24 (DCM: MeOH; 94:6); ^1^H NMR (400 MHz, DMSO-*d*_6_): δ 7.75 (d, 1H, Ar–H, *J* = 8.4 Hz, 7.57 (d, 1H, Ar–H, *J* = 8.4 Hz), 7.50 (t, 1H, Ar–H, *J* = 6.2 Hz), 7.36 (t, 1H, Ar–H, *J* = 7.6 Hz), 7.28 (d, 2H, Ar–H, *J* = 7.8 Hz), 7.05 (d, 2H, Ar–H, *J* = 7.9 Hz), 6.77 (s, 2H, −NH_2_), 5.91 (s, 2H, −CH_2_−), 4.78 (s, 2H, −CH_2_−), 4.09 (d, 2H, −CH_2_–NH–, *J* = 6.4 Hz), 3.49 (q, 2H, -OCH_2_CH_3_, *J* = 6.8 Hz), 2.76 (s, 3H, −CH_3_), 0.97 (t, 3H, −CH_3_ ether, *J* = 6.8 Hz); ^13^C NMR (100 MHz, DMSO-*d*_6_): δ 152.3, 150.0, 137.8, 136.0, 134.1, 128.5, 127.4, 126.7, 126.1, 121.5, 121.1, 114.7 (Ar–C), 65.8, 64.6 (2x-OCH_2_– ether), 48.9 (−CH_2_−), 46.1 (−CH_2_–NH−), 40.2 (−CH_3_), 15.2 (−CH_3_ ether); ESI/MS (*m*/*z*) for C_22_H_25_N_5_O_3_S: [M + H]^+^_theoretical_: *m*/*z* 440.2, [M + H]^+^_found_: *m*/*z* 440.2.

#### (**S2**)

Ethanesulfonyl chloride: 19.57 mg, 0.152 mmol; Duration: 36 h; Yield: 24.20%; *R*_f_: 0.28 (DCM: MeOH; 94:6); ^1^H NMR (400 MHz, DMSO-*d*_6_): δ 7.76 (d, 1H, Ar–H, *J* = 8.1 Hz), 7.59 (d, 1H, Ar–H, *J* = 8.2 Hz), 7.54 (t, 1H, −NH–CH_2_–, *J* = 6.2 Hz), 7.37 (t, 1H, Ar–H, *J* = 7.6 Hz), 7.28 (d, 2H, Ar–H, *J* = 8.0 Hz), 7.07–7.04 (m, 3H, Ar–H), 6.90 (s, 2H, −NH_2_), 5.91 (s, 2H, −CH_2_−), 4.79 (s, 2H, −CH_2_−), 4.07 (d, 2H, −CH_2_–NH–, *J* = 6.2 Hz), 3.49 (q, 2H, -OCH_2_CH_3_, *J* = 6.9 Hz), 2.80 (q, 2H, −CH_2_–CH_3_, *J* = 7.3 Hz), 1.05 (t, 2H, −CH_2_–CH_3_, *J* = 7.3 Hz), 0.98 (t, 3H, −CH_3_ ether, *J* = 6.9 Hz); ^13^C NMR (100 MHz, DMSO-*d*_6_): δ 152.2, 150.2, 138.1, 135.9, 134.3, 128.5, 127.6, 126.7, 126.1, 125.8, 121.7, 121.2, 114.6 (Ar–C), 65.9, 64.6 (2x-OCH_2_– ether), 48.9 (−CH_2_−), 46.4 (−CH_2_–NH−), 45.9 (−CH_2_−), 15.2 (−CH_3_ ether), 8.4 (−CH_3_); ESI/MS (*m*/*z*) for C_23_H_27_N_5_O_3_S: [M + H]^+^_theoretical_= *m*/*z* 454.2, [M + H]^+^_observed_= *m*/*z* 454.2.

#### (**S3**)

*n*-Propanesulfonyl chloride: 22.05 mg, 0.155 mmol; Duration: 24 h; Yield: 50.83%; *R*_f_: 0.30 (DCM: MeOH; 94:6); ^1^H NMR (400 MHz, DMSO-*d*_6_): δ 7.82 (d, 1H, Ar–H, *J* = 8.2 Hz, 7.64 (d, 1H, Ar–H, *J* = 8.3 Hz), 7.57 (m, 3H, −NH–CH_2_–/–NH_2_), 7.45 (t, 1H, Ar–H, *J* = 7.6 Hz), 7.29 (d, 2H, Ar–H, *J* = 8.0 Hz), 7.14 (t, 1H, Ar–H, *J* = 7.6 Hz), 7.07 (d, 2H, Ar–H, *J* = 8.0 Hz), 5.94 (s, 2H, −CH_2_−), 4.80 (s, 2H, −CH_2_−), 4.08 (d, 2H, −CH_2_–NH–, *J* = 6.0 Hz), 3.50 (q, 2H, -OCH_2_CH_3_, *J* = 6.9 Hz), 2.79 (t, 2H, −CH_2_CH_2_–, *J* = 7.7 Hz), 1.54 (sextet, 2H, −CH_2_CH_2_CH_3_, *J* = 7.7 Hz), 0.98 (t, 3H, −CH_3_ ether, *J* = 6.9 Hz), 0.82 (t, 3H, −CH_2_CH_3_, *J* = 7.4 Hz; ^13^C NMR (100 MHz, DMSO-*d*_6_): δ 151.6, 150.9, 138.2, 135.7, 134.8, 128.4, 128.3, 126.3, 126.2, 124.0, 122.6, 121.4, 114.2 (Ar–C), 65.9, 64.5 (2x-OCH_2_– ether), 53.7, 17.2, 13.0 (*n*-propyl carbons), 49.0 (−CH_2_−), 45.9 (−CH_2_–NH−), 15.2 (−CH_3_ ether); ESI/MS (*m*/*z*) for C_24_H_29_N_5_O_3_S: [M + H]^+^_theoretical_: *m*/*z* 468.2, [M + H]^+^_found_: *m*/*z* 468.2.

#### (**S4**)

Cyclopropanesulfonyl chloride: 21.40 mg, 0.152 mmol; Duration: 24 h; Yield: 49.10%; *R*_f_: 0.27 (DCM: MeOH; 94:6); ^1^H NMR (400 MHz, DMSO-*d*_6_): δ 7.82 (d, 1H, Ar–H, *J* = 8.2 Hz), 7.67 (d, 1H, Ar–H, *J* = 8.3 Hz), 7.62 (t, 1H, −NH–CH_2_–, *J* = 6.4 Hz), 7.48 (t, 1H, Ar–H, *J* = 7.6 Hz), 7.30 (d, 2H, Ar–H, *J* = 8.0 Hz), 7.16 (t, 1H, Ar–H, *J* = 7.6 Hz), 7.06 (d, 2H, Ar–H, *J* = 8.0 Hz), 5.95 (s, 2H, −CH_2_−), 4.82 (s, 2H, −CH_2_−), 4.13 (d, 2H, −CH_2_–NH–, *J* = 6.2 Hz), 3.50 (q, 2H, -OCH_2_CH_3_, *J* = 7.1 Hz), 2.32–2.26 (m, 1H, cyclopropyl-H), 0.97 (t, 3H, −CH_3_ ether, *J* = 7.0 Hz), 0.83–0.79 (m, 2H, cyclopropyl-H), 0.76–0.70 (m, 2H, cyclopropyl-H); ^13^C NMR (100 MHz, DMSO-*d*_6_): δ 151.4, 138.5, 135.5, 135.1, 128.7, 128.4, 126.2, 123.0, 121.8, 113.9 (Ar–C), 65.9, 64.5 (2x-OCH_2_– ether), 30.3, 29.5, 5.2 (cyclopropyl carbons), 49.0 (−CH_2_−), 46.1 (−CH_2_–NH−), 15.2 (−CH_3_ ether); ESI/MS (*m*/*z*) for C_24_H_27_N_5_O_3_S: [M + H]^+^_theoretical_: *m*/*z* 466.2, [M + H]^+^_found_: *m*/*z* 466.2.

#### (**S5**)

Pyrrolidinesulfonyl chloride: 28.19 mg, 0.17 mmol; Duration: 24 h; Yield: 1.61%; *R*_f_: 0.28 (DCM: MeOH; 93:7); ^1^H NMR (400 MHz, DMSO-*d*_6_): δ 7.86 (d, 1H, Ar–H, *J* = 8.4 Hz), 7.71 (d, 1H, Ar–H, *J* = 8.0 Hz), 7.58–7.53 (m, 2H, −NH–CH_2_– and Ar–H), 7.28 (t, 1H, Ar–H, *J* = 7.6 Hz), 7.22 (d, 2H, Ar–H, *J* = 8.0 Hz), 7.01 (d, 2H, Ar–H, *J* = 8.0 Hz), 5.92 (s, 2H, −CH_2_−), 4.76 (s, 2H, −CH_2_−), 3.99 (d, 2H, −CH_2_–NH–, *J* = 6.0 Hz), 3.44 (q, 2H, -OCH_2_CH_3_, *J* = 7.0 Hz), 2.94 (t, 4H, pyrrolidine-H, *J* = 6.6 Hz), 1.53 (t, 4H, pyrrolidine-H, *J* = 6.6 Hz), 0.91 (t, 3H, −CH_3_ ether, *J* = 7.0 Hz); ^13^C NMR (100 MHz, DMSO-*d*_6_): δ 153.1, 149.8, 138.8, 136.4, 134.7, 134.5, 130.3, 128.5, 126.1, 125.2, 125.1, 122.5, 118.9, 112.8 (Ar–C), 66.2, 64.3 (2x-OCH_2_– ether), 49.3 (−CH_2_−), 47.8 (pyrrolidine-C), 46.3 (−CH_2_–NH−), 25.3 (pyrrolidine-C), 15.1 (−CH_3_ ether); ESI/MS (*m*/*z*) for C_25_H_30_N_6_O_3_S: [M + H]^+^_theoretical_= *m*/*z* 495.2, [M + H]^+^_observed_= *m*/*z* 495.2.

#### (**S6**)

Thiophene-2-sulfonyl chloride: 28.60 mg, 0.157 mmol; Duration: 24 h; Yield: 29.91%; *R*_f_: 0.32 (DCM: MeOH; 94:6); ^1^H NMR (400 MHz, DMSO-*d*_6_): δ 8.31 (t, 1H, −NH–CH_2_–, *J* = 5.4 Hz), 7.82 (d, 1H, thiophene-H, *J* = 4.9 Hz), 7.77 (d, 1H, Ar–H, *J* = 8.1 Hz), 7.58 (d, 1H, Ar–H, *J* = 8.2 Hz), 7.53 (d, 1H, thiophene-H, *J* = 2.6 Hz), 7.36 (t, 1H, Ar–H, *J* = 7.6 Hz), 7.19 (d, 2H, Ar–H, *J* = 8.0 Hz), 7.09–7.03 (m, 2H, Ar–H and thiophene-H), 7.00 (d, 2H, Ar–H, *J* = 8.0 Hz), 6.67 (s, 2H, −NH_2_), 5.89 (s, 2H, −CH_2_−), 4.77 (s, 2H, −CH_2_−), 4.01 (d, 2H, −CH_2_–NH–, *J* = 5.2 Hz), 3.50 (q, 2H, -OCH_2_CH_3_, *J* = 6.9 Hz), 0.99 (t, 3H, −CH_3_ ether, *J* = 6.9 Hz); ^13^C NMR (100 MHz, DMSO-*d*_6_): δ 152.5, 149.9, 137.0, 136.0, 134.0, 128.4, 128.0, 127.3, 126.8, 126.5, 126.0, 121.4, 121.0, 114.8 (Ar–C), 145.7, 142.0, 132.7, 131.9 (thiophene-C), 65.8, 64.6 (2x-OCH_2_– ether), 48.8 (−CH_2_−), 46.3 (−CH_2_–NH−), 15.2 (−CH_3_ ether); ESI/MS (*m*/*z*) for C_25_H_25_N_5_O_3_S_2_: [M + H]^+^_theoretical_: *m*/*z* 508.1, [M + H]^+^_found_: *m*/*z* 508.2.

#### (**S7**)

Piperidine-1-sulfonyl chloride: 27.85 mg, 0.152 mmol; Duration: 22 h; Yield: 23.26%; *R*_f_: 0.29 (DCM: MeOH; 92:8); ^1^H NMR (400 MHz, DMSO-*d*_6_): δ 7.72 (d, 1H, Ar–H, *J* = 8.2 Hz), 7.57 (t, 1H, −NH–CH_2_–, *J* = 6.2 Hz), 7.52 (d, 1H, Ar–H, *J* = 8.3 Hz), 7.31 (t, 1H, Ar–H, *J* = 7.6 Hz), 7.19 (d, 2H, Ar–H, *J* = 8.0 Hz), 7.07–7.05 (m, 3H, Ar–H), 6.87 (s, 2H, −NH_2_), 5.84 (s, 2H, −CH_2_−), 4.71 (s, 2H, −CH_2_−), 3.94 (d, 2H, −CH_2_–NH–, *J* = 6.1 Hz), 3.42 (q, 2H, -OCH_2_CH_3_, *J* = 6.9 Hz), 2.83–2.81 (m, 4H, piperidine-H), 1.29–1.25 (m, 6H, piperidine-H), 0.91 (t, 3H, −CH_3_ ether, *J* = 6.9 Hz); ^13^C NMR (100 MHz, DMSO-*d*_6_): δ 152.2, 150.2, 138.3, 135.8, 134.3, 128.5, 127.6, 126.6, 126.0, 125.7, 121.8, 121.2, 114.6 (Ar–C), 65.9, 64.6 (2x-OCH_2_– ether), 48.9 (−CH_2_−), 46.3 (−CH_2_–NH−), 46.6, 25.2, 23.6 (piperidine-C), 15.2 (−CH_3_ ether); ESI/MS (*m*/*z*) for C_26_H_32_N_6_O_3_S: [M + H]^+^_theoretical_= *m*/*z* 509.2, [M + H]^+^_observed_= *m*/*z* 509.2.

#### (**S8**)

Benzenesulfonyl chloride: 27.00 mg, 0.153 mmol; Duration: 48 h; Yield: 46.08%; *R*_f_: 0.54 (DCM: MeOH; 94:6); ^1^H NMR (400 MHz, DMSO-*d*_6_): δ 8.13 (t, 1H, −NH–CH_2_–, *J* = 6.2 Hz), 7.81 (d, 1H, Ar–H, *J* = 8.2 Hz), 7.75 (d, 2H, Ar–H, *J* = 7.2 Hz), 7.65 (d, 1H, Ar–H, *J* = 8.2 Hz), 7.59–7.45 (m, 5H, Ar–H), 7.18–7.14 (m, 3H, Ar–H), 7.00 (d, 2H, Ar–H, *J* = 8.0 Hz), 5.91 (s, 2H, −CH_2_−), 4.78 (s, 2H, −CH_2_−), 3.92 (d, 2H, −CH_2_–NH–, *J* = 6.1 Hz), 3.49 (q, 2H, -OCH_2_CH_3_, *J* = 6.9 Hz), 0.98 (t, 3H, −CH_3_ ether, *J* = 6.9 Hz); ^13^C NMR (100 MHz, DMSO-*d*_6_): δ 151.5, 150.9, 141.1, 137.4, 135.6, 134.7, 132.7, 129.6, 128.4, 126.9, 126.3, 126.0, 124.1, 122.6, 121.5, 114.2 (Ar–C), 65.9, 64.5 (2x-OCH_2_– ether), 48.9 (−CH_2_−), 46.1 (−CH_2_–NH−), 15.2 (−CH_3_ ether); ESI/MS (*m*/*z*) for C_27_H_27_N_5_O_3_S: [M + H]^+^_theoretical_: *m*/*z* 502.2, [M + H]^+^_found_: *m*/*z* 502.2.

#### (**S9**)

4-Methylbenzenesulfonyl chloride: 29.00 mg, 0.152 mmol; Duration: 24 h; Yield: 43.19%; *R*_f_: 0.29 (DCM: MeOH; 94:6); ^1^H NMR (500 MHz, DMSO-*d*_6_): δ 8.01 (s, 1H, −NH−), 7.77 (d, 1H, Ar–H, *J* = 7.8 Hz), 7.64 (d, 2H, Ar–H, *J* = 7.9 Hz), 7.59 (d, 1H, Ar–H, *J* = 8.0 Hz), 7.37 (t, 1H, Ar–H, *J* = 7.0 Hz), 7.31 (d, 2H, Ar–H, *J* = 7.6 Hz), 7.17 (d, 2H, Ar–H, *J* = 7.6 Hz), 7.05 (t, 1H, Ar–H, *J* = 7.0 Hz), 6.99 (d, 2H, Ar–H, *J* = 7.6 Hz), 6.71 (s, 2H, −NH_2_), 5.89 (s, 2H, −CH_2_−), 4.77 (s, 2H, −CH_2_−), 3.89 (d, 2H, −CH_2_–NH–, *J* = 4.4 Hz), 3.49 (q, 2H, -OCH_2_CH_3_, *J* = 6.8 Hz), 0.99 (t, 3H, −CH_3_ ether, *J* = 6.8 Hz); ^13^C NMR (126 MHz, DMSO-*d*_6_): δ 152.5, 149.8, 145.7, 143.0, 138.2, 137.2, 135.9, 133.9, 130.0, 128.8, 127.3, 126.9, 126.8, 126.6, 126.0, 121.4, 121.0, 114.8 (Ar–C), 65.8, 64.6 (2x-OCH_2_– ether), 48.8 (−CH_2_−), 46.1 (−CH_2_–NH−), 21.36 (−CH_3_), 15.2 (−CH_3_ ether); ESI/MS (*m*/*z*) for C_28_H_29_N_5_O_3_S: [M + H]^+^_theoretical_: *m*/*z* 516.2, [M + H]^+^_found_: *m*/*z* 516.2.

#### (**S10**)

4-Methoxybenzenesulfonyl chloride: 32.10 mg, 0.155 mmol; Duration: 24 h; Yield: 46.13%; *R*_f_: 0.28 (DCM: MeOH; 94:6); ^1^H NMR (400 MHz, DMSO-*d*_6_): δ 7.95 (t, 1H, −NH–CH_2_–, *J* = 6.2 Hz), 7.81 (d, 1H, Ar–H, *J* = 8.2 Hz), 7.70 (d, 2H, Ar–H, *J* = 8.8 Hz), 7.64 (d, 1H, Ar–H, *J* = 8.2 Hz), 7.44 (t, 1H, Ar–H, *J* = 7.6 Hz), 7.34 (s, 2H, −NH_2_), 7.19 (d, 2H, Ar–H, *J* = 8.0 Hz), 7.13 (t, 1H, Ar–H, *J* = 7.6 Hz), 7.06 (d, 2H, Ar–H, *J* = 8.8 Hz), 7.01 (d, 2H, Ar–H, *J* = 8.0 Hz), 5.91 (s, 2H, −CH_2_−), 4.79 (s, 2H, −CH_2_−), 3.88 (d, 2H, −CH_2_–NH–, *J* = 6.1 Hz), 3.82 (s, 3H, -OCH_3_), 3.50 (q, 2H, -OCH_2_CH_3_, *J* = 6.9 Hz), 0.99 (t, 3H, −CH_3_ ether, *J* = 6.9 Hz); ^13^C NMR (100 MHz, DMSO-*d*_6_): δ 162.5, 151.8, 150.7, 142.9, 137.5, 135.7, 134.6, 132.7, 129.1, 128.4, 128.1, 126.4, 126.0, 124.6, 122.4, 121.4, 114.7, 114.3 (Ar–C), 65.9, 64.5 (2x-OCH_2_– ether), 56.1 (−OCH_3_), 48.9 (−CH_2_−), 46.1 (−CH_2_–NH−), 15.2 (−CH_3_ ether); ESI/MS (*m*/*z*) for C_28_H_29_N_5_O_4_S: [M + H]^+^_theoretical_: *m*/*z* 532.2, [M + H]^+^_found_: *m*/*z* 532.2.

#### (**S11**)

4-Chlorobenzenesulfonyl chloride: 32.08 mg, 0.152 mmol; Duration: 20 h; Yield: 33.28%; *R*_f_: 0.53 (DCM: MeOH; 94:6); ^1^H NMR (400 MHz, DMSO-*d*_6_): δ 8.23 (t, 1H, −NH–CH_2_–, *J* = 6.2 Hz), 7.76–7.72 (m, 3H, Ar–H), 7.59–7.57 (m, 3H, Ar–H), 7.36 (t, 1H, Ar–H, *J* = 7.6 Hz), 7.16 (d, 2H, Ar–H, *J* = 8.1 Hz), 7.05 (t, 1H, Ar–H, *J* = 7.6 Hz), 6.98 (d, 2H, Ar–H, *J* = 8.1 Hz), 6.75 (s, 2H, −NH_2_), 5.88 (s, 2H, −CH_2_−), 4.76 (s, 2H, −CH_2_−), 3.94 (d, 2H, −CH_2_–NH–, *J* = 6.0 Hz), 3.49 (q, 2H, -OCH_2_CH_3_, *J* = 6.9 Hz), 0.98 (t, 3H, −CH_3_ ether, *J* = 6.9 Hz); ^13^C NMR (100 MHz, DMSO-*d*_6_): δ 152.4, 149.9, 140.0, 137.6, 137.0, 136.0, 134.0, 129.7, 128.9, 128.4, 127.4, 126.8, 126.4, 126.0, 121.3, 121.1, 114.8 (Ar–C), 65.9, 64.6 (2x-OCH_2_– ether), 48.8 (−CH_2_−), 46.2 (−CH_2_–NH−), 15.2 (−CH_3_ ether); ESI/MS (*m*/*z*) for C_27_H_26_ClN_5_O_3_S: [M + H]^+^_theoretical_: *m*/*z* 536.2, [M + H]^+^_found_: *m*/*z* 536.2.

#### (**S12**)

4-Nitrobenzenesulfonyl chloride: 33.74 mg, 0.152 mmol; Duration: 24 h; Yield: 40.00%; *R*_f_: 0.45 (DCM: MeOH; 94:6); ^1^H NMR (400 MHz, DMSO-*d*_6_): δ 8.51 (s, −NH), 8.35 (d, 2H, Ar–H, *J* = 8.8 Hz), 7.99 (d, 2H, Ar–H, *J* = 8.8 Hz), 7.73 (d, 1H, Ar–H, *J* = 8.3 Hz), 7.57 (d, 1H, Ar–H, *J* = 8.3 Hz), 7.35 (t, 1H, Ar–H, *J* = 7.6 Hz), 7.16 (d, 2H, Ar–H, *J* = 8.0 Hz), 7.03 (t, 1H, Ar–H, *J* = 7.6 Hz), 6.98 (d, 2H, Ar–H, *J* = 8.0 Hz), 6.81 (s, 2H, −NH_2_), 5.87 (s, 2H, −CH_2_−), 4.75 (s, 2H, −CH_2_−), 3.99 (d, 2H, −CH_2_–NH–, *J* = 4.4 Hz), 3.48 (q, 2H, -OCH_2_CH_3_, *J* = 7.2 Hz), 0.97 (t, 3H, −CH_3_ ether, *J* = 7.2 Hz); ^13^C NMR (100 MHz, DMSO-*d*_6_): δ 152.6, 151.1, 149.9, 145.8, 144.2, 137.5, 140.0, 133.9, 129.9, 129.6, 127.3, 126.9, 126.7, 126.4, 125.1, 121.3, 120.9, 114.8 (Ar–C), 65.8, 64.6 (2x-OCH_2_– ether), 52.8 (−CH_2_−), 48.8 (−CH_2_–NH−), 15.2 (−CH_3_ ether); ESI/MS (*m*/*z*) for C_27_H_26_N_6_O_5_S: [M + H]^+^_theoretical_: *m*/*z* 547.2, [M + H]^+^_found_: *m*/*z* 547.3, [M + Na]^+^_theoretical_: 569.3, [M + Na]^+^_found_: 569.3.

#### (**S-13**)

Coumarin-6-sulfonyl chloride: 37.23 mg, 0.152 mmol; Duration: 20.5 h; Yield: 58.48%; *R*_f_: 0.52 (DCM: MeOH; 94:6); ^1^H NMR (400 MHz, DMSO-*d*_6_): δ 8.25 (t, 1H, −NH–CH_2_–, *J* = 6.2 Hz), 8.19–8.15 (m, 2H, Ar–H+coumarin-H), 7.92 (dd, 1H, coumarin-H, *J* = 8.7, 1.91 Hz), 7.82 (d, 1H, Ar–H, *J* = 8.2 Hz), 7.67 (d, 1H, coumarin-H, *J* = 8.3 Hz), 7.53 (d, 1H, coumarin-H, *J* = 8.7 Hz), 7.49 (t, 1H, Ar–H, *J* = 7.7 Hz), 7.20–7.17 (m, 3H, Ar–H), 7.01 (d, 2H, Ar–H, *J* = 8.0 Hz), 6.62 (d, 1H, coumarin-H, *J* = 9.6 Hz), 5.90 (s, 2H, −CH_2_−), 4.78 (s, 2H, −CH_2_−), 3.97 (d, 2H, −CH_2_–NH–, *J* = 6.0 Hz), 3.49 (q, 2H, -OCH_2_CH_3_, *J* = 6.9 Hz), 0.97 (t, 3H, −CH_3_ ether, *J* = 6.9 Hz); ^13^C NMR (100 MHz, DMSO-*d*_6_): δ 159.7, 158.9, 155.9, 151.4, 151.1, 144.1, 137.8, 137.1, 135.5, 135.1, 130.0, 128.8, 128.5, 127.9, 126.1, 126.0, 123.2, 121.7, 119.3, 118.1, 117.9, 113.9 (Ar–C), 65.9, 64.4 (2x-OCH_2_– ether), 48.9 (−CH_2_−), 46.1 (−CH_2_–NH−), 15.2 (−CH_3_ ether); ESI/MS (*m*/*z*) for C_30_H_27_N_5_O_5_S: [M + Na]^+^_theoretical_: *m*/*z* 570.2, [M + H]^+^_found_: *m*/*z* 570.2.

### Purity Statement

Active lead compounds (**S3** and **S5**) were quantified by HPLC and found to be >95% pure. All compounds were also assessed for purity by ^1^H NMR. HPLC traces and NMR spectra are available in the Supporting Information.

### Cell Lines and Culture Media

#### RAW-Blue Cells

RAW-Blue macrophages expressing a secreted embryonic alkaline phosphatase (SEAP) gene induced by transcriptional activation of NF-*k*B and activator protein 1 (AP-1) upon pattern recognition receptor (PRR) stimulation were purchased from InvivoGen and grown at 37 °C in a 5% CO_2_ incubator according to manufacturer instructions. Briefly, cells were grown to 80% confluence in T-75 culture flasks in high-glucose Dulbecco’s Modified Eagle Medium (DMEM, 4.5 g of glucose/L) supplemented with 2 mM l-glutamine, 10% (v/v) FBS, 100 μg/mL Normocin, and pen-strep (100 U/mL–100 μg/mL). When the cells had been passaged twice, 100 μg/mL of Zeocin was added to the growth medium every passage. Passage numbers between 3 and 15 were used for all experiments.

#### B16 Cells

The B16–F10–Luc2 (B16) mouse melanoma cell line was purchased from ATCC and cultured similarly to manufacturer instructions. Briefly, the B16 cells were grown in T-75 flasks using complete culture media composed of DMEM supplemented with 2 mM l-glutamine, 10 μg/mL Blasticidin, 100 μg/mL Normocin, and 10% (v/v) FBS. Media was changed every 3–4 days. Upon reaching 80% confluence, cells were passaged using 0.25% trypsin-EDTA solution per manufacturer instructions. To establish the MDR version of this cell line, the chemotherapeutic doxorubicin (Dox) was used. A starting concentration of 1 nM Dox was added to the complete growth media, and the concentration of Dox was doubled at each passage until a final resistance concentration of 1 μM Dox was reached to obtain the multidrug-resistant cell line (MDR-B16). All cultures were maintained in a 37 °C incubator with 5% CO_2_.

### RAW-Blue NF-*k*B Assay

RAW-Blue cell activation was measured via a colorimetric enzyme reaction performed to detect secreted alkaline phosphatase (SEAP) activity expressed in the cell culture supernatant as a consequence of RAW-Blue cell activation. The assay was performed using test media composed of raw-blue cell culture media without Normocin or Zeocin (to reduce any potential for interference). To test cell activation, cell suspensions were obtained from RAW-Blue cells (passages 3 to 15) grown to 80% confluence by scraping and resuspension/homogenization in the test medium. Following counting and dilution in test media, 180 μL of cell suspension (∼100,000 cells) is added to each well of a 96-well plate. The serial dilution of synthetic compounds, ligands (100–0.01 μM), negative control, and positive control is made in each well in a flat 96-well plate by adding 20 μL of the respective stock solution for a final volume of 200 μL/well. Wells are mixed by gently pipetting up and down in each well before covering with a lid and incubating (37 °C, 5% CO_2_) for 24 h. Following incubation, 20 μL of cell supernatant from each well is added to a new 96-well plate containing 180 μL of SEAP detection reagent (QUANTI-Blue) in each well for a final volume of 200 μL/well. Importantly, the supernatant is removed without disturbing the cells attached at the bottom of each well. The plate is then incubated at 37 °C until absorbance of the positive control is near 1 (1–24 h) when measured at 620 nm. All samples are subsequently normalized to the positive and negative controls within each biological replicate.

### P-gp Membrane Vesicle Assay

The ATPase activity of the P-gp membrane vesicles in the MDR1 ATPase Kit (SOLVO Biotechnology, catalog no. SBPE01) transporting imidazoquinoline conjugates was determined by measuring inorganic phosphate (P_i_) liberated by P-gp activity. The amount of inorganic phosphate liberated is determined using a phosphate detection reagent and calibrant provided with the kit. Serial dilution of synthetic compounds was prepared by first dissolving compounds in DMSO and then performing serial dilutions such that the final concentration of organic solvent is 2% during the efflux assay. Based on control experiments, DMSO at this concentration had no effect on the ATPase activity. To prepare the assay mix, 870 μL of 10xMedium was diluted to 8700 μL by adding 7830 μL distilled water. Next, the MgATP solution was prepared by mixing 50 μL of 0.2 M MgATP and 950 μL of distilled water. Blocker solutions and Developer solutions were similarly prepared by diluting supplied stock solutions with distilled water. Membrane suspension (4 mg of membrane protein) was equilibrated with Assay mix and test drugs at 37 °C in the incubator for 10 min. Then, the ATPase reaction was started by the addition of 10 μL MgATP solution to each well except those for negative controls and phosphate calibration. The 96-well plate was incubated at 37 °C for 10 min. The ATPase reaction was stopped by adding 100 μL of developer solution at room temperature. Finally, 100 μL of blocker solution was added to the wells after 2 min at room temperature, and the plate was incubated for 30 min. P_i_ was determined by absorbance using a microplate reader (590 and 630 nm).

### Dual Efflux-Immunogenicity Assay

MDR-B16 melanoma and treatment-naive B16 cells were passaged and resuspended at a density of 1 × 10^6^ cells per tube, in a solution containing 100 μM of test compound dissolved in 200 μL of PBS. The tubes were then allowed to sit on ice for 2 h to load compounds into cells. Next, the tubes were centrifuged (two times) at 4 °C and the supernatant was carefully removed.

After centrifugation, the pellet was resuspended in 40 μL of growth media followed by incubation at 37 °C for 2 h, ensuring that the caps were slightly ajar to allow for gas exchange. Following incubation, the tubes were again centrifuged, and 10 μL of the supernatant was added to 90 μL of 1 × 10^5^ RAW-Blue cells in a 96-well plate, preparing triplicate wells for each sample. The plate was again incubated at 37 °C for 24 h. Then, 20 μL of the cell supernatant from each well was transferred to a new 96-well plate, which already had been filled with 180 μL of the QUANTI-Blue solution and incubated at 37 °C before reading absorbance at 620 nm. For controls, wells with 1% DMSO in PBS and IMQ kept on ice in parallel to experimental samples were used.

### Cell Viability Assays

The effect of imidazoquinoline conjugates on cell viability was assessed using a resazurin reduction assay. Within live cells, the blue nonfluorescent resazurin reagent is enzymatically reduced to fluorescent pink resorufin by intracellular oxido-reductases, located primarily in the mitochondria of viable cells.

For RAW-Blue cell viability, cells were seeded at a density of 1.0 × 10^5^ cells per well in 96-well plates containing 200 μL of complete test medium, and the plates were incubated (37 °C, 5% CO_2_) with compounds at a final concentration of 10 μM for 24 h before incubation with a resazurin solution as with the B16 cells below.

To assess cell viability in B16 parent cells and MDR-B16 cells, 20,000 cells/well were seeded in 96-well plates and allowed to adhere for 24 h prior to the addition of the compound. The final concentrations of tested compounds in each well were 50 μM, 5 μM, 0.5 μM, 50 nM, and 5 nM, in the presence and absence of 1 μM Dox. The compounds were dissolved in DMSO before serial dilutions in PBS, and the final DMSO concentration did not exceed 2%. 2% DMSO in PBS and 1 μM Dox were used as the negative vehicle and positive controls, respectively. Both Parent and MDR B16 cells were incubated with the compounds for 36 h. Before the addition of the resazurin solution, the old growth medium was replaced with a clear complete DMEM medium without phenol red, and 10 μL of resazurin solution was added to each well, bringing the final volume to 100 μL per well. Absorbance was taken at 570 and 600 nm after incubation with the resazurin reagent. Cells were incubated with the resazurin solution for variable time points (2–24 h) at 37 °C. The 4 h time point is shown because it captures optimal metabolic activity without signal oversaturation. Cell viability was calculated as the difference between the absorbances at these wavelengths and expressed as a percentage relative to the negative vehicle control corresponding to 100% viability.

### Flow Cytometry

Parent and MDR-B16 cells were seeded in six-well plates in complete DMEM growth medium and allowed to reach 70% confluency. Compounds **I**, **S3**, **S5** (50 μM), or verapamil (VPM, 10 μM) were then administered for 16 h before the addition of 10 μM Dox for 30 min at 37 °C. Cells were then passaged, resuspended in ice-cold PBS, and collected into microcentrifuge tubes for subsequent flow cytometric analysis using the Attune NxT flow cytometer. All runs were gated on single cell populations at 30,000 events/sample, and fluorescence corresponds to the emission of Dox upon excitation by blue laser.

### Western Blot

Lysates were collected from parent and MDR-B16 cells using 0.1% Triton X-100 with protease inhibitors, and protein concentration was quantified via BCA assay. Twenty-five μg of protein/sample was loaded on an 8% polyacrylamide gel and separated by SDS-PAGE before electrotransferring onto a PVDF membrane. The membrane was blocked in 5% nonfat milk overnight, followed by incubations with rabbit antimouse P-gp and rabbit antimouse β-actin primary antibodies for 2 h at RT, and then with HRP-conjugated goat antirabbit secondary antibodies (1 h RT). TBST washes (3 × 10 min each) were performed between each antibody incubation, and the signal was detected by chemiluminescence.

### Data Analysis and Statistics

All absorption data for bioassays were analyzed in GraphPad using dose–response stimulation with variable slope (4 parameters, curve fit). Microsoft Excel was used for the statistical analysis, applying ANOVA: single factor (α = 0.05) and two-tailed *t* tests assuming unequal variance to examine the significant difference between the treated cells and the positive controls.

## Supporting Information Available

Docking of imidazoquinoline derivatives (Figure S1) and associated molecular properties (Tables S1 and S2), more detailed biological studies including immunogenicity, efflux, cytotoxicity, Dox sensitization, and comparison to in-silico predictions (Figures S2–S7), molecular characterization data (Figures S8–S103), statistical significance calculations (Table S3), and additional experimental protocols and synthetic transformations (Schemes S1–S4) (PDF)
