# Supplementary material for: Efflux-Enhanced Imidazoquinolines To Exploit Chemoresistance
Source: ACS Omega. 2025 Mar 17;10(12):12319–33. doi: 10.1021/acsomega.4c11297 (PMC11966297; doi:10.1021/acsomega.4c11297)
Supplement: Supplementary file 1 — ao4c11297_si_001.pdf [file ao4c11297_si_001.pdf]

Efflux-Enhanced Imidazoquinolines to Exploit Chemoresistance

Muhammad Haroon,[1Ψ] Sharmin Sultana,[1Ψ] Seyedeh A. Najibi,[1] Emily T. Wang,[1] Abbey Michaelson,[1] Pranto S.M. Al Muied,[1] Amy E. Nielsen,[2] Rock J. Mancini[1,2]*

[1] Department of Chemistry and Biochemistry, Miami University, 651 E. High Street, Oxford, OH 45056, USA

[2] Astante Therapeutics Inc., 201 E. Fifth Street, Cincinnati, OH, 45202, USA

[Ψ] Authors contributing equally to this work.

*** Corresponding Author:** Rock J Mancini: mancinr@miamioh.edu

Table of Contents

Figure S1: Docking of imidazoquinoline conjugates 2-8

Figure S2: Immunogenicity and P-gp efflux of imidazoquinoline conjugates 9

Figure S3: Conjugate cytotoxicity on RAW-Blue Cells..10

Figure S4: Dox-mediated killing of (MDR) melanoma with Amide Conjugates 11

Figure S5: Dox-mediated killing of (MDR) melanoma with Sulfonamide Conjugates ...12

Figure S6: Comparison of Conjugate-induced Dox retention 13

Figure S7: Correlation of Binding energy (kcal/mol) and Efflux susceptibility (ΔPi).....14

Table S1: Conjugate predicted P-gp binding 15

Table S2: Conjugate physicochemical properties 16

Table S3: Statistical confidence for enhanced conjugate activity relative to **I** 17

Synthetic Experimental Details of Scheme S1-S4: 18-23

Figure S8-S106: Characterization Data: ^{1}H NMR, ^{13}C NMR, and ESI-MS 24-86

References: 87

Figure S1. Molecular Docking of Imidazoquinoline Conjugates**.** To validate the interaction of proposed ligands with P-gp, molecular docking simulations were performed between P-gp and compounds (**A1-A12, S1-S13**) using reported literature. All docking studies were performed using Autodock Vina (ver. 1.2.3). The crystal structure of P-gp (PDB ID: 3G60) was retrieved from protein data bank. Co-crystallized ligands and water molecules were removed and the protein was converted to pdbqt format using Autodock Tools keeping co-factor intact. The 2D structures of ligands were drawn using Chemdraw 12.0 and converted to 3D format by Openbabel (ver. 2.3.1). PDBQT files were prepared in MGL Tools. All the compounds were docked using Autodock Vina. The other parameters were left as default. Discovery Studio (ver. 21.1.0.20298) and LigPlot software were used for 3D molecular graphics, structural alignments, and visualizations of 2D and 3D images showing interactions of conjugates with the P-gp protein.

**Compound**	**3D images**	**2D images**
**A1**	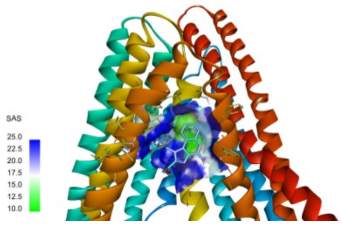	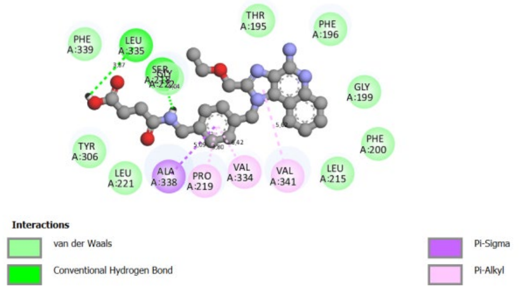
**A2**	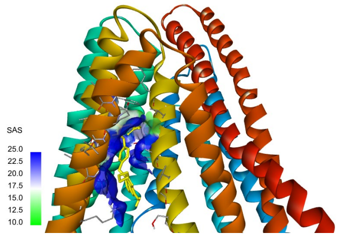	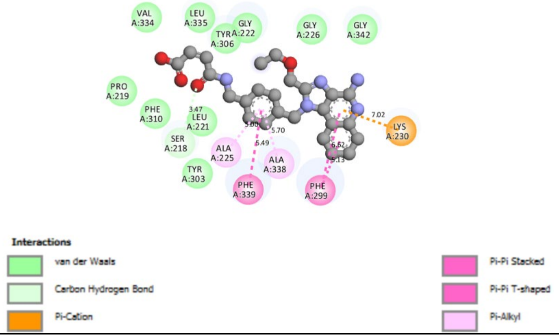

<table>
<tr><td>A3</td><td>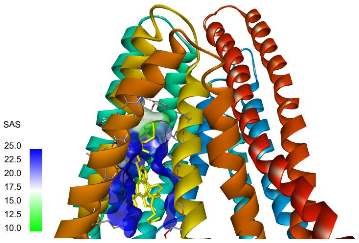
</td><td>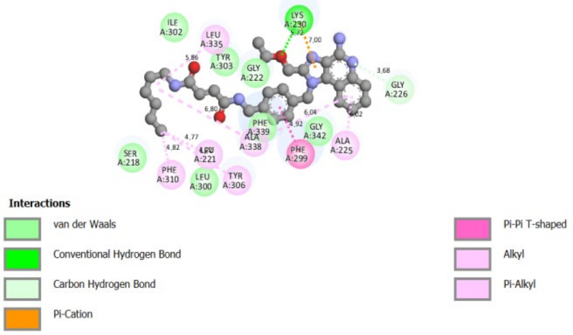
</td></tr>
<tr><td>A4</td><td>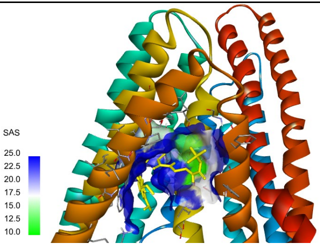
</td><td>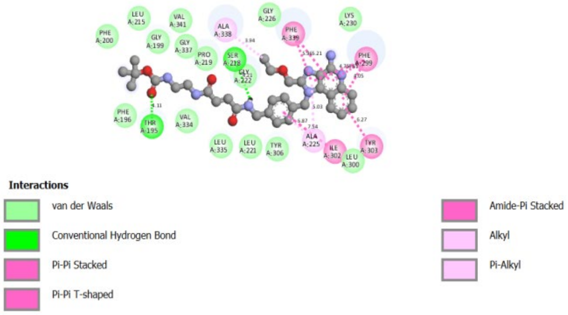
</td></tr>
<tr><td>A5</td><td>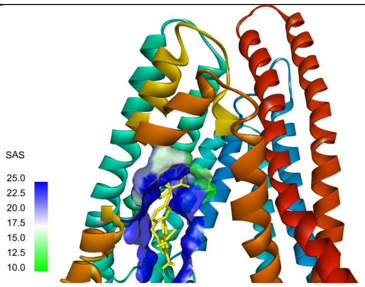
</td><td>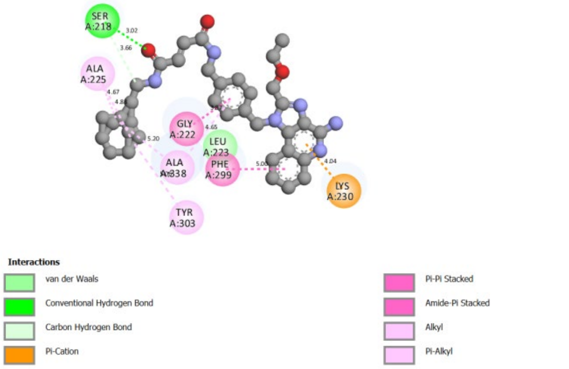
</td></tr>
<tr><td>A6</td><td>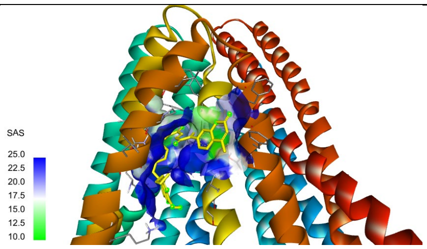
</td><td>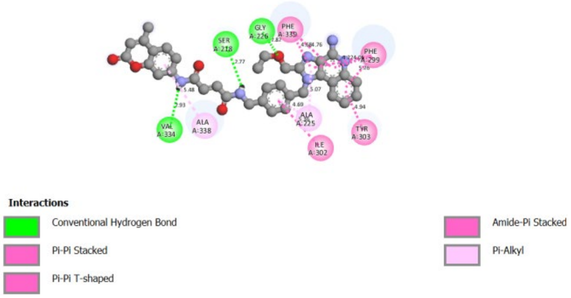
</td></tr>
</table>

A7

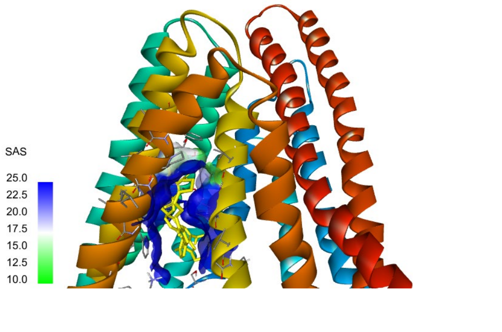
SAS
25.0
22.5
20.0
17.5
15.0
12.5
10.0

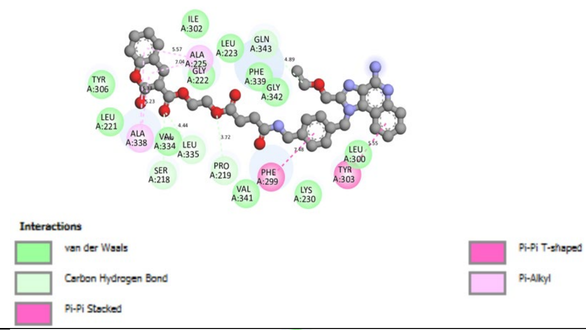
Interactions
van der Waals
Carbon Hydrogen Bond
Pi-Pi Stacked
Pi-Pi T-shaped
Pi-Alkyl

A8

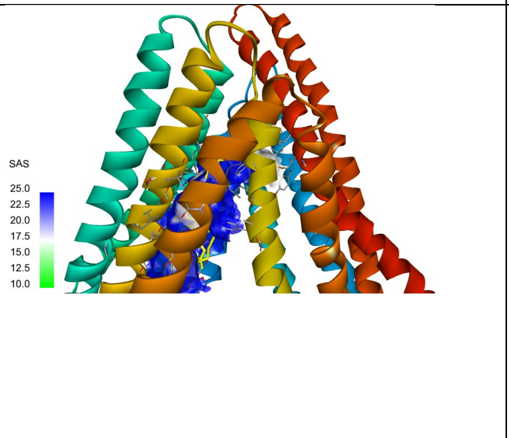
SAS
25.0
22.5
20.0
17.5
15.0
12.5
10.0

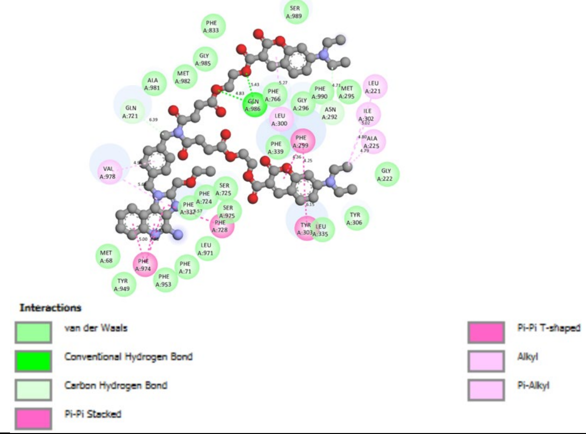
Interactions
van der Waals
Conventional Hydrogen Bond
Carbon Hydrogen Bond
Pi-Pi Stacked
Pi-Pi T-shaped
Alkyl
Pi-Alkyl

A9

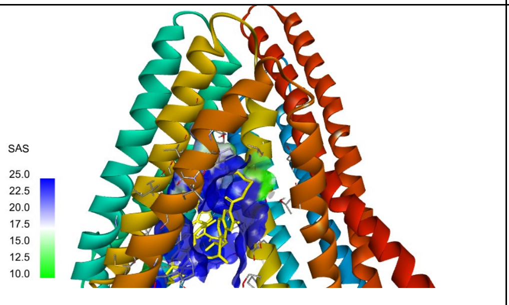
SAS
25.0
22.5
20.0
17.5
15.0
12.5
10.0

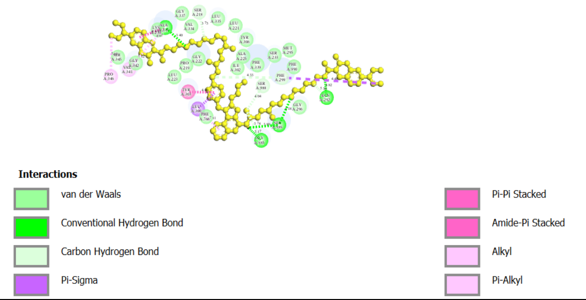
Interactions
van der Waals
Conventional Hydrogen Bond
Carbon Hydrogen Bond
Pi-Sigma
Pi-Pi Stacked
Amide-Pi Stacked
Alkyl
Pi-Alkyl

A10

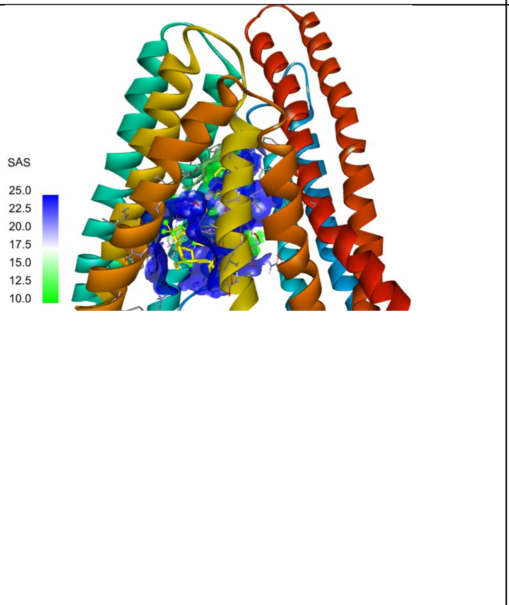
SAS
25.0
22.5
20.0
17.5
15.0
12.5
10.0

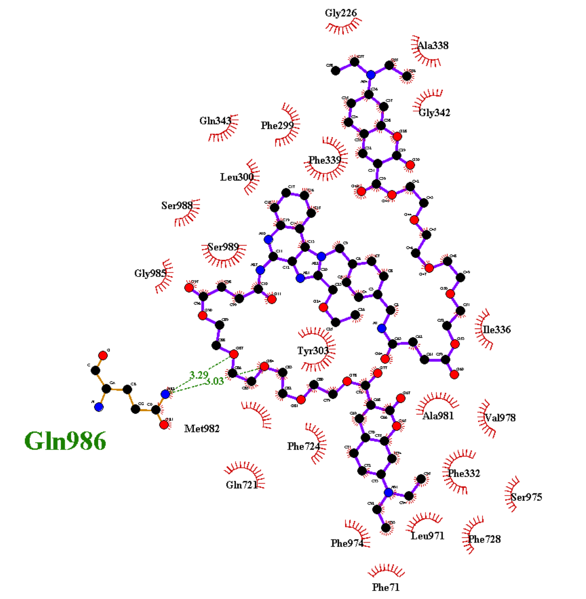
Gly226
Ala338
Gly342
Gln343
Phe299
Phe339
Leu300
Ser988
Ser989
Gly985
Ile336
Tyr303
3.29
3.03
Gln986
Met982
Ala981
Val978
Phe724
Phe332
Gln721
Ser975
Phe974
Leu971
Phe728
Phe71

<table>
<tr><td>**A11**</td><td>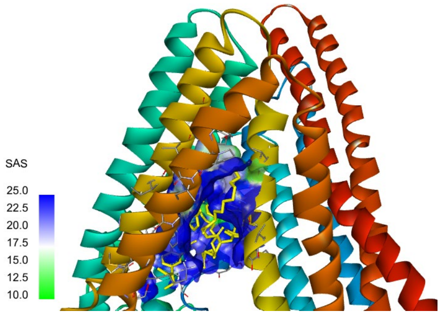</td><td>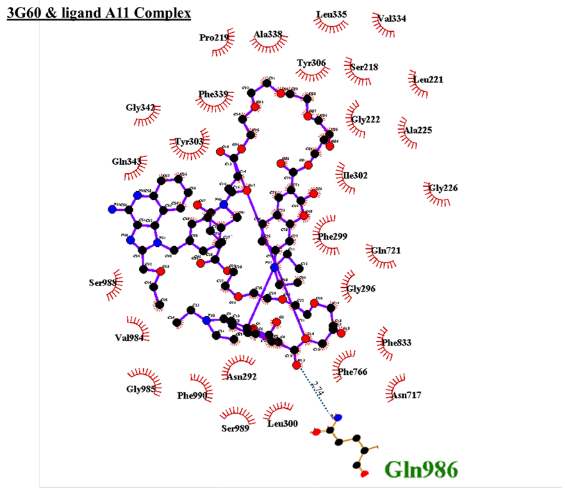</td></tr>
<tr><td>**A12**</td><td>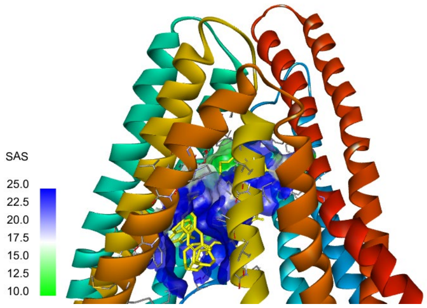</td><td>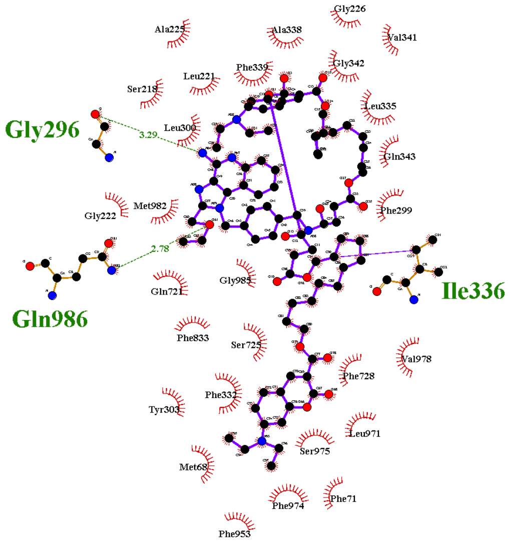</td></tr>
<tr><td>**S1**</td><td>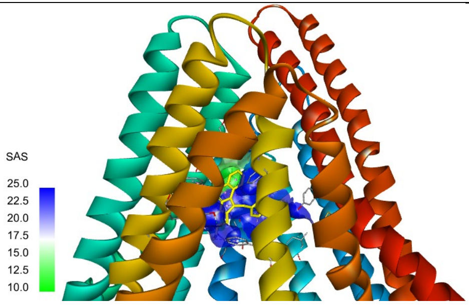</td><td>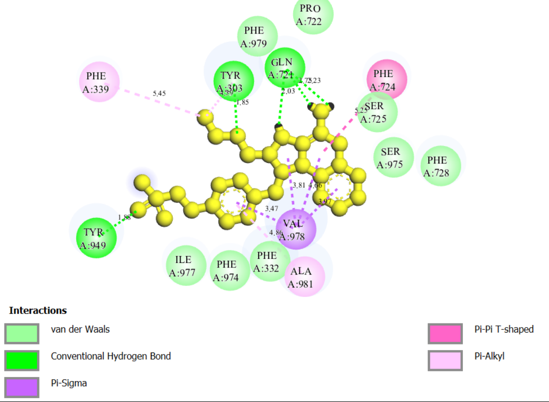</td></tr>
</table>

S2

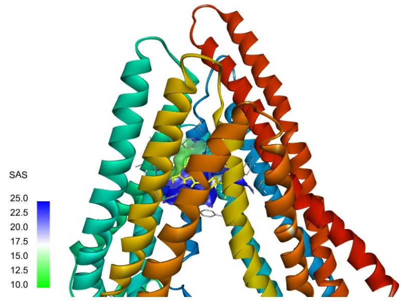
SAS
25.0
22.5
20.0
17.5
15.0
12.5
10.0

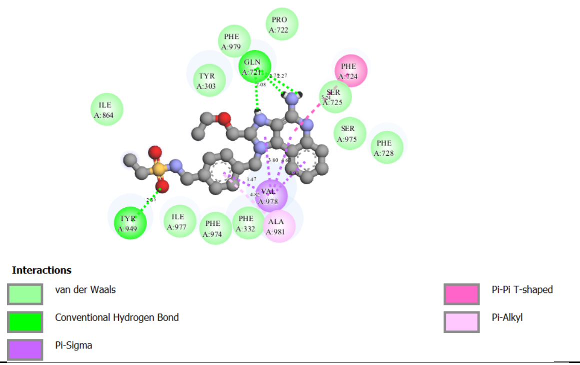
Interactions
van der Waals
Conventional Hydrogen Bond
Pi-Sigma
Pi-Pi T-shaped
Pi-Alkyl

S3

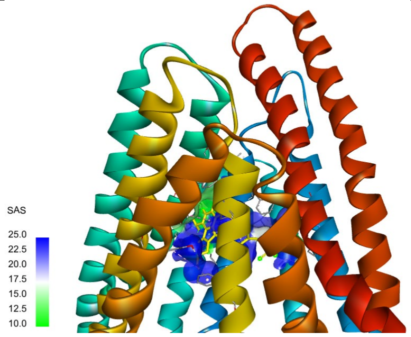
SAS
25.0
22.5
20.0
17.5
15.0
12.5
10.0

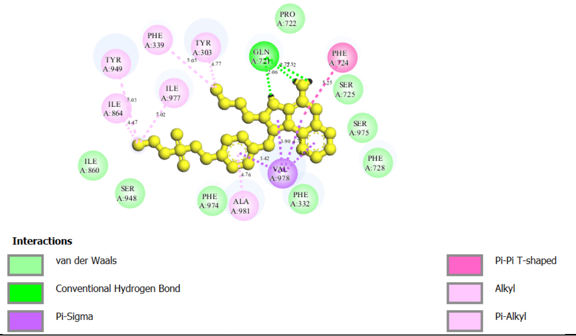
Interactions
van der Waals
Conventional Hydrogen Bond
Pi-Sigma
Pi-Pi T-shaped
Alkyl
Pi-Alkyl

S4

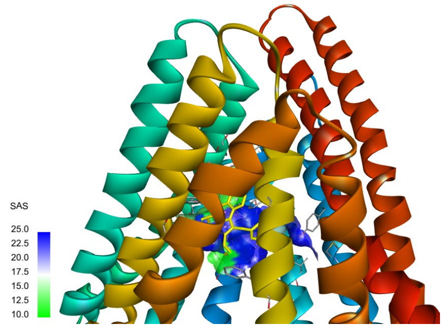
SAS
25.0
22.5
20.0
17.5
15.0
12.5
10.0

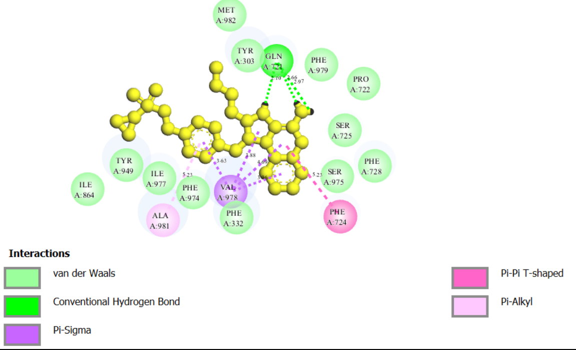
Interactions
van der Waals
Conventional Hydrogen Bond
Pi-Sigma
Pi-Pi T-shaped
Pi-Alkyl

S5

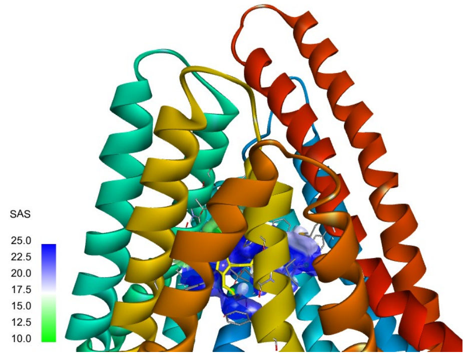
SAS
25.0
22.5
20.0
17.5
15.0
12.5
10.0

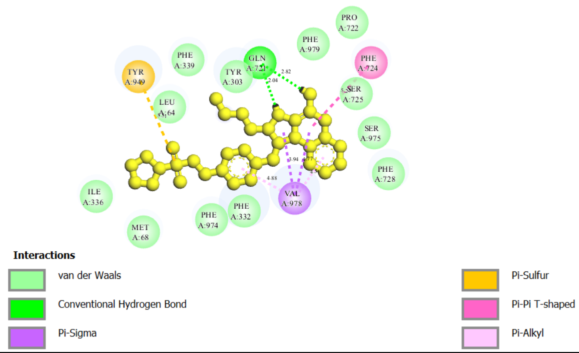
Interactions
van der Waals
Conventional Hydrogen Bond
Pi-Sigma
Pi-Sulfur
Pi-Pi T-shaped
Pi-Alkyl

<table>
<tr><td>S6</td><td>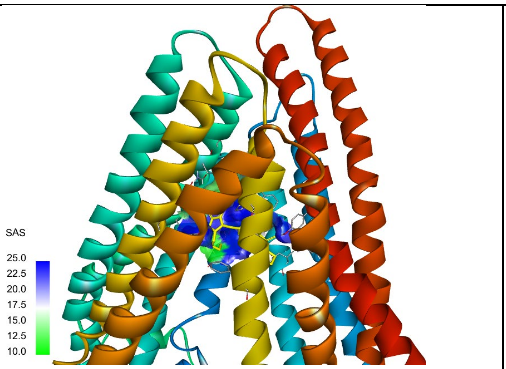</td><td>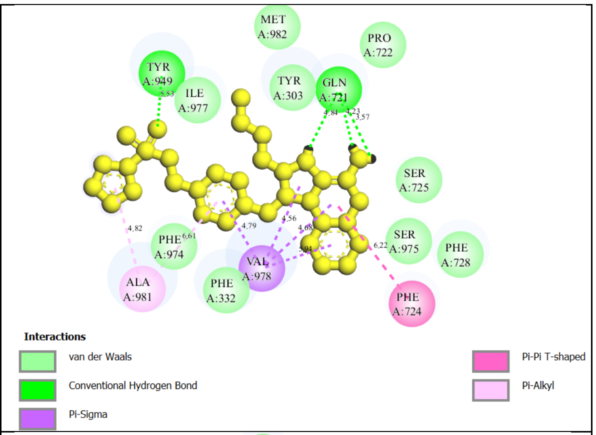</td></tr>
<tr><td>S7</td><td>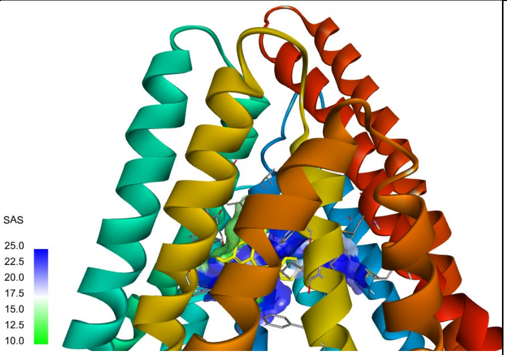</td><td>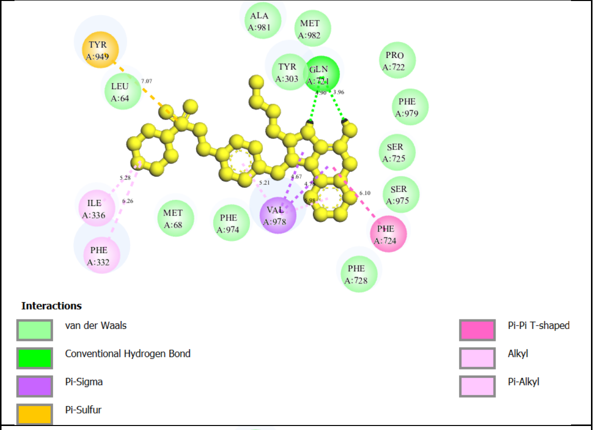</td></tr>
<tr><td>S8</td><td>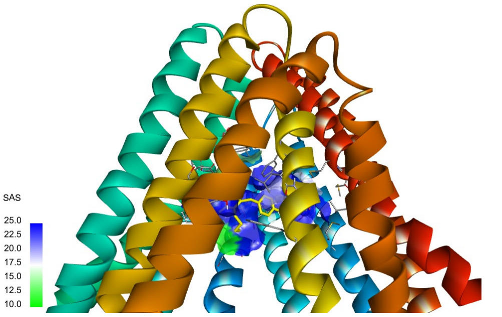</td><td>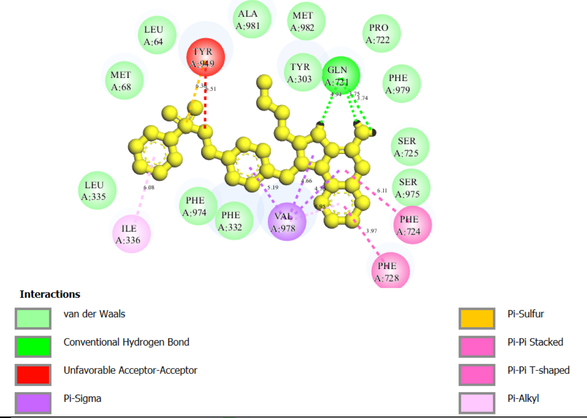</td></tr>
<tr><td>S9</td><td>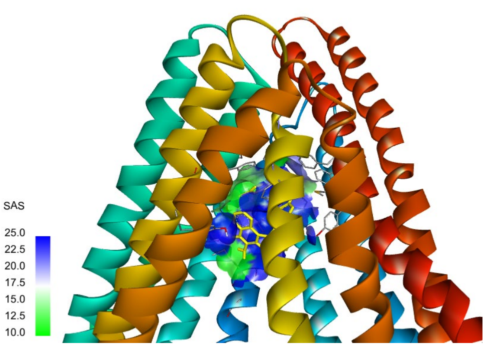</td><td>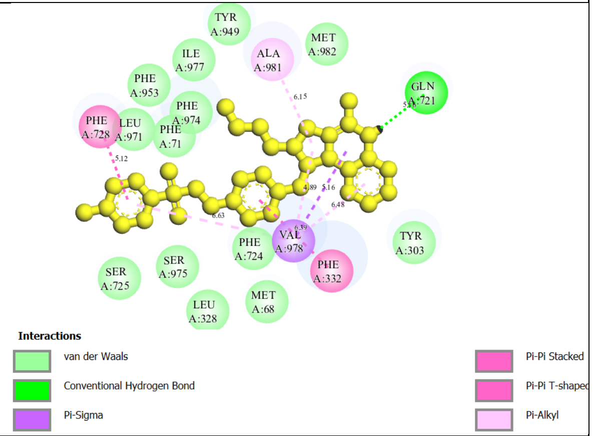</td></tr>
</table>

S10

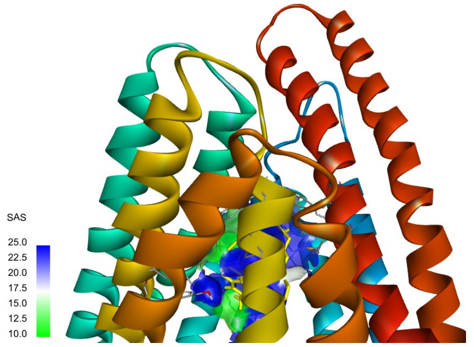

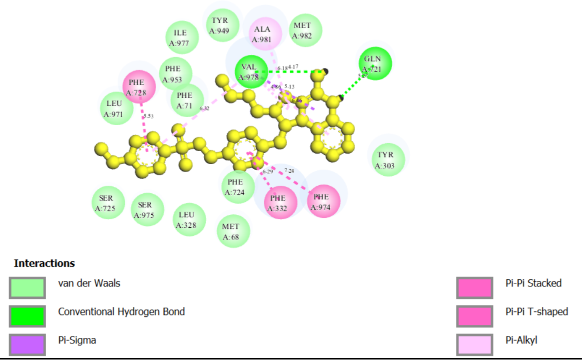

S11

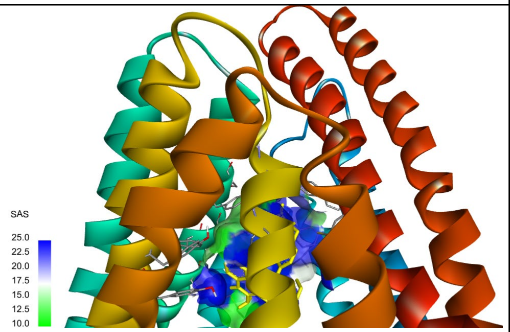

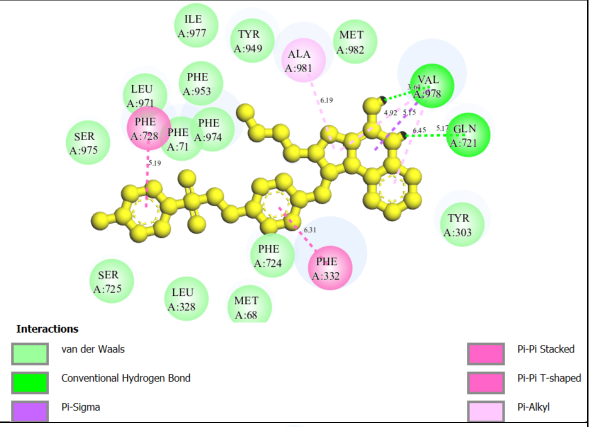

S12

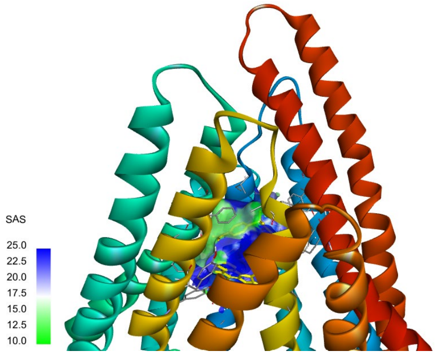

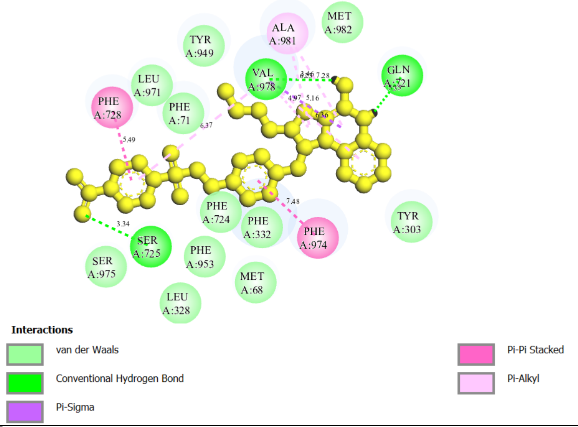

S13

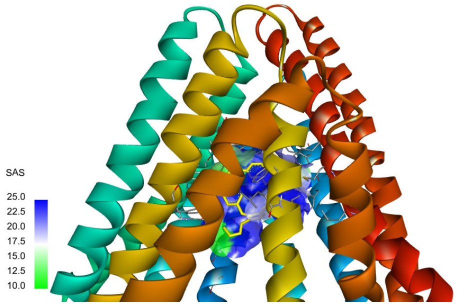

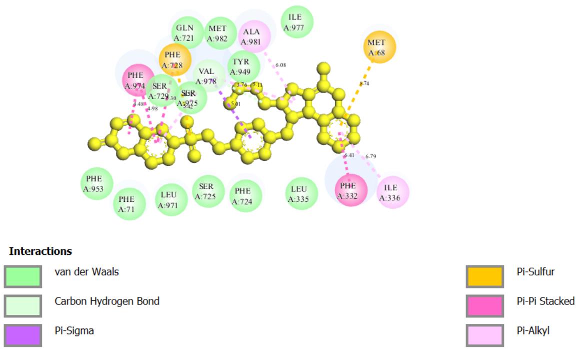

Figure S2: Immunogenicity and P-gp efflux susceptibility of imidazoquinoline conjugates. **a)** NF-*k*B activation of amide-conjugates was measured in Raw-Blue cells following detection of corresponding alkaline phosphatase activity using a colorimetric assay (Abs. 620 nm). Data points are the mean values of experiments repeated in triplicate and normalized relative to vehicle and positive control (**I**) at the indicated concentrations. *$p<0.05$, ***$p<0.001$ for **A11** relative to (**I**). **b)** NF-*k*B activation of sulfonamide-conjugates was measured in Raw-Blue cells as in part a. Data points are the mean values of experiments repeated in triplicate and normalized relative to vehicle and positive control (**I**) at 10 μM. *$p<0.05$, **$p<0.01$, ***$p<0.001$ for potency of **S5** and **S3** relative to (**I**) at the indicated concentrations. **c)** P-gp efflux susceptibility was examined by membrane vesicle assay measuring inorganic phosphorus (P_i) as a consequence of ATP-dependent P-gp efflux. Data points are the mean of experiments performed in triplicate and plotted as the difference relative to (**I**) positive control (ΔP_i) for each compound relative to normalized NF-*k*B measured in the Raw-Blue assay at 10 μM for all compounds. **d**) Because the sulfonamides exhibited irregular dose-response behavior, efflux susceptibility relative to (**I**) was also related to potency using ΔpEC_{50}.

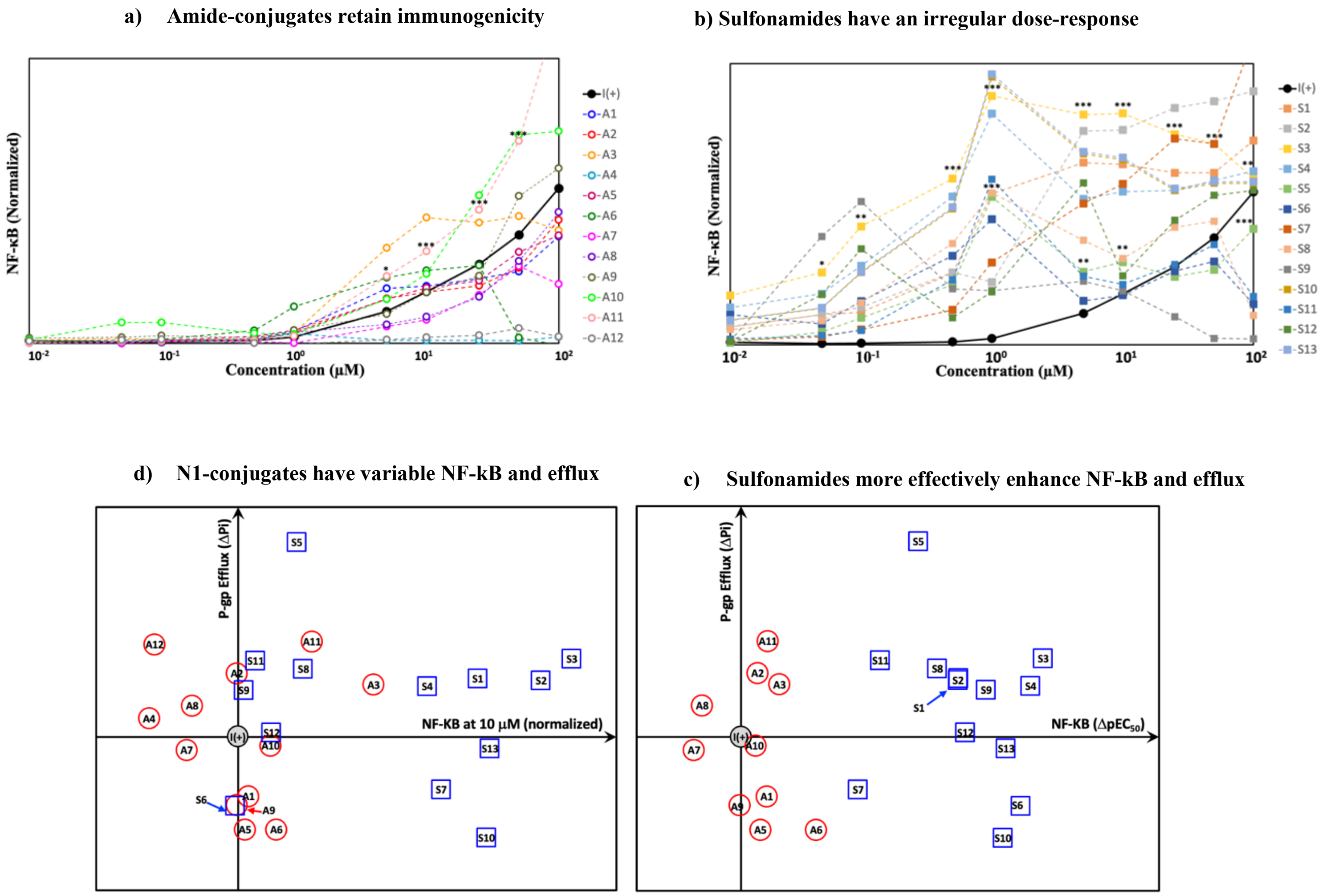

Figure S3. Conjugate cytotoxicity on RAW-Blue Cells. Cells were seeded at a density of a 100,000 cells/well in a 96-well plate using complete media without phenolphthalein indicator. and incubated overnight before treatment. Cells were treated with synthesized compounds for 24 h to assess cell viability. Resazurin solution (10% of the culture volume) was added, and the relative absorbance was measured to assess cell viability. PBS was used as a negative control. Data are presented as the mean ± SD from three independent experiments.

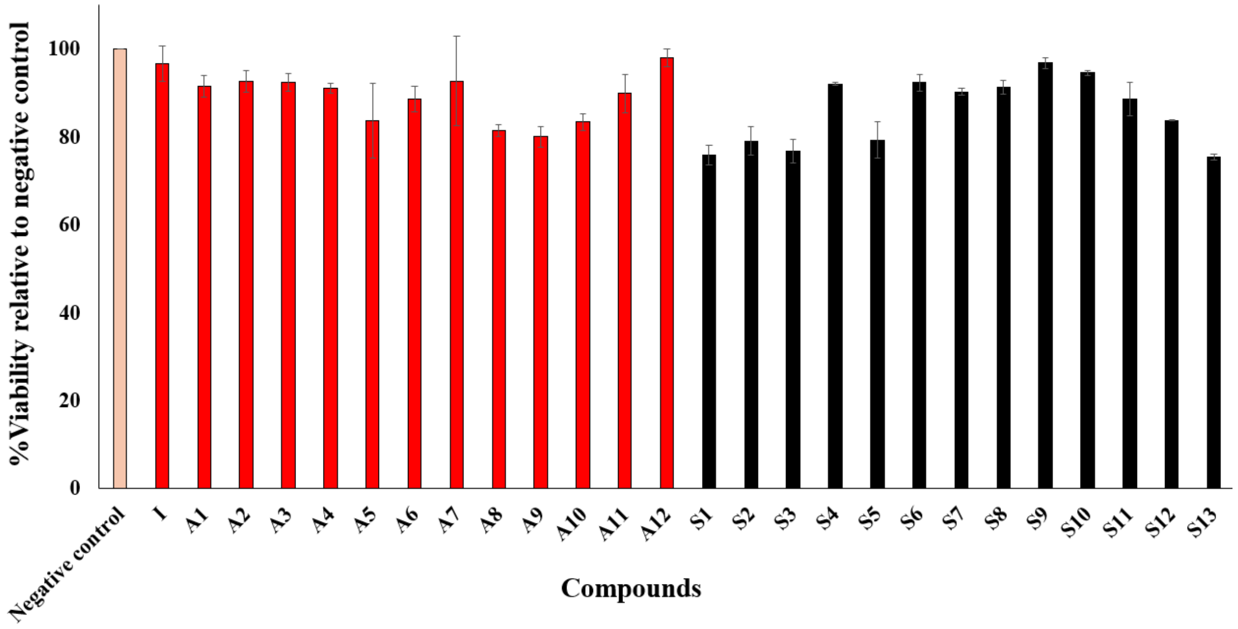

Figure S4: Cell viability assay of (**a**) B16 and (**b**) MDR-B16 cells treated with amide conjugates with or without doxorubicin (DOX) at the indicated concentrations for 36 hours. Cells were seeded at a density of a 2x10[4] cells/well in a 96-well plate and incubated overnight before treatment. Resazurin solution (10% of the culture volume) was added, and the relative absorbance was measured to assess cell viability. PBS was used as a negative control. Values represent the mean ± SD from three independent experiments.

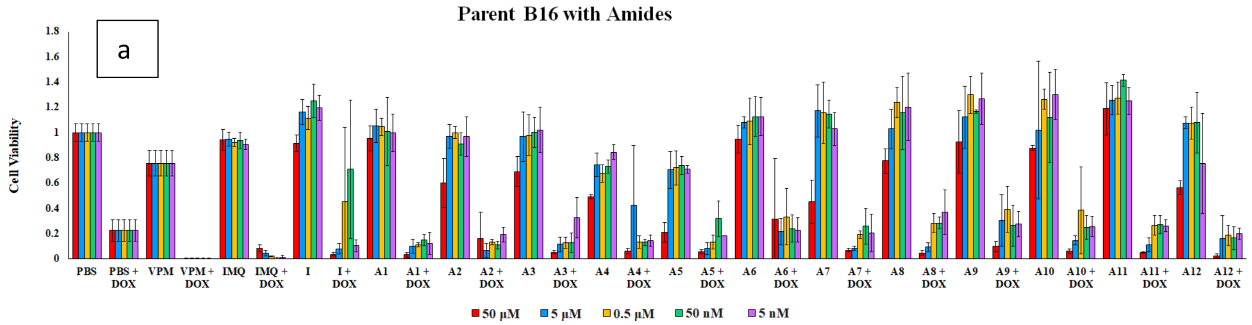

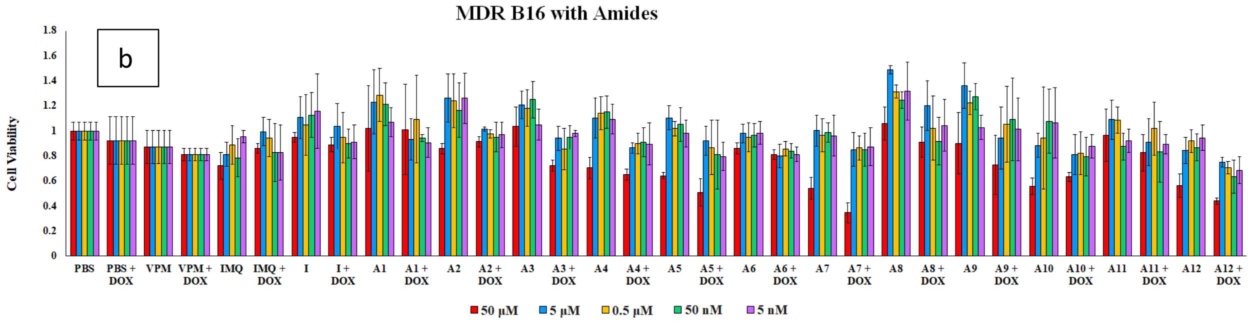

Figure S5: Cell viability assay of (**a**) B16 and (**b**) MDR-B16 cells treated with sulfonamide conjugates with or without doxorubicin (DOX) at different concentrations for 36 hours. Cells were seeded at a density of 2x10^{4} cells/well in a 96-well plate and incubated overnight before treatment. Resazurin solution (10% of the culture volume) was added, and the relative absorbance was measured to assess cell viability. PBS was used as a negative control. Values represent the mean ± SD from three independent experiments.

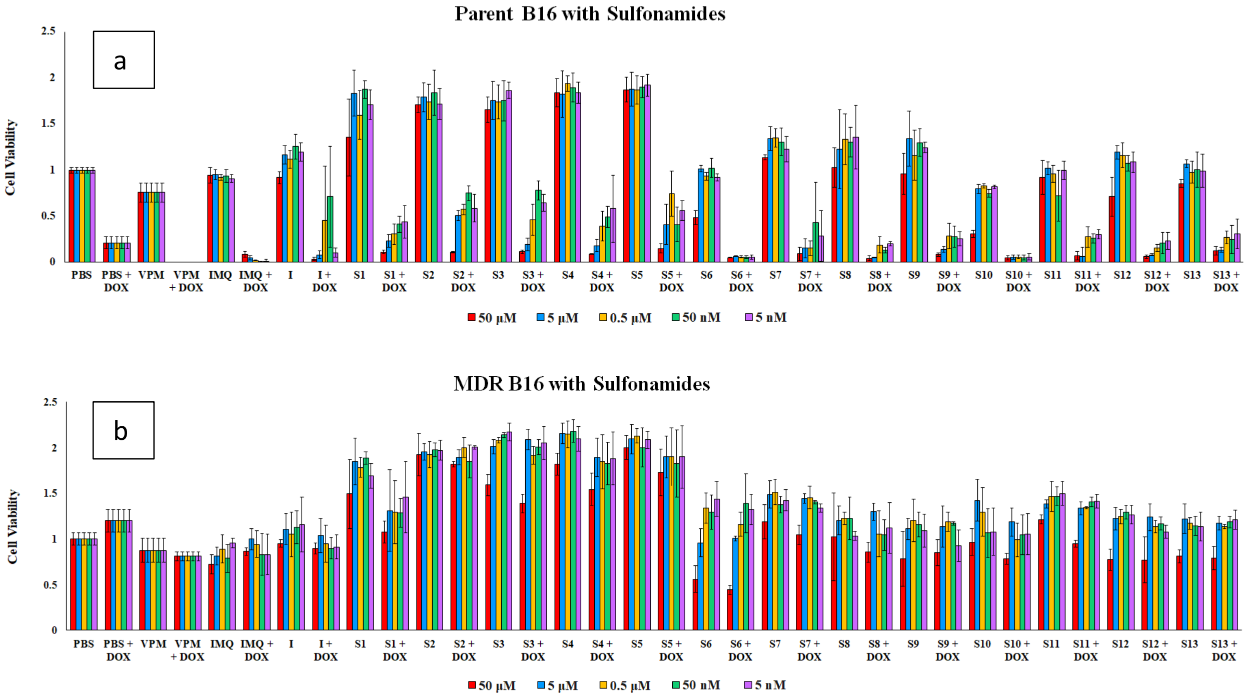

Figure S6: Flow cytometry readouts of DOX fluorescence in parent and MDR B16 cells following pre-treatment with 50 µM of compounds **S3**, **S5**, and **I**, or with 10 µM verapamil (**a** known P-gp inhibitor). Data are represented as mean fluorescent intensities (MFI).

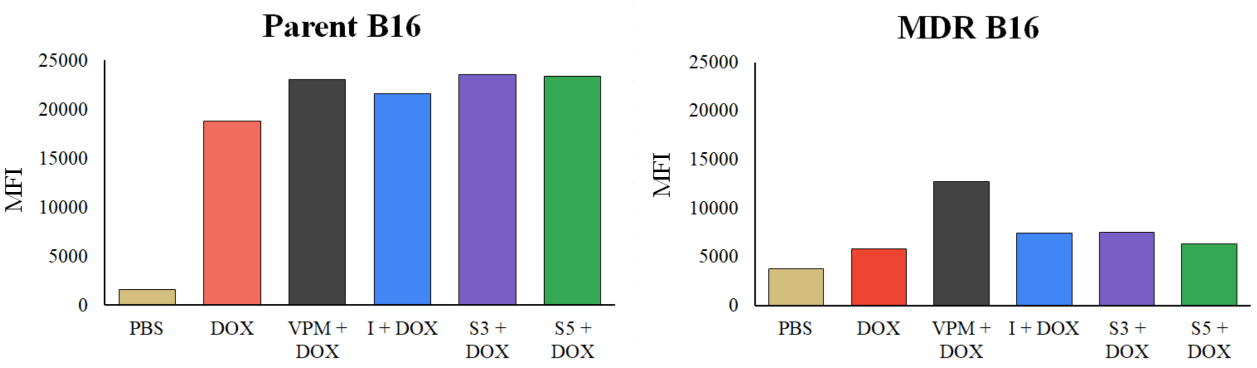

Figure S7: Correlation of Binding energy (kcal/mol) and Efflux pump susceptibility (ΔPi)

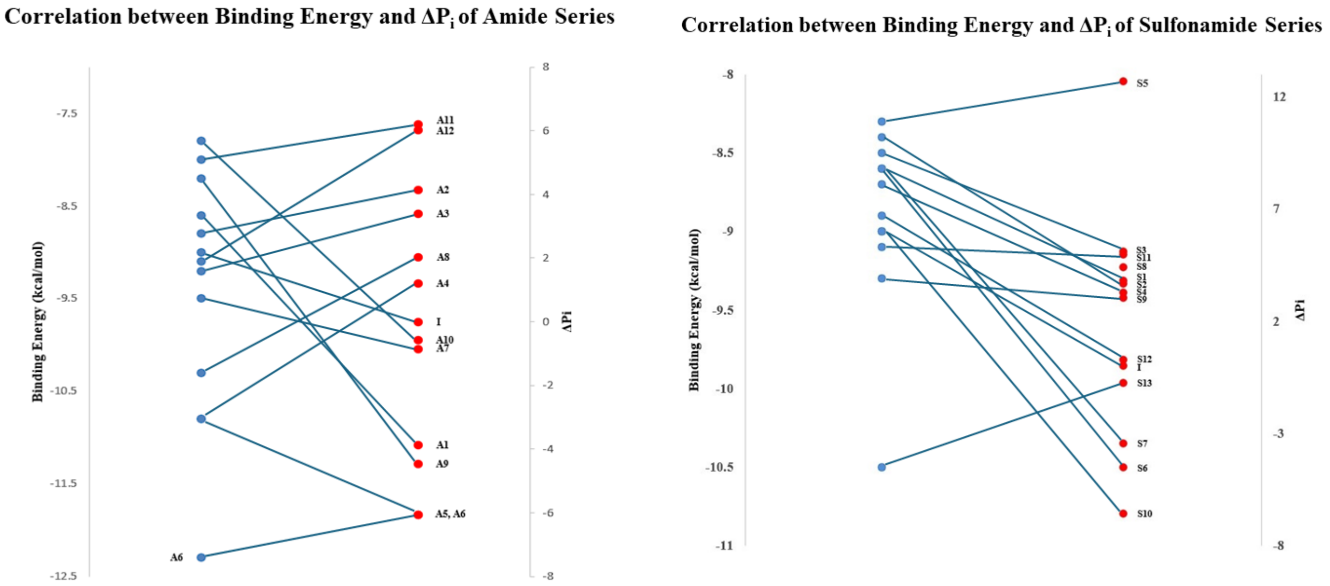

Table S1. Swiss ADME P-gp predictions and binding energies from the docking study.

Conjugates	P-gp substrate	Binding energy kcal/mol	Conjugates	P-gp substrate	Binding energy kcal/mol
**A1**	Yes	-8.6	**S1**	Yes	-8.6
**A2**	Yes	-8.8	**S2**	Yes	-8.4
**A3**	Yes	-9.2	**S3**	Yes	-8.5
**A4**	Yes	-10.8	**S4**	Yes	-8.7
**A5**	Yes	-10.8	**S5**	Yes	-8.3
**A6**	No	-12.3	**S6**	Yes	-8.6
**A7**	No	-9.5	**S7**	Yes	-8.6
**A8**	Yes	-10.3	**S8**	Yes	-9.0
**A9**	Yes	-8.2	**S9**	Yes	-9.3
**A10**	Yes	-7.8	**S10**	Yes	-9.0
**A11**	Yes	-8.0	**S11**	No	-9.1
**A12**	Yes	-9.1	**S12**	Yes	-8.9
			**S13**	No	-10.5

Table S2: Physicochemical properties of imidazoquinoline conjugates generated by Swiss ADME.

Compound	MW (g/mol)	HBD	HBA	clogP	TPSA < 140 (Å²)	Rotatable bonds	Lipinski	Veber
**I**	361.44	2	4	2.45	91.98	6	yes	yes
**A1**	461.51	3	6	2.3	132.36	11	Yes	No
**A2**	459.5	3	6	2.25	132.36	10	Yes	Yes
**A3**	542.67	3	5	3.97	124.16	16	Yes	No
**A4**	601.7	4	7	2.89	162.49	17	No	No
**A5**	606.76	3	5	4.38	124.16	13	Yes	No
**A6**	616.67	3	7	3.82	154.37	12	No	No
**A7**	677.7	2	10	3.93	177.87	17	No	No
**A8**	1136.21	2	17	6.78	270.24	34	No	No
**A9**	1312.42	2	21	6.97	307.16	46	No	No
**A10**	1400.52	2	23	7.15	325.62	52	No	No
**A11**	1488.63	2	25	7.49	344.08	58	No	No
**A12**	1416.72	2	17	13.3	270.24	54	No	No
**S1**	439.53	2	6	2.17	120.51	8	Yes	Yes
**S2**	453.56	2	6	2.69	120.51	9	Yes	Yes
**S3**	467.58	2	6	2.92	120.51	10	Yes	Yes
**S4**	465.57	2	6	2.66	120.51	9	Yes	Yes
**S5**	494.61	2	7	2.66	123.75	9	Yes	Yes
**S6**	507.63	2	6	3.53	148.75	9	Yes	Yes
**S7**	508.68	2	7	2.93	123.75	9	Yes	Yes
**S8**	501.6	2	6	3.45	120.51	9	Yes	Yes
**S9**	515.63	2	6	3.75	120.51	9	Yes	Yes
**S10**	531.63	2	7	3.49	129.74	10	Yes	Yes
**S11**	536.05	2	6	4.01	120.51	9	Yes	Yes
**S12**	548.59	3	9	2.75	183.06	10	No	No
**S13**	569.63	2	8	3.56	150.72	9	Yes	No

Table S3: Statistical confidence represented as p-values (demarcated as $p < 0.001$, 0.01, 0.05, and n.s.) for conjugate activity relative to parent compound I. Experiments were performed in triplicate biological replicates at each concentration.

**Compounds**	**p-value**									
**Amides**	**100uM**	**50uM**	**25uM**	**10uM**	**5uM**	**1uM**	**0.5uM**	**0.1uM**	**0.05uM**	**0.01uM**
**A1**	0.003	0.006	0.02	ns	ns	0.001	0.001	0.002	0.0006	0.001
**A2**	0.001	0.01	ns	ns	ns	0.0003	0.0004	0.002	0.001	0.001
**A3**	ns	0.00002	ns	0.006	0.01	0.0007	0.0006	0.002	0.0001	0.001
**A4**	0.002	0.002	0.002	0.002	0.001	0.001	0.0006	0.001	0.003	0.001
**A5**	0.00008	0.002	0.04	ns	ns	0.0003	0.0007	0.002	0.0008	0.001
**A6**	0.0008	0.0003	0.04	0.006	0.04	0.01	0.001	0.002	0.001	0.001
**A7**	ns	0.003	ns	0.001	0.0006	0.0001	0.002	0.002	0.001	0.001
**A8**	0.009	0.01	ns	0.002	0.0001	0.0007	0.00004	0.001	0.0001	0.001
**A9**	0.0006	0.0002	ns	ns	0.02	0.007	0.006	0.006	0.007	0.007
**A10**	0.03	0.002	0.00009	0.04	ns	0.008	0.004	0.01	ns	0.006
**A11**	0.0003	0.0000005	0.0009	0.0001	0.05	0.00002	0.0005	0.0003	0.0001	0.001
**A12**	0.006	0.002	0.008	0.003	0.006	0.006	0.005	0.006	0.007	0.006
**Sulfonamides**	**100uM**	**50uM**	**25uM**	**10uM**	**5uM**	**1uM**	**0.5uM**	**0.1uM**	**0.05uM**	**0.01uM**
**S1**	0.000002	0.00003	0.000008	0.007	0.01	0.009	ns	ns	0.01	0.001
**S2**	0.000003	0.0003	0.003	0.000009	0.0006	ns	ns	ns	0.02	0.02
**S3**	0.006	0.001	0.00001	0.00002	0.00008	0.00003	0.00006	0.003	0.02	ns
**S4**	0.01	0.00004	0.0001	0.0002	0.001	0.003	0.0001	0.005	ns	0.01
**S5**	0.0001	0.006	0.03	0.003	0.01	0.00008	ns	0.009	0.001	0.003
**S6**	ns	0.004	0.02	ns	ns	0.01	0.01	ns	0.01	0.03
**S7**	0.002	0.001	0.01	0.0004	0.001	ns	0.009	0.0003	0.0002	0.0006
**S8**	ns	0.0001	ns	0.04	0.0007	0.02	0.02	0.01	0.001	0.0001
**S9**	0.006	0.006	0.01	ns	ns	ns	ns	0.01	ns	0.007
**S10**	0.0008	0.001	0.0002	0.0007	0.000001	0.0007	0.00005	0.004	ns	0.001
**S11**	ns	0.001	0.01	ns	ns	0.001	ns	0.01	0.007	0.002
**S12**	ns	ns	0.005	ns	0.01	ns	ns	ns	ns	0.0007
**S13**	0.0001	0.0002	0.00005	0.00008	0.009	0.0001	0.0009	0.03	ns	0.009

Experimental

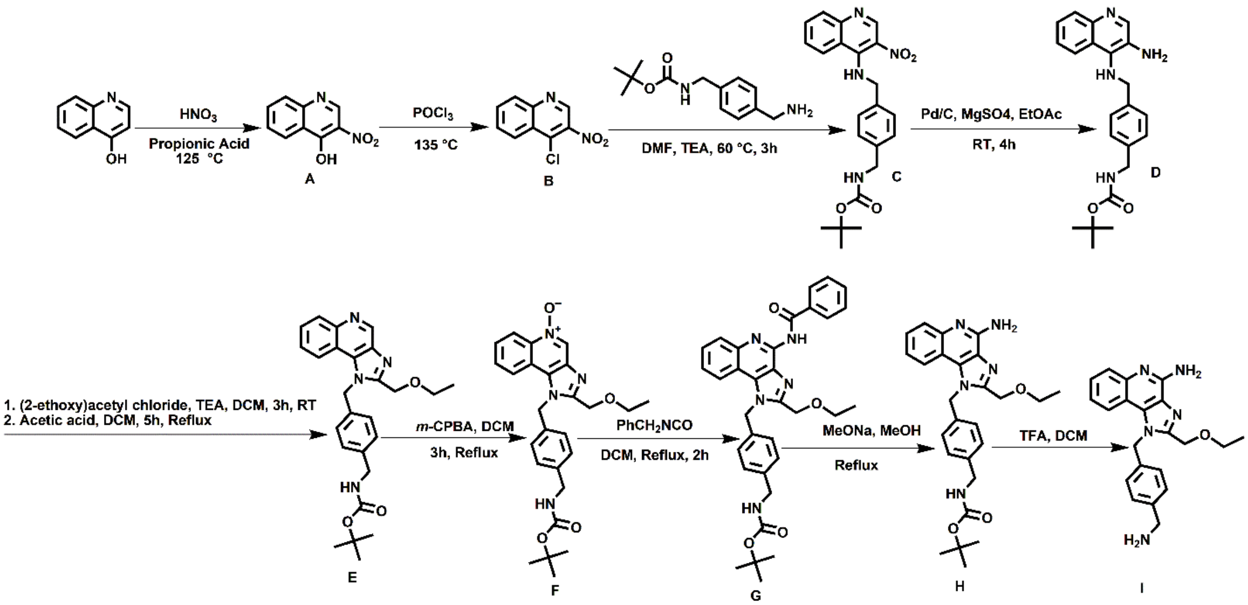

Scheme S1: Synthetic route to obtain main intermediate **I** similar to literature procedure**.**

General synthetic method for the synthesis of 2a-2e

To the solutions of Coumarin acids (**1a**= 500 mg, 2.63 mmol, **1b**= 500 mg, 1.63 mmol) in dichloromethane (20 mL) were added corresponding glycols (ethylene glycol, triethylene glycol, tetraethylene glycol and pentaethylene glycol= 15 equivalent each), EDCI (**1a**= 500 mg, 2.64 mmol, **1b**= 500 mg, 1.64 mmol) and DMAP (**1a**= 160 mg, 1.31 mmol, **1b**= 100 mg, 0.81 mmol). The mixture was stirred for 6 h at room temperature, and then, diluted with dichloromethane (20 mL). The organic layer was collected with solvent extraction. The organic layer was washed with water and brine, dried with anhydrous $MgSO_4$, and concentrated *in vacuo*. The crude product was purified with gradient column chromatography using DCM: MeOH solvent system.

2-Hydroxyethyl 2-oxo-2*H*-chromene-3-carboxylate (2a)

For synthesis, physical properties, and spectroscopic data of **2a** see Joel Alvim Jr *et. al.*[1]

2-Hydroxyethyl 7-(diethylamino)-2-oxo-2*H*-chromene-3-carboxylate (2b)

For synthesis, physical properties, and spectroscopic data of **2b** see Jinyui Xu *et. al.*[2]

2-(2-(2-Hydroxyethoxy)ethoxy)ethyl 7-(diethylamino)-2-oxo-2*H*-chromene-3-carboxylate (2c)

For synthesis, physical properties, and spectroscopic data of **2c** see Jinyui Xu *et. al.*[2]

2-(2-(2-(2-Hydroxyethoxy)ethoxy)ethoxy)ethyl 7-(diethylamino)-2-oxo-2*H*-chromene-3-carboxylate (2d)

Yield: 75%; R_f: 0.61 (DCM:MeOH; 99:1); ^{1}H NMR (400 MHz, $CDCl_3$): δ 8.45 (s, 1H, Coumarin ring-H), 7.38 (d, 1H, Coumarin ring-H, *J*= 8.8 Hz), 6.63 (dd, 1H, Coumarin ring-H, *J*= 8.8, 2.0 Hz), 6.45 (d, 1H, Coumarin ring-H, *J*= 2.0 Hz), 4.46 (t, 2H, -O-CH_2-CH_2-, *J*= 4.8 Hz), 3.84 (t, 2H, -O-CH_2-CH_2-, *J*= 4.8 Hz), 3.74-3.67 (m, 10H, 5x-CH_2-), 3.60 (t, 2H, -CH_2-CH_2-, *J*= 4.8 Hz), 3.46 (q, 4H, 2x-CH_2-CH_3, *J*= 7.0 Hz), 1.23 (t, 6H, 2x-CH_2-CH_3, *J*= 7.0 Hz); ^{13}C NMR (100 MHz, $CDCl_3$): δ 163.9 (C=O ester), 158.5 (C=O ketone), 158.2, 152.8, 149.4, 131.2, 109.7, 108.6, 107.8, 96.9 (Coumarin ring-C), 77.2, 72.5, 70.6, 70.5, 70.4, 69.2, 64.1, 61.7 (8x-O-CH_2-), 45.4 (-CH_2-N-), 12.4 (-CH_3); ESI/MS (m/z): $[M+H]^+_{theoretical}$: 438.2; $[M+H]^+_{found}$: 438.2.

2-(2-(2-(2-(2-Hydroxyethoxy)ethoxy)ethoxy)ethoxy)ethyl 7-(diethylamino)-2-oxo-2*H*-chromene-3-carboxylate (2e)

Yield: 45%; R_f: 0.63 (DCM:MeOH; 99:1); ^{1}H NMR (400 MHz, $CDCl_3$): δ 8.47 (s, 1H, Coumarin ring-H), 7.39 (d, 1H, Coumarin ring-H, *J*= 8.8 Hz), 6.66 (d, 1H, Coumarin ring-H, *J*= 8.8 Hz), 6.50 (s, 1H, Coumarin ring-H), 4.47 (t, 2H, -O-CH_2-CH_2-, *J*= 4.8 Hz), 3.85 (t, 2H, -O-CH_2-CH_2-, *J*= 4.9 Hz), 3.74-3.73 (m, 4H, -CH_2-CH_2-OH), 3.70-3.61 (m, 10H, 5x-O-CH_2-), 3.46 (q, 4H, 2x-CH_2-CH_3, *J*= 7.1 Hz), 1.25 (t, 6H, 2x-CH_2-CH_3, *J*= 7.0 Hz); ^{13}C NMR (100 MHz, $CDCl_3$): δ 163.8 (C=O ester), 158.3 (C=O ketone), 158.0, 152.5, 149.2, 131.1, 110.0, 108.9, 108.1, 97.3 (Coumarin ring-C), 77.2, 72.5, 70.6, 70.5, 70.3, 69.1, 64.1, 61.7 (8x-O-CH_2-), 45.4 (-CH_2-N-), 12.3 (-CH_3); ESI/MS (m/z): $[M+H]^+_{theoretical}$: 482.2; $[M+H]^+_{found}$: 482.2.

General synthetic method for the synthesis of 4a-4e

To the solution of compounds **2a-2e** (**2a**= 200 mg, 0.85 mmol; **2b**= 330 mg, 1.08 mmol; **2c**= 370 mg, 0.76 mmol; **2d**= 370 mg, 0.76 mmol; **2e**= 370 mg, 0.76 mmol) in dichloromethane (10 mL) were added succinic anhydride (**2a**= 130 mg, 1.3 mmol; **2b**= 160 mg, 1.6 mmol; **2c**= 120 mg, 1.2 mmol; **2d**=120 mg, 1.2 mmol **2e**= 120 mg, 1.2 mmol) and DMAP (**2a**= 50 mg, 0.40 mmol; **2b**= 65.97 mg, 0.49 mmol; **2c**= 46.92 mg, 0.38 mmol; **2d**= 46.92 mg, 0.38 mmol; **2e**= 46.92 mg, 0.38 mmol). The reaction mixture was stirred overnight at room temperature. The reaction mixture was diluted with 10 mL of dichloromethane and washed with 10% HCl. The organic layer was collected with solvent extraction (DCM) and washed further with brine. The obtained organic fraction was dried over anhydrous $MgSO_4$ and collected *in vacuo*. The impure organic component was purified with column chromatography (DCM: MeOH; 99:1).

4-Oxo-4-(2-(2-oxo-2*H*-chromene-3-carbonyloxy)ethoxy)butanoic acid (4a)

Yield: 67%; R_f: 0.13 (DCM:MeOH; 99:1); ^{1}H NMR (400 MHz, $CDCl_3$): δ 12.24 (s, 1H, -COOH), 8.75 (s, 1H, Coumarin ring-H), 7.93 (d, 1H, Coumarin ring-H, *J*= 7.6 Hz), 7.76 (t, 1H, Coumarin ring-H, *J*= 7.6 Hz), 7.46-7.41 (m, 2H, Coumarin ring-H), 4.46-4.44 (m, 2H, -CH_2-), 4.36-4.34 (m, 2H, -CH_2-), 2.57-2.54 (m, 2H, -CH_2-), 2.48 (m, 2H, -CH_2-); ^{13}C NMR (100 MHz, $CDCl_3$): δ 173.8 (-COOH), 172.6 (C=O ester), 162.7 (C=O ester), 156.4 (C=O ketone), 155.0, 149.5, 135.1, 130.9, 125.4, 118.2, 117.6, 116.7 (Coumarin ring-C), 63.6, 62.3 (2x-CH_2-), 29.1, 29.0 (2x-CH_2-).

4-(2-(7-(Diethylamino)-2-oxo-2*H*-chromene-3-carbonyloxy)ethoxy)-4-oxobutanoic acid (4b)

For physical properties and spectroscopic data of compound **4b** see Jinyui Xu *et. al.*[2]

4-(2-(2-(2-(7-(Diethylamino)-2-oxo-2*H*-chromene-3-carbonyloxy)ethoxy)ethoxy)ethoxy)-4-oxobutanoic acid (4c)

For physical properties and spectroscopic data of compound **4c** see Jinyui Xu *et. al.*[2]

4-(2-(2-(2-(2-(7-(Diethylamino)-2-oxo-2*H*-chromene-3-carbonyloxy)ethoxy)ethoxy)ethoxy)ethoxy)-4-oxobutanoic acid (4d)

Yield: 57%; R_f: 0.23 (DCM:MeOH; 99:1); ^{1}H NMR (400 MHz, $CDCl_3$): δ 8.47 (s, 1H, Coumarin ring-H), 7.39 (d, 1H, Coumarin ring-H, *J*= 8.8 Hz), 6.65 (dd, 1H, Coumarin ring-H, *J*= 2.0, 8.8 Hz), 6.49 (d, 1H, Coumarin ring-H, *J*= 2.0 Hz), 4.47 (t, 2H, -O-CH_2-CH_2-, *J*= 4.8 Hz), 4.27 (t, 2H, -O-CH_2-CH_2-, *J*= 4.6 Hz), 3.85 (t, 2H, -O-CH_2-CH_2-, *J*= 4.8 Hz), 3.78-3.76 (m, 2H, -OCH_2-), 3.72-3.65 (m, 8H, 4x-O-CH_2-), 3.47 (q, 4H, 2x-CH_2-, *J*= 7.1 Hz), 2.66 (s, 4H, 2x-CH_2-), 1.25 (t, 6H, 2x-CH_3, *J*= 7.1 Hz); ^{13}C NMR (100 MHz, $CDCl_3$): δ 173.8 (-COOH), 172.7, 163.7 (2xC=O ester), 158.6 (C=O ketone), 157.4, 153.4, 149.7, 132.3, 110.3, 107.6, 107.4, 96.3 (Coumarin ring-C), 70.3, 70.2, 70.2, 68.8, 68.7, 64.1, 63.8 (7x-O-CH_2-), 44.8 (-CH_2-N-), 29.3, 29.2 (2x-CH_2-), 12.8 (-CH_3); ESI/MS (m/z): $[M+H]^+_{theoretical}$: 538.3; $[M+H]^+_{found}$: 538.3.

4-(2-(2-(2-(2-(2-(7-(Diethylamino)-2-oxo-2*H*-chromene-3-carbonyloxy)ethoxy)ethoxy)ethoxy)ethoxy)ethoxy)-4-oxobutanoic acid (4e)

Yield: 51.35%; R_f: 0.22 (DCM:MeOH; 99:1); ^{1}H NMR (400 MHz, $CDCl_3$): δ 8.47 (s, 1H, Coumarin ring-H), 7.28 (s, 1H, Coumarin ring-H), 6.65 (dd, 1H, Coumarin ring-H, *J*= 2.0, 8.8 Hz), 6.49 (d, 1H, Coumarin ring-H, *J*= 1.6 Hz), 4.47 (t, 2H, -O-CH_2-CH_2-, *J*= 4.7 Hz), 4.27 (t, 2H, -O-CH_2-CH_2-, *J*= 4.4 Hz), 3.84 (t, 2H, -O-CH_2-CH_2-, *J*= 4.9 Hz), 3.75-3.64 (m, 14H, 7x-O-CH_2-), 3.47 (q, 4H, 2x-CH_2-, *J*= 7.1 Hz), 2.70-2.63 (m, 4H, 2x-CH_2-), 1.25 (t, 6H, 2x-CH_3, *J*= 7.1 Hz); ^{13}C NMR (100 MHz, $CDCl_3$): δ 174.5 (-COOH), 171.9, 163.9 (2xC=O ester), 158.4 (C=O ketone), 158.2, 152.7, 149.4, 131.1, 109.8, 108.7, 107.9, 97.0 (Coumarin ring-C), 70.7, 70.5, 70.5, 70.4, 70.4, 69.1, 68.9, 64.1, 63.8 (9x-O-CH_2-), 45.3 (-CH_2-N-), 29.6, 29.0 (2x-CH_2-), 12.3 (-CH_3); ESI/MS (m/z): $[M+H]^+_{theoretical}$: 582.2; $[M+H]^+_{found}$: 582.2.

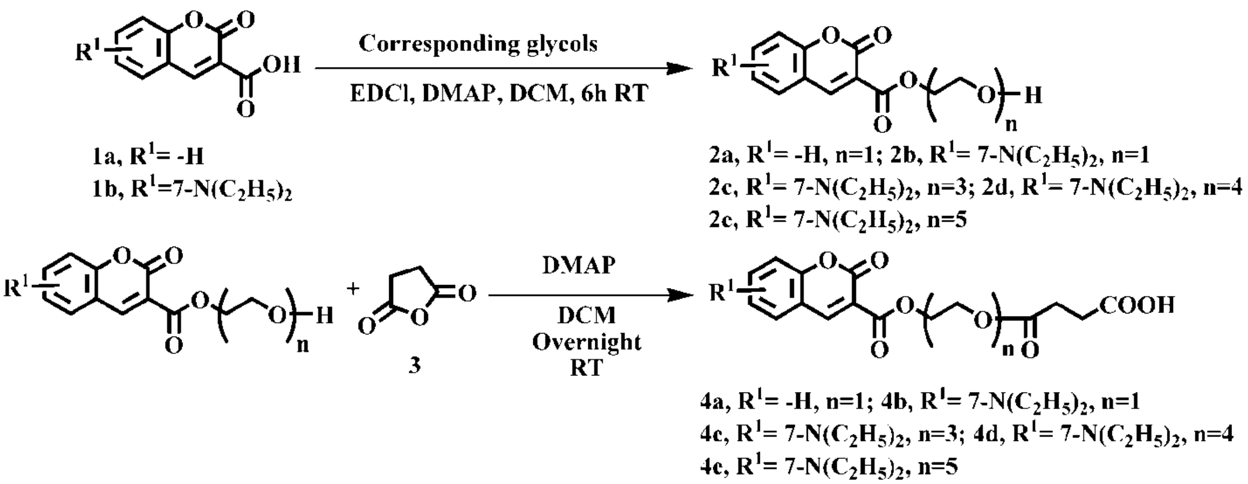

Scheme S2: General representation for the synthesis of **2a-2e** and **4a-4e**

General synthetic method for the synthesis of 7a-7d

In an oven dried round bottom flask, respective amine and maleic anhydride (for amount and duration of reaction see individual data) were added with 20 mL of ethyl acetate. After stirring at room temperature, solid starts precipitating. The precipitated solid was collected as pure with filtration under reduced pressure.

Synthesis of 4-(hexylamino)-4-oxobut-2-enoic acid (7a)

n-Hexyl amine (1 mL, 7.56 mmol); maleic anhydride (900 mg, 9.18 mmol); Duration: 17 h; Yield: 73.10%; R_f: 0.34 (DCM:MeOH; 98:2); ^{1}H NMR (400 MHz, DMSO-d6): δ 15.20 (s, 1H, -COOH), 9.11 (s, 1H, -NH), 6.40 (d, 1H, -CH=CH-, *J*= 12.5 Hz), 6.24 (d, 1H, -CH=CH-, *J*= 12.5 Hz), 3.17 (q, 2H, -NH-CH_2-CH_2-, *J*= 6.56 Hz), 1.47-1.42 (m, 2H, -CH_2-), 1.28 (s, 6H, -CH_2-CH_2-CH_2-), 0.86 (t, 3H, -CH_3, *J*= 6.44 Hz); ^{13}C NMR (100 MHz, DMSO-d6): δ 165.8 (-COOH), 165.7 (-CO-NH-), 133.5, 132.2 (-CH=CH-), 39.6, 31.3, 28.7, 26.5, 22.5, 14.4 (hexyl chain carbons).

Synthesis of 4-(2-(*tert*-butoxycarbonyl)ethylamino)-4-oxobut-2-enoic acid (7b)

For physical properties and spectroscopic data of compound **7b** see Eken *et.al.*[3]

Synthesis of 4-(4-methyl-2-oxo-2*H*-chromen-7-ylamino)-4-oxobut-2-enoic acid (7c)

For physical properties and spectroscopic data of compound **7c** see Reddy, P. Y.[4]

Synthesis of 4-oxo-4-[(adamantylmethyl)amino]but-2-enoic acid (7d)

1-Adamantanemethylamine (0.2 mL, 1.12 mmol); maleic anhydride (121.8 mg, 1.24 mmol); Duration: 30 minute; Yield: 58.82%; R_f: 0.41 (DCM:MeOH; 95:5); ^{1}H NMR (400 MHz, DMSO-d6): δ 15.25 (s, 1H, -COOH), 8.98 (t, 1H, -NH-CH_2-), 6.52 (d, 1H, -CH=CH-, *J*= 12.8 Hz), 6.26 (d, 1H, -CH=CH-, *J*= 12.4 Hz), 2.91 (d, 2H, -CH_2-NH-, *J*= 6.4 Hz), 1.94 (s, 3H, adamantyl-H), 1.68-1.58 (m, 6H, adamantyl-H), 1.47 (m, 6H, adamantyl-H); ^{13}C NMR (100 MHz, DMSO-d6): δ 166.3 (-COOH), 165.8 (-C=O), 133.6, 132.3 (-CH=CH-), 51.2 (-CH_2-), 40.1, 36.8, 34.3, 28.1 (adamantyl-C); ESI/MS (m/z): $[M+H]^+_{theoretical}$:

264.1, $[M+H]^+_{found}$: 264.1; $[M+Na]^+_{theoretical}$: 286.1, $[M+Na]^+_{found}$: 286.1; $[2M+Na]^+_{theoretical}$: 549.3, $[2M+Na]^+_{found}$: 549.3.

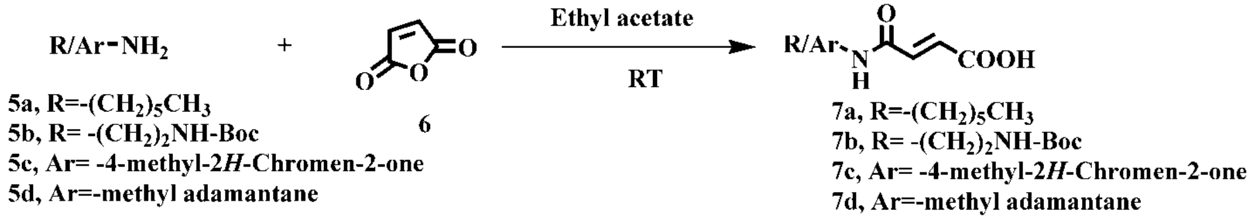

Scheme S3: Synthetic representation of compounds **7a-7d**

Synthesis of 12-hydroxydodecyl 7-(diethylamino)-2-oxo-2*H*-chromene-3-carboxylate (9)

In a solution of compound **1b** (500 mg, 1.63 mmol) in DCM (15 mL) were added 1,12-dodecandiol (1520 mg, 7.51 mmol), EDCl (310 mg, 1.64 mmol) and DMAP (100 mg, 0.81 mmol). The reaction mixture was stirred 6 h at room temperature. The progress of the reaction mixture was monitored with TLC plate after every 30 minutes. Once reaction reached to their maximum completion, the reaction mixture was further diluted with 10 mL of DCM. 10% HCl solution was added to wash the organic layer. The organic layer was separated with solvent extraction (DCM). The obtained organic layer was further washed with brine and collected again with solvent extraction (DCM). The oily impure residue was obtained after evaporation *in vacuo* and purified with column chromatography (DCM: MeOH 99:1).

Yield: 66.00%; R_f: 0.57 (DCM:MeOH; 99:1); ^{1}H NMR (400 MHz, $CDCl_3$): δ 8.33 (s, 1H, Coumarin ring-H), 7.29 (d, 1H, Coumarin ring-H, *J*= 8.8 Hz), 6.57 (dd, 1H, Coumarin ring-H, *J*= 2.0, 8.8 Hz), 6.42 (d, 1H, Coumarin ring-H, *J*= 1.9 Hz), 4.22 (t, 2H, -O-CH_2-CH_2-, *J*= 6.8 Hz), 3.56 (t, 2H, -O-CH_2-CH_2-, *J*= 6.6 Hz), 3.37 (q, 4H, 2x-CH_2-, *J*= 7.1 Hz), 1.68 (quintet, 2H, -CH_2-CH_2-CH_2-, *J*= 6.8 Hz), 1.49 (quintet, 2H, -CH_2-CH_2-CH_2-, *J*= 6.6 Hz), 1.35-1.20 (m, 16H, 8x-CH_2-), 1.16 (t, 6H, 2x-CH_3, *J*= 7.1 Hz); ^{13}C NMR (100 MHz, $CDCl_3$): δ 164.2 (C=O ester), 158.3 (C=O ketone), 158.0, 152.2, 148.9, 131.0, 110.0, 109.7, 108.3, 97.4 (Coumarin ring-C), 65.4, 63.1 (2x-O-CH_2-), 45.5 (-CH_2-N-), 32.8, 29.5, 29.5, 29.4, 29.4, 29.2, 28.7, 25.9, 25.7 (9x-CH_2-), 12.3 (-CH_3); ESI/MS (m/z): $[M+H]^+_{theoretical}$: 446.2; $[M+H]^+_{found}$: 446.2; $[M+Na]^+_{theoretical}$: 468.2; $[M+Na]^+_{found}$: 468.2; $[2M+Na]^+_{theoretical}$: 913.5; $[2M+Na]^+_{found}$: 913.4.

Synthesis of 12-hydroxydodecyl 7-(diethylamino)-2-oxo-2*H*-chromene-3-carboxylate (10)

In an oven dried round bottom flask, compound **9** (160 mg, 0.35 mmol) and succinic anhydride (50 mg, 0.5 mmol) and DMAP (20 mg, 0.16 mmol) were added in 10 mL of DCM. The reaction mixture was stirred over night at room temperature. Then reaction mixture was washed with 10% HCl and excess of brine. The organic fraction was collected with solvent extraction (DCM) and dried over anhydrous $MgSO_4$. The collected impure oily residue was purified with manual column chromatography (DCM: MeOH 99:1).

Yield: 65.18%; R_f: 0.39 (DCM:MeOH; 99:1); ^{1}H NMR (400 MHz, $CDCl_3$): δ 8.34 (s, 1H, Coumarin ring-H), 7.29 (d, 1H, Coumarin ring-H, *J*= 8.8 Hz), 6.57 (dd, 1H, Coumarin ring-H, *J*= 1.8, 8.9 Hz), 6.42 (d, 1H, Coumarin ring-H, *J*= 1.6 Hz), 4.22 (t, 2H, -O-CH_2-CH_2-, *J*= 6.8 Hz), 4.01 (t, 2H, -O-CH_2-CH_2-, *J*= 6.6 Hz), 3.37 (q, 4H, 2x-CH_2-, *J*= 7.1 Hz), 2.63-2.59 (m, 2H. -CH_2-), 2.57-2.54 (m, 2H, -CH_2-), 1.68 (quintet, 2H, -CH_2-CH_2-CH_2-, *J*= 6.8 Hz), 1.54 (quintet, 2H, -CH_2-CH_2-CH_2-, *J*= 6.4 Hz), 1.35-1.20 (m,

16H, 8x-CH_2-), 1.16 (t, 6H, 2x-CH_3, J= 7.1 Hz); ^{13}C NMR (100 MHz, $CDCl_3$): δ 176.3 (-COOH), 172.2 (C=O), 164.3 (C=O ester), 158.3 (C=O ketone), 158.2, 149.0, 131.0, 109.9, 108.1, 97.2 (Coumarin ring-C), 65.4, 65.0 (2x-O-CH_2-), 45.3 (-CH_2-N-), 29.4, 29.4, 29.4, 29.2, 29.1, 28.9, 28.7, 28.6, 28.5, 25.9, 25.8 (11x-CH_2-), 12.3 (-CH_3); ESI/MS (m/z): $[M+H]^+_{theoretical}$: 546.2; $[M+H]^+_{found}$: 546.2; $[M+Na]^+_{theoretical}$: 568.2; $[M+Na]^+_{found}$: 568.2.

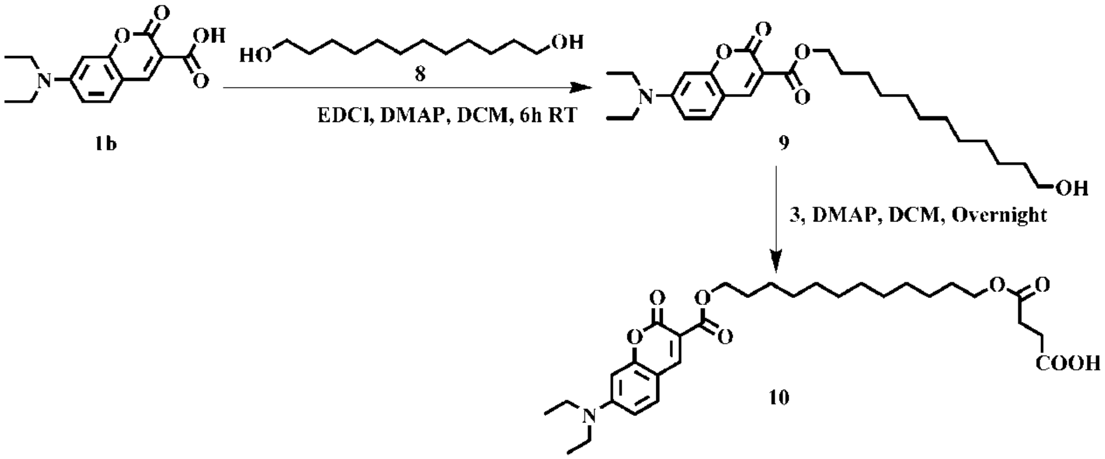

Scheme S4: Synthetic route for compound **9** and **10**

Figure S8: ^{1}H NMR of **2d**.

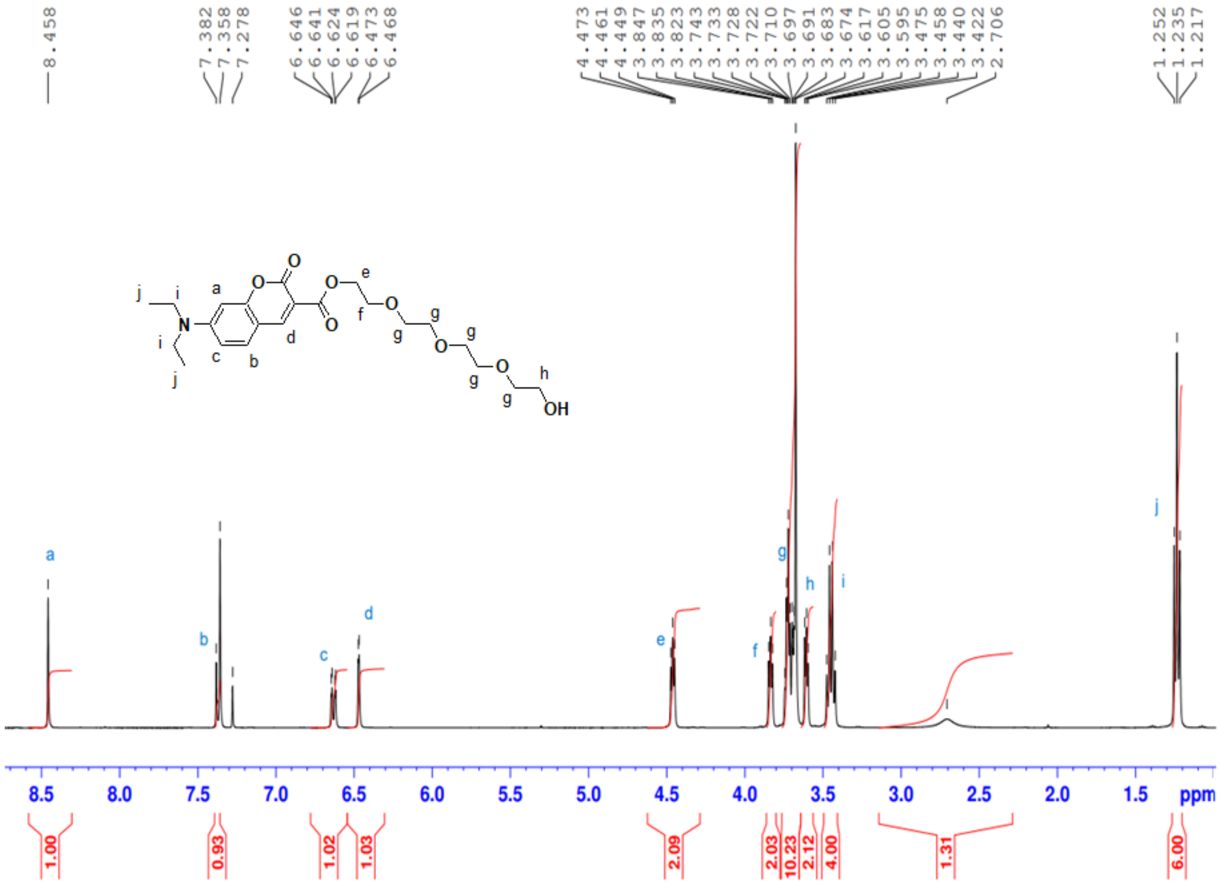

Figure S9: ^{13}C NMR of **2d**.

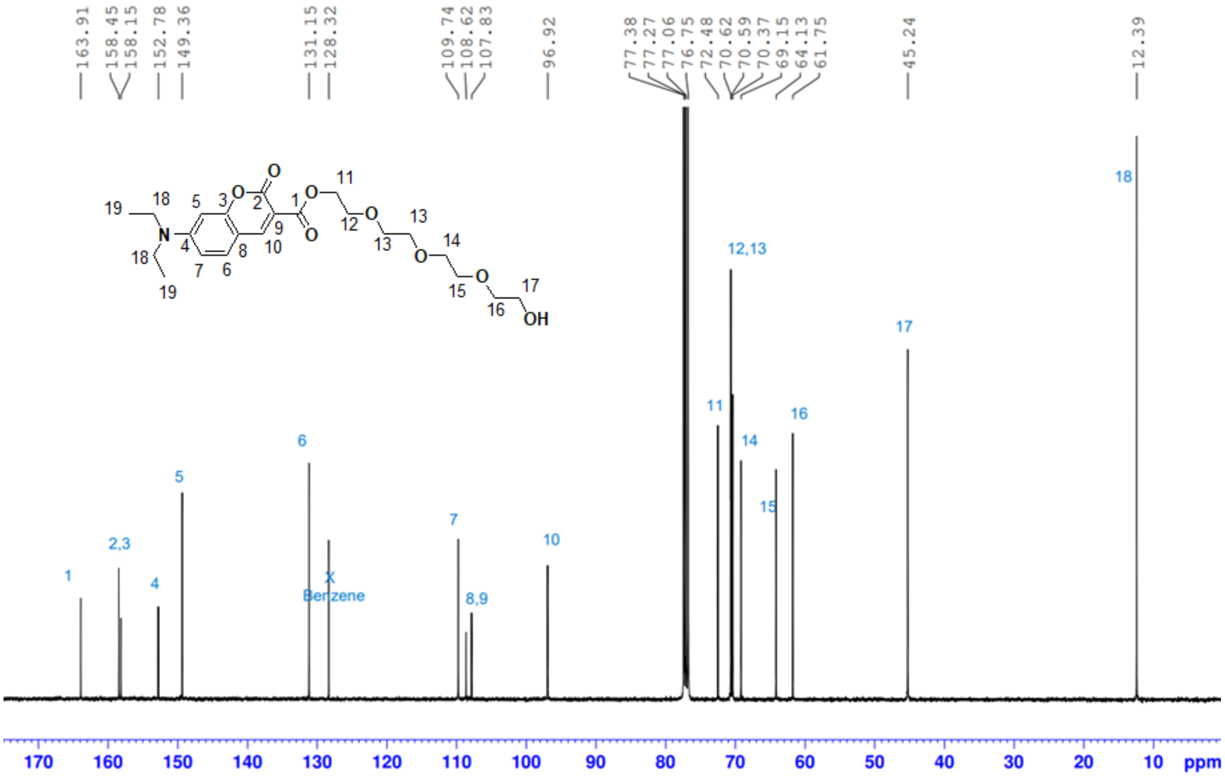

Figure S10: ESI/MS of **2d.**

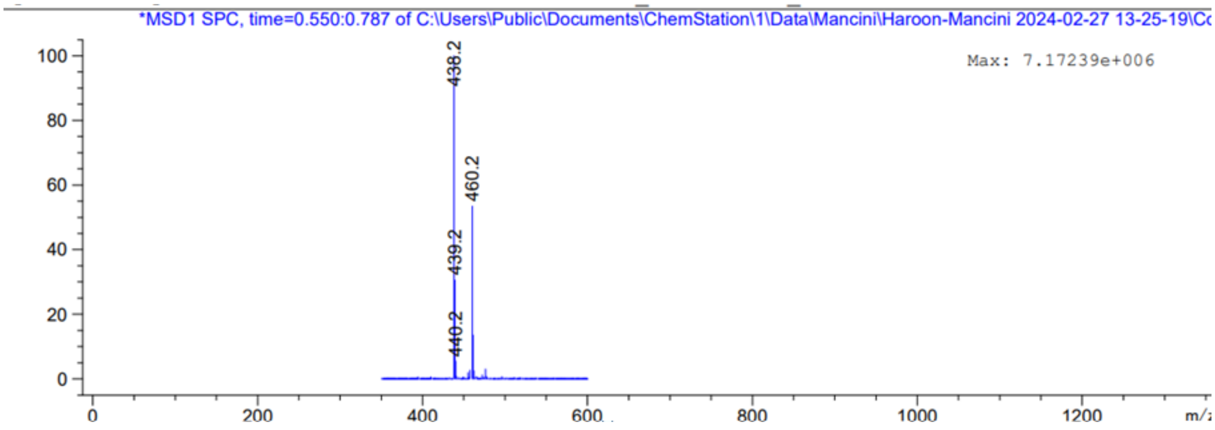

Figure S11: ^{1}H NMR of **2e**.

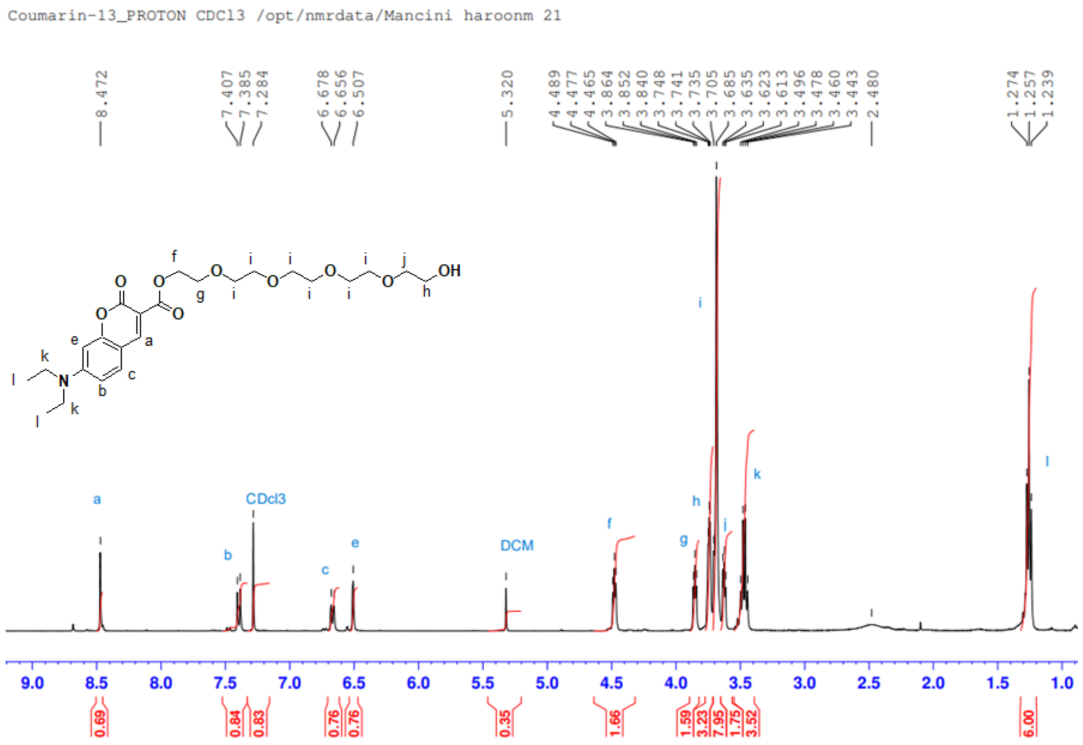

Figure S12: ^{13}C NMR of **2e**.

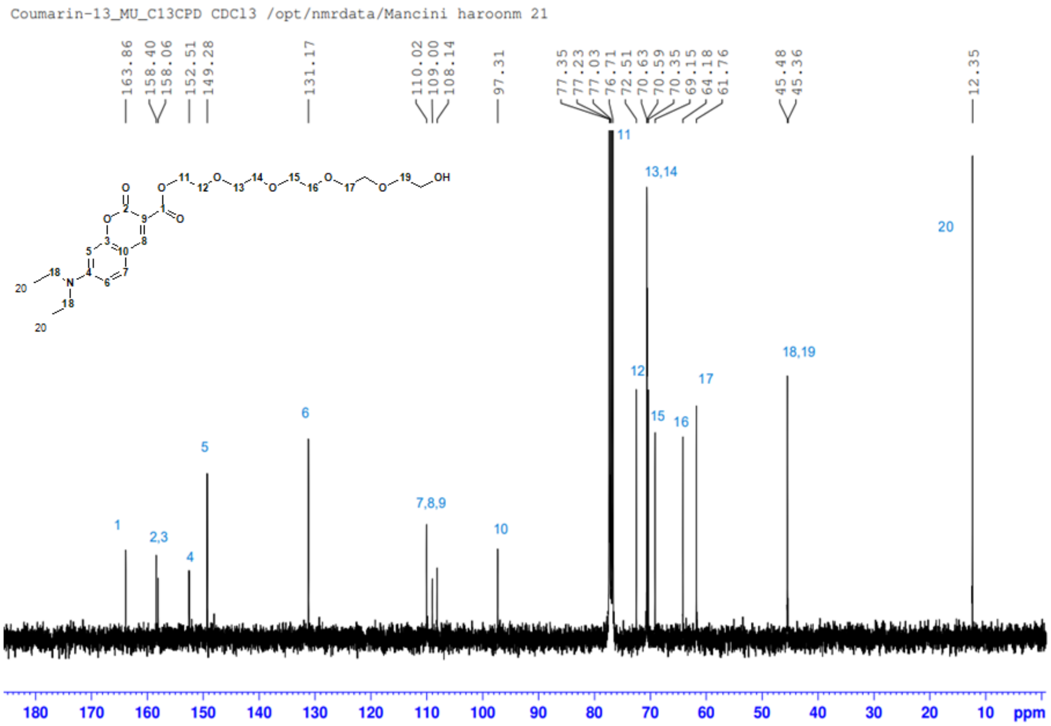

Figure S13: ESI/MS of **2e.**

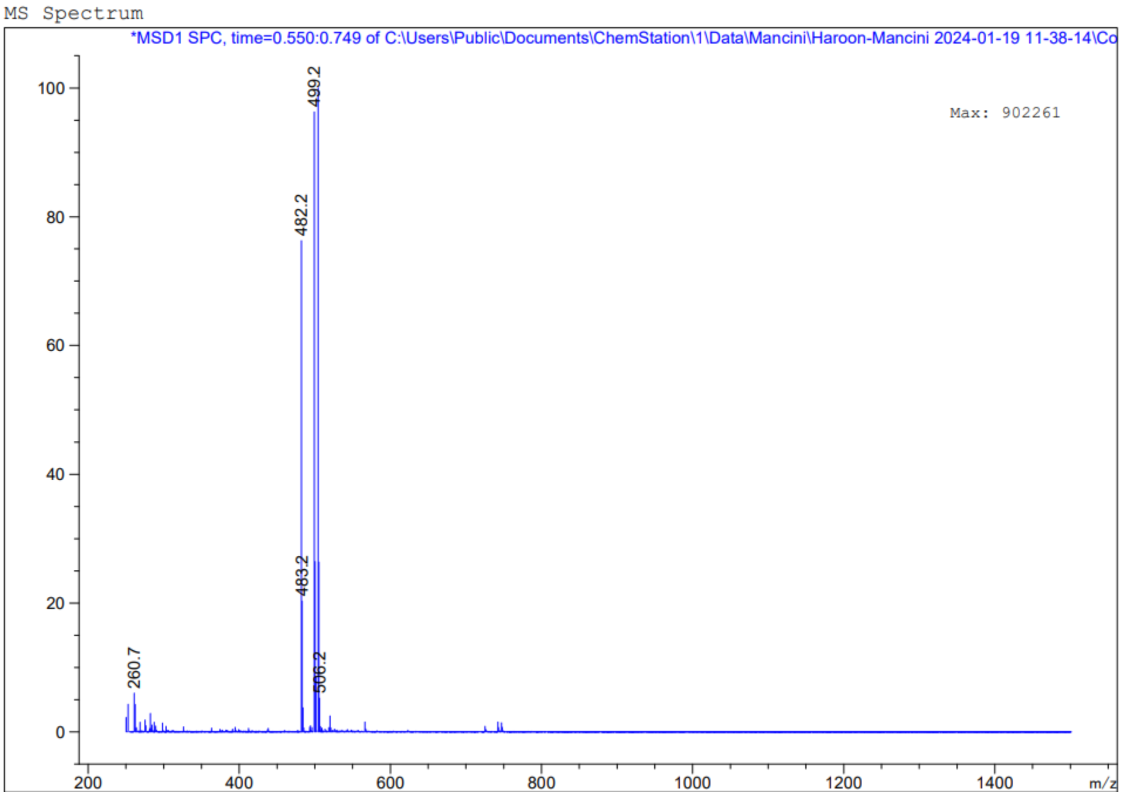

Figure S14: ^{1}H NMR of **4a.**

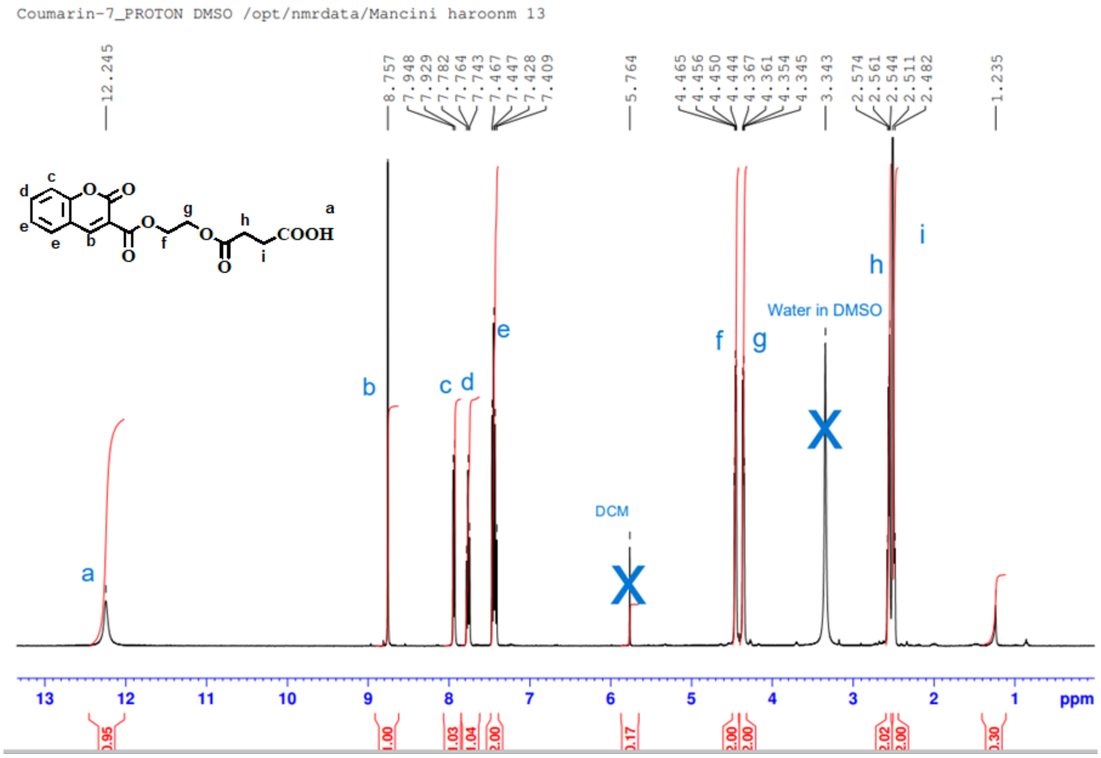

Figure S15: ^{13}C NMR of **4a.**

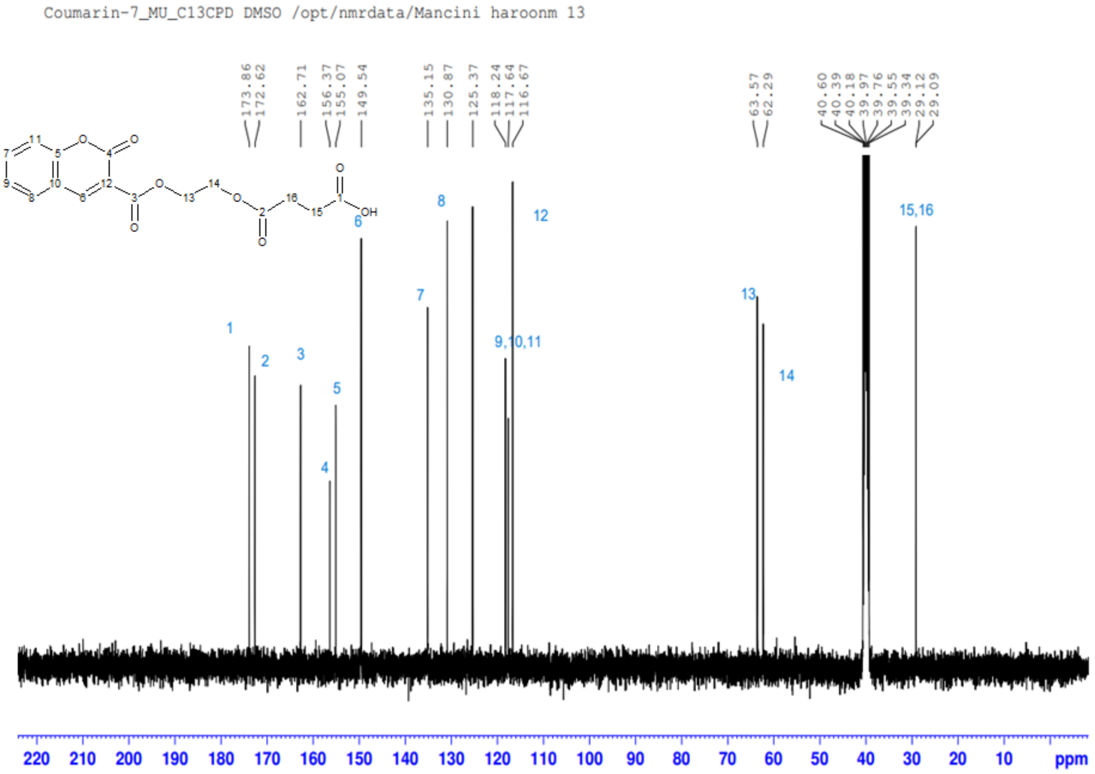

Figure S16: ^{1}H NMR of **4d.**

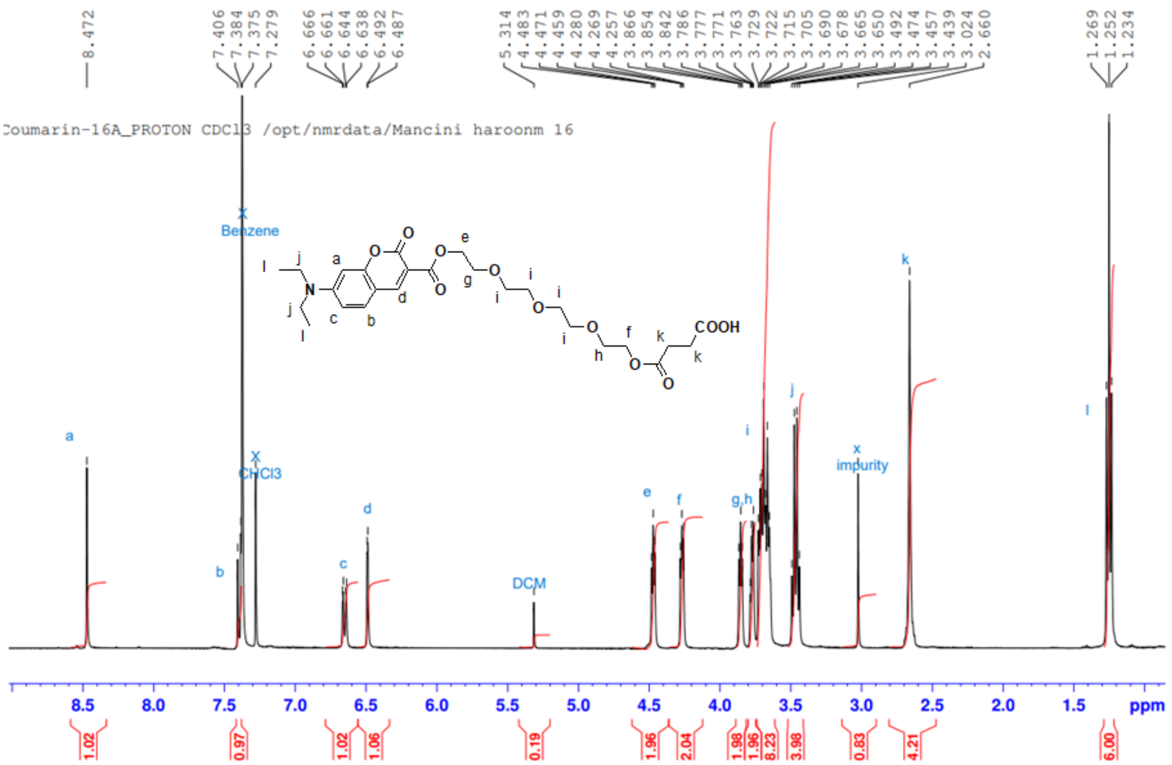

Figure S17: ^{13}C NMR of **4d.**

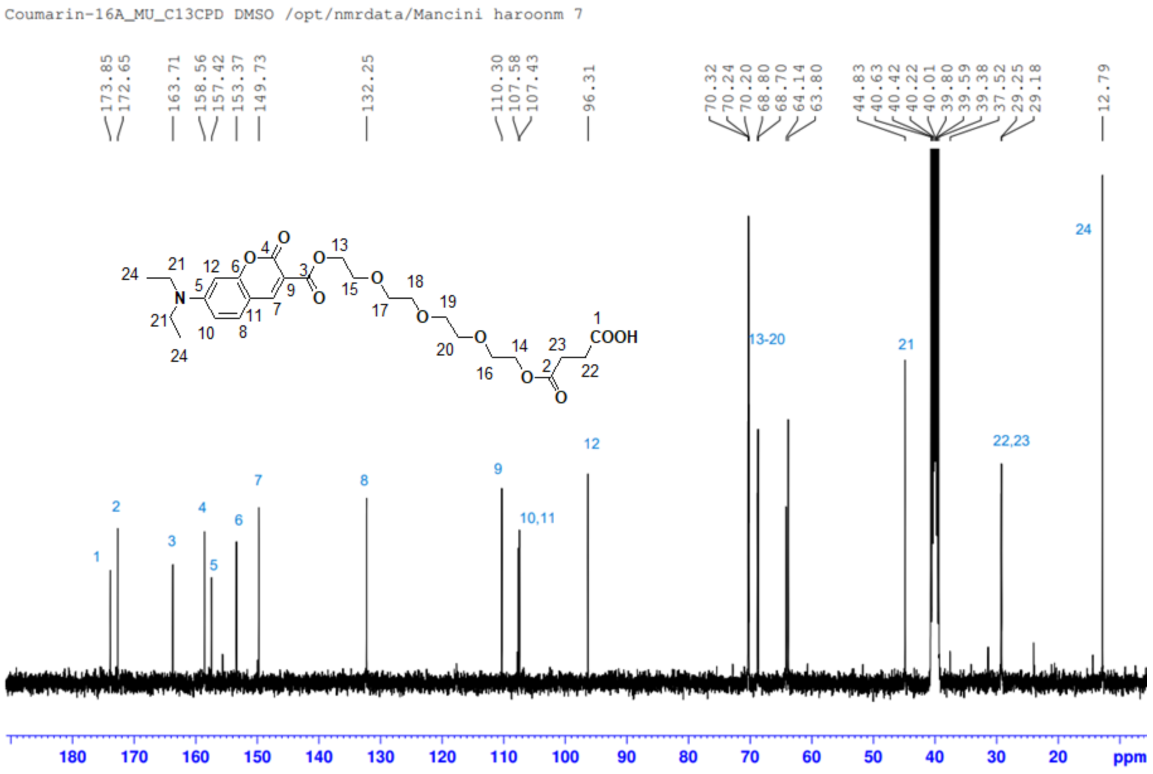

Figure S18: ESI/MS of **4d.**

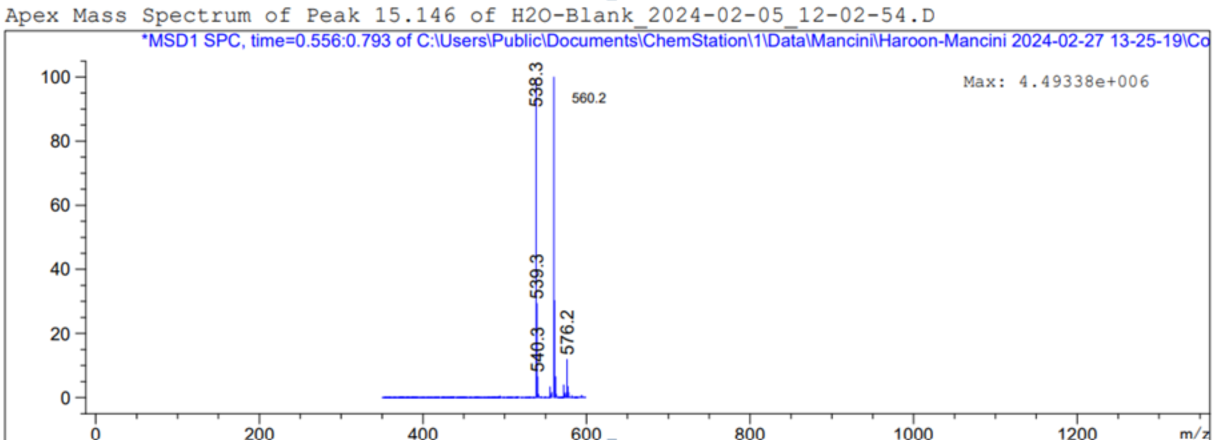

Figure S19: ^{1}H NMR of **4e.**

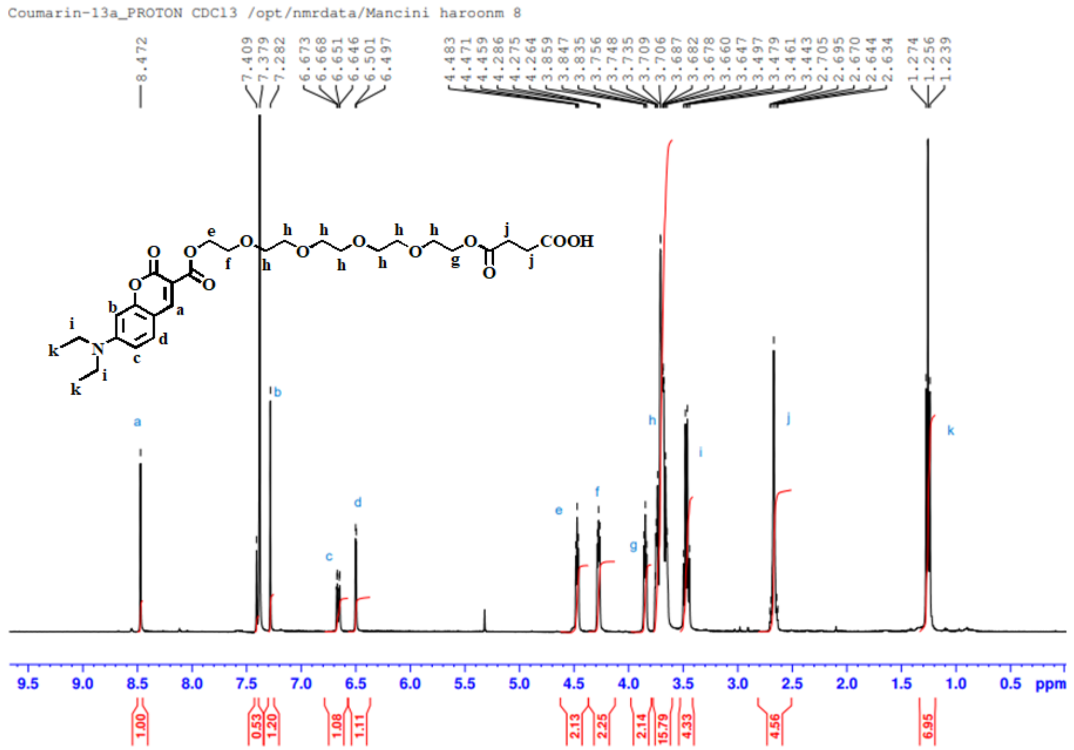

Figure S20: ^{13}C NMR of **4e**.

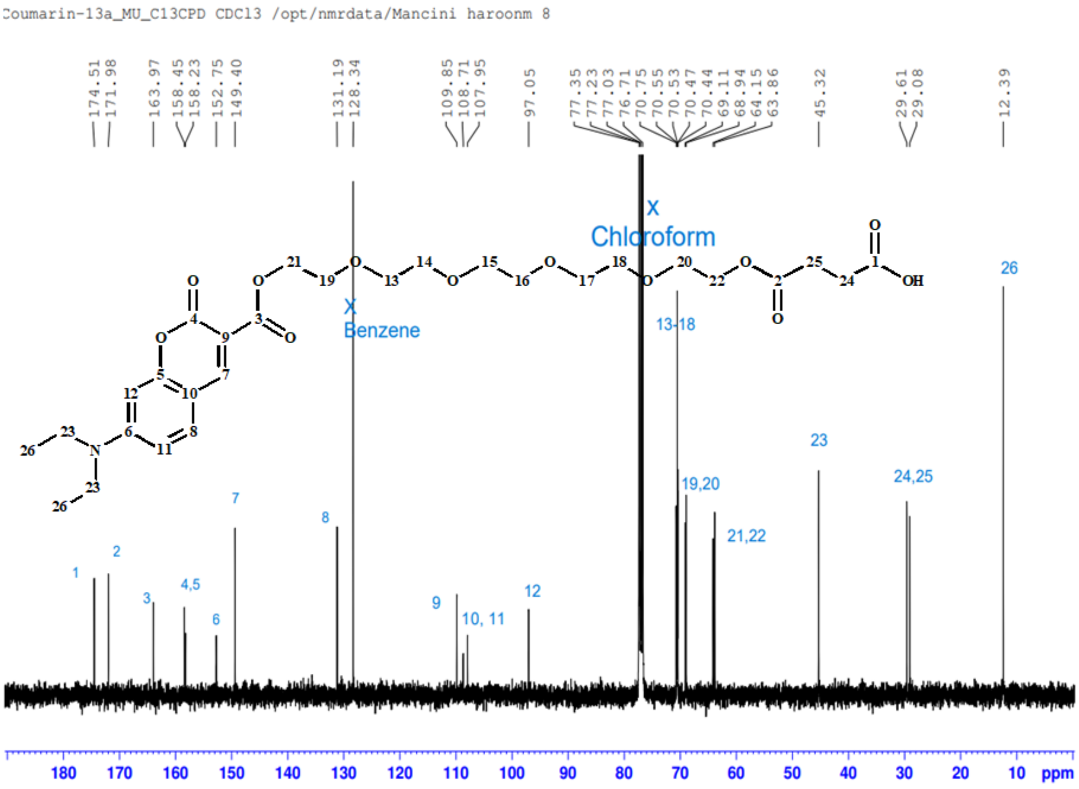

Figure S21: ESI/MS of **4e**.

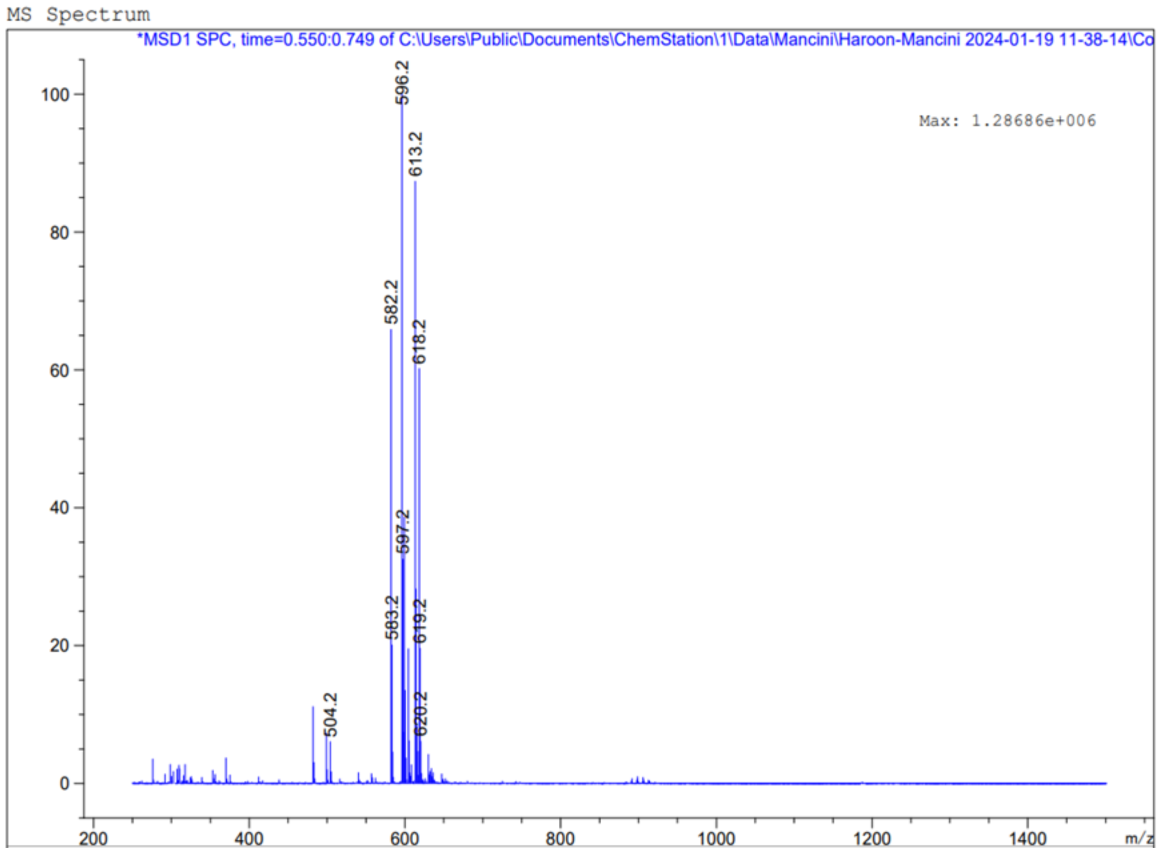

Figure S22: ^{1}H NMR of **7a**.

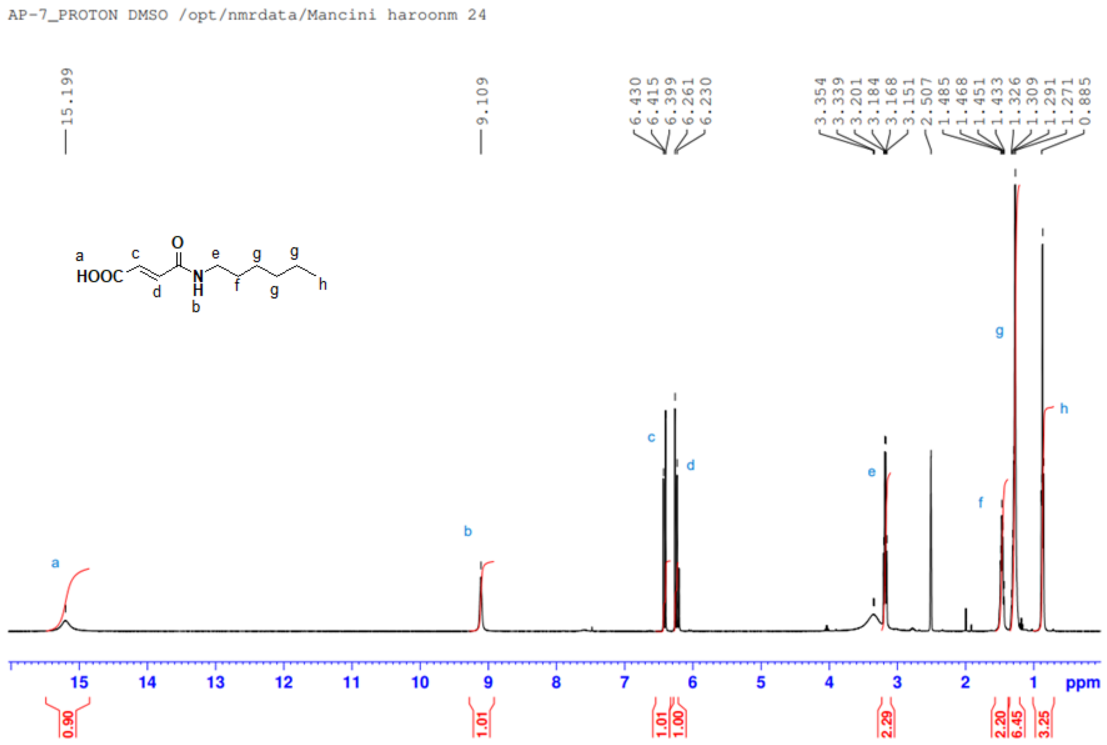

Figure S23: ^{13}C NMR of **7a**.

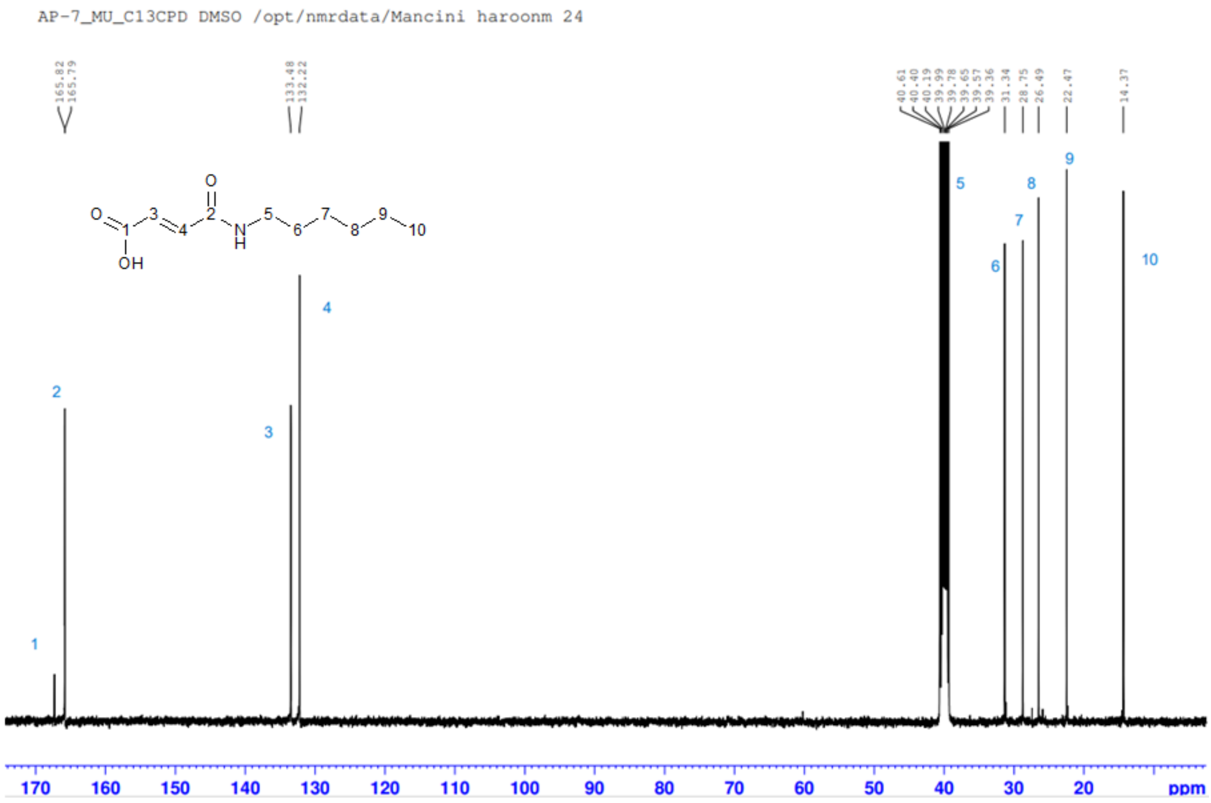

Figure S24: ^{1}H NMR of **7d.**

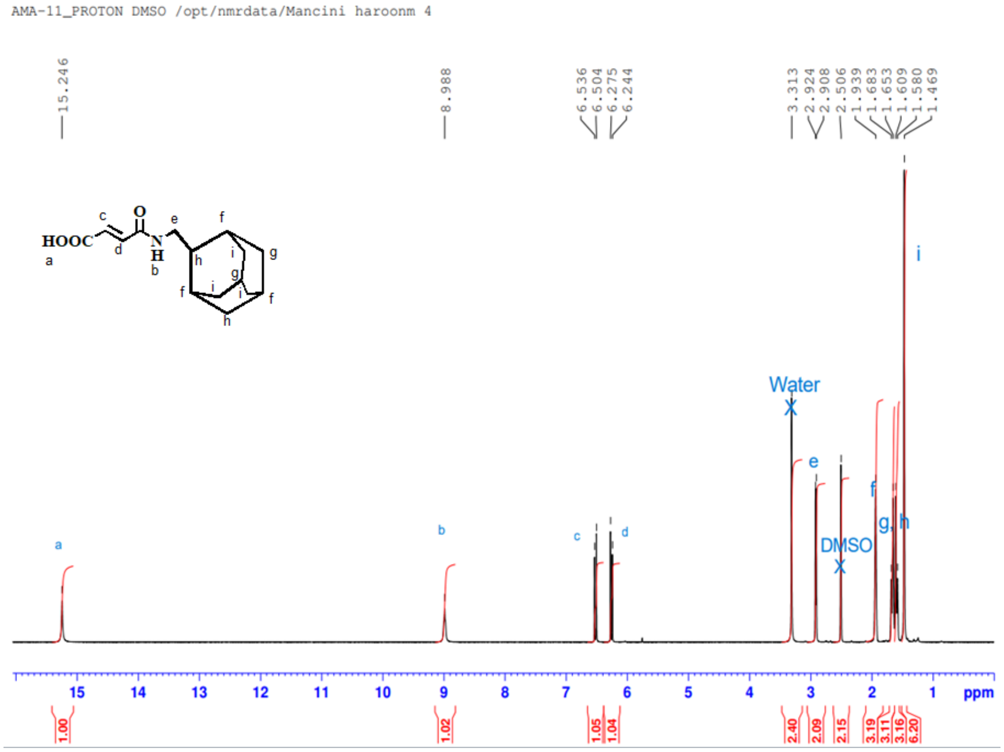

Figure S25:^{13}C NMR of **7d.**

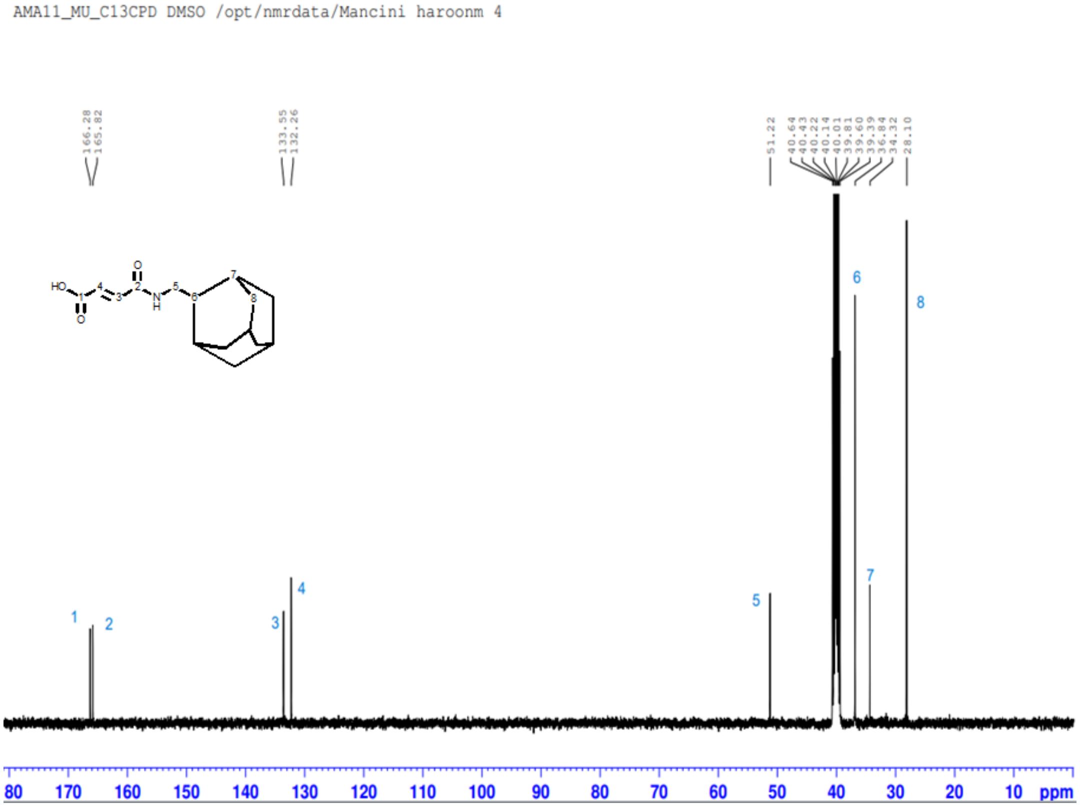

Figure S26: ^{1}H NMR of **9.**

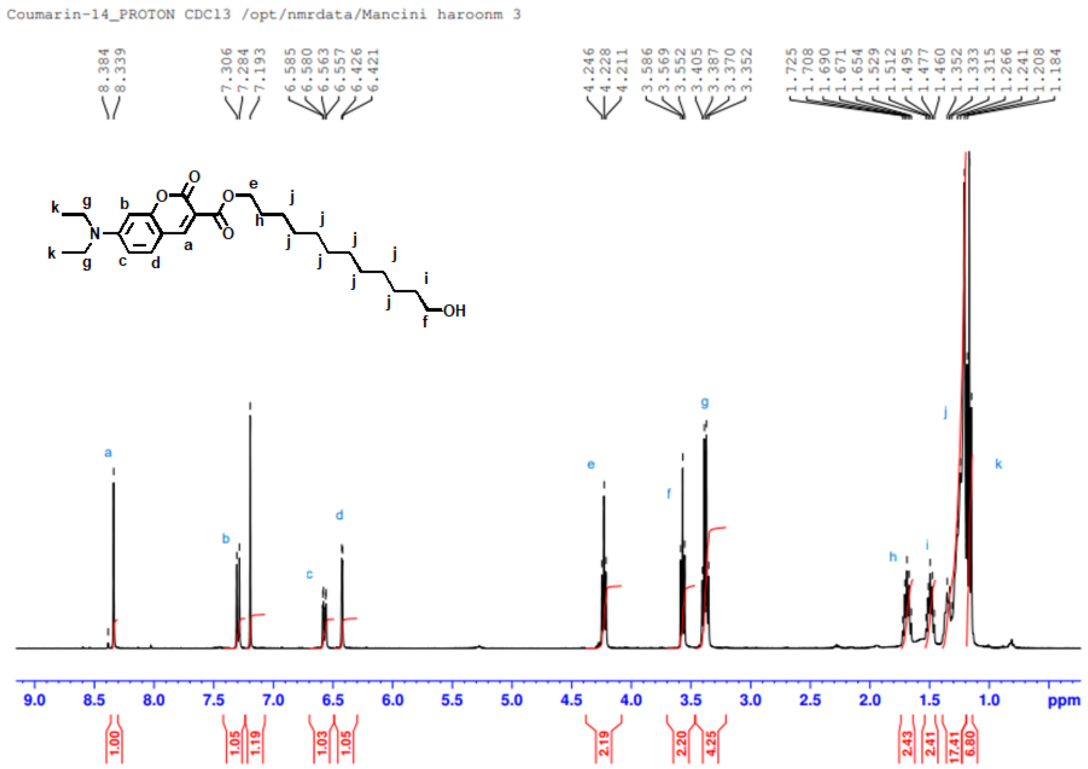

Figure S27: ^{13}C NMR of **9.**

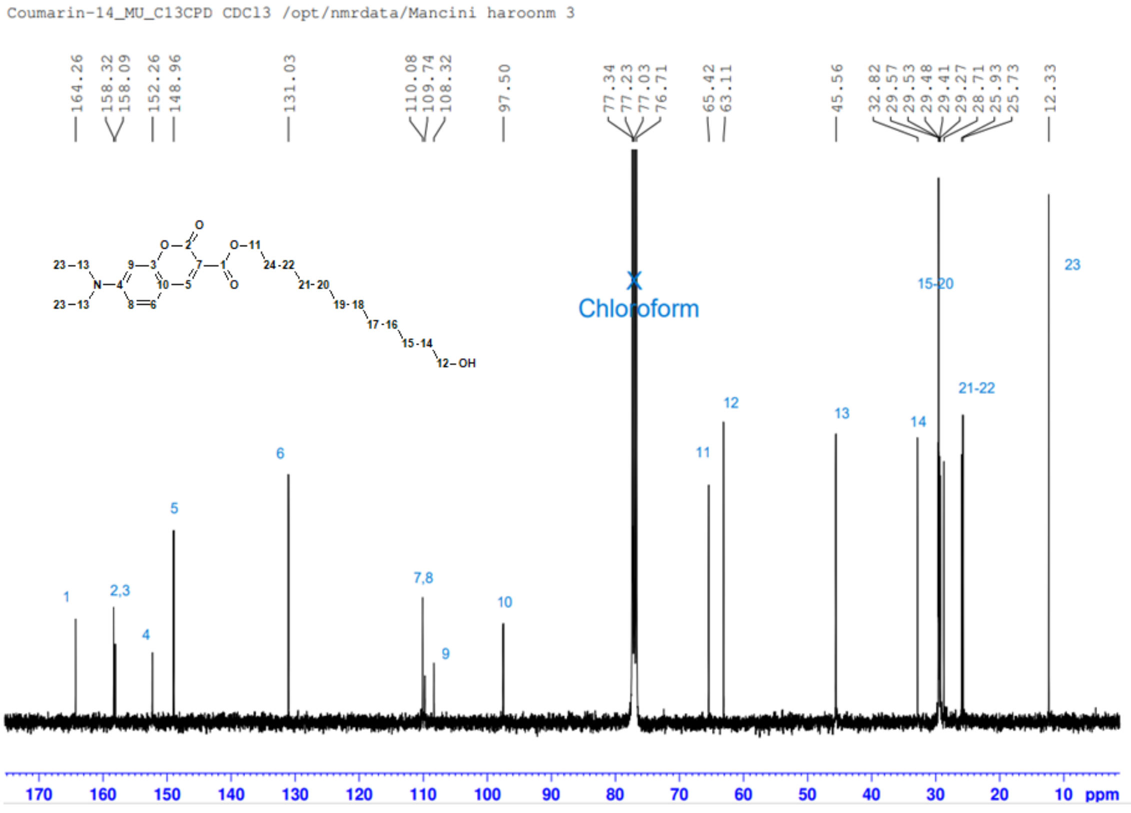

Figure S28: ESI/MS of **9.**

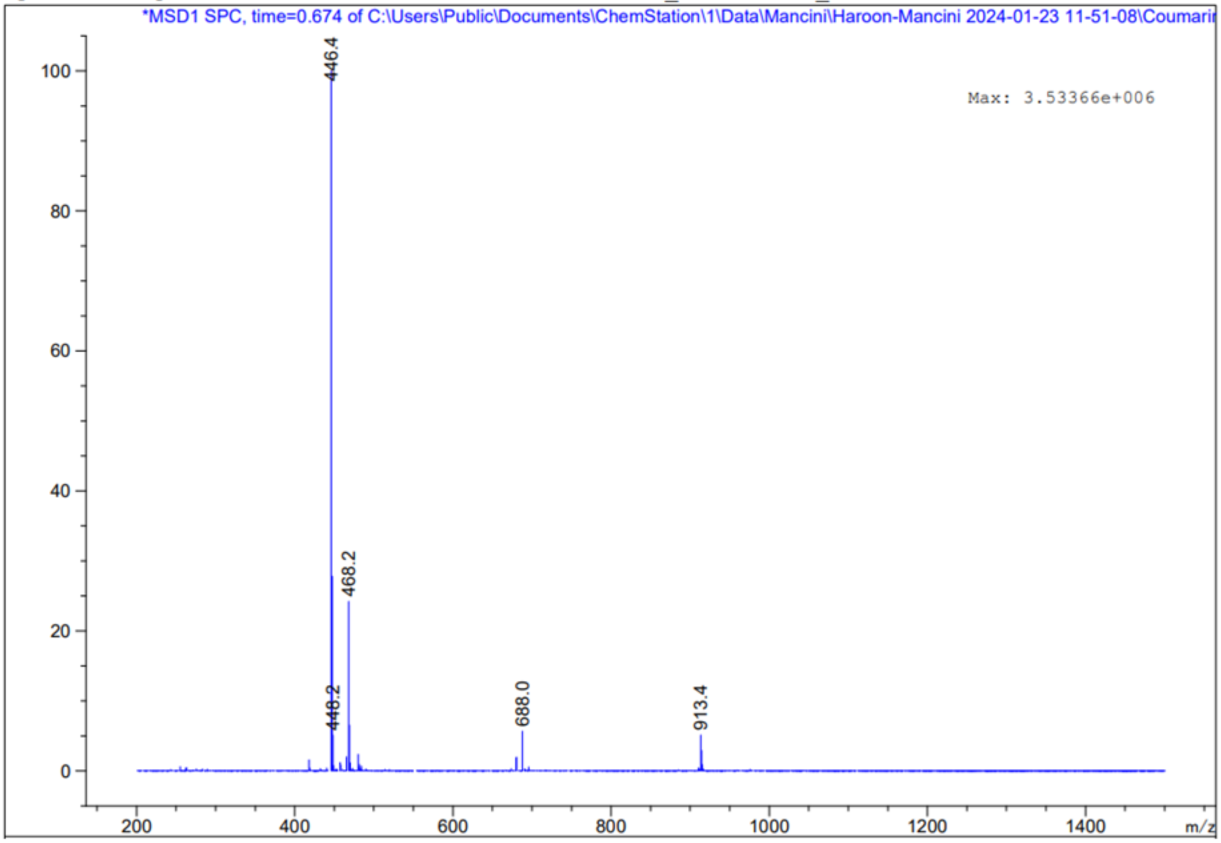

Figure S29: ^{1}H NMR of **10.**

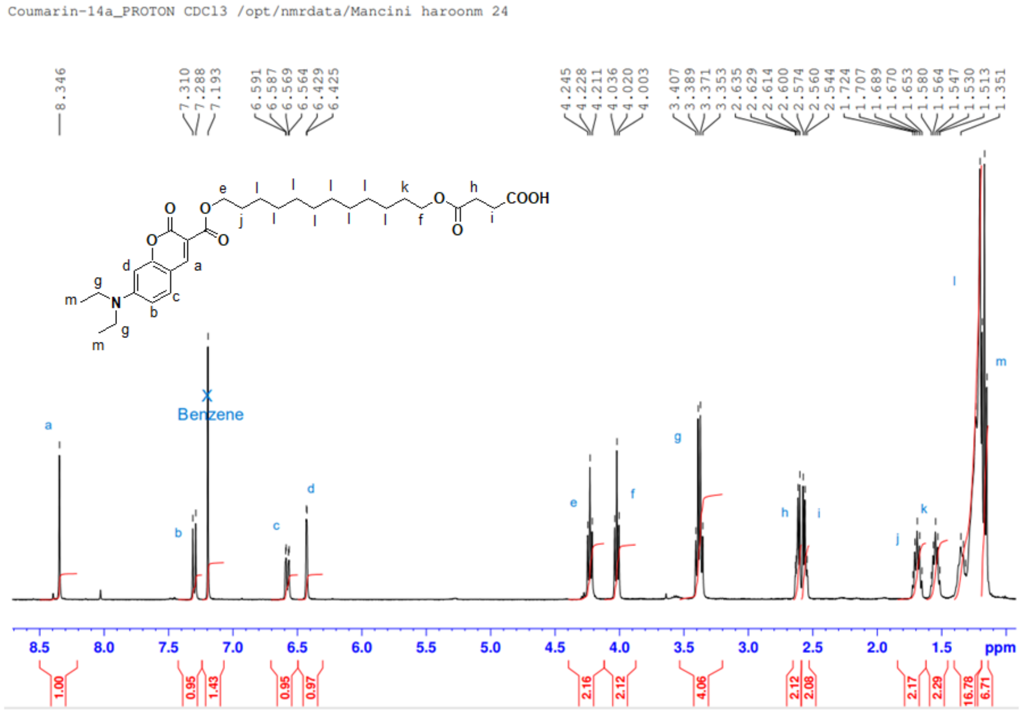

Figure S30: ^{13}C NMR of **10.**

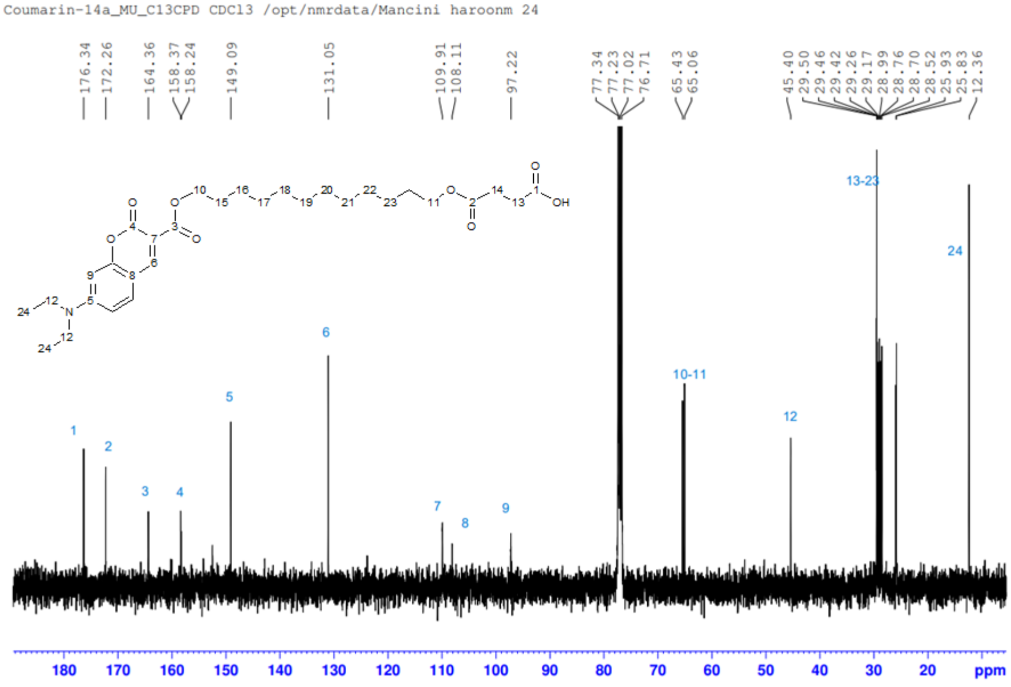

Figure S31: ESI/MS of **10.**

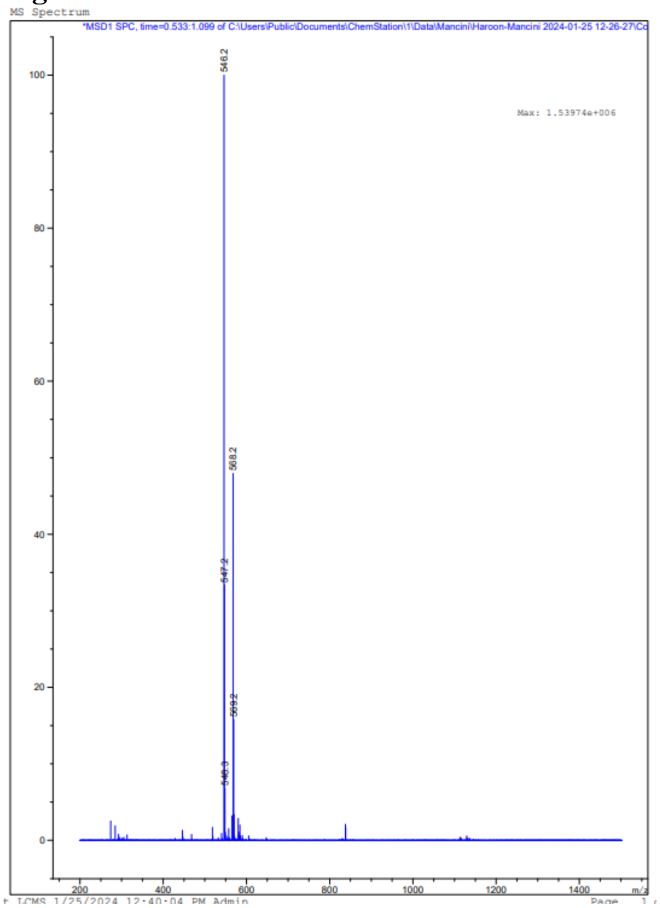

Figure S32: ^{1}H NMR of **A1.**

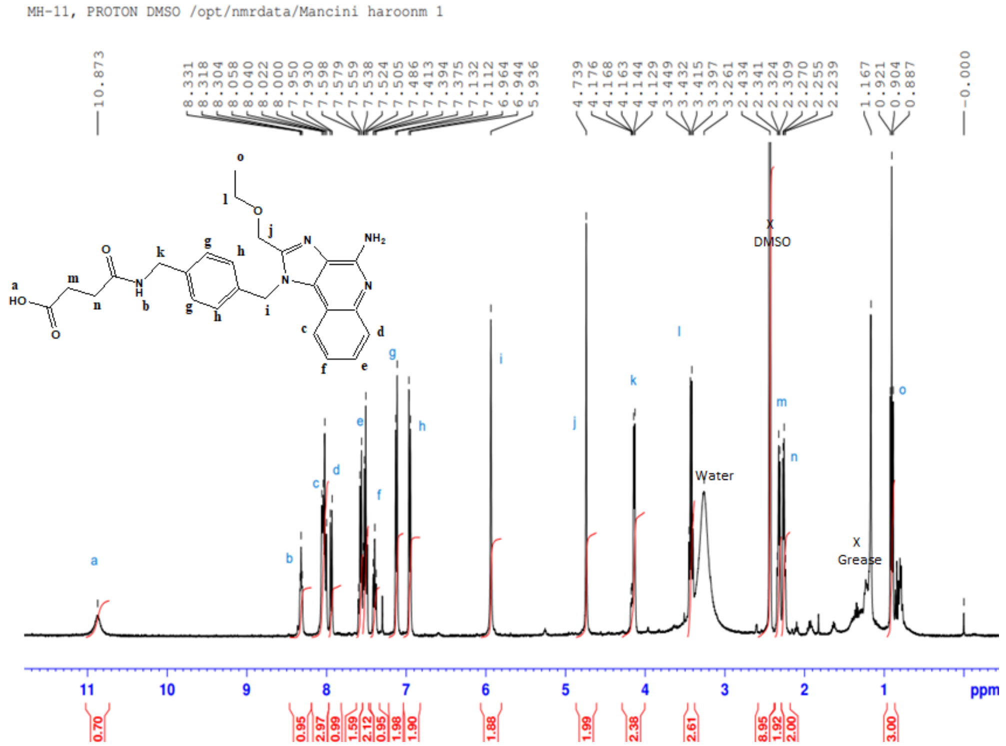

Figure S33: ^{13}C NMR of **A1.**

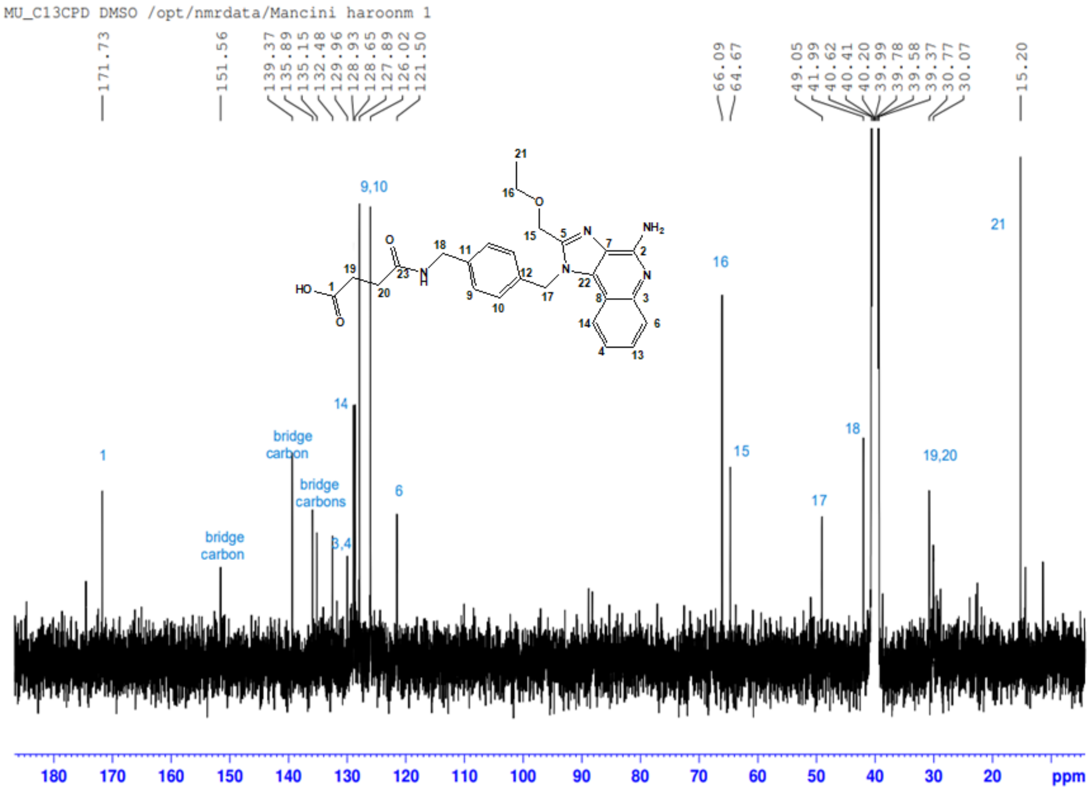

Figure S34: ESI/MS of **A1.**

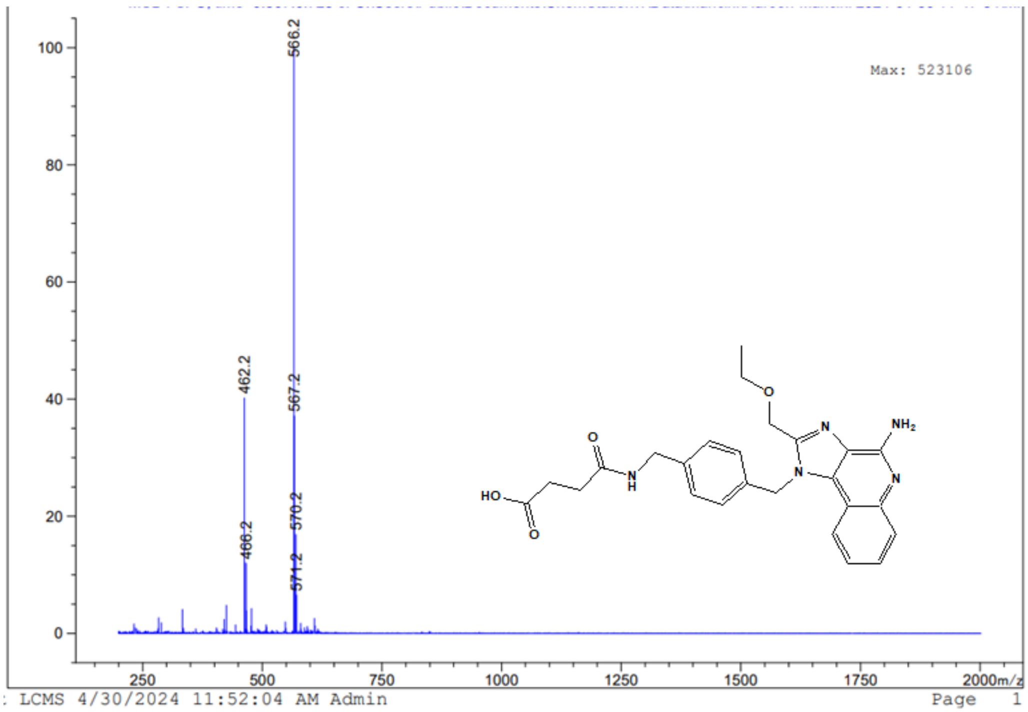

$[M+H]^+_{theoretical}$= 462.2 m/z, $[M+H]^+_{observed}$ = 462.2 m/z

Figure S35: ^{1}H NMR of **A2.**

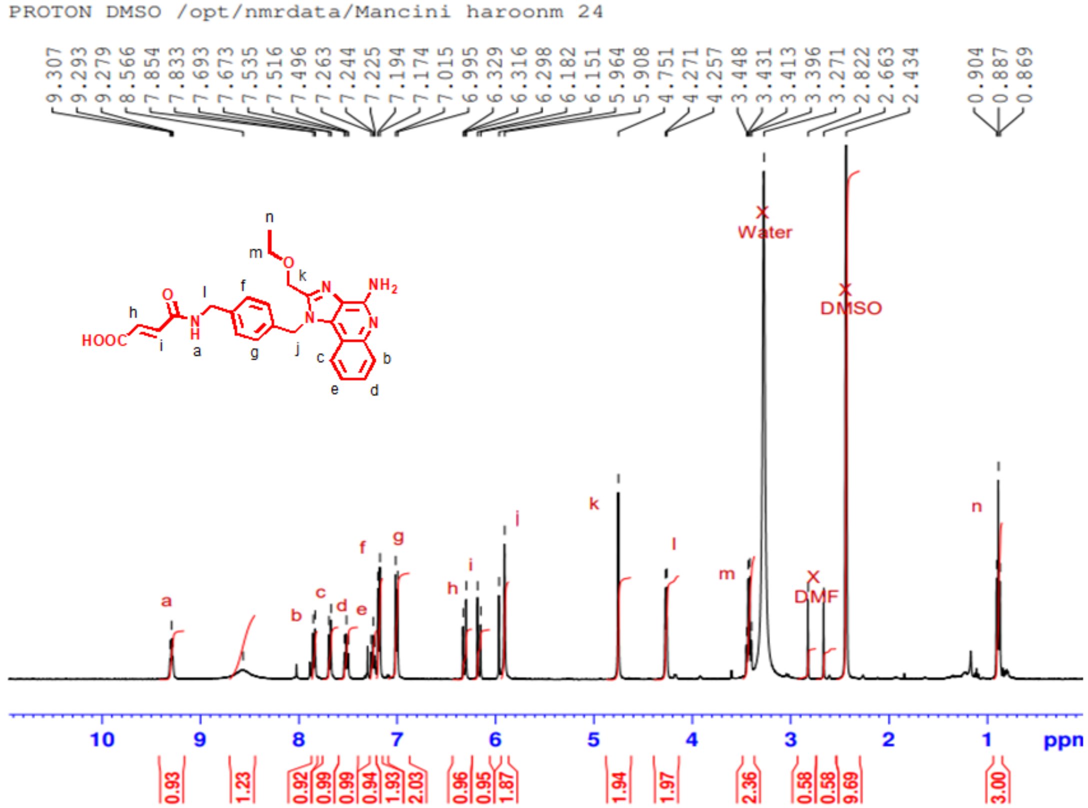

Figure S36: ^{13}C NMR of **A2.**

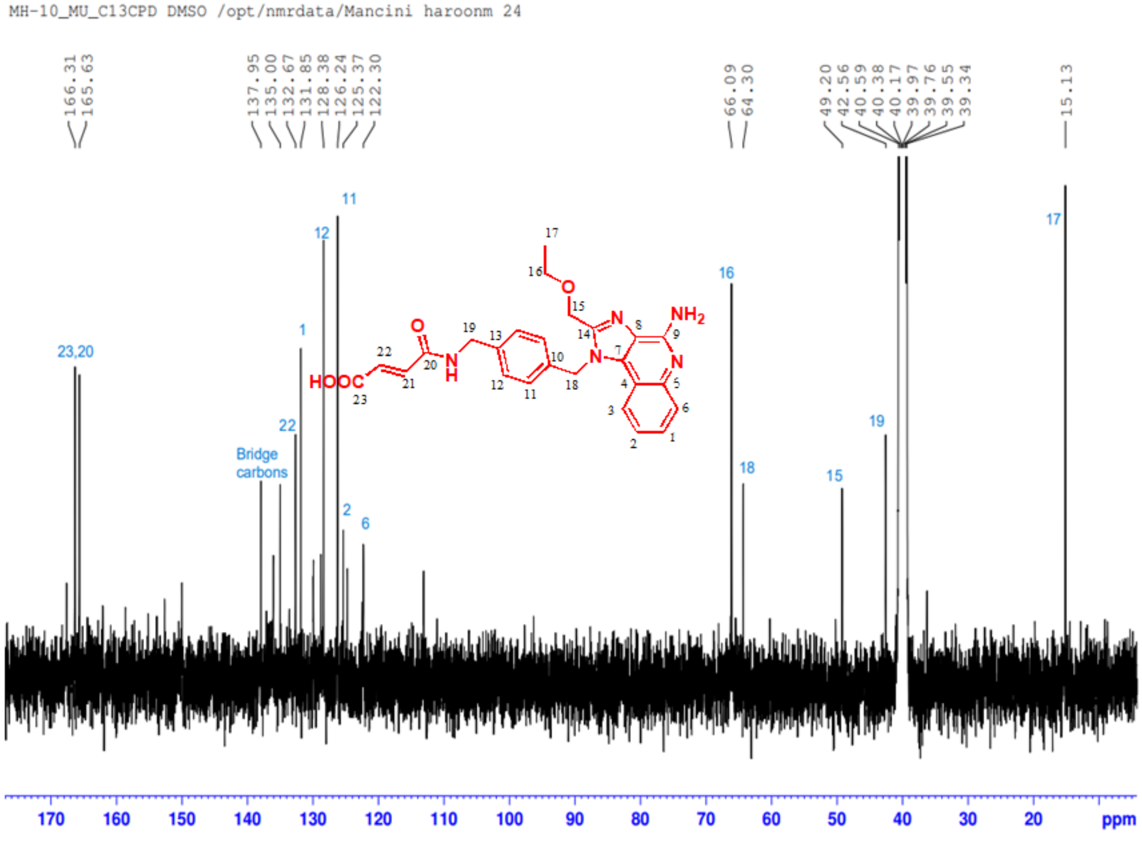

Figure S37: ESI/MS of **A2.**

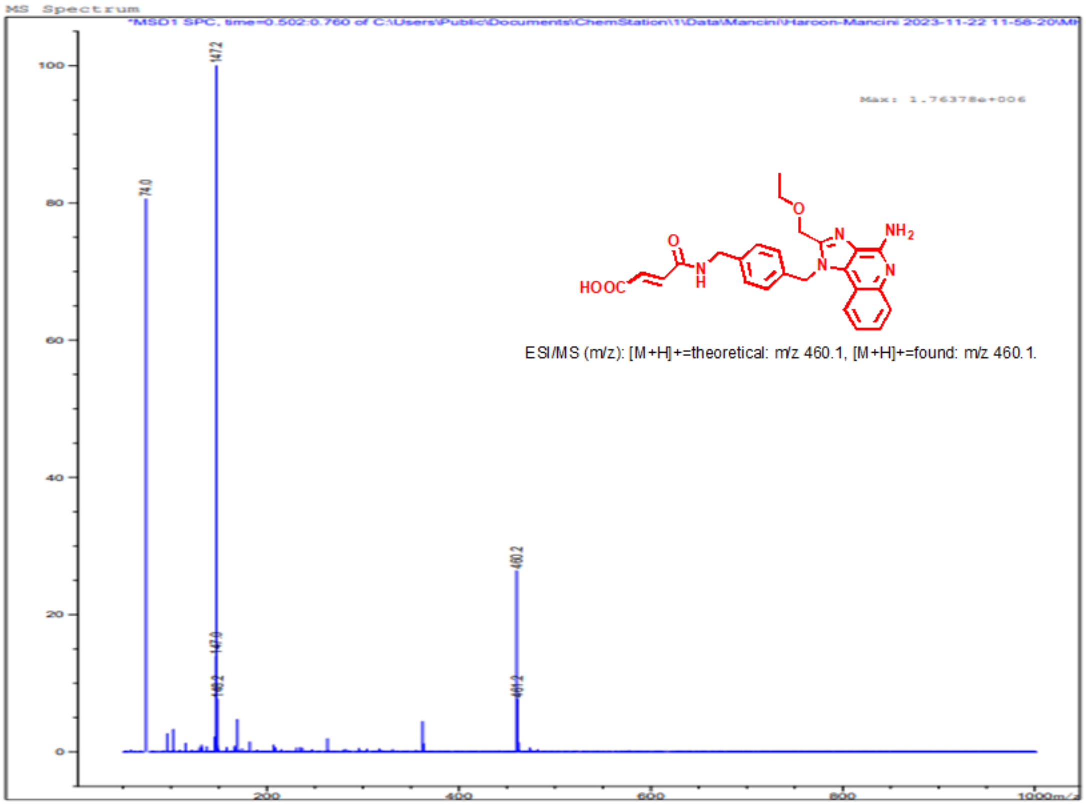

Figure S38: ^{1}H NMR of **A3.**

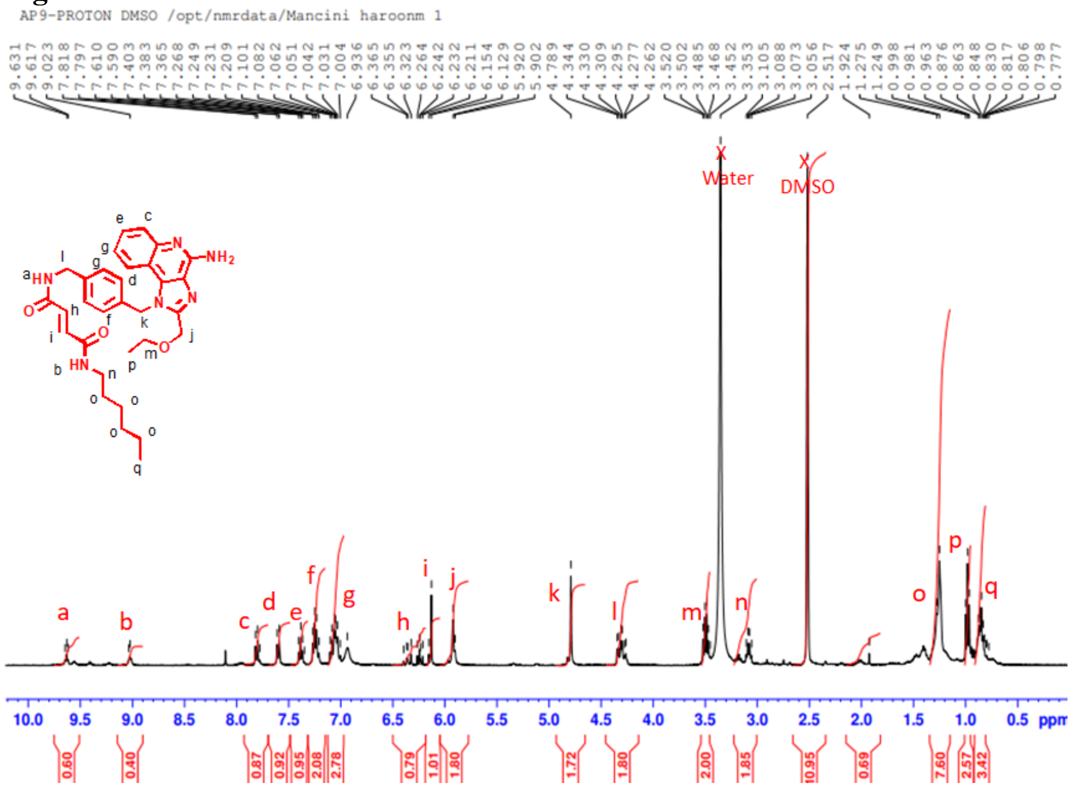

Figure S39: ^{13}C NMR of **A3.**

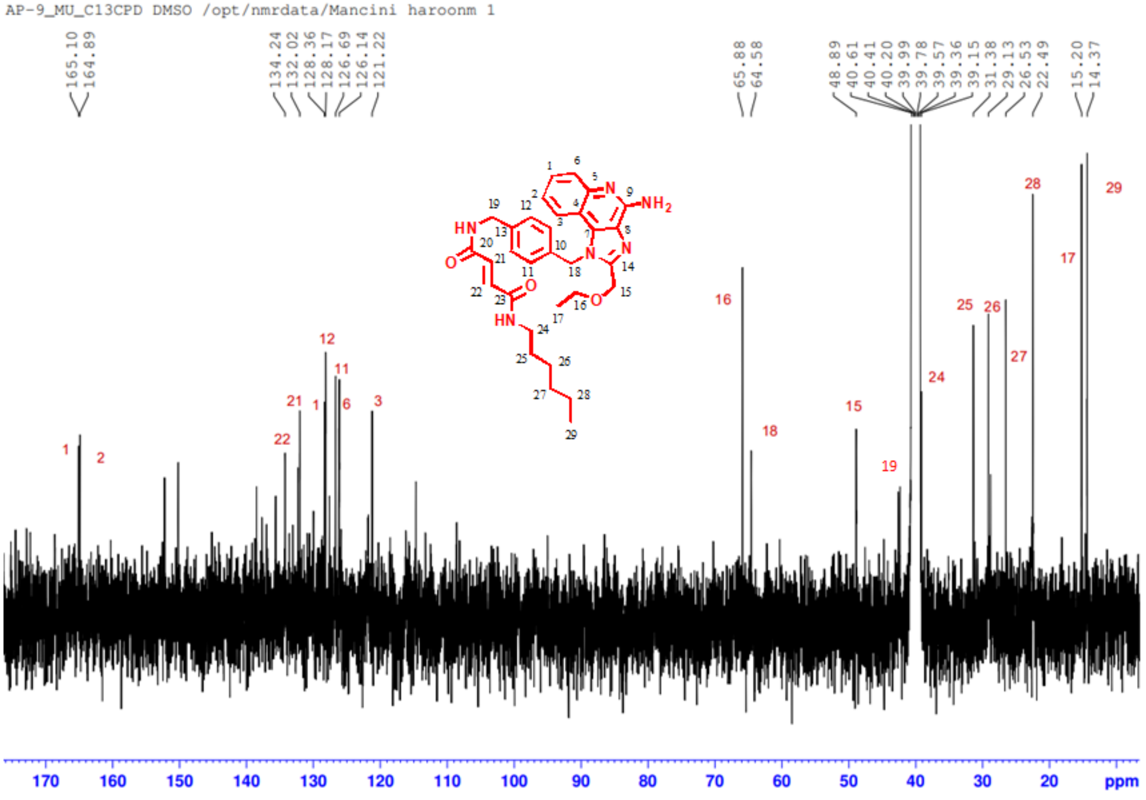

Figure S40: ESI/MS of **A3.**

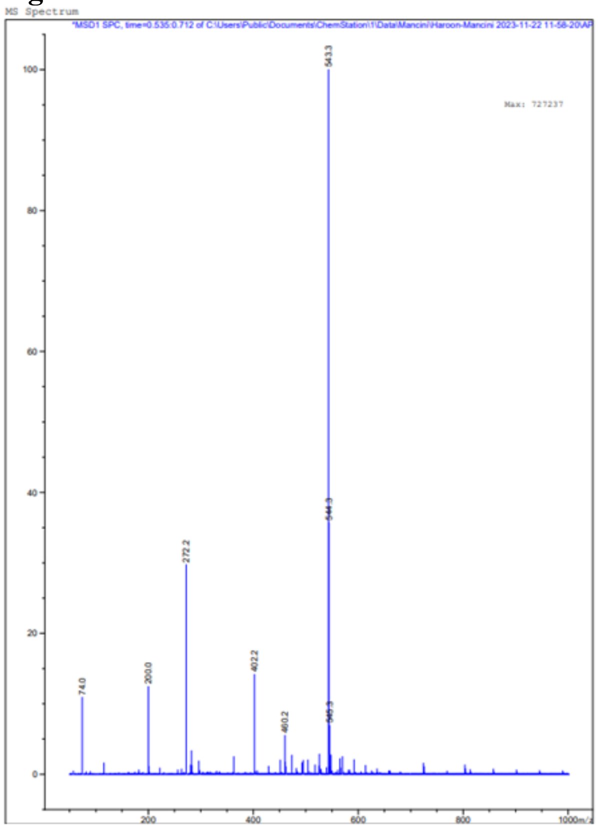

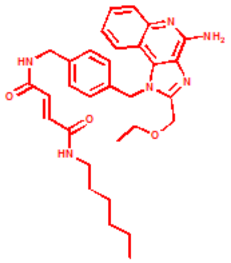

$[M+H]^+_{theoretical}$= 543.3 m/z, $[M+H]^+_{observed}$ = 543.3 m/z

Figure S41: ^{1}H NMR of **A4.**

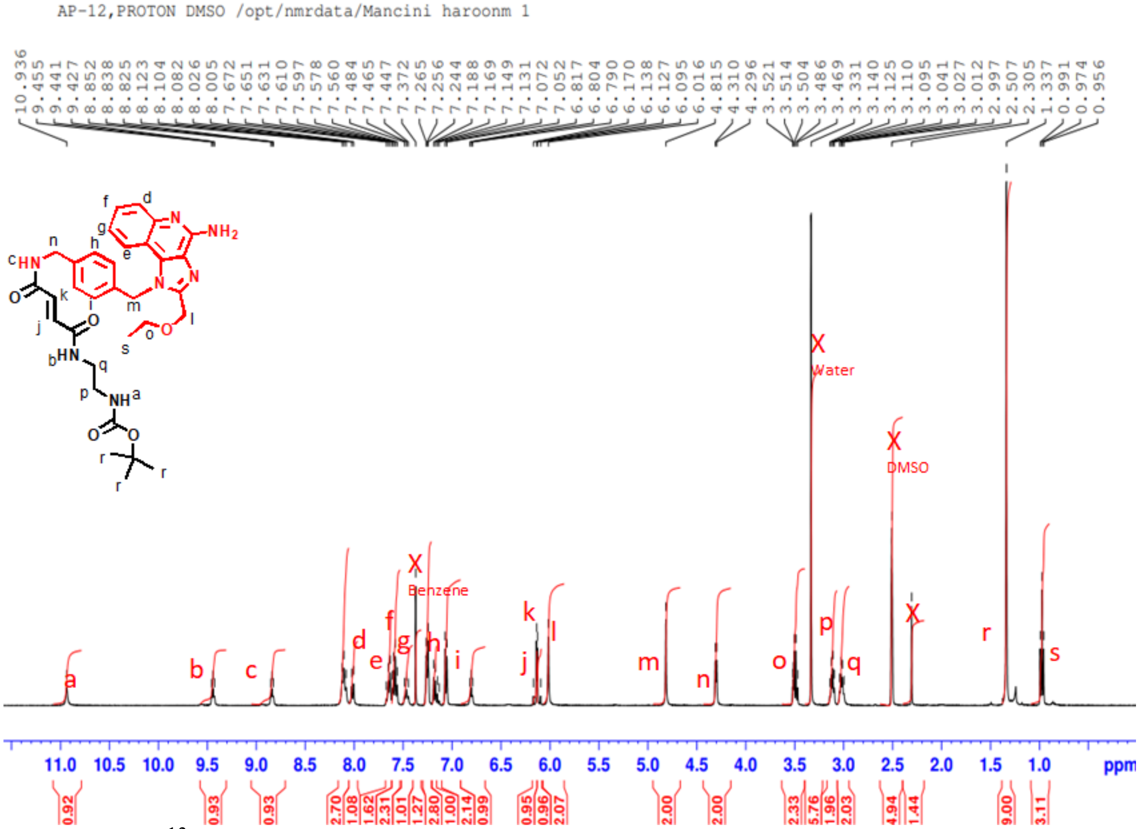

Figure S42: ^{13}C NMR of **A4.**

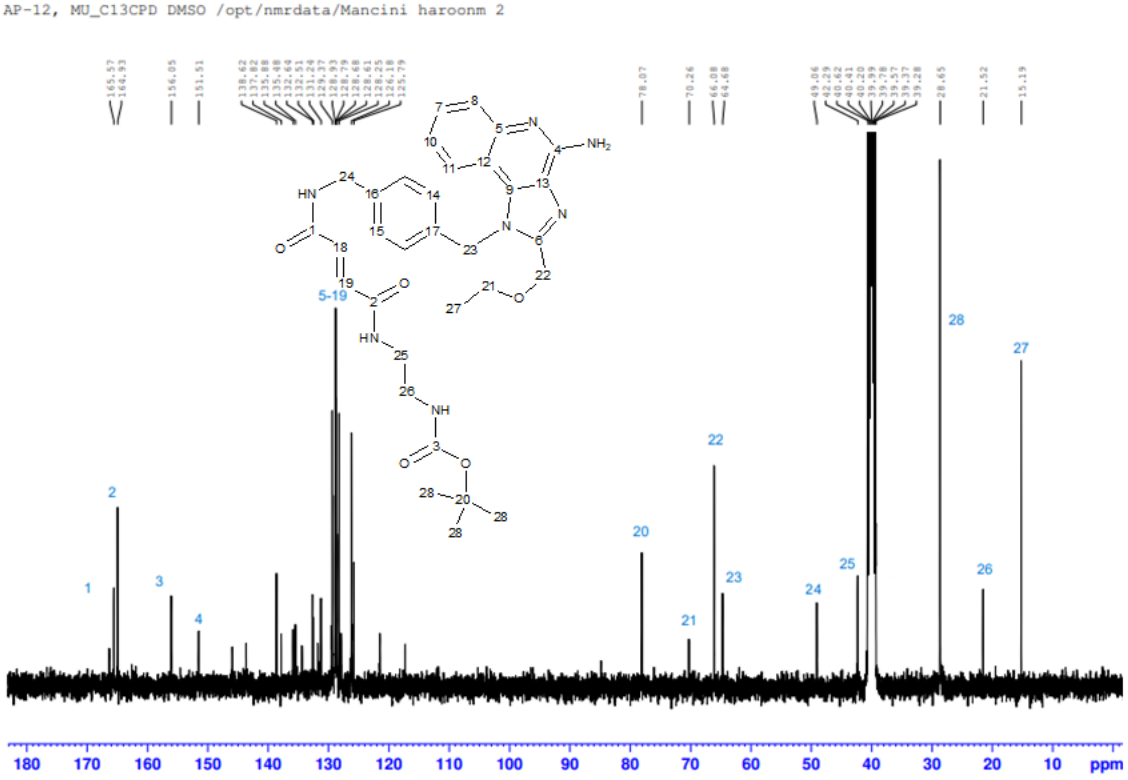

Figure S43: ^{1}H NMR of **A5.**

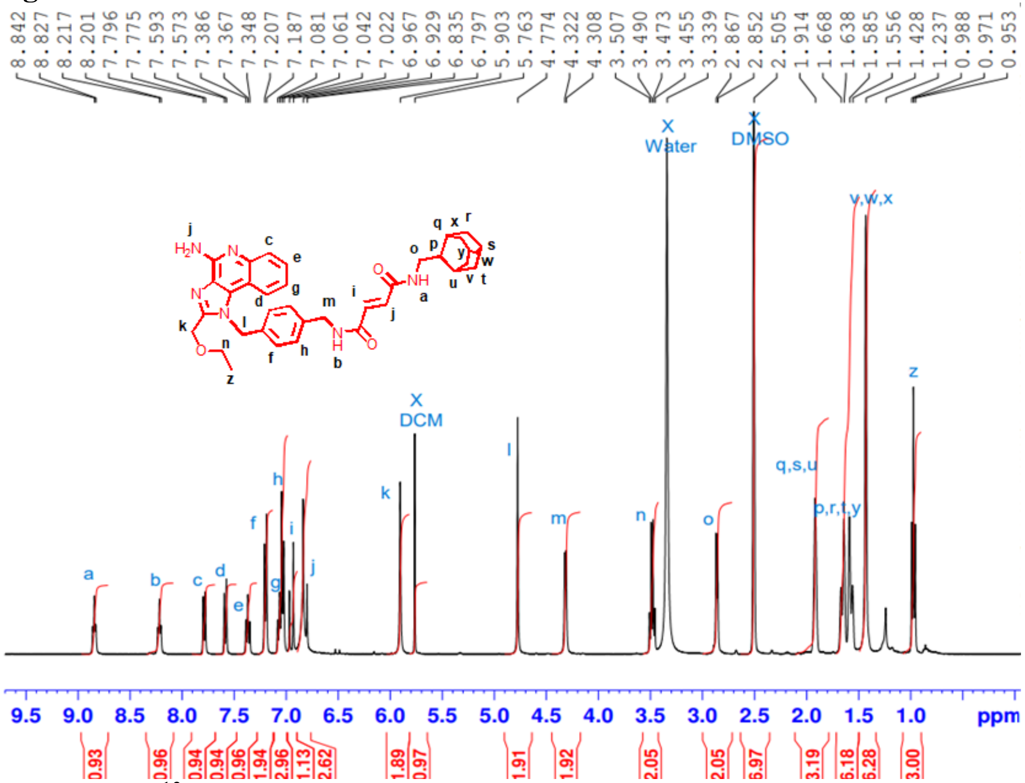

Figure S44: ^{13}C NMR of **A5.**

AMA-12_MU_C13CPD DMSO /opt/nmrdata/Mancini haroonm 14

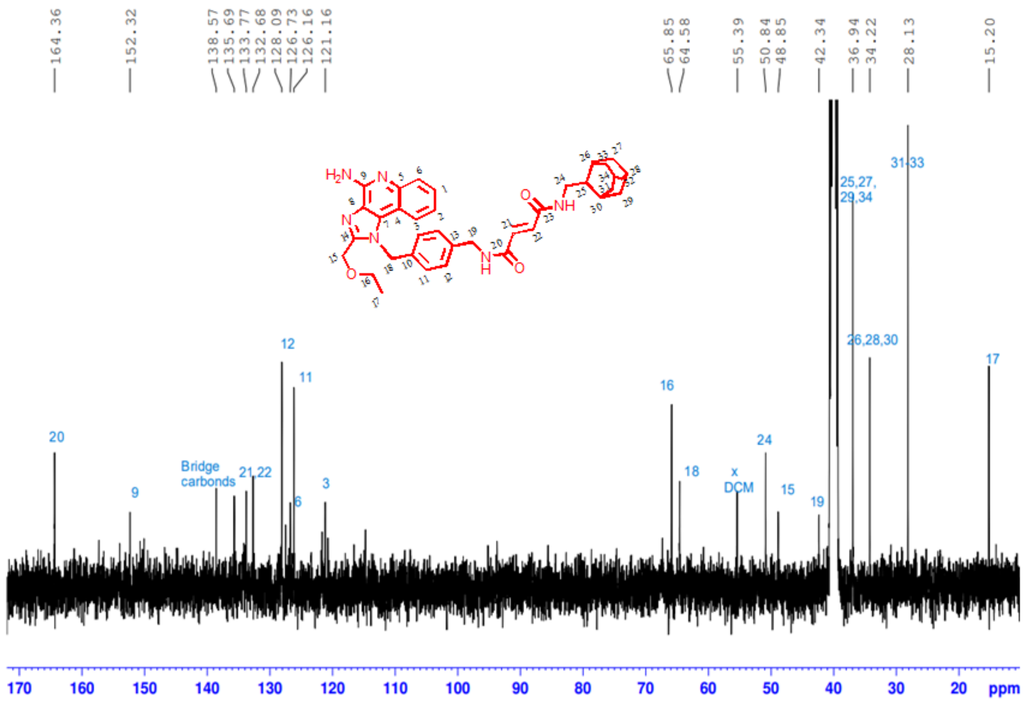

Figure S45: ESI/MS of **A5.**

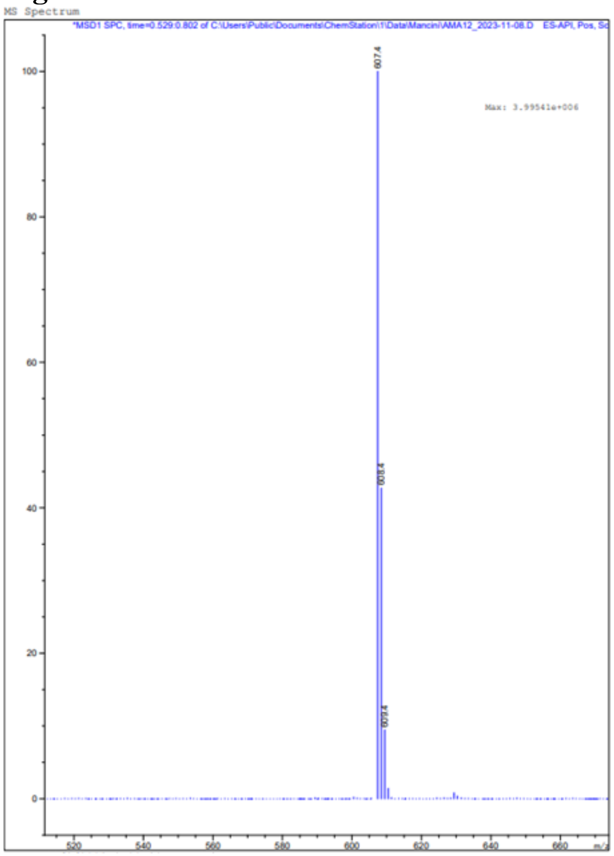

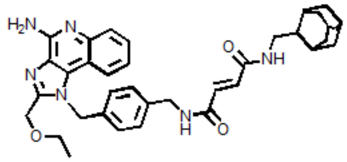

ESI/MS (m/z): $[M+H]^+_{theoretical}$= 607.4 m/z, $[M+H]^+_{found}$= 607.4 m/z

Figure S46: ^{1}H NMR of **A6.**

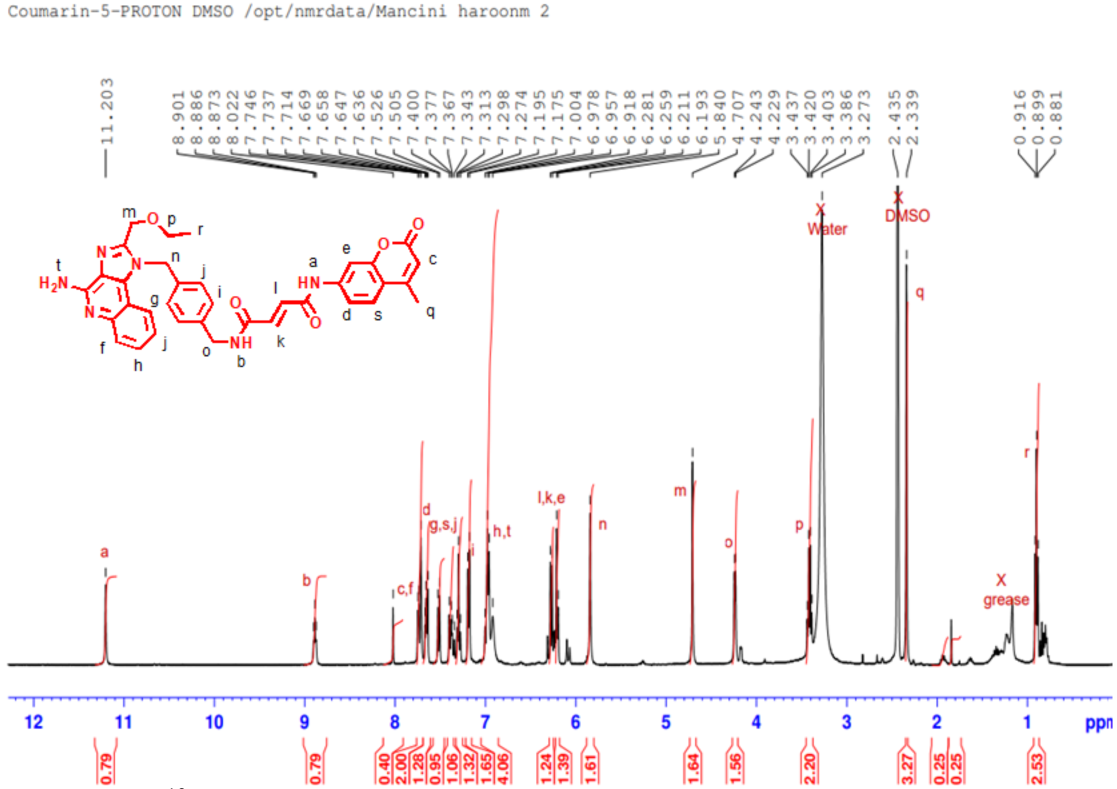

Figure S47: ^{13}C NMR of **A6.**

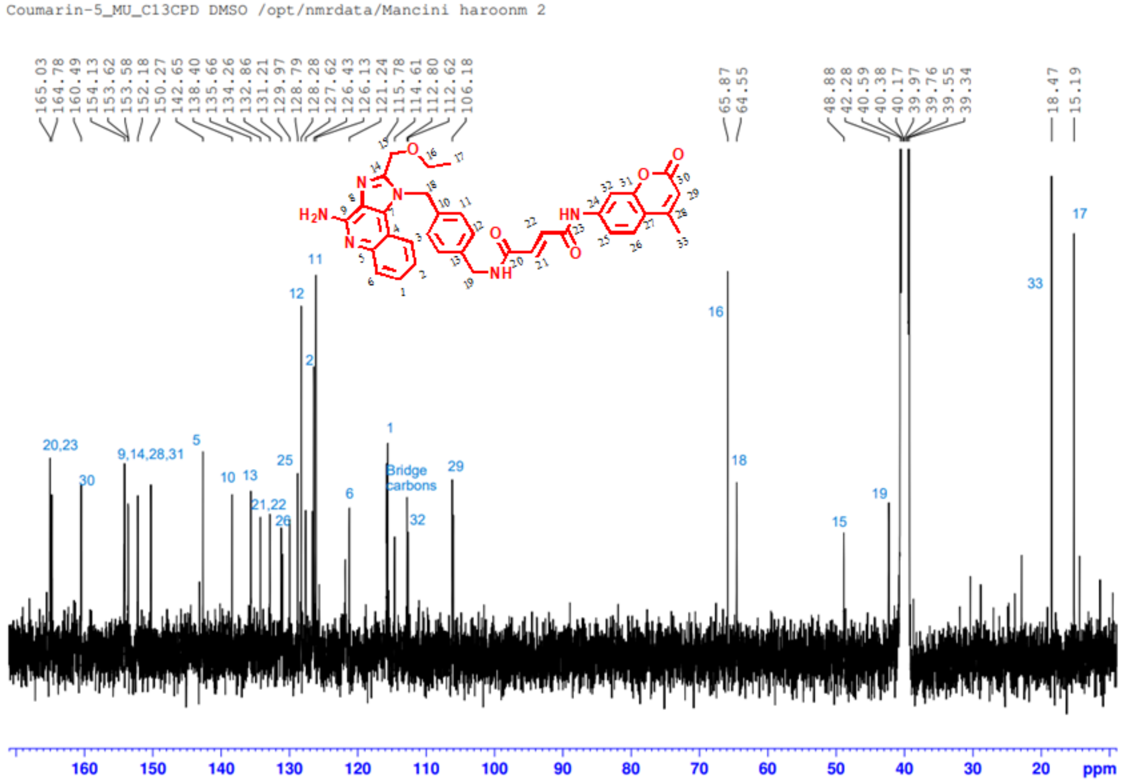

Figure S48: ESI/MS of **A6.**

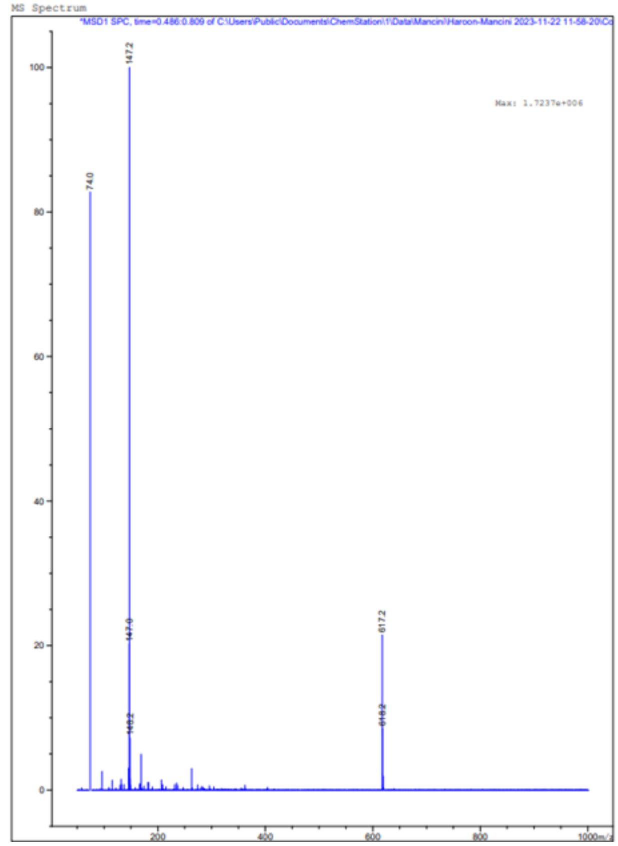

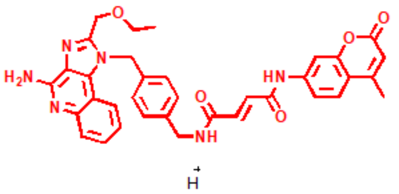

ESI/MS (m/z): $[M+H]^+_{theoretical}$= 617.2, $[M+H]^+_{found}$= 617.2.

Figure S49: ^{1}H NMR of **A7.**

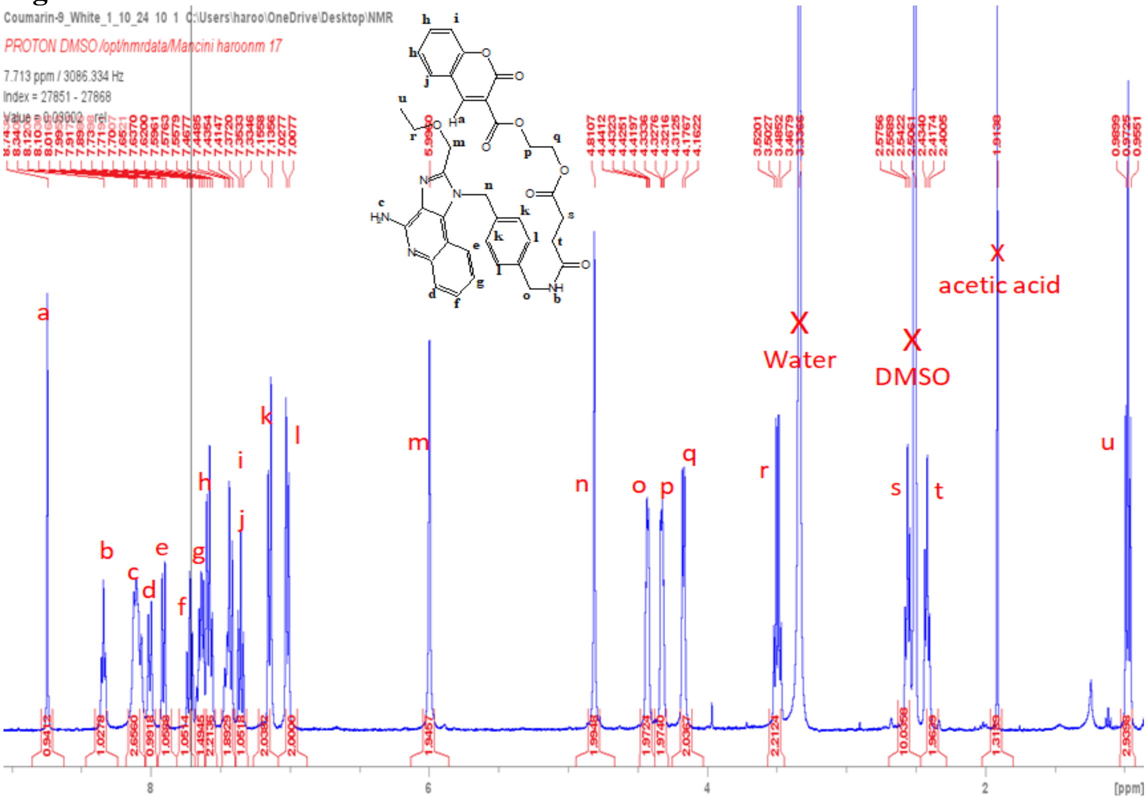

Figure S50: ^{13}C NMR of **A7.**

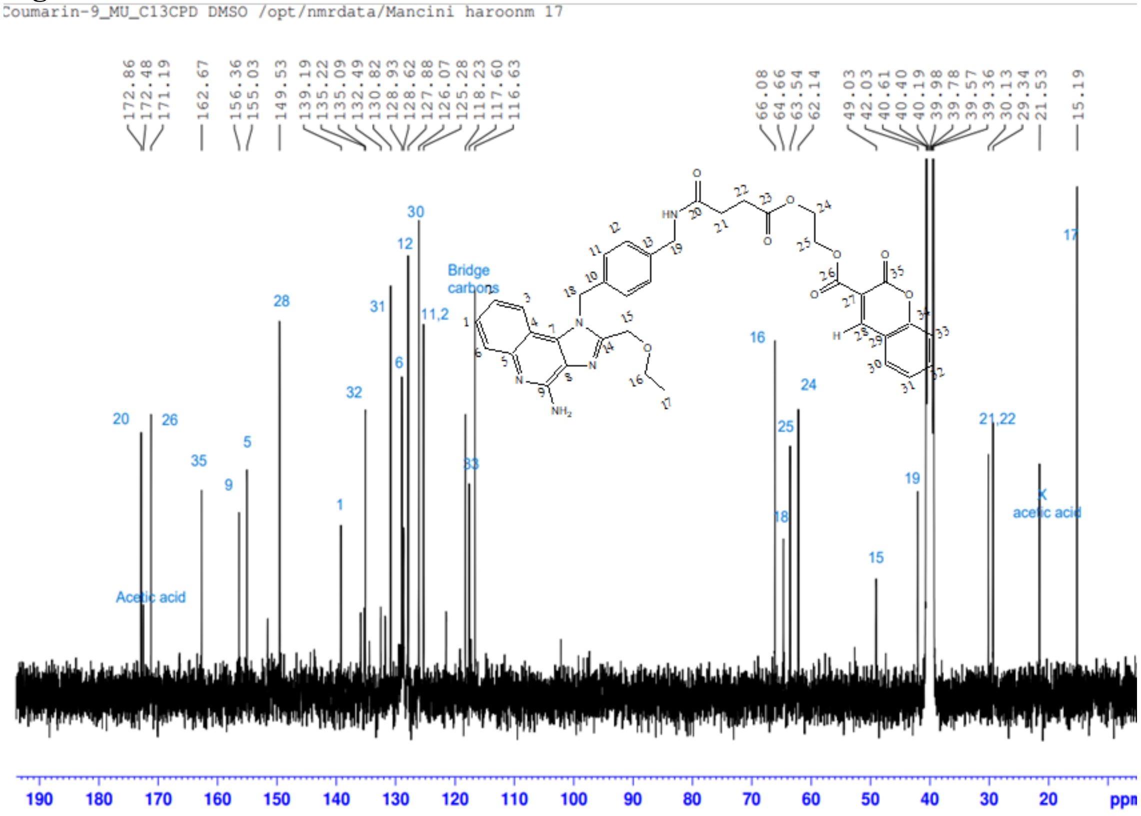

Figure S51: ESI/MS of **A7.**

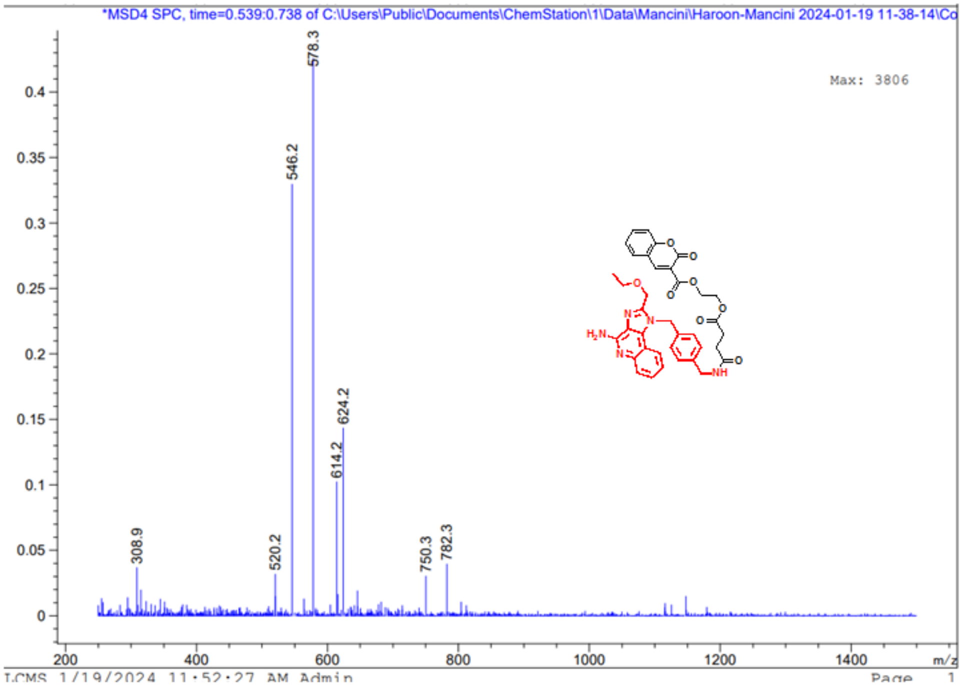

ESI/MS= $M^{-1}_{theoretical}$= 624.2 m/z, $M^{-1}_{observed}$= 624.2 m/z.

Figure S52: ^{1}H NMR of **A8.**

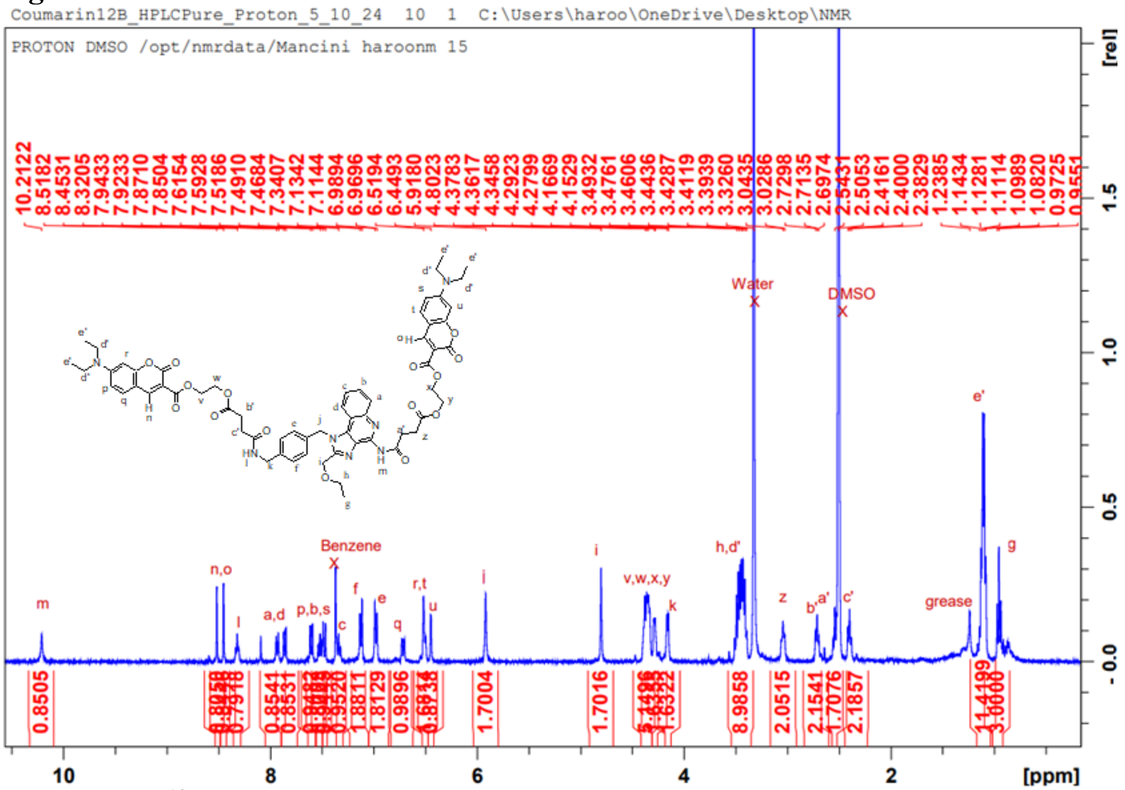

Figure S53: ^{13}C NMR of **A8.**

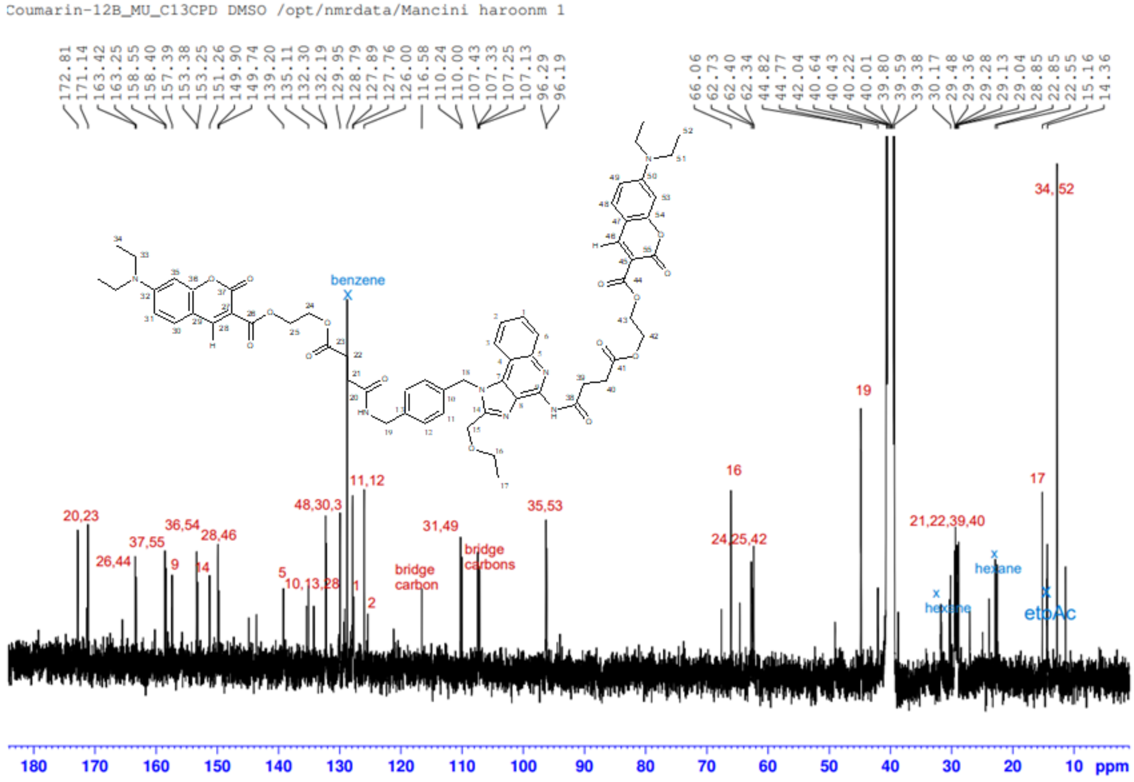

Figure S54: ESI/MS of **A8.**

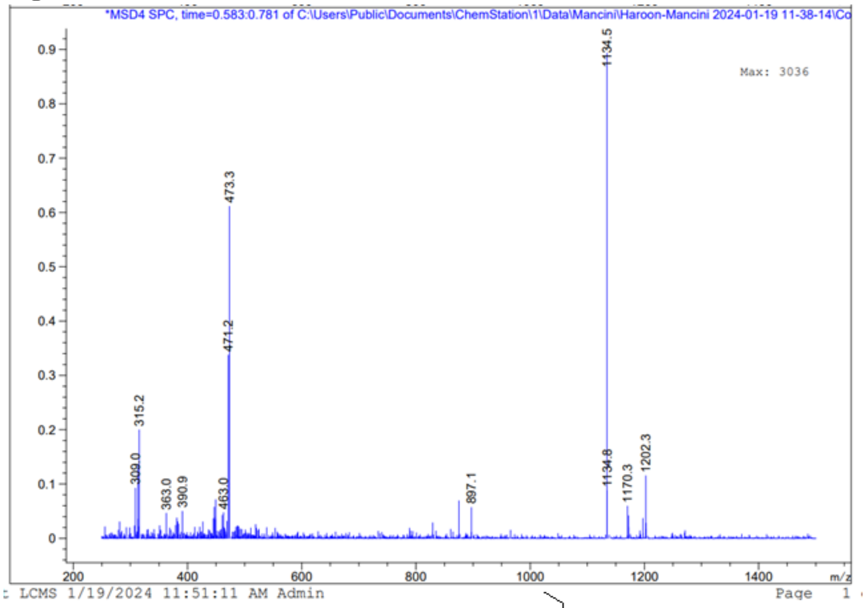

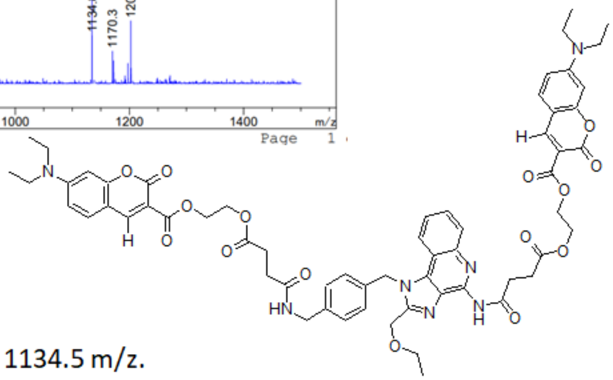

ESI/MS= $M^{-1}_{theoretical}$= 1134.5 m/z, $M^{-1}_{observed}$= 1134.5 m/z.

Figure S55: ^{1}H NMR of **A9.**

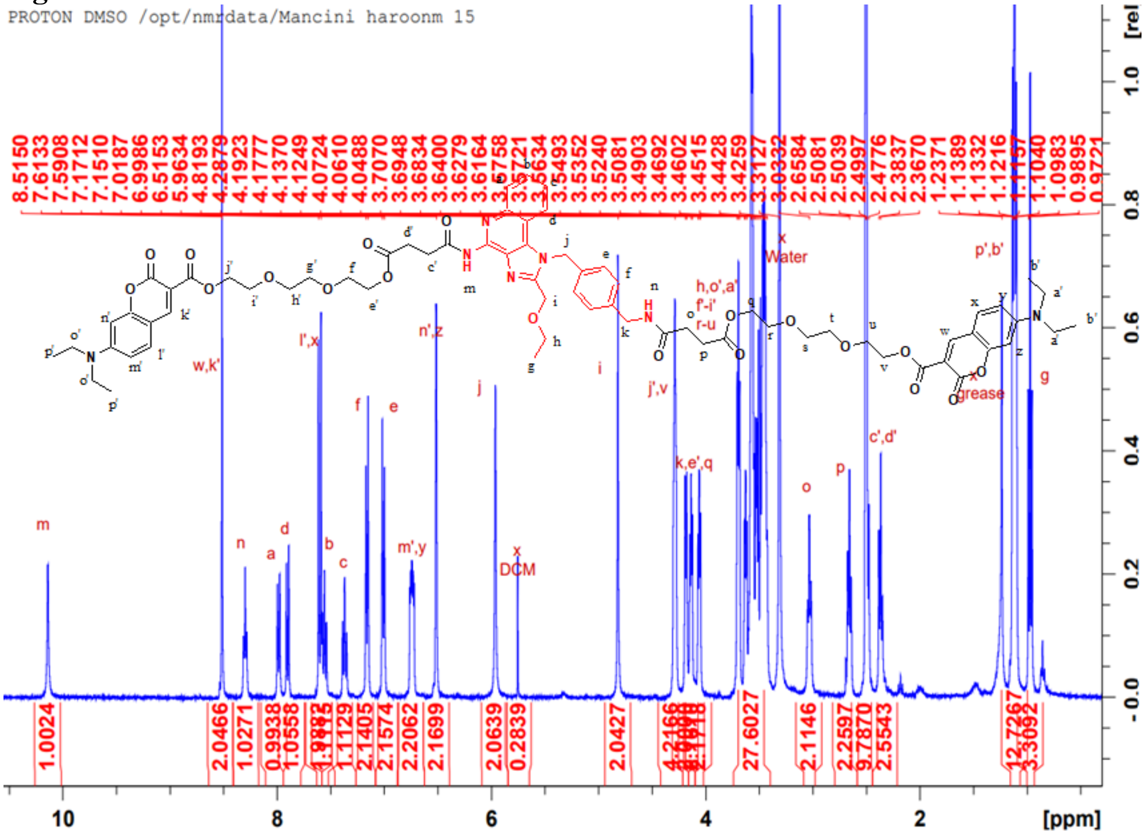

Figure S56: ^{13}C NMR of **A9.**

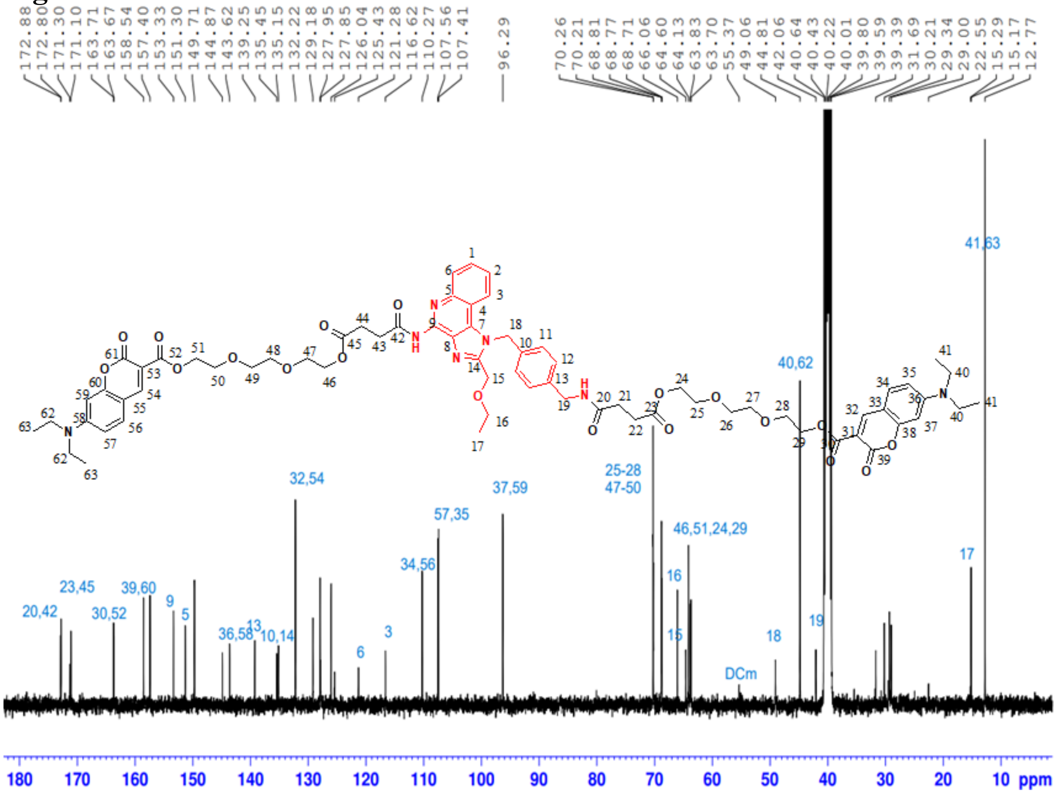

Figure S57: ESI/MS of **A9.**

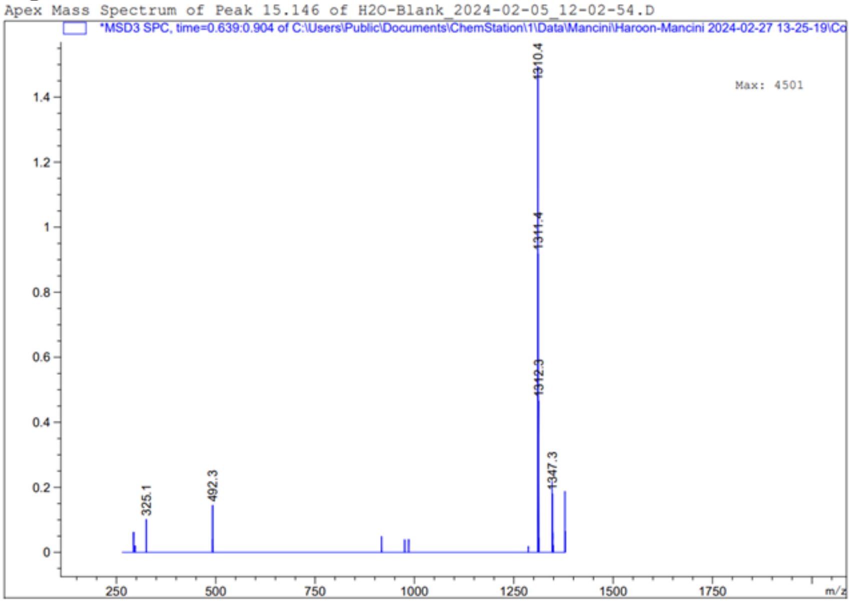

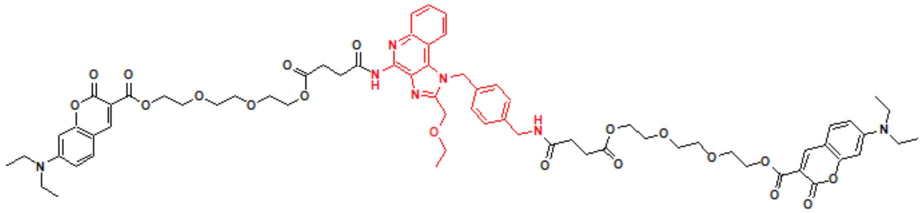

ESI/MS= $M^{-1}_{theoretical}$= 1310.4 m/z, $M^{-1}_{observed}$= 1310.4 m/z.

Figure S58: ^{1}H NMR of **A10.**

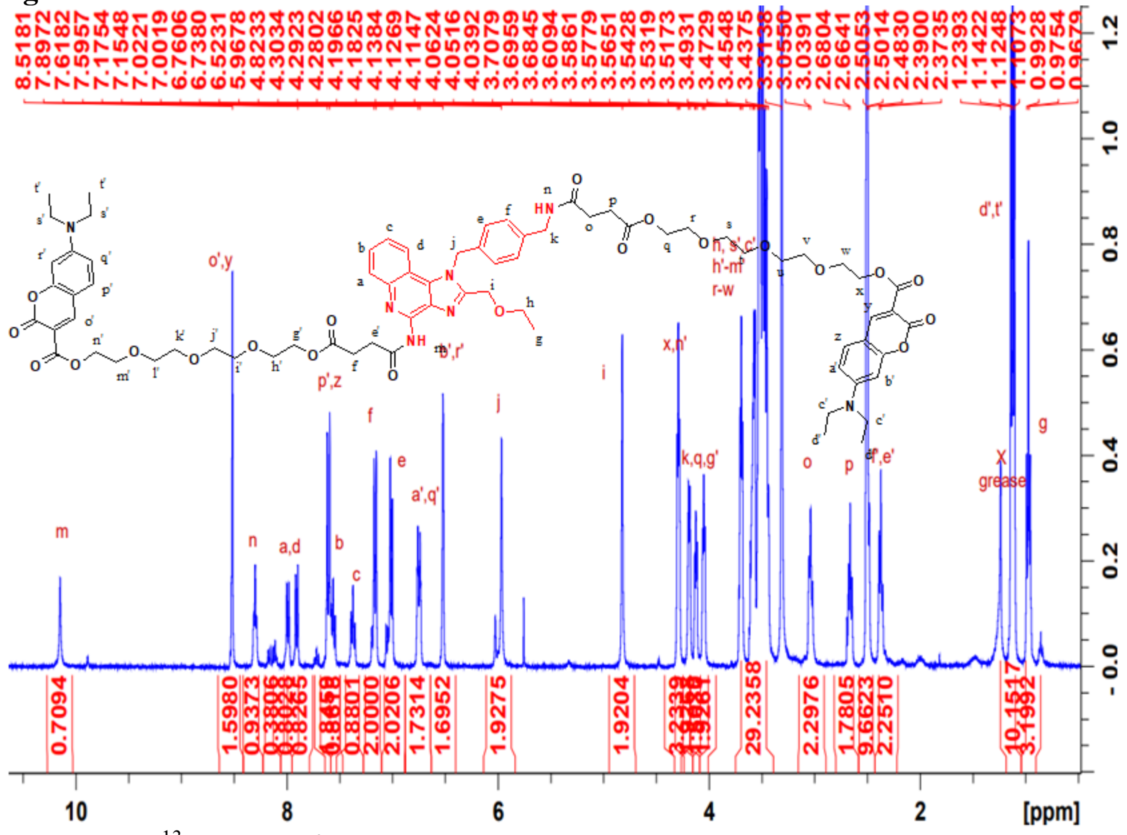

Figure S59: ^{13}C NMR of **A10.**

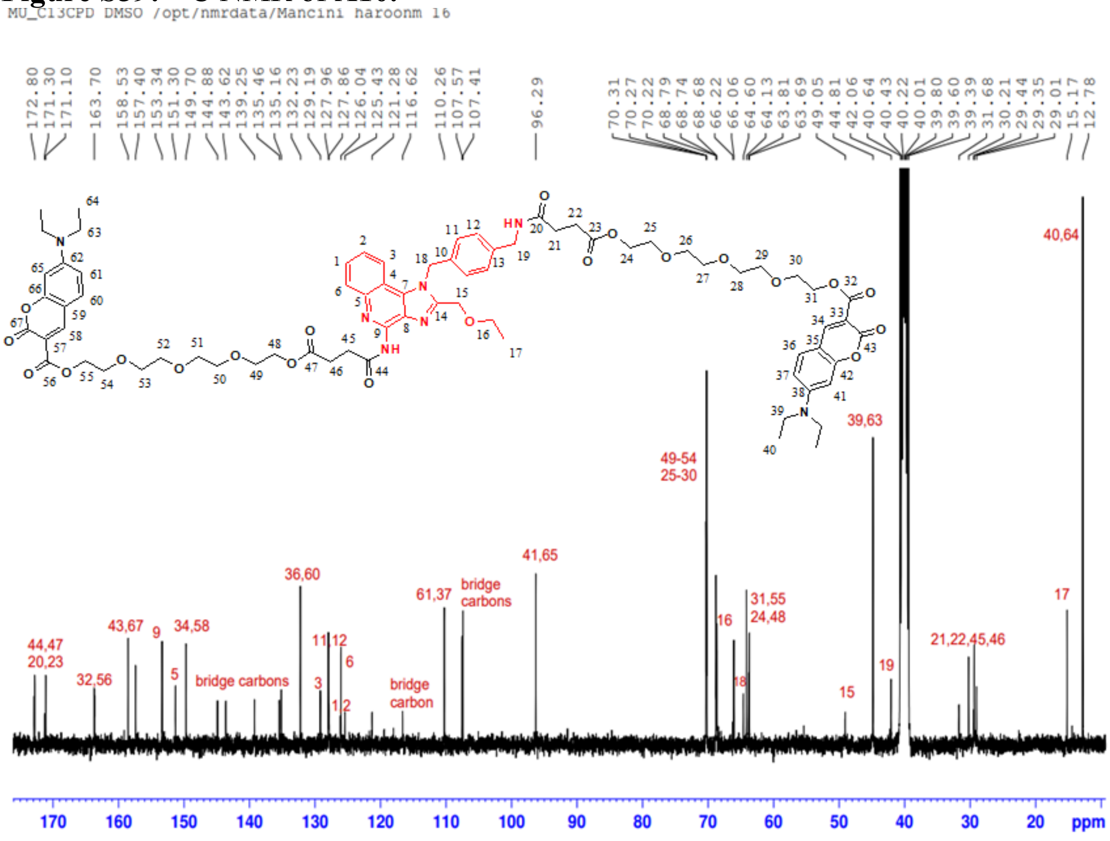

Figure S60: ESI/MS of **A10.**

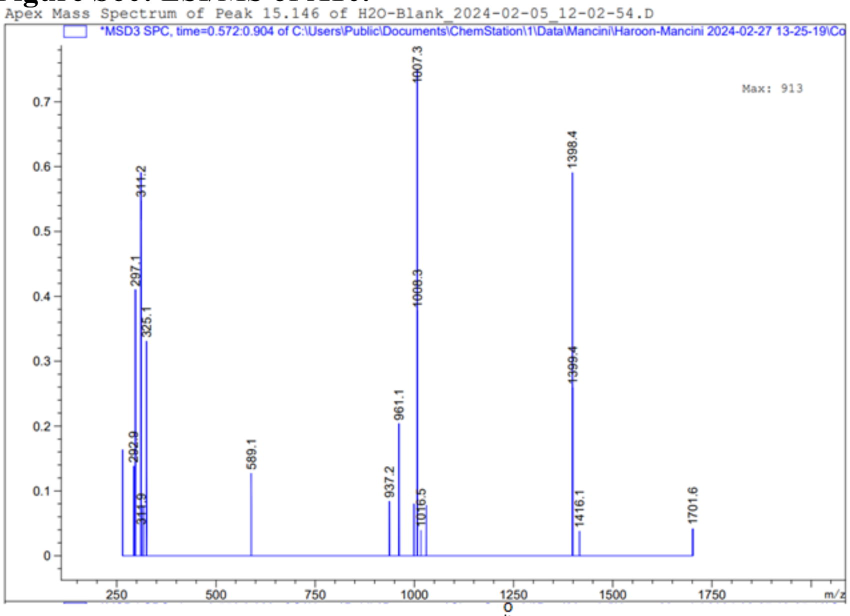

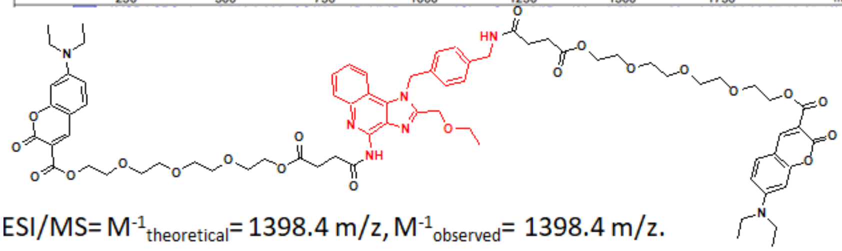

ESI/MS= $M^{-1}_{theoretical}$= 1398.4 m/z, $M^{-1}_{observed}$= 1398.4 m/z.

Figure S61: ^{1}H NMR of **A11.**

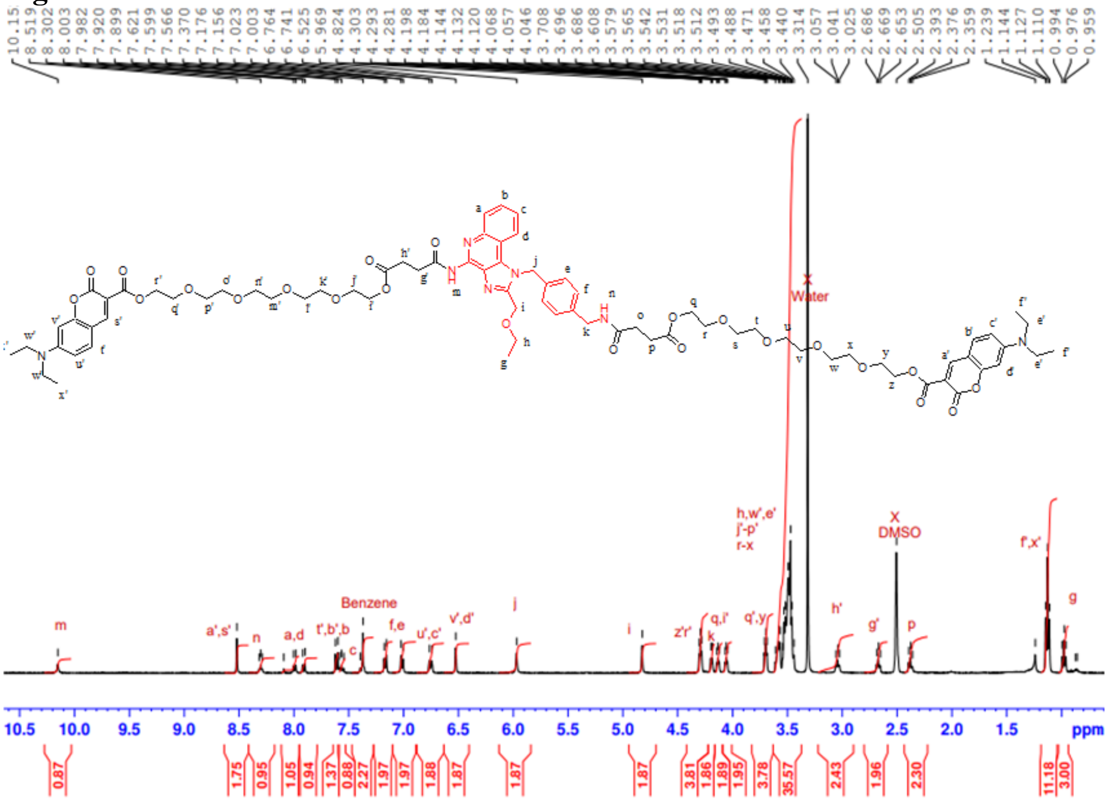

Figure S62: ^{13}C NMR of **A11.**

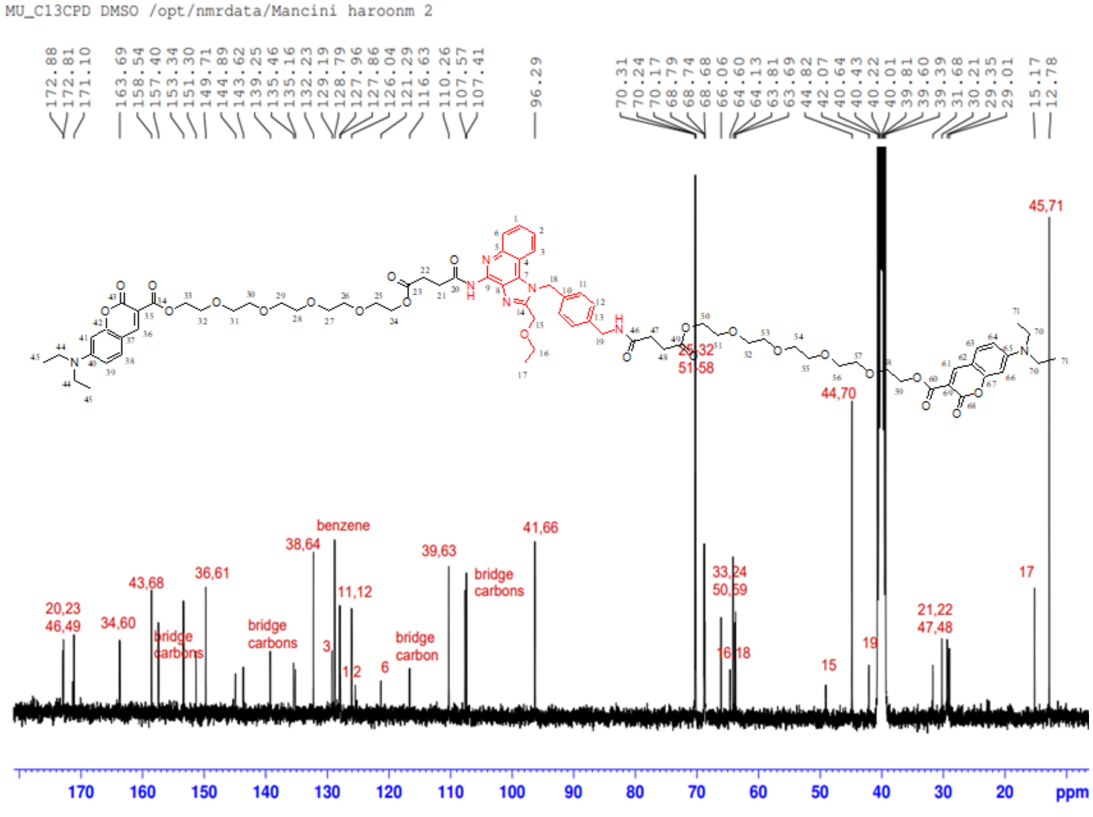

Figure S63: ESI/MS of **A11**

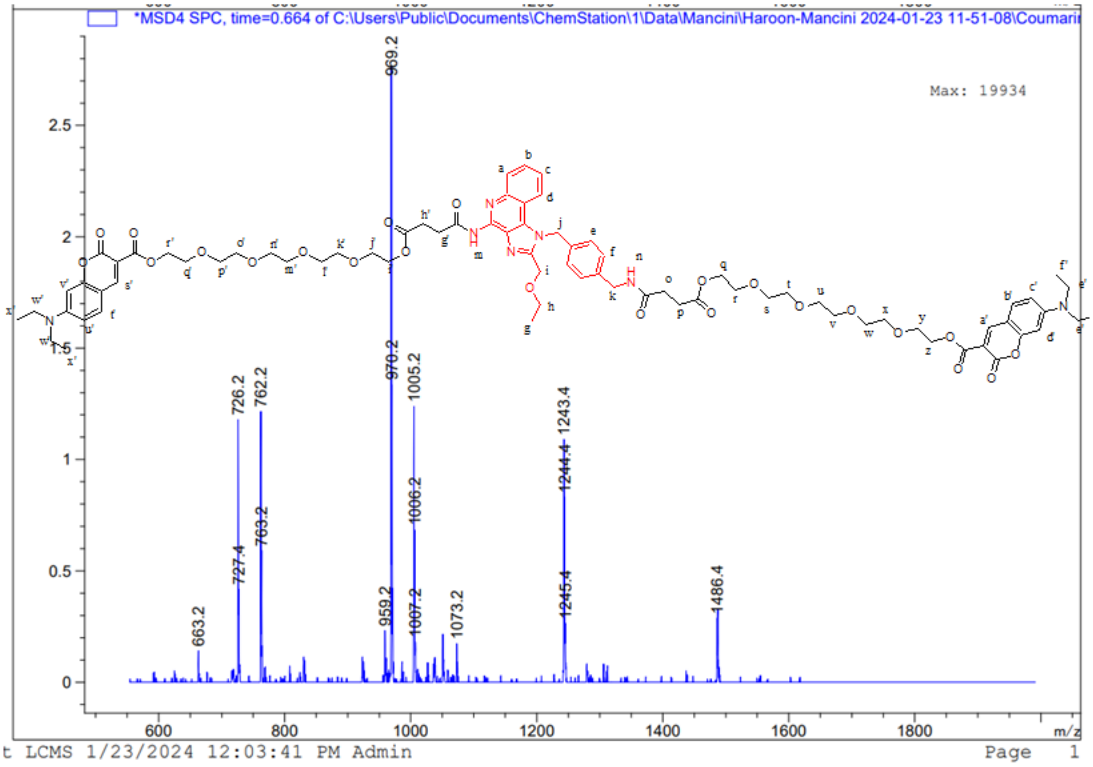

ESI/MS= $M^{-1}_{theoretical}$= 1486.4 m/z. $M^{-1}_{observed}$= 1486.4 m/z.

Figure S64: ^{1}H NMR of **A12.**

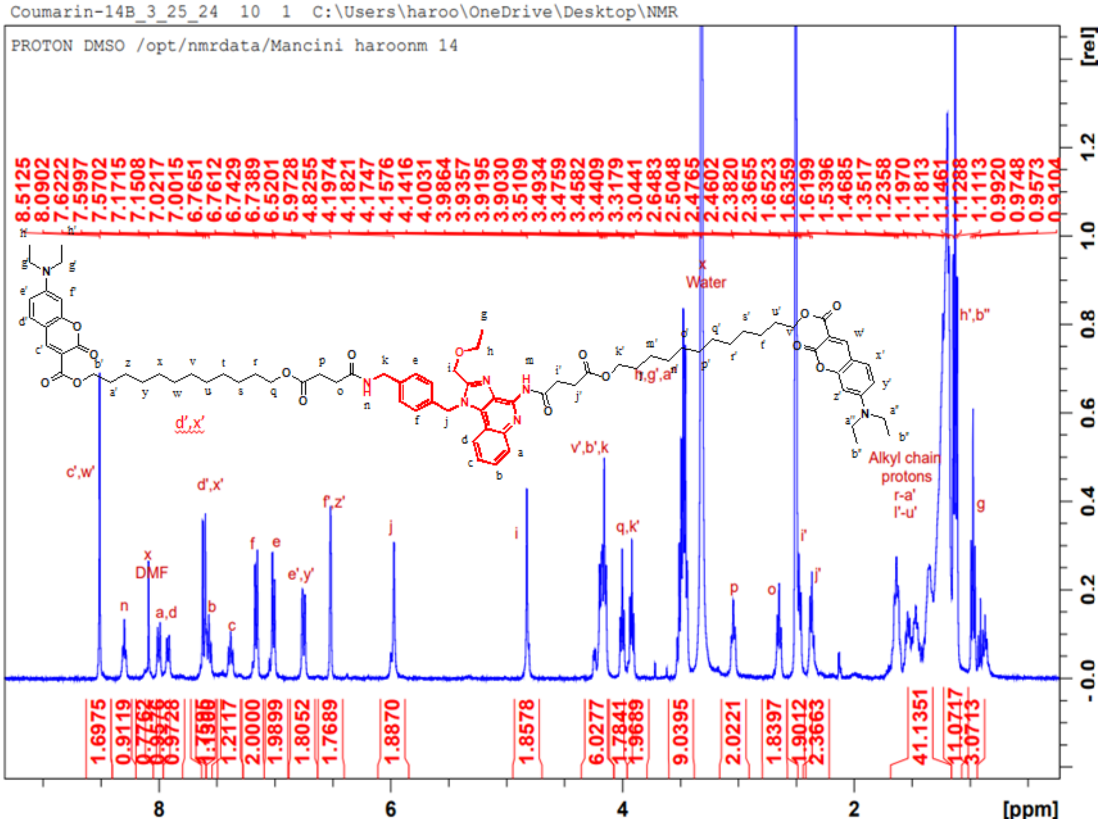

Figure S65: ^{13}C NMR of **A12.**

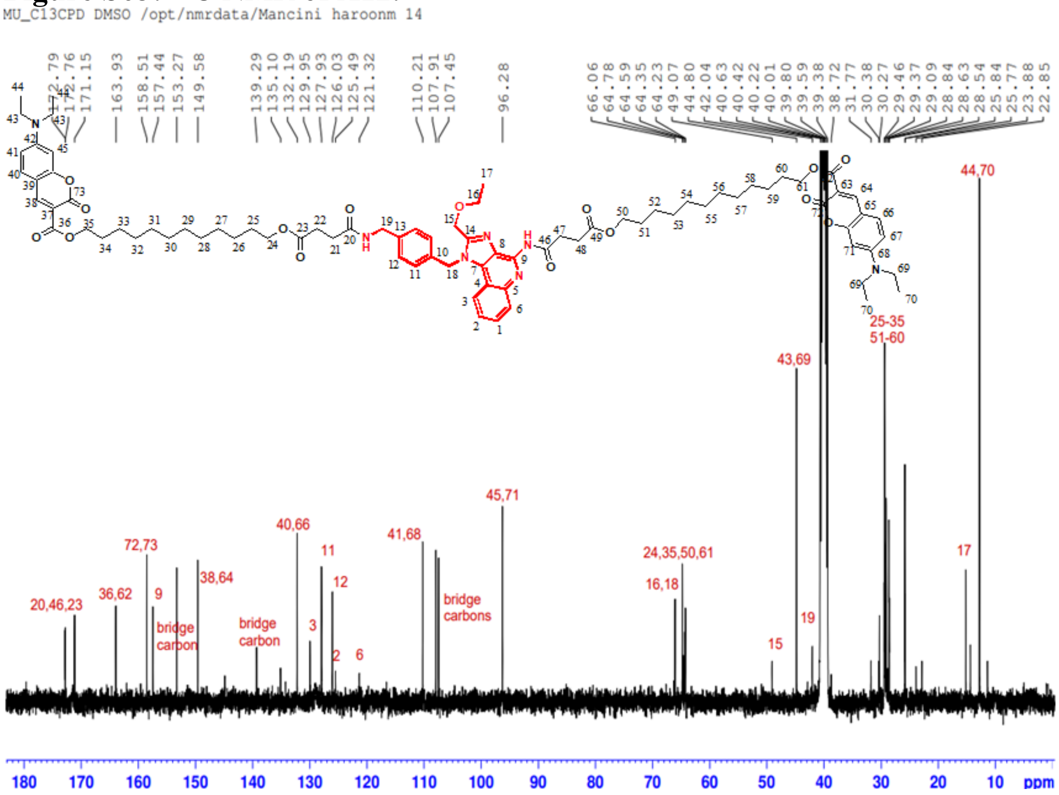

Figure S66: ^{1}H NMR of **S1.**

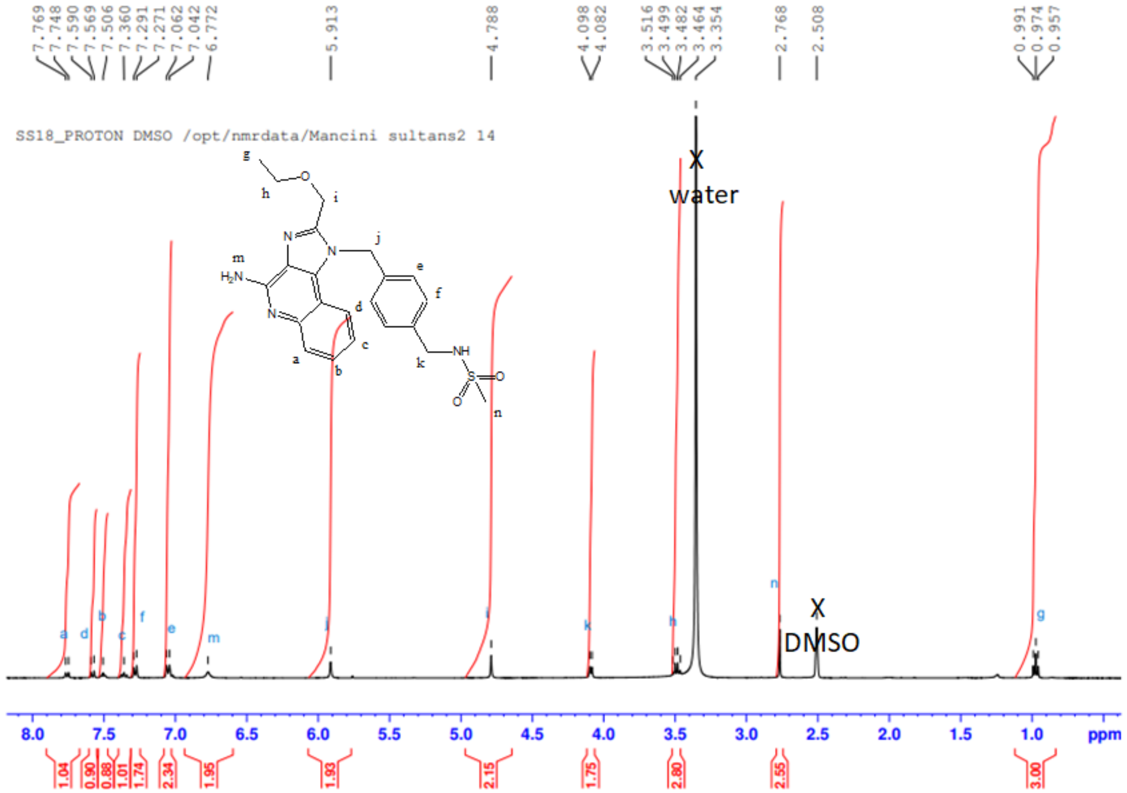

Figure S67: ^{13}C NMR of **S1.**

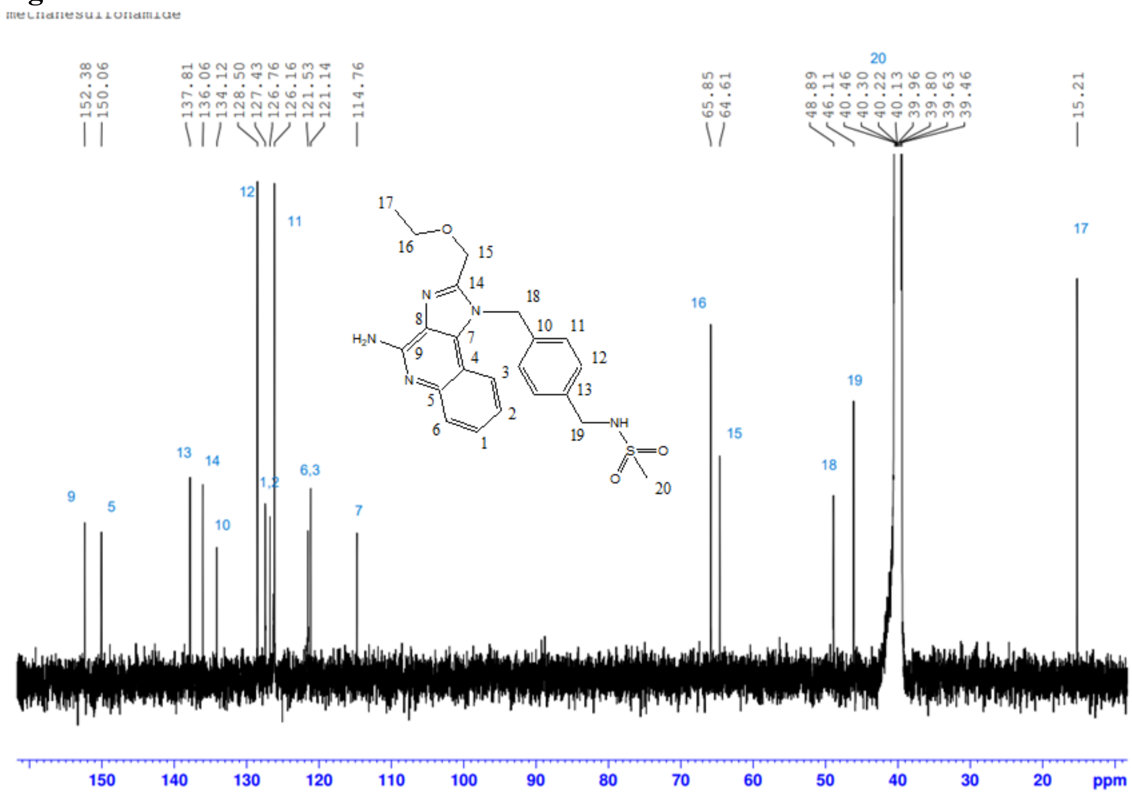

Figure S68: ESI/MS of **S1.**

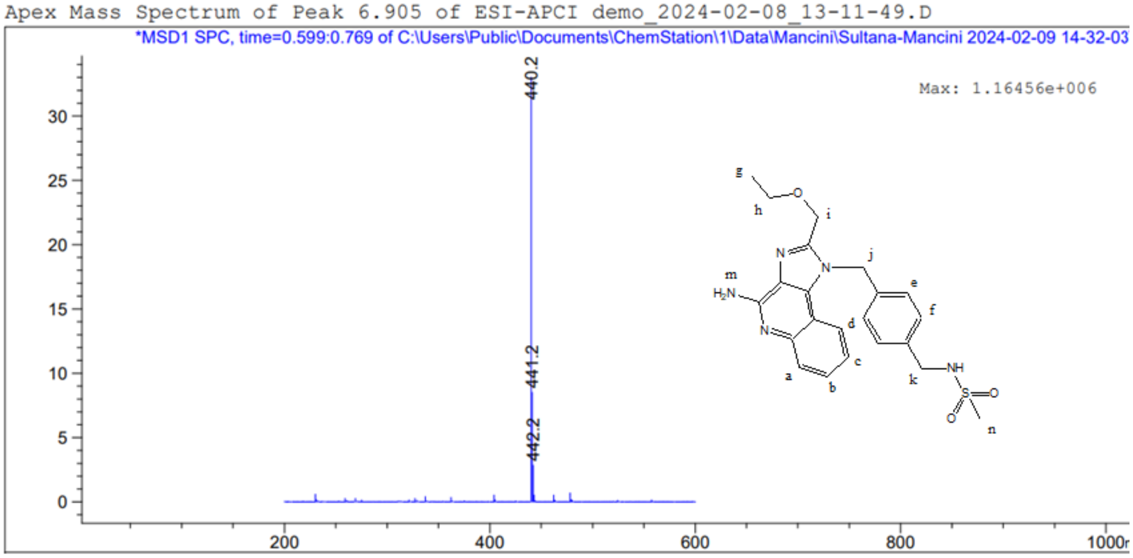

[M+H]$^+_{theoretical}$= 440.2 m/z, [M+H]$^+_{observed}$ = 440.2 m/z

Figure S69: ^{1}H NMR of **S2.**

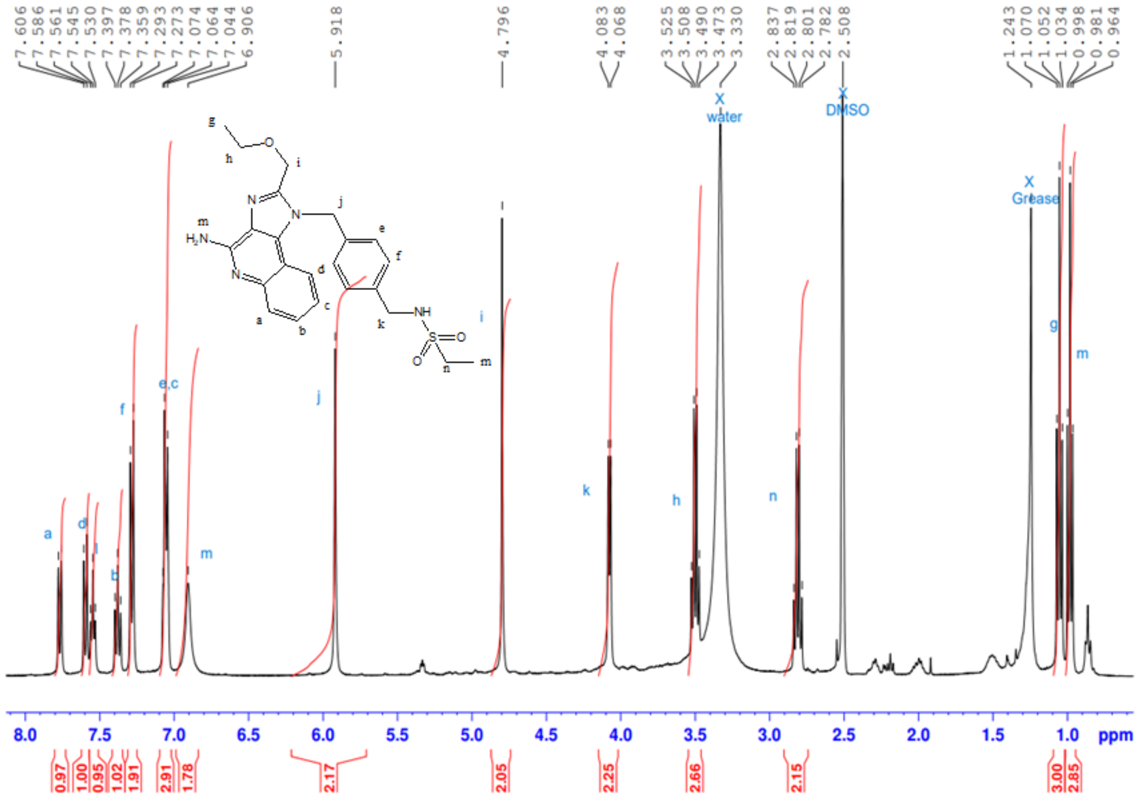

Figure S70: ^{13}C NMR of **S2.**

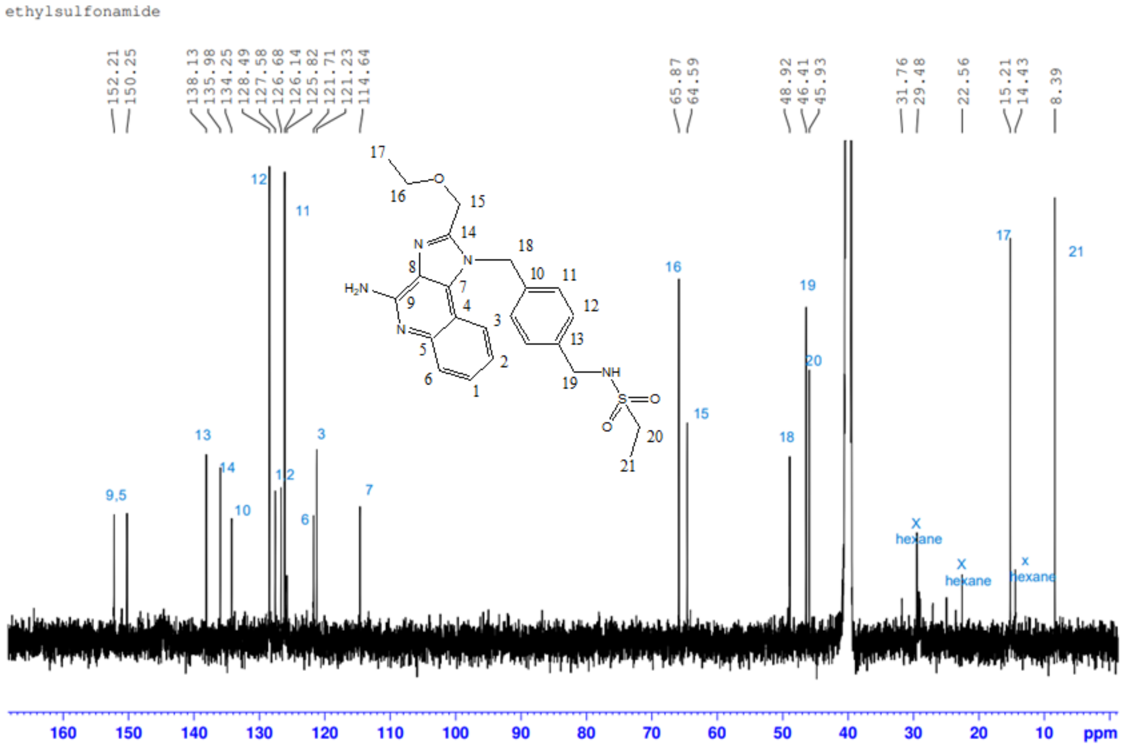

Figure S71: ESI/MS of **S2.**

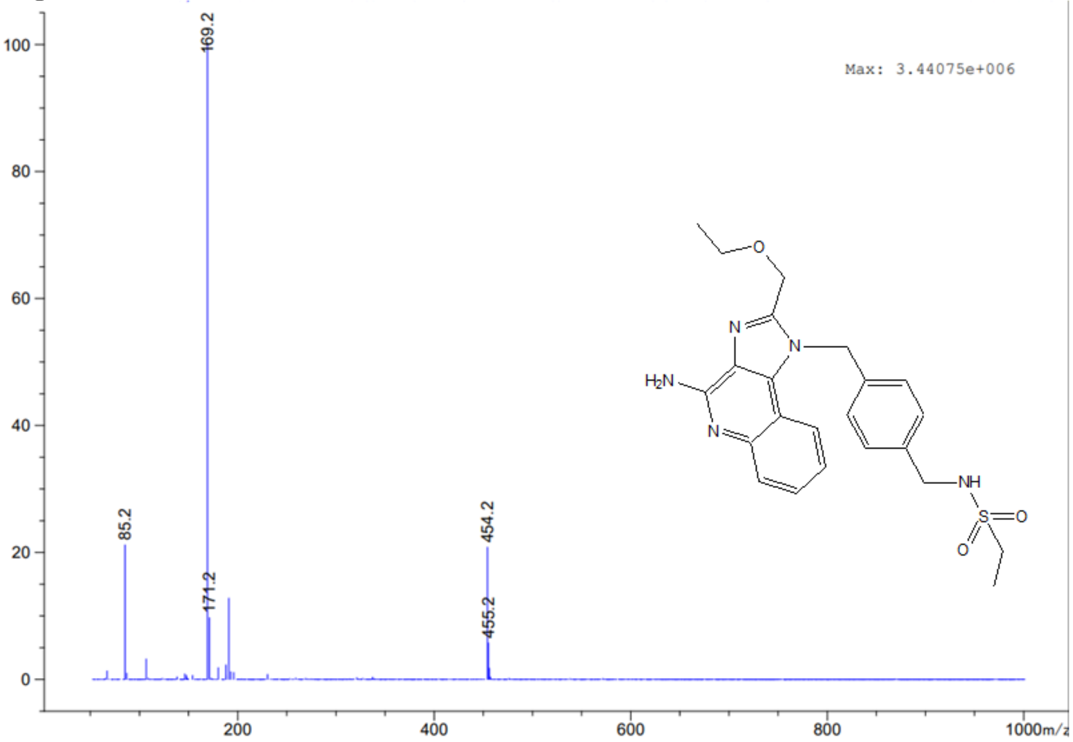

$[M+H]^+_{theoretical}$= 454.2 m/z, $[M+H]^+_{observed}$ = 454.2 m/z

Figure S72: ^{1}H NMR of **S3.**

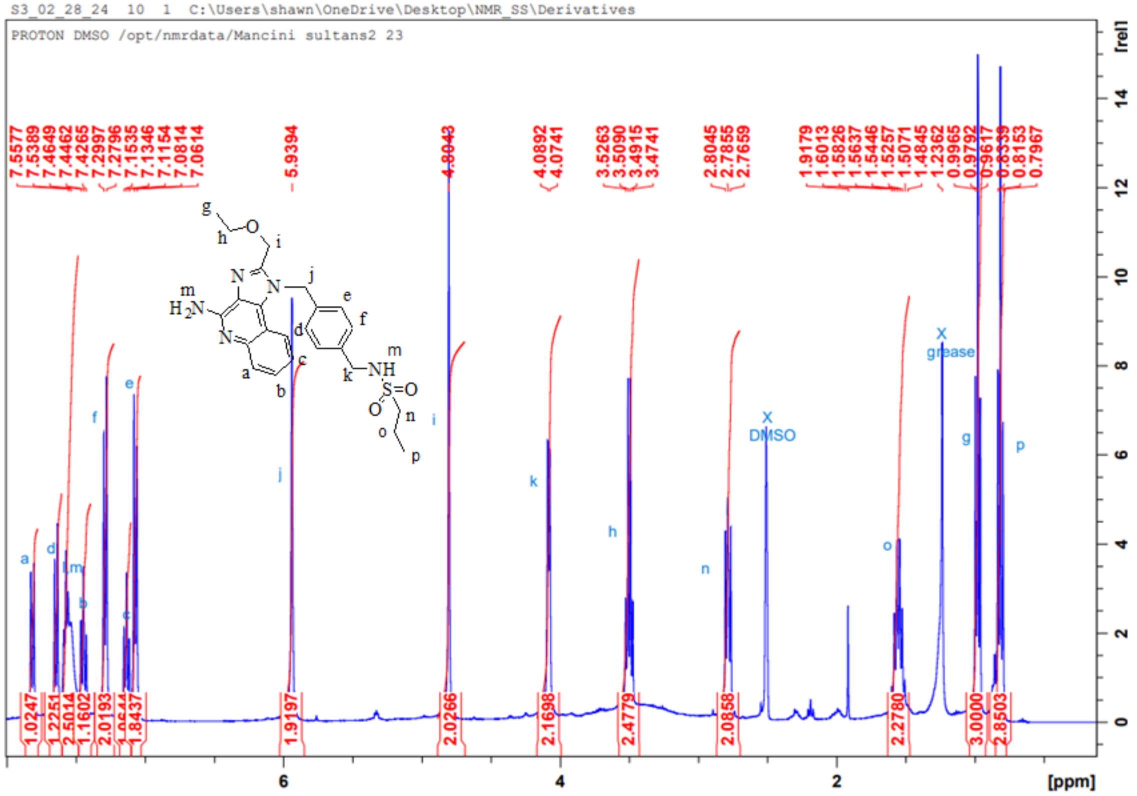

Figure S73: ^{13}C NMR of **S3.**

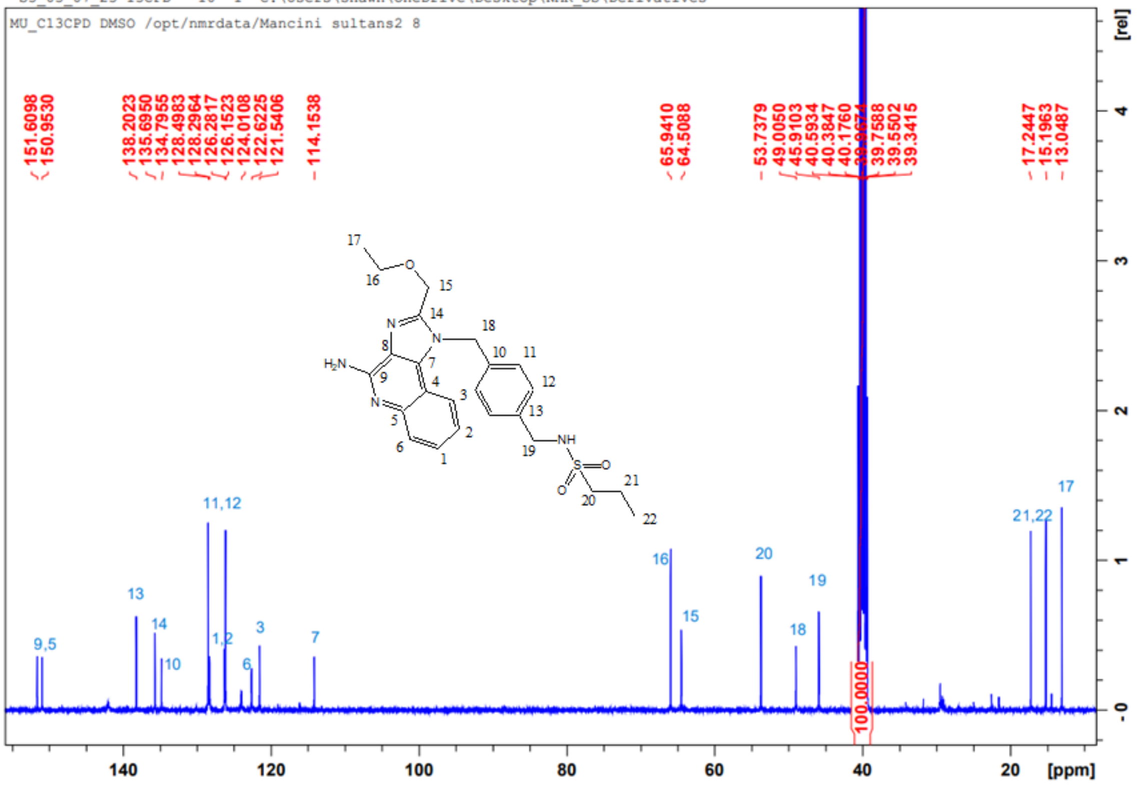

Figure S74: ESI/MS of **S3.**

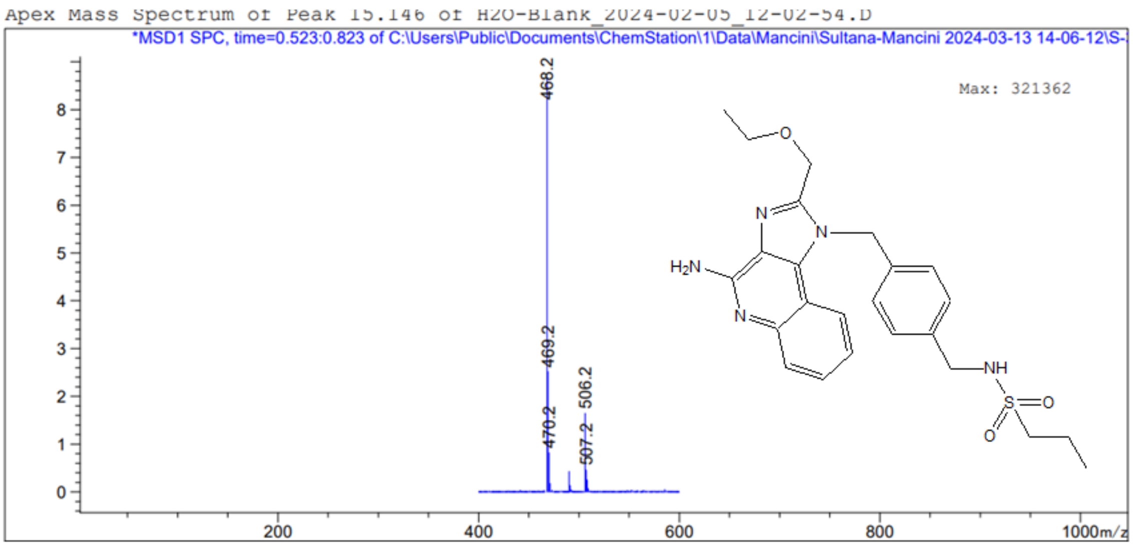

$[M+H]^+_{theoretical}$= 468.2 m/z, $[M+H]^+_{observed}$ = 468.2 m/z

Figure S75: HPLC of **S3.** Sample was loaded in a minimal amount of DMSO in MeOH. Detection is at 250nm over 20 minutes using a gradient mobile phase of Water / ACN and 0.1% formic acid.

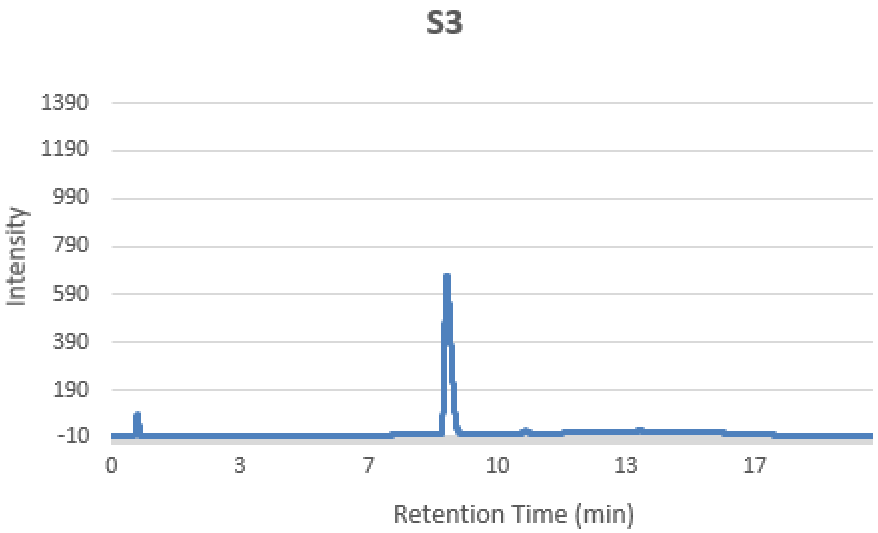

Figure S76: ^{1}H NMR of **S4.**

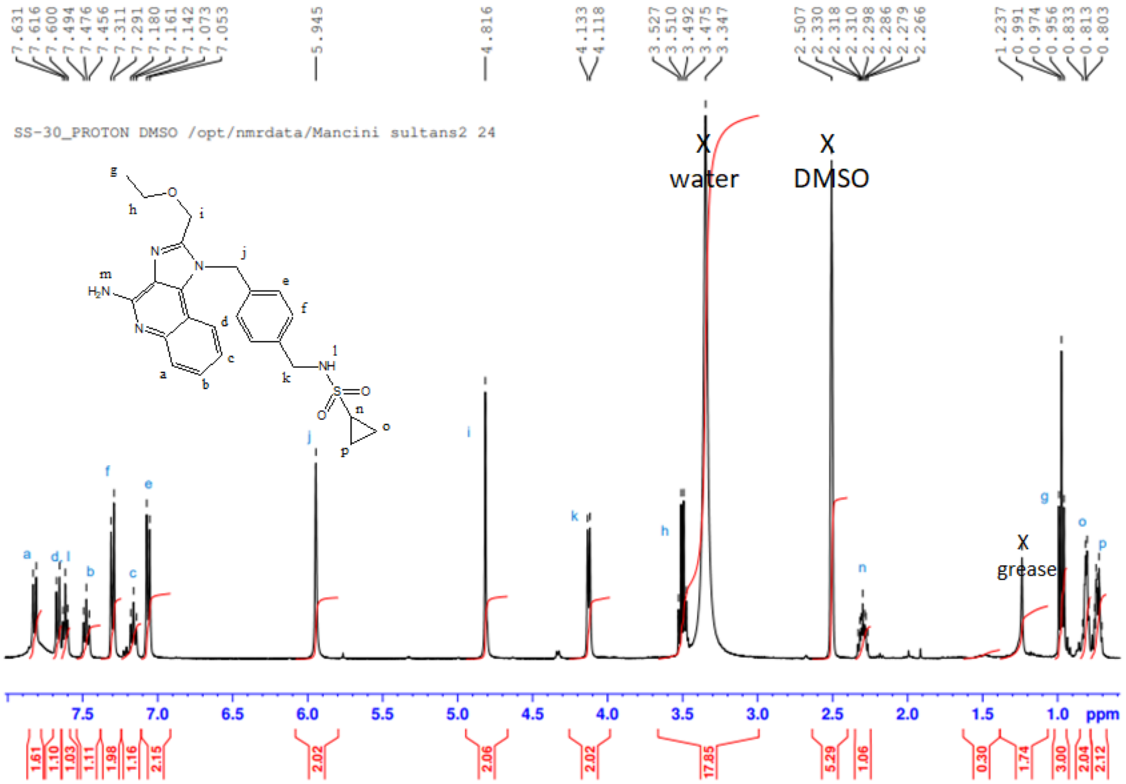

Figure S77: ^{13}C NMR of **S4.**

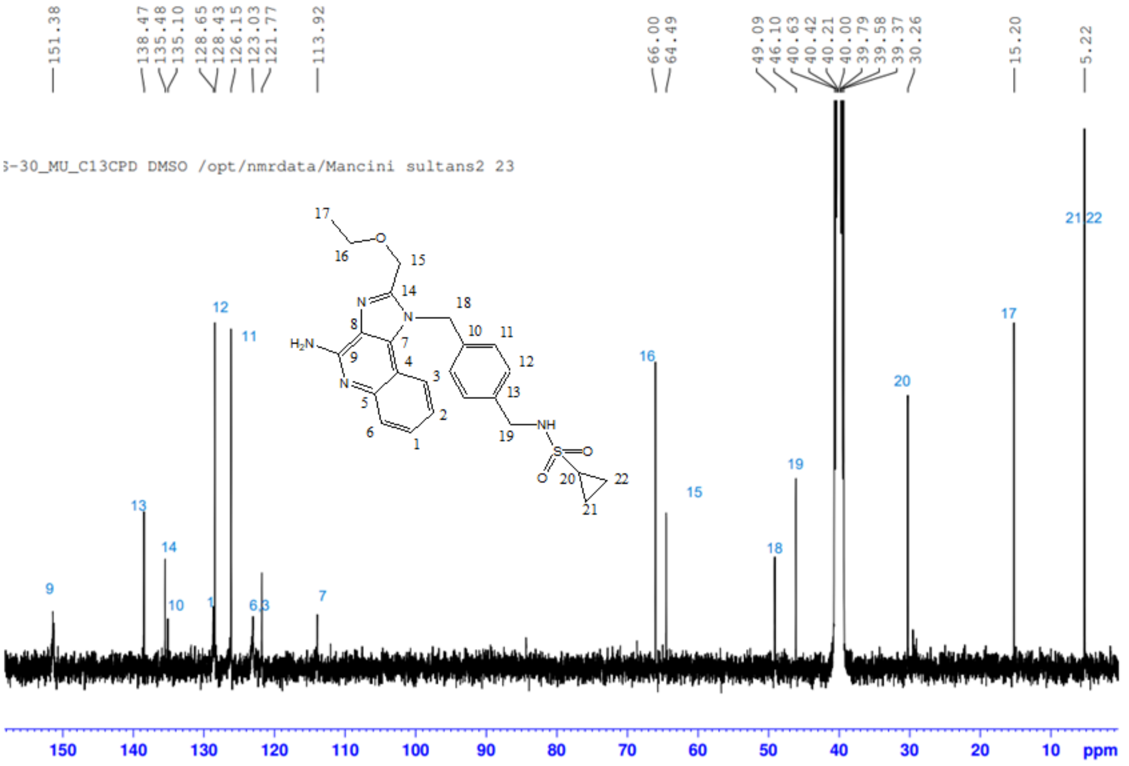

Figure S78: ESI/MS of **S4.**

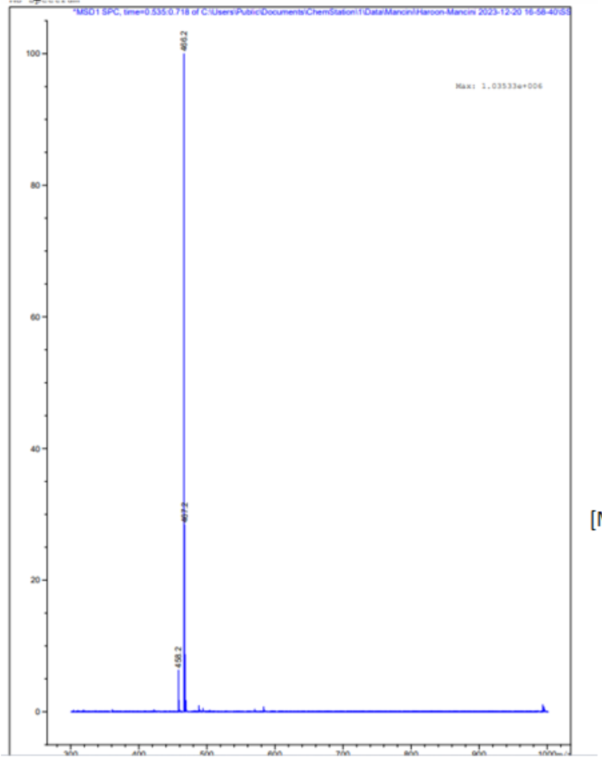

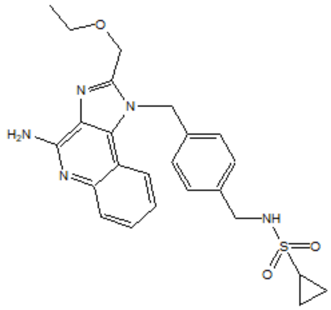

$[M+H]^+_{theoretical}$= 466.2 m/z, $[M+H]^+_{observed}$ = 466.2 m/z

Figure S79: ^{1}H NMR of **S5.**

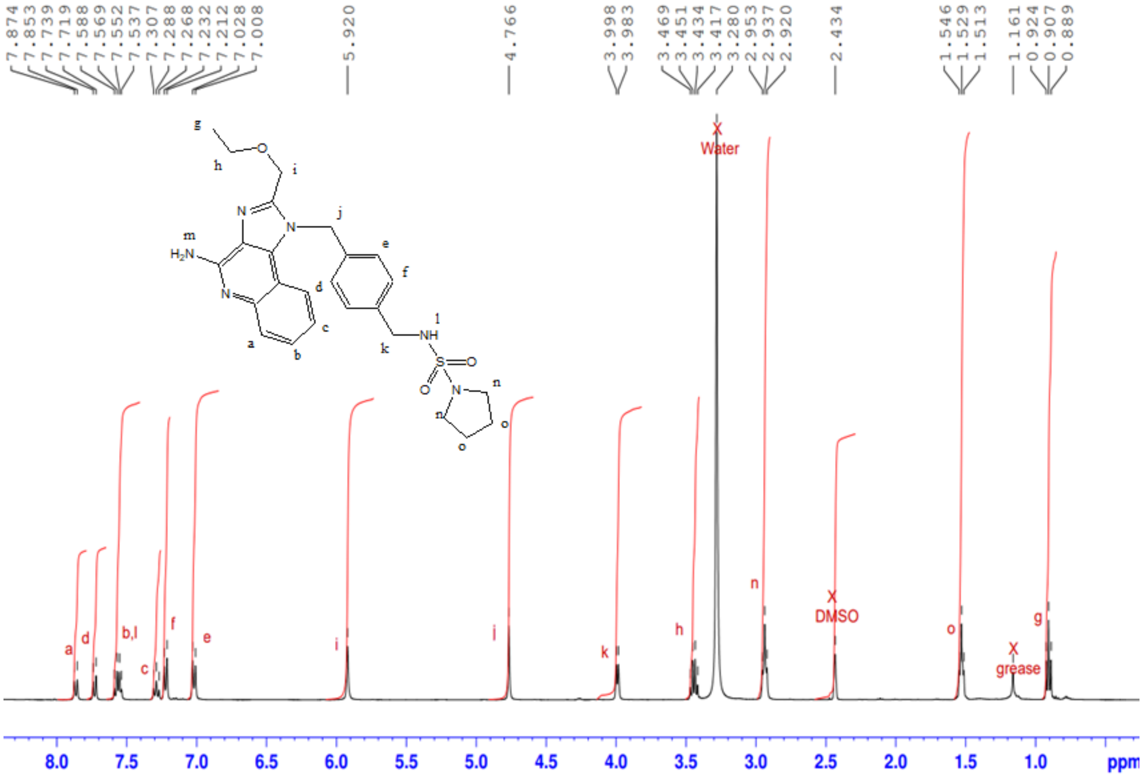

Figure S80: ^{13}C NMR of **S5.**

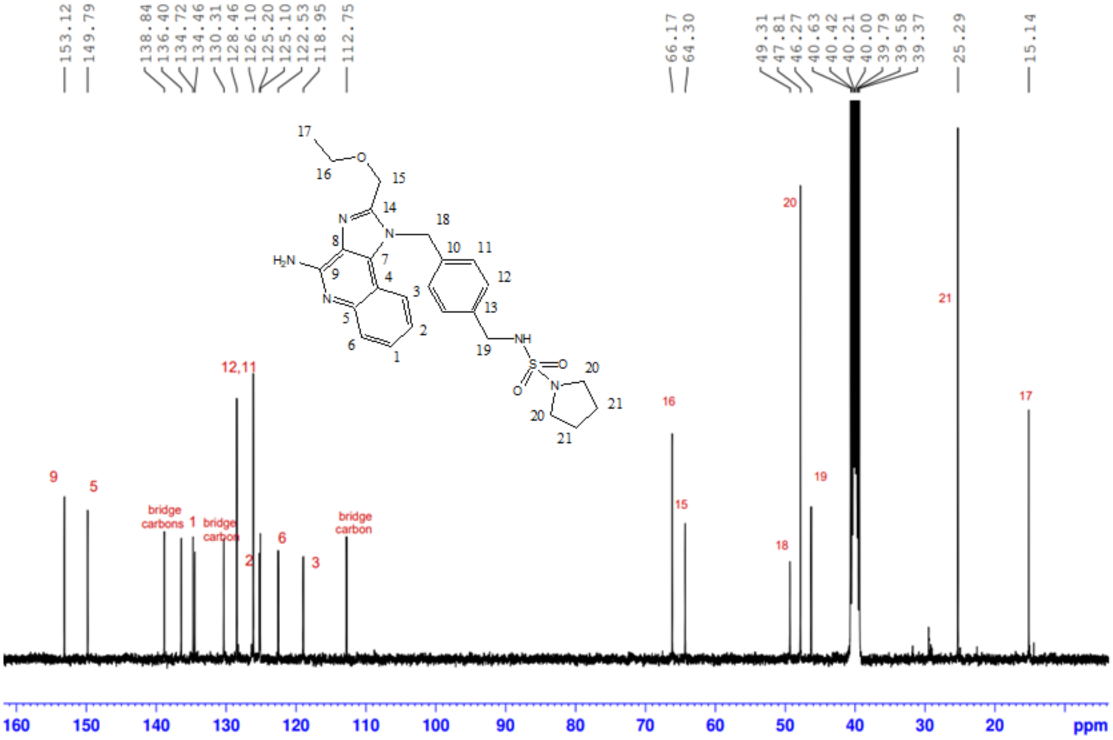

Figure S81: ESI/MS of **S5.**

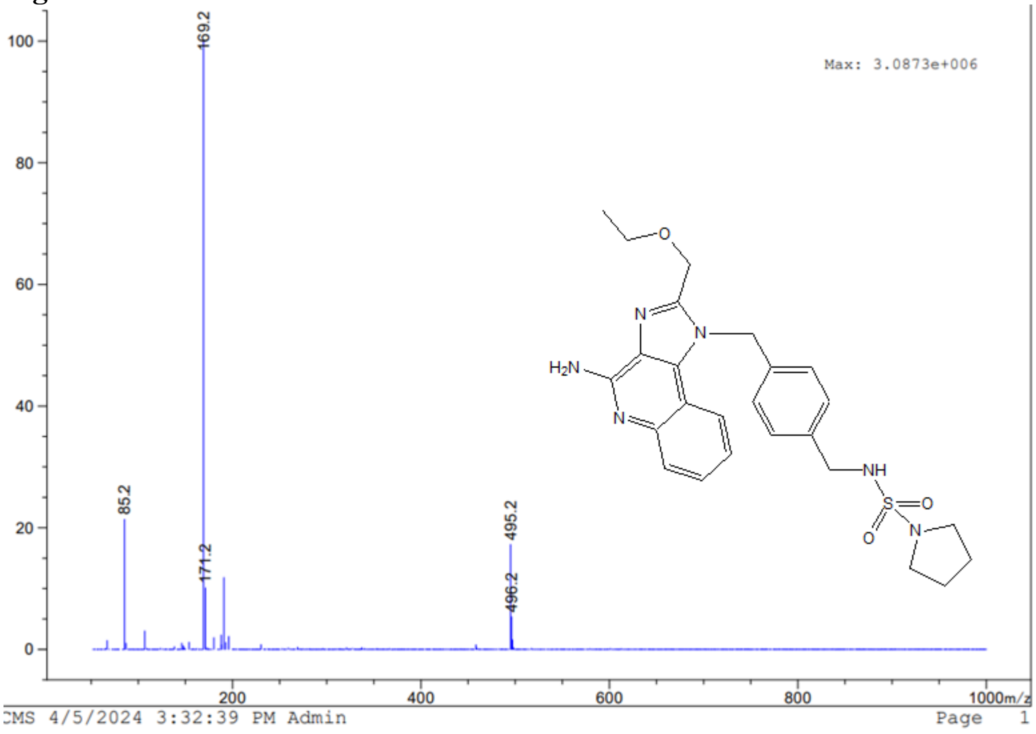

$[M+H]^{+}_{theoretical}$ = 495.2 m/z, $[M+H]^{+}_{observed}$ = 495.2 m/z

Figure S82: HPLC of **S5.** Sample was loaded in a minimal amount of DMSO in MeOH. Detection is at 250nm over 20 minutes using a gradient mobile phase of Water / ACN and 0.1% formic acid.

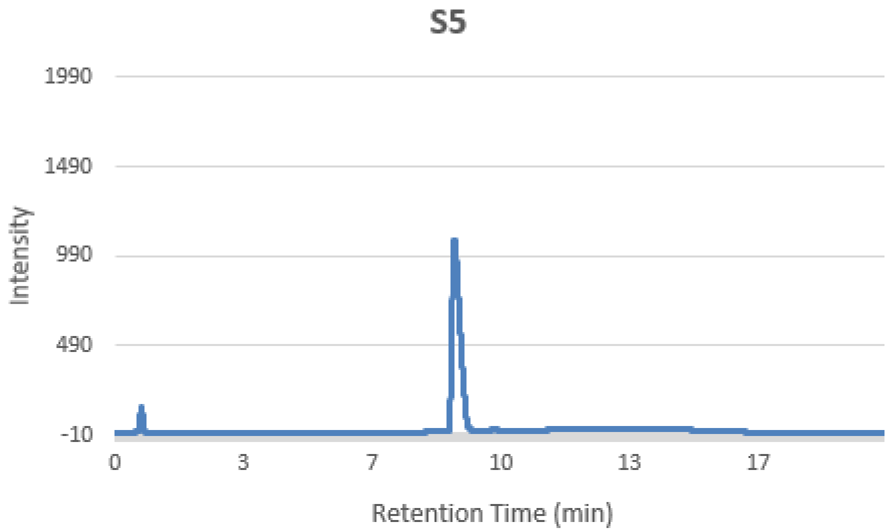

Figure S83: ^{1}H NMR of **S6**

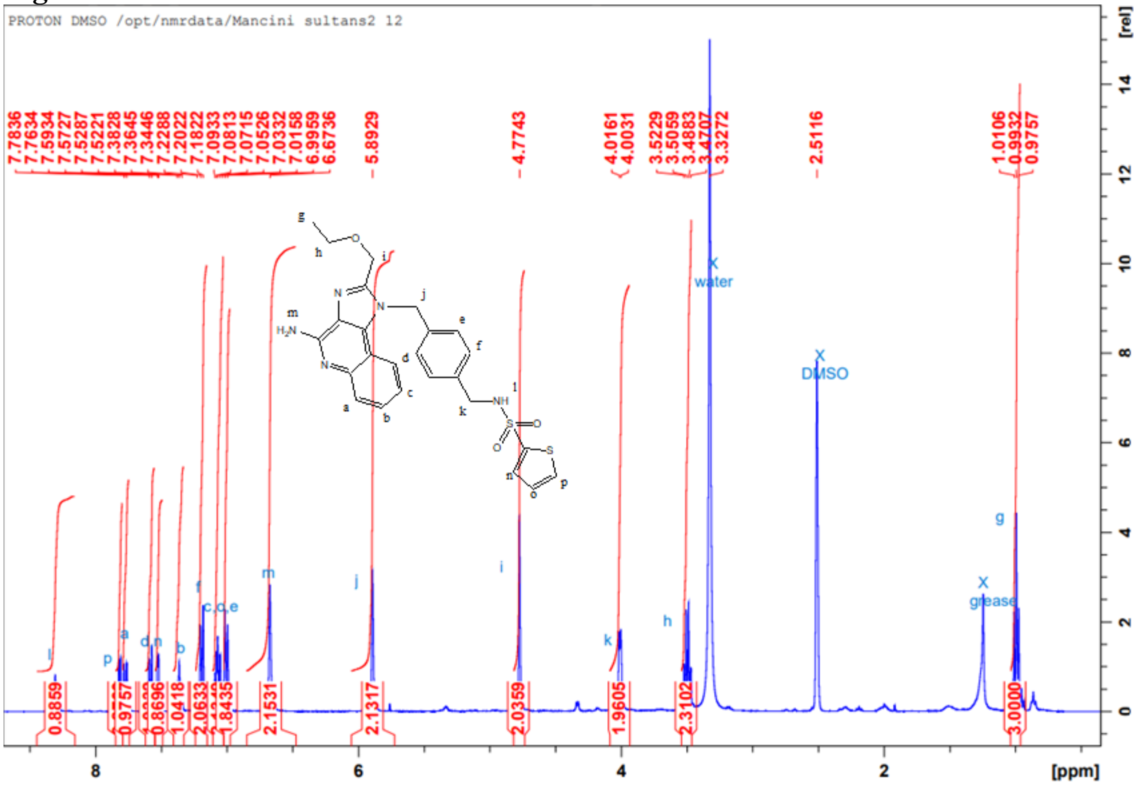

Figure S84: ^{13}C NMR of **S6.**

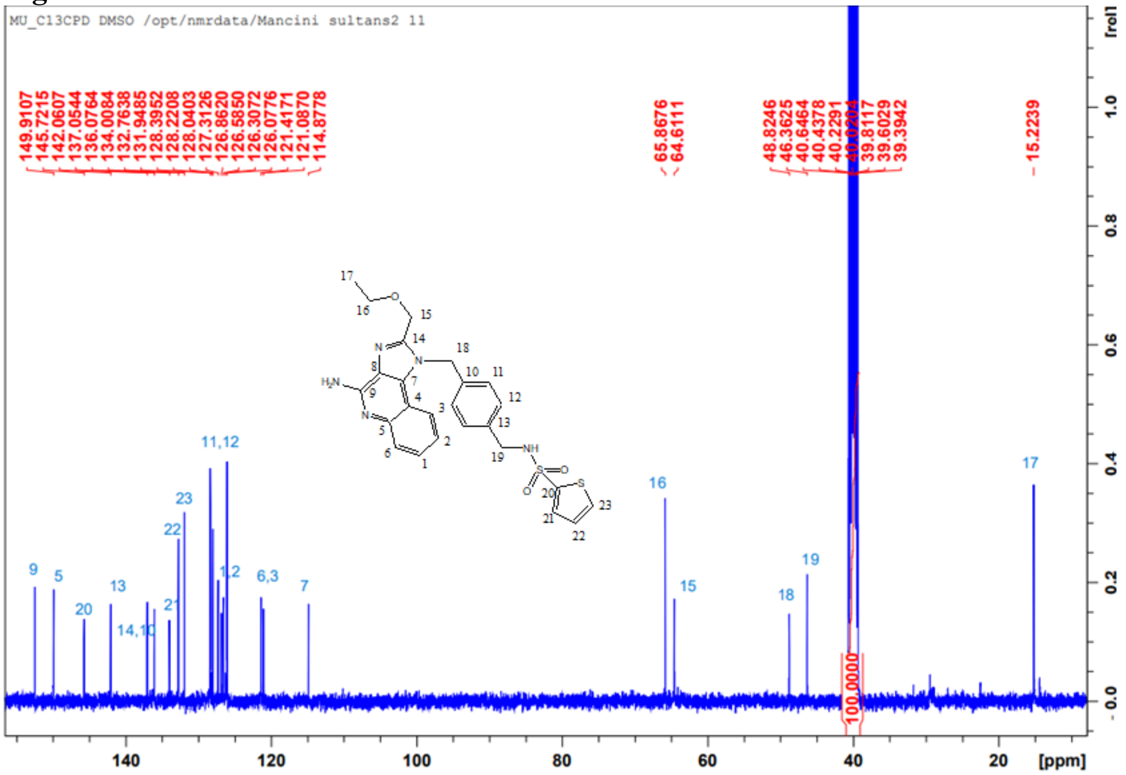

Figure S85: ESI/MS of **S6.**

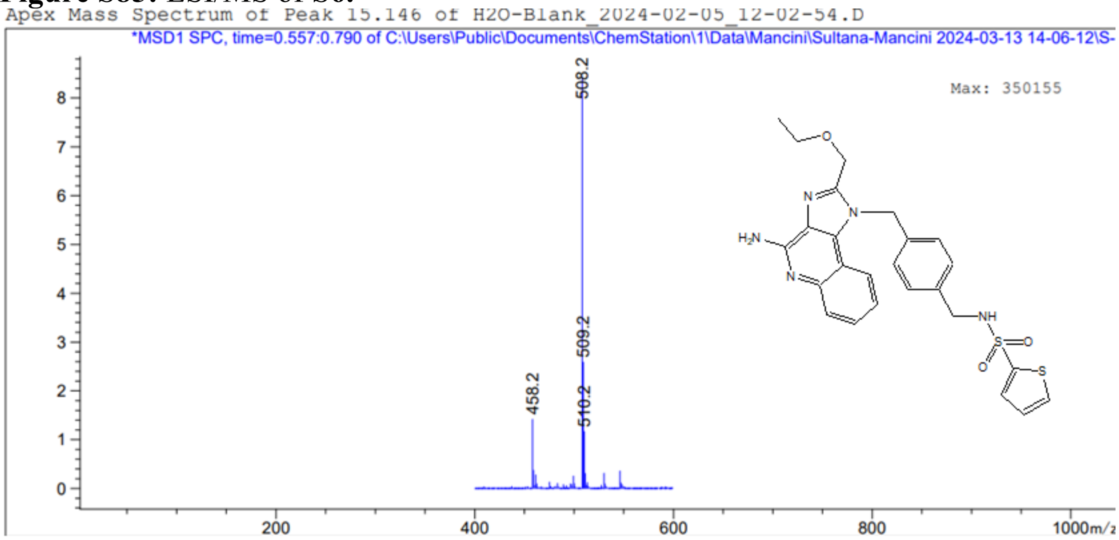

$[M+H]^+_{theoretical}$ = 508.2 m/z, $[M+H]^+_{observed}$ = 508.2 m/z

Figure S86: ^{1}H NMR of **S7.**

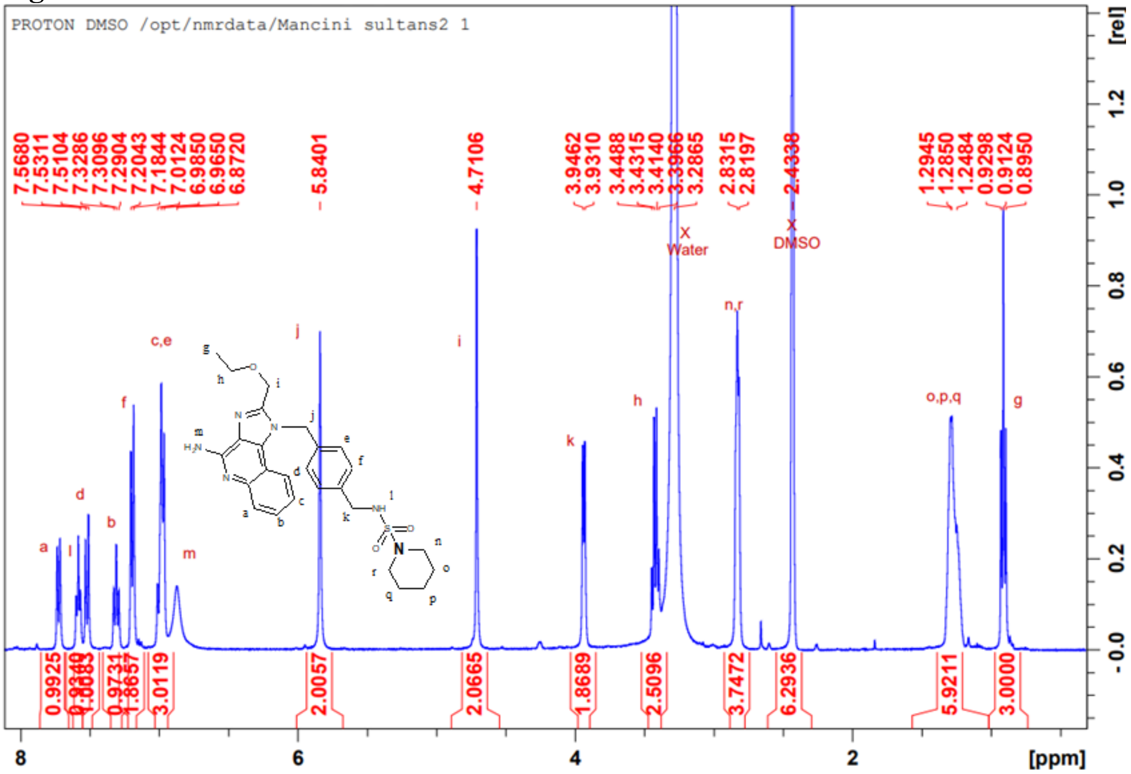

Figure S87: ^{13}C NMR of **S7.**

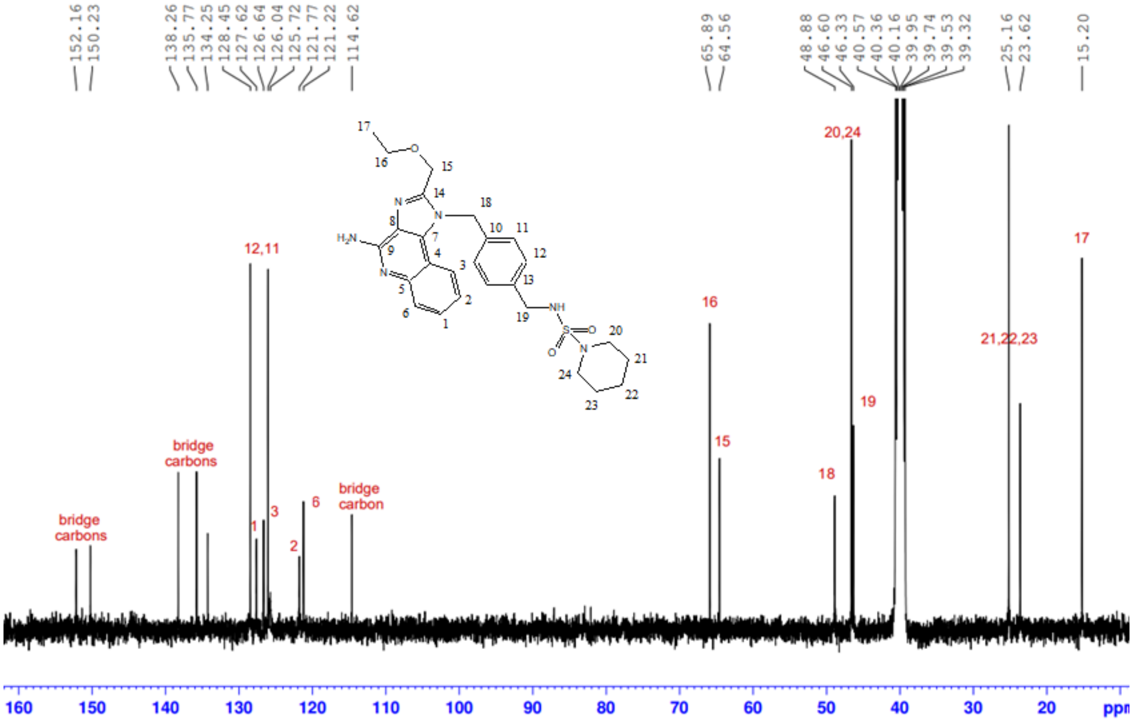

Figure S88: ESI/MS of **S7.**

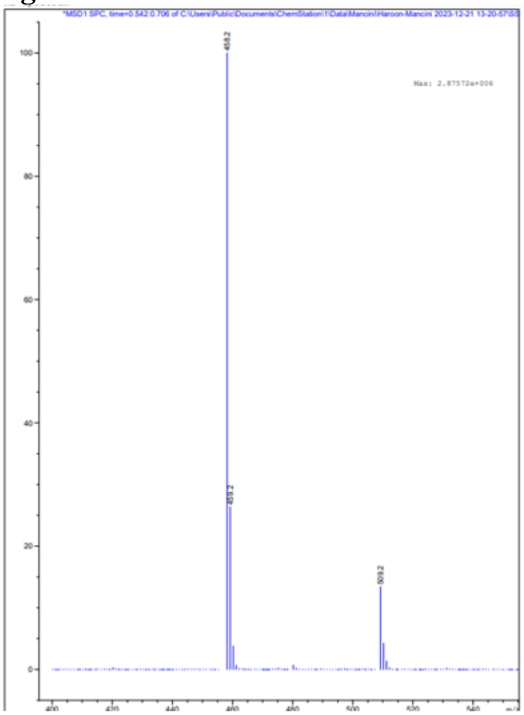

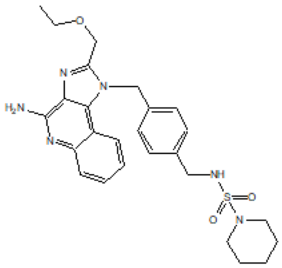

$[M+H]^+_{theoretical}$= 509.2 m/z, $[M+H]^+_{observed}$ = 509.2 m/z

Figure S89: ^{1}H NMR of **S8.**

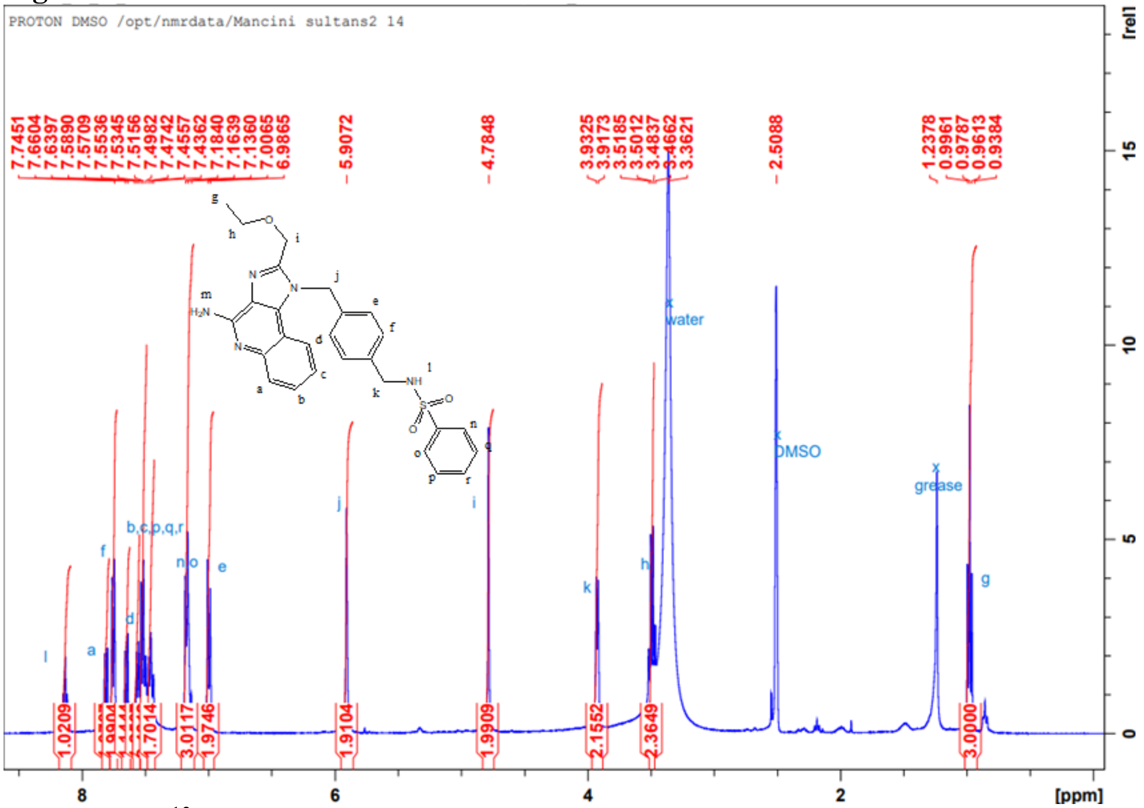

Figure S90: ^{13}C NMR of **S8.**

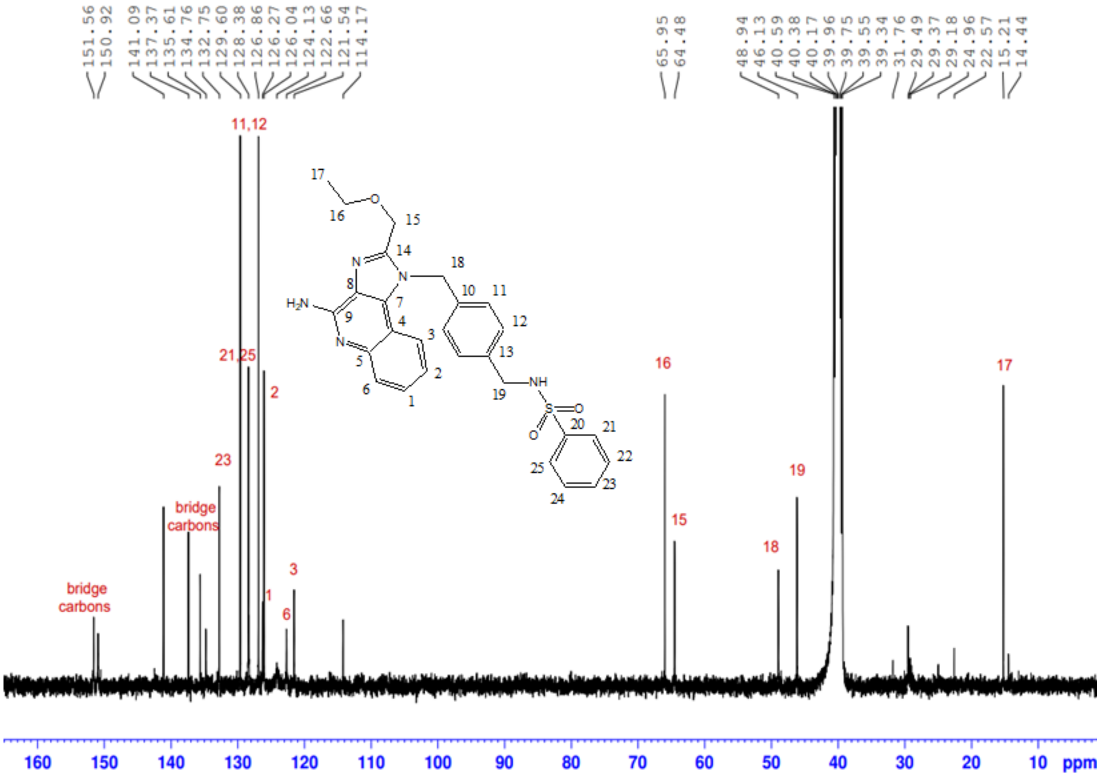

Figure S91: ESI/MS of **S8.**

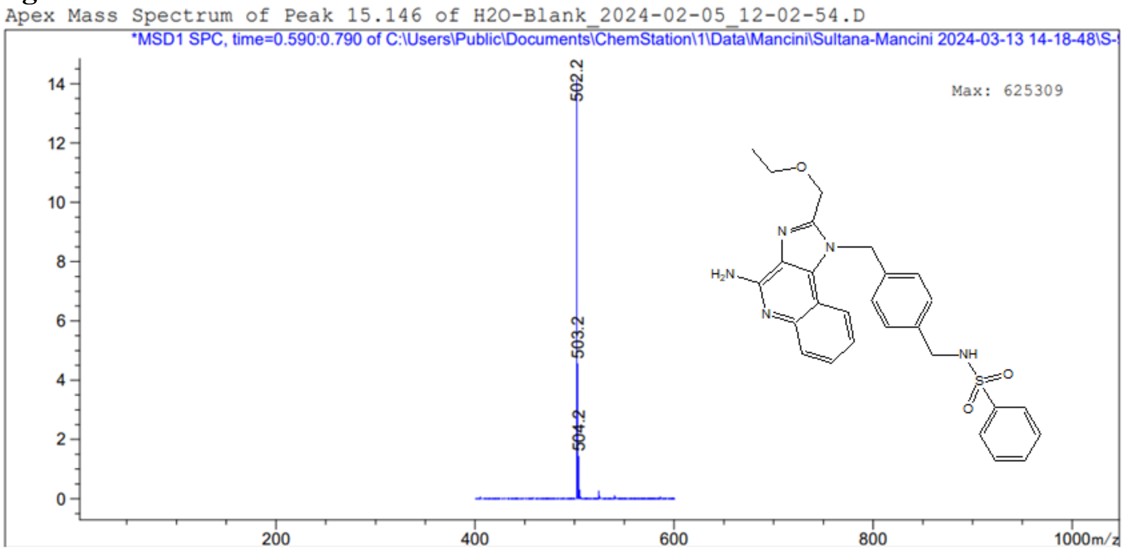

$[M+H]^+_{theoretical}$= 502.2 m/z, $[M+H]^+_{observed}$ = 502.2 m/z

Figure S92: ^{1}H NMR of **S9.**

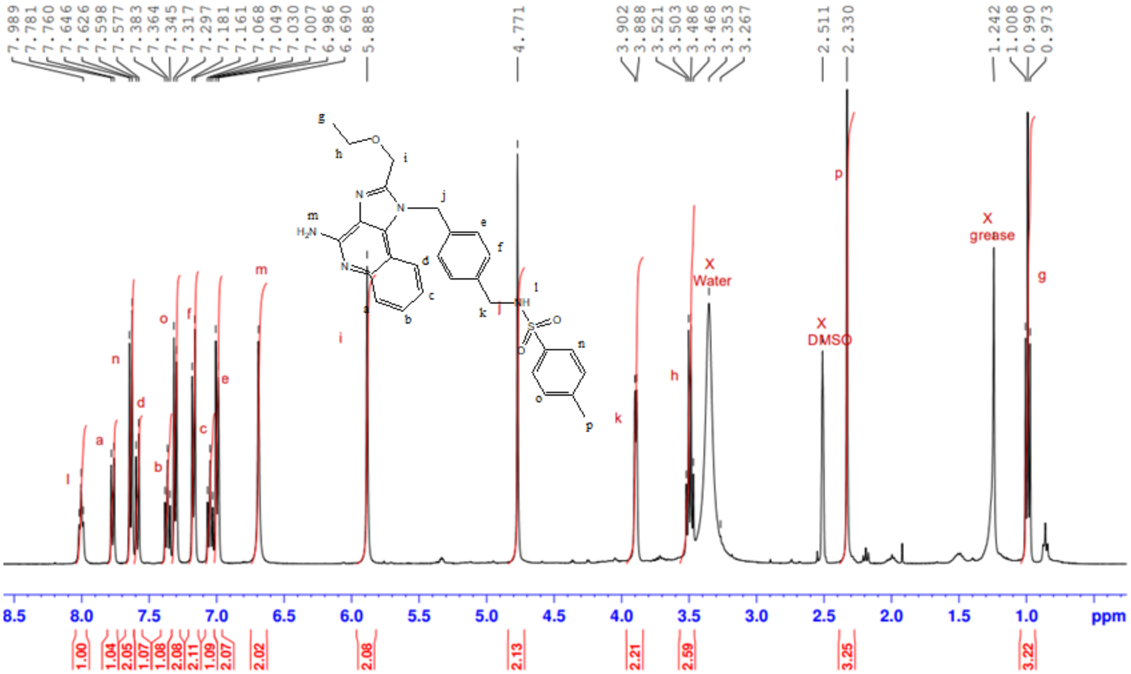

Figure S93: ^{13}C NMR of **S9.**

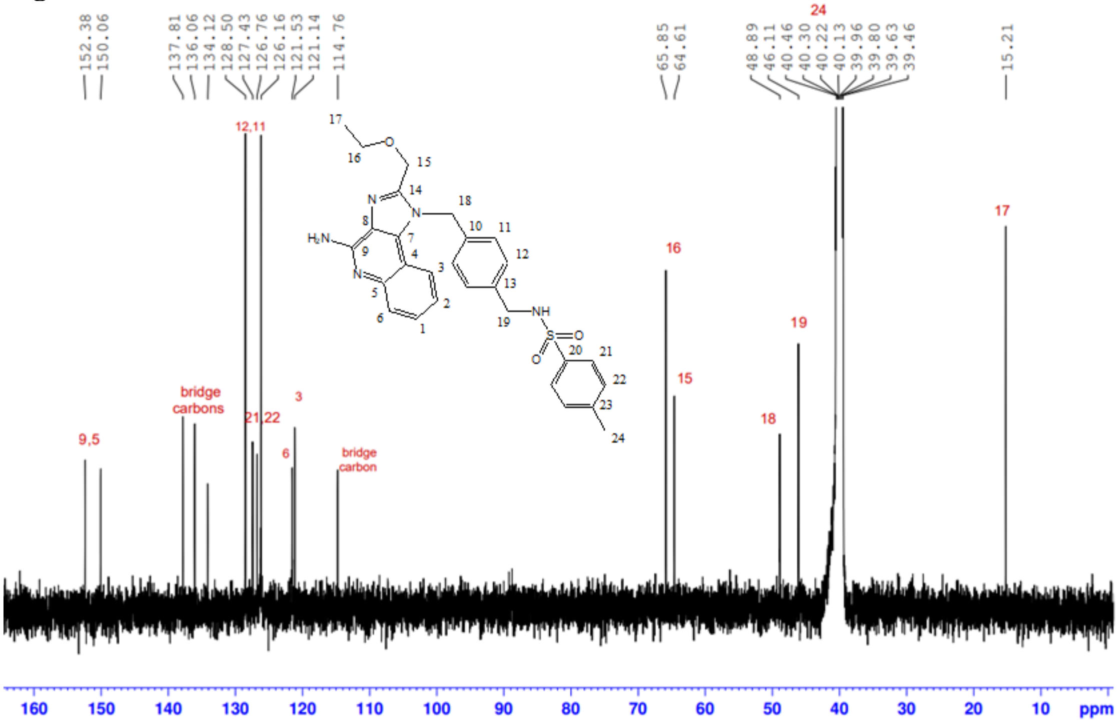

Figure S94: ESI/MS of **S9.**

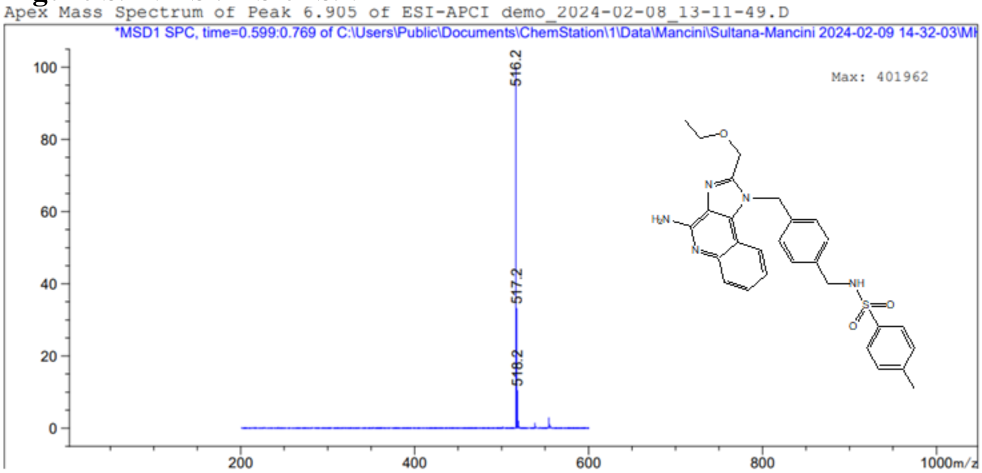

$[M+H]^+_{theoretical}$= 516.2 m/z, $[M+H]^+_{observed}$ = 516.2 m/z

Figure S95: ^{1}H NMR of **S10.**

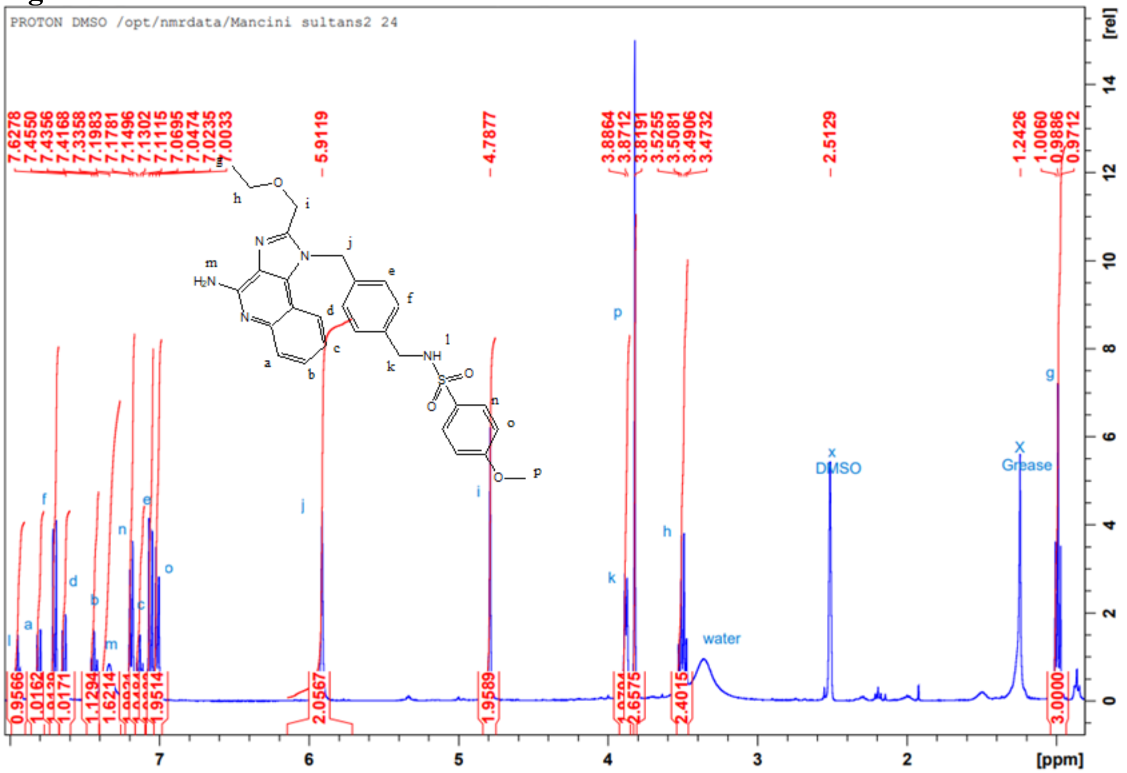

Figure S96: ^{13}C NMR of **S10.**

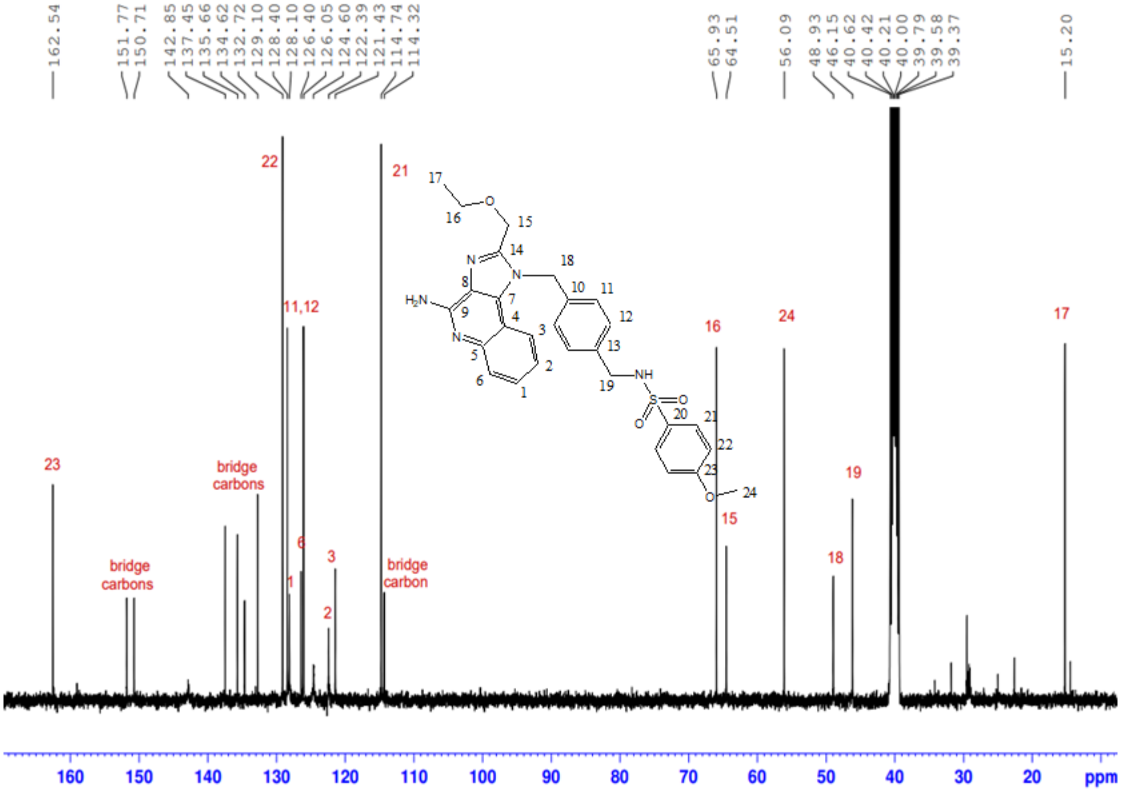

Figure S97: ESI/MS of **S10.**

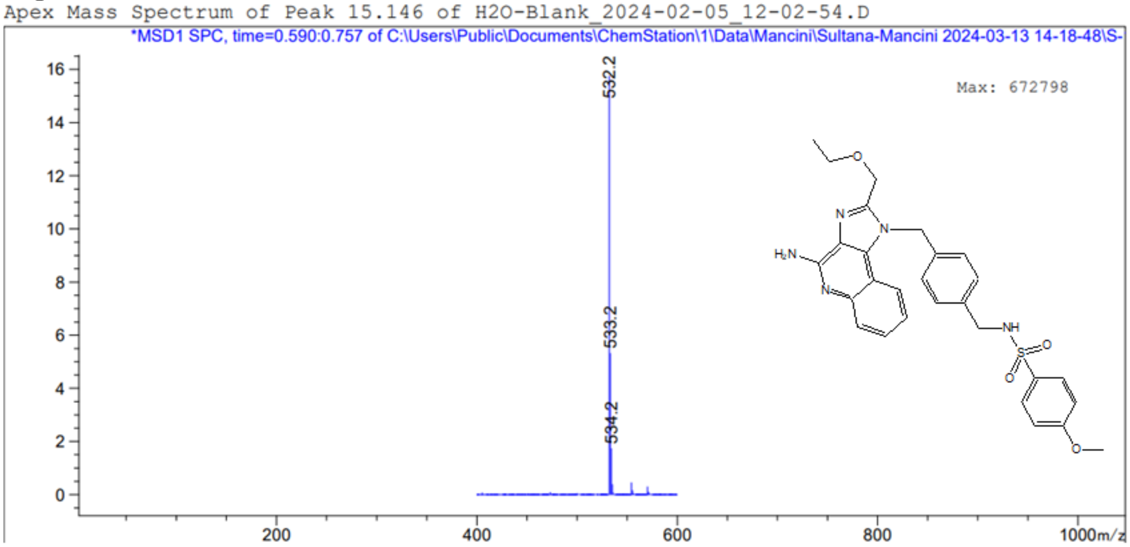

$[M+H]^+_{theoretical}$= 532.2 m/z, $[M+H]^+_{observed}$ = 532.2 m/z

Figure S98: ^{1}H NMR of **S11.**

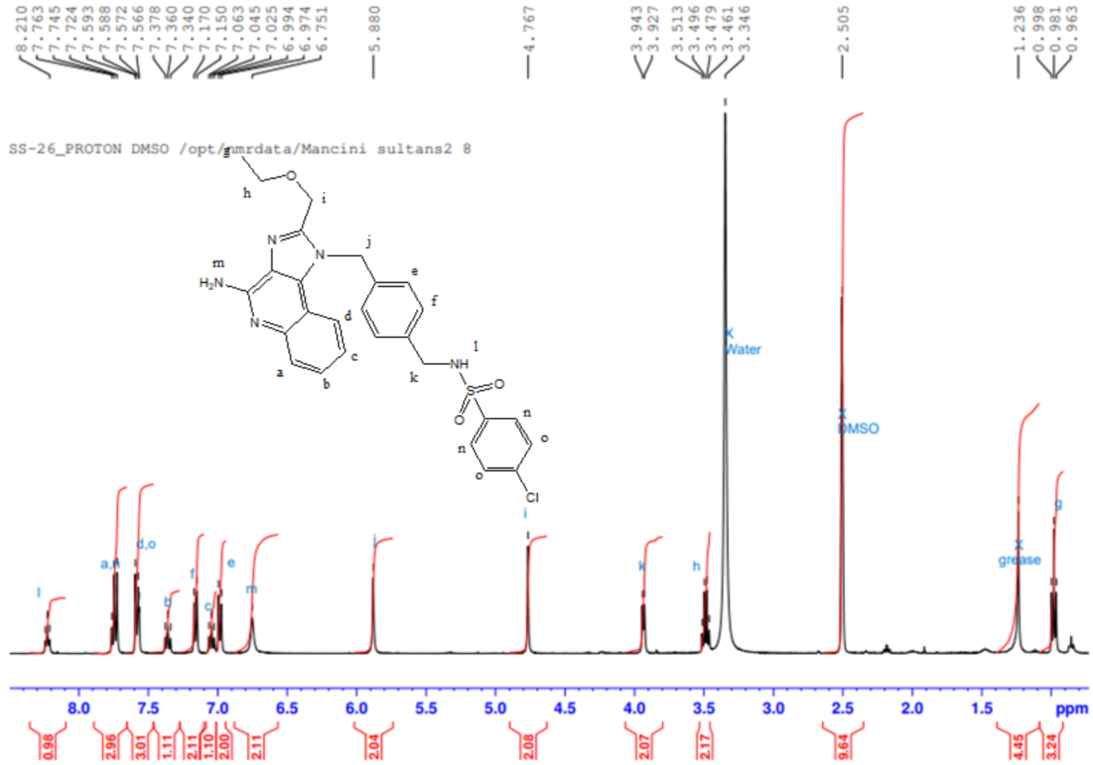

Figure S99: ^{13}C NMR of **S11.**

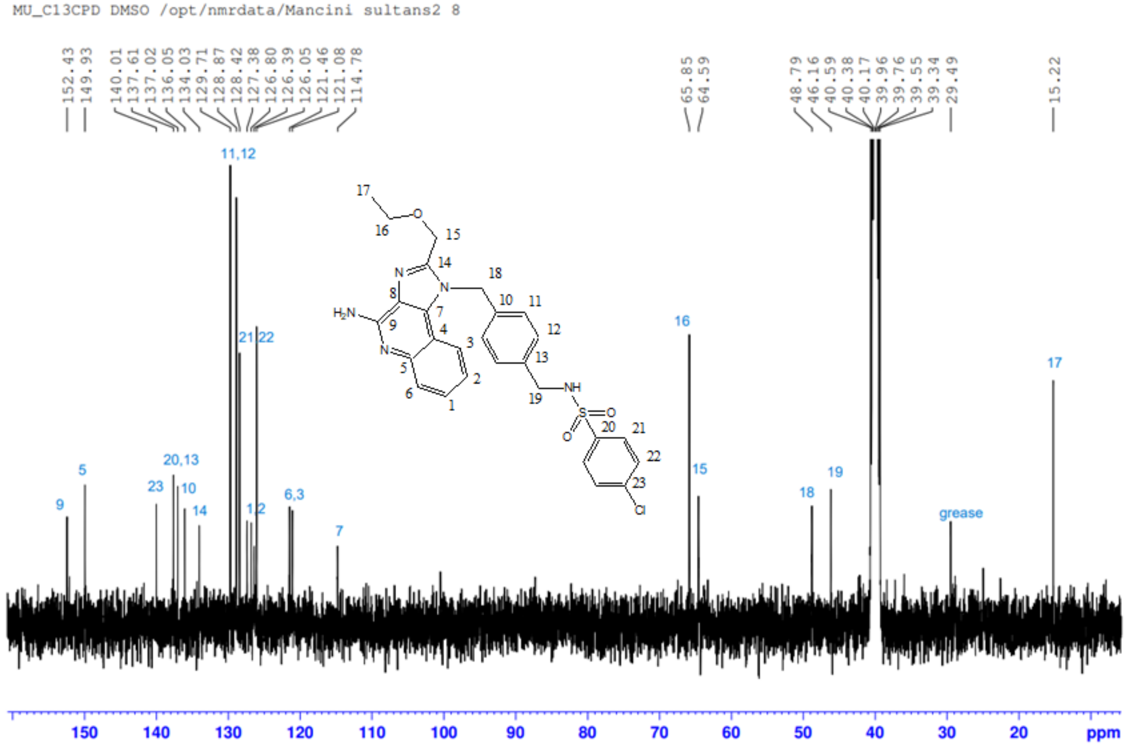

Figure S100: ESI/MS of **S11.**

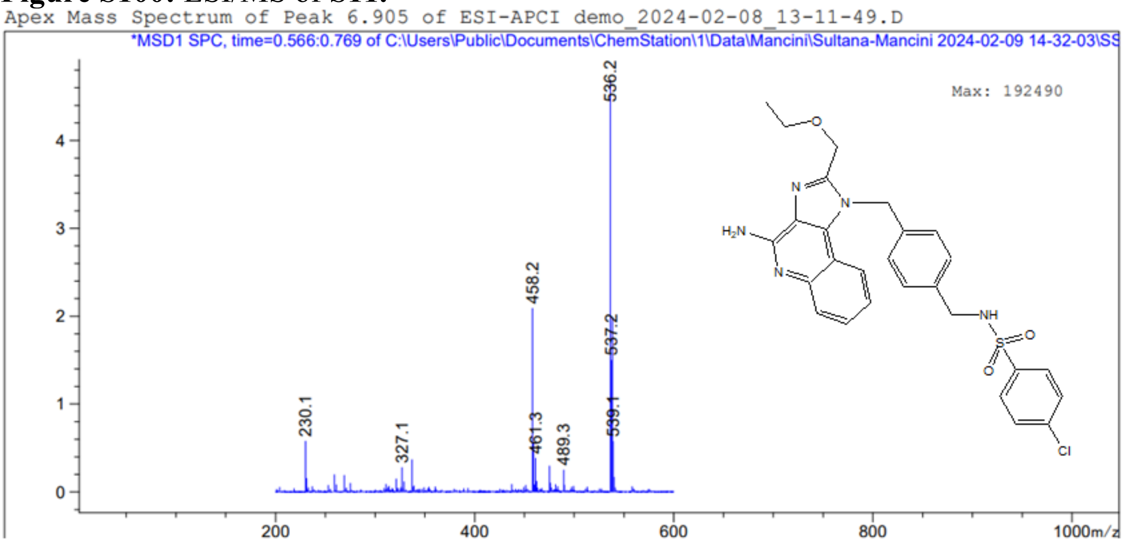

[M+H]$^{+}_{theoretical}$= 536.2 m/z, [M+H]$^{+}_{observed}$ = 536.2 m/z

Figure S101: ^{1}H NMR of **S12.**

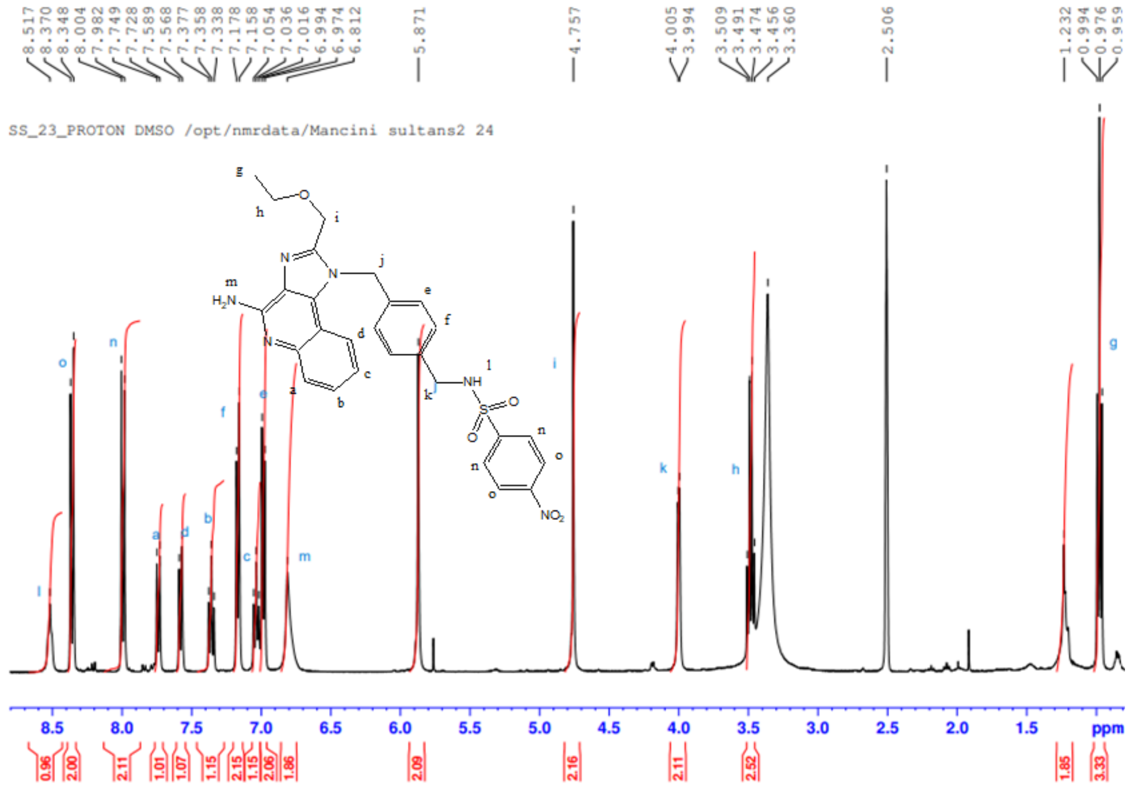

Figure S102: ^{13}C NMR of **S12.**

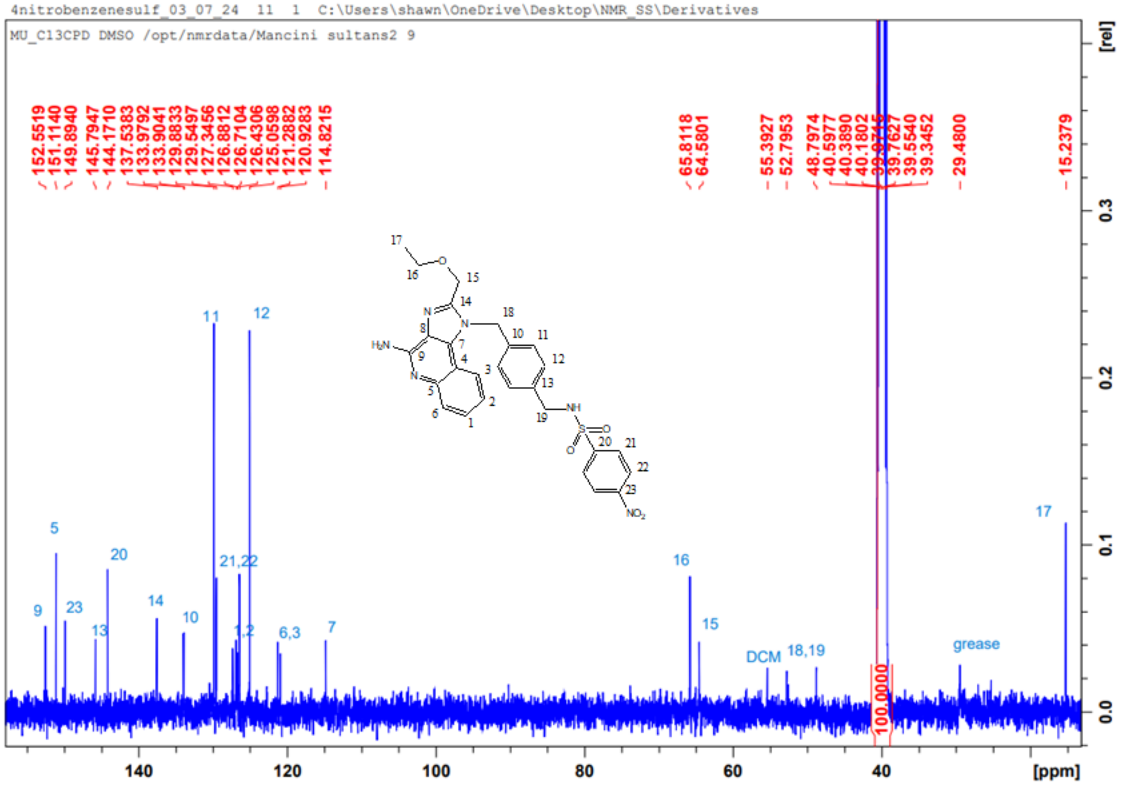

Figure S103: ESI/MS of **S12.**

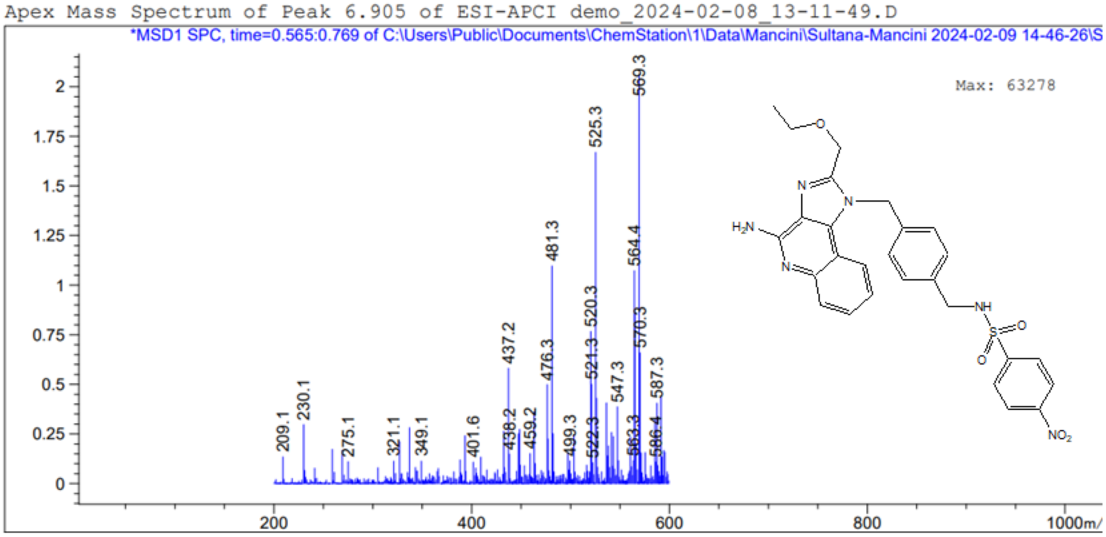

$[M+H]^+_{theoretical}$: 547.2, $[M+H]^+_{found}$: 547.3, $[M+Na]^+_{theoretical}$= 569.3 m/z, $[M+Na]^+_{observed}$ = 569.3 m/z

Figure S104: ^{1}H NMR of **S13.**

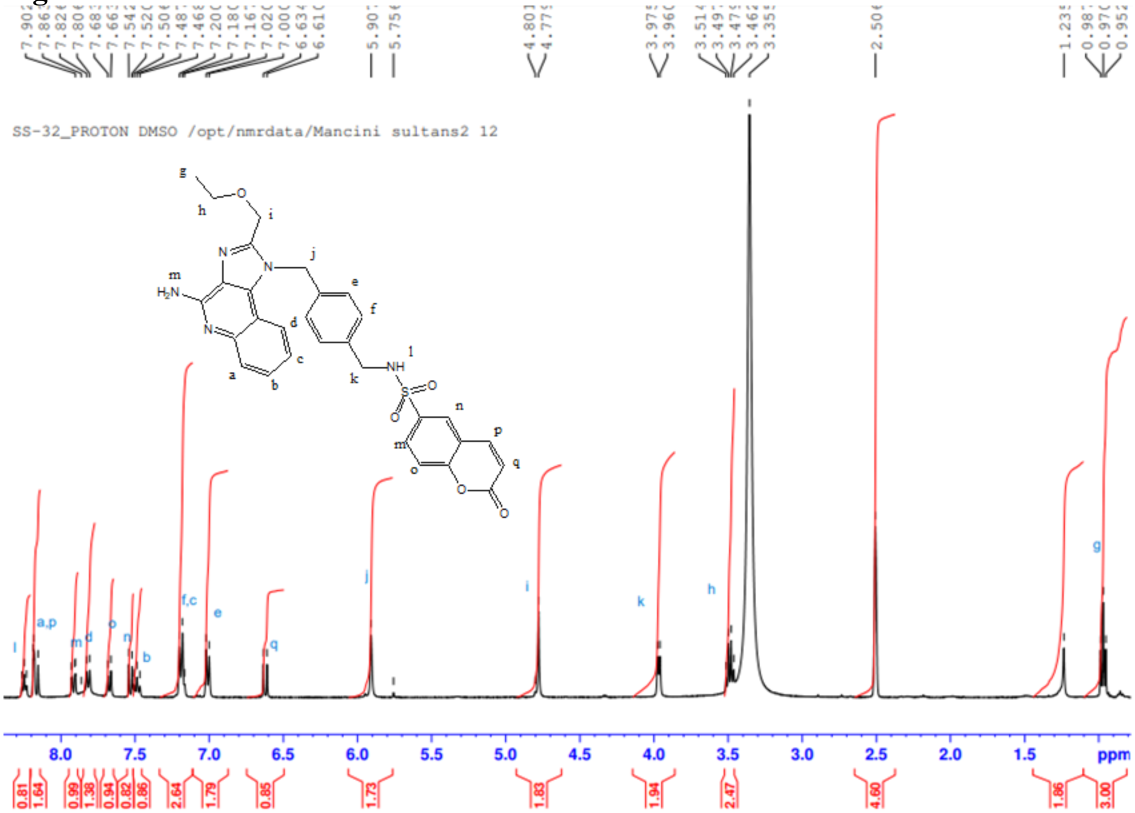

Figure S105: ^{13}C NMR of **S13.**

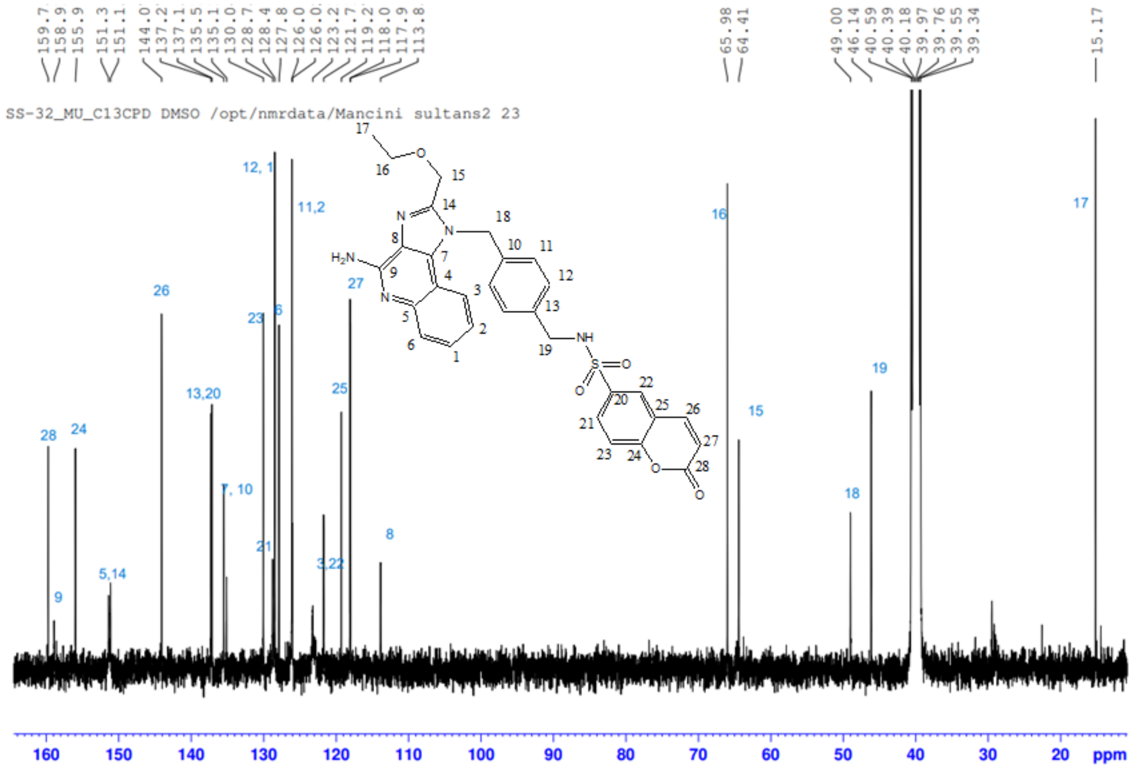

Figure S106: ESI/MS of **S13.**

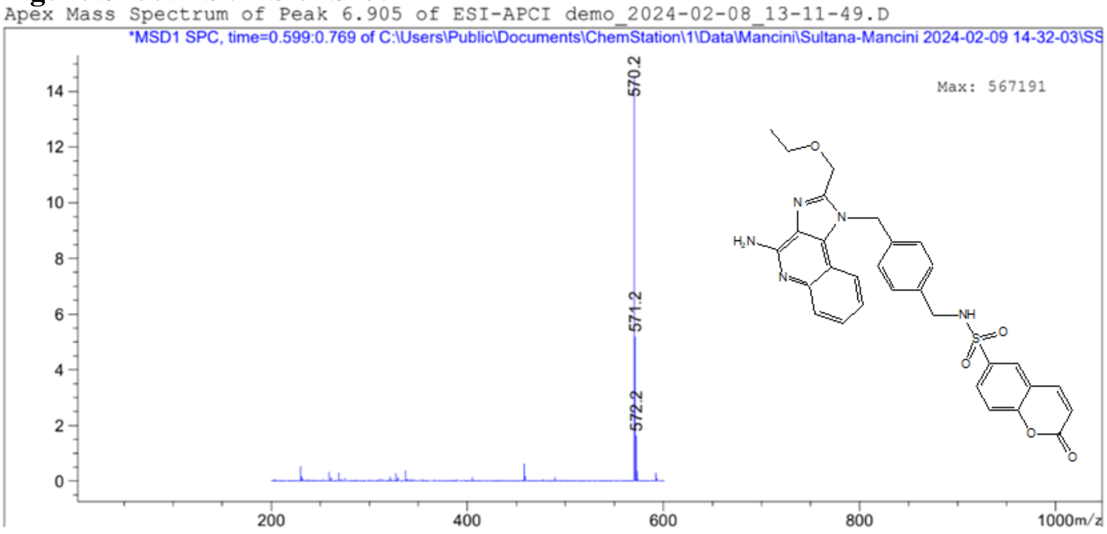

$[M+H]^+_{theoretical}$= 570.2 m/z, $[M+H]^+_{observed}$ = 570.2 m/z

References

(1) Alvim Jr., J.; Dias, R. L. A.; Castilho, M. S.; Oliva, G.; Corrêa, A. G. Preparation and Evaluation of a Coumarin Library towards the Inhibitory Activity of the Enzyme gGAPDH from Trypanosoma Cruzi. *J. Braz. Chem. Soc.* **2005**, *16* (4), 763–773. https://doi.org/10.1590/S0103-50532005000500014.

(2) Yao, H.; Wei, G.; Liu, Y.; Yao, H.; Zhu, Z.; Ye, W.; Wu, X.; Xu, J.; Xu, S. Synthesis, Biological Evaluation of Fluorescent 23-Hydroxybetulinic Acid Probes, and Their Cellular Localization Studies. *ACS Med. Chem. Lett.* **2018**, *9* (10), 1030–1034. https://doi.org/10.1021/acsmedchemlett.8b00321.

(3) Eken, G. A.; Huang, Y.; Guo, Y.; Ober, C. Visualization of pH Response through Autofluorescent Poly(Styrene-Alt-N-Maleimide)Polyelectrolyte Brushes. *ACS Appl Polym Mater* **2023**, *5* (2), 1613–1623.

(4) Reddy, P. Y. Efficient Synthesis of Fluorophore-Linked Maleimide Derivatives. *Synthesis* **1998**, *1998* (07), 999–1002. https://doi.org/10.1055/s-1998-2097.
